# Structures of Naturally Evolved *CUP1* Tandem Arrays in Yeast Indicate That These Arrays Are Generated by Unequal Nonhomologous Recombination

**DOI:** 10.1534/g3.114.012922

**Published:** 2014-09-17

**Authors:** Ying Zhao, Pooja K. Strope, Stanislav G. Kozmin, John H. McCusker, Fred S. Dietrich, Robert J. Kokoska, Thomas D. Petes

**Affiliations:** * Department of Molecular Genetics and Microbiology and University Program in Genetics and Genomics, Duke University Medical Center, Durham, North Carolina 27710; † Physical Sciences Directorate, U. S. Army Research Office, Research Triangle Park, North Carolina 27709

**Keywords:** copper resistance, tandemly repeated genes, recombination, *Saccharomyces cerevisiae*

## Abstract

An important issue in genome evolution is the mechanism by which tandem duplications are generated from single-copy genes. In the yeast *Saccharomyces cerevisiae*, most strains contain tandemly duplicated copies of *CUP1*, a gene that encodes a copper-binding metallothionein. By screening 101 natural isolates of *S. cerevisiae*, we identified five different types of *CUP1*-containing repeats, as well as strains that only had one copy of *CUP1*. A comparison of the DNA sequences of these strains indicates that the *CUP1* tandem arrays were generated by unequal nonhomologous recombination events from strains that had one *CUP1* gene.

One common and important source of genomic alterations is copy-number variation (CNV). The term is often used to describe a variant genome that contains deletions or duplications of sequences (100 bp to 1 Mb) relative to a standard genome. The rate of CNV formation in human cells is about 2 × 10^−6^ to 10^−4^ per generation per locus (Zhang *et al.* 2009). In comparison, the rate of point mutations in human cells is about 2 × 10^−8^/bp/generation (Zhang *et al.* 2009). Here we will emphasize those CNV events that affect alterations in a pre-existing tandem array or that generate tandem duplications of single-copy genomic sequences.

In a pre-existing tandem array, one mechanism that can alter the number of repeats is unequal crossovers (Figure 1A), a mechanism first demonstrated in studies of Bar eye *in Drosophila* almost 100 years ago (Sturtevant and Morgan 1923). As shown in the figure, homologous recombination between misaligned tandem arrays can result in both deletions and duplications of repeats. Unequal crossovers can occur between sister chromatids (as shown) or between homologs. Deletions also can be formed nonreciprocally by several other mechanisms, including “pop-outs” (in which an intrachromatid crossover produces a circular DNA molecule containing one or more repeats and a shorter tandem array), or by single-strand annealing (in which processing of a break within the tandem array followed by annealing of the broken ends deletes one or more repeats) (Ivanov *et al.* 1996). In yeast, unequal crossover events within the tandemly repeated ribosomal RNA genes are very frequent, occurring at a mitotic frequency of >10^−2^ per mitotic division (Szostak and Wu 1980) and >10^−1^ per meiotic division (Petes 1980).

**Figure 1:**
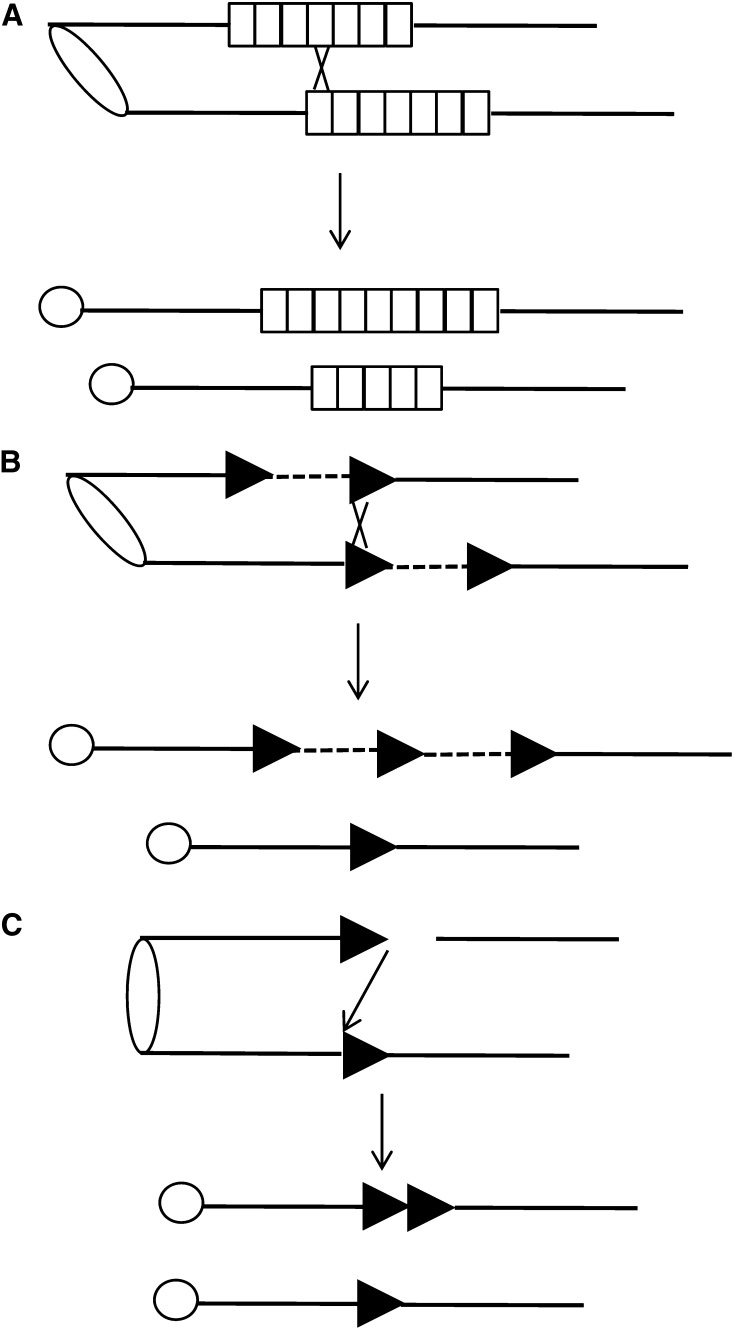
Mechanisms for altering or generating tandem gene arrays. Events are depicted as occurring between sister chromatids, and centromeres are shown as ovals or circles. (A) Unequal crossovers between misaligned sister chromatids can generate arrays with greater or fewer repeats per array. Repeats are indicated by small rectangles. (B) Unequal crossovers between flanking repeats (triangles) can produce a duplication of single-copy sequences (dashed line). (C) In this model, a broken end invades a sister chromatid using microhomology. Break-induced replication of the invaded chromosome results in a duplication of the sequence (shown as a triangle) initially present in one copy.

Homologous recombination between noncontiguous direct repeats can also result in duplication or deletion of single-copy sequences located between the repeats (Figure 1B). Such events have been detected in mammalian/human genomes (reviewed by Zhang *et al.* 2009) and in yeast (Koszul *et al.* 2004; Gresham *et al.* 2008; Zhang *et al.* 2013; Finn and Li 2013). Although most of these studies were performed in wild-type strains, Finn and Li (2013) showed that re-replication resulting from a prematurely initiated second round of DNA synthesis substantially elevated the frequency of this class of nonallelic homologous recombination.

Two other mechanisms that lead to duplications of single-copy genes also have been observed. The first mechanism involves a specialized type of break-induced replication (BIR). As shown in Figure 1C, a broken chromosome end invades a nonallelic region of a sister chromatid, copying sequences from the chromatid. This initial invasion involves little or no sequence homology. In yeast, which has small chromosomes (<2 Mb), synthesis could continue to the end of the chromosome. This type of event (termed “microhomology-mediated break-induced replication,” or MMBIR) was demonstrated to be the causal mechanism for some duplications of the yeast ribosomal protein gene *RPL20B* (Payen *et al.* 2008). A related mechanism that sometimes involves multiple invasions and template switches was proposed to account for some classes of CNVs in mammalian cells (Lee *et al.* 2007).

Because different selective procedures were used to obtain *de novo* duplications in the yeast experiments described previously, it is difficult to compare the relative rates of duplications by these pathways. In experiments in which duplications of a reporter gene were located between two retrotransposons located about 50 kb apart, duplications were observed at an approximate frequency of 10^−6^−10^−7^ (Zhang *et al.* 2013); all of the detected duplications in a haploid strain resulted from unequal crossovers between the flanking Ty1 elements (Zhang *et al.* 2013). This result argues that, at least in this chromosomal context, nonallelic homologous recombination events generate duplications more frequently than MMBIR or related mechanisms. In contrast, duplications of the *RPL20B* gene were generated by nonallelic homologous recombination and MMBIR with approximately equal frequencies (about 5 × 10^−8^; Payen *et al.* 2008).

In the studies described herein, we characterized the *CUP1* locus in a collection of yeast strains isolated from the wild and from clinical specimens. In the yeast strain S288c, which was the first strain sequenced (Saccharomyces Genome Database [SGD]), the *CUP1* locus is depicted as having two repeats (shown in red brackets in Figure 2A). The repeat has a complex structure that begins about 150 bp upstream of the *RUF5-1* gene, which encodes a transcript of unknown function (McCutcheon and Eddy 2003). The *CUP1* gene (encoding a protein of only 61 amino acids; Karin *et al.* 1984) is embedded within the *RUF5* gene, but is transcribed in the opposite orientation (http://www.yeastgenome.org/cgi-bin/seqTools). There is a second intergenic region of about 70 bp separating the 3′ end of the *RUF5-1* gene from the 3′ end of *YHR054C*. *YHR054C* is identical to the terminal 1 kb of *RSC30*. There is also an ARS element within each repeat that overlaps with the 3′ end of *RUF5*, and extends to the 3′ end of *RSC30*. At the centromere-distal end of the array is a complete copy of *RSC30*.

**Figure 2:**
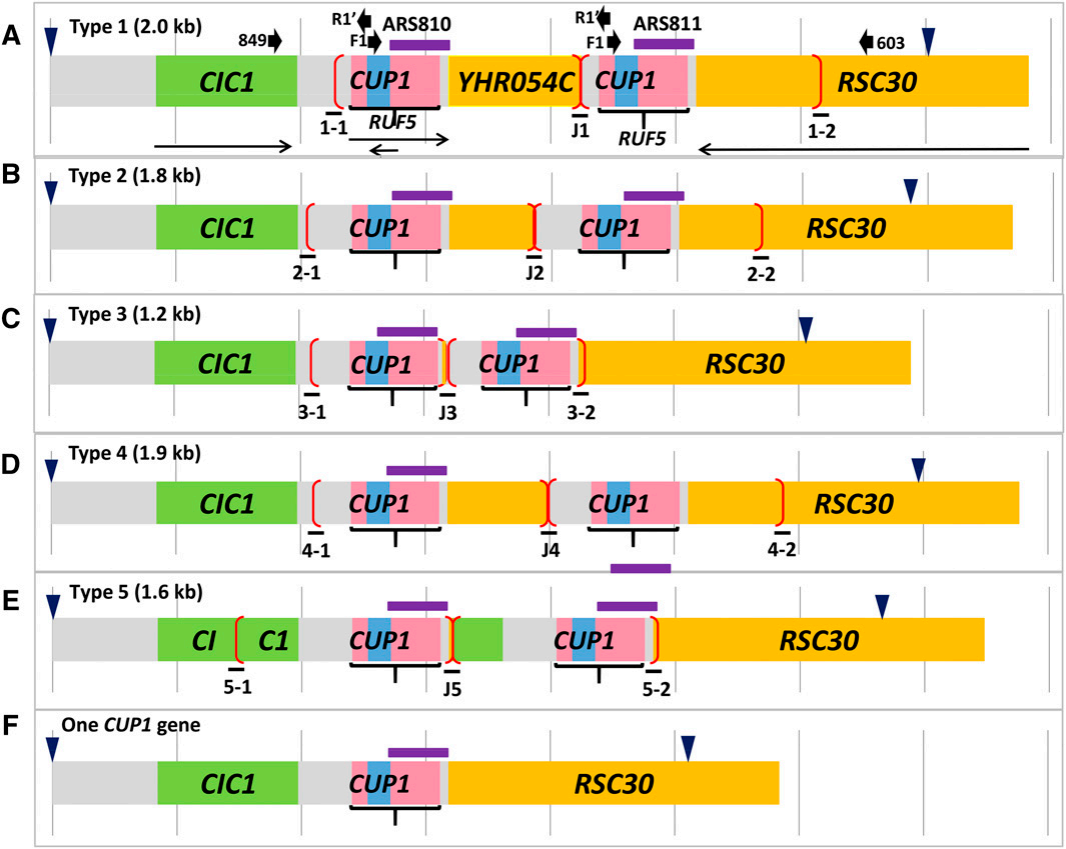
Structures of the *CUP1* loci for five types of *CUP1* repeats and a single-copy *CUP1* locus. The *CUP1* gene (blue) is located within a noncoding RNA gene (*RUF5*, pink) between the genes *CIC1* (green), and *RSC30* (orange) on chromosome VIII. We show only two copies of each repeat (bracketed in red), although most strains have more than two copies. Thin horizontal arrows show the direction of transcription, and short fat arrows indicate the location of primers used in the analysis. The vertical gray lines are 1 kb apart, and the blue arrows show the position of *EcoR*I sites relevant to Southern analysis. *ARS* elements are indicated by purple lines. The lines labeled J1-J5 represent the junction fragments between the end of one repeat and the beginning of the next. Lines 1−1 to 5−1 represent the leftmost end of the tandem arrays, and lines labeled 1−2 to 5−2 represent the rightmost end of the array. (A) Type 1 repeats (2.0 kb) found in strains S288c and W303-1A. *YHR054C* is an open reading frame derived from the 3′ end of *RSC30*. (B) Type 2 repeats (1.8 kb) found in strains YJM189 and YJM972. (C) Type 3 repeats (1.2 kb) found in strains YJM693, YJM789, and YJM1549. (D) Type 4 repeats (1.9 kb) found in strains YJM271, and YJM1307. (E) Type 5 repeats (1.6 kb) found in strains YJM456, YJM969, and YJM978. (F) Single-copy *CUP1* locus found in the copper-sensitive strain DTY3.

The repeat length in S288c is 1998 bp, which we will refer to as the Type 1 repeat. Although only two repeats are shown in the genomic sequence of S288c in the SGD, the closely related strain X2180-1A has about 15 repeats (Fogel and Welch 1982). Herein, we find that S288c also has about 15 repeats. A copper-sensitive strain BZ31-1-7Ba had a restriction enzyme digest pattern consistent with the presence of a single *CUP1* gene (Fogel and Welch 1982), and industrial yeast strains were observed that had tandem *CUP1* repeats of about 1.5 and 1.7 kb (Welch *et al.* 1983). Neither the single-copy *CUP1* repeat nor the variant repeats were sequenced in previous studies.

Whole-genome sequencing and assembly recently has been performed for 93 diverse strains of *Saccharomyces cerevisiae* (P. K. Strope, D. A. Skelly, S. G. Kozmin, G. Mahadevan, E. A. Stone, P. M. Magwene, F. S. Dietrich, and J. H. McCusker, personal communication); this collection, which also includes seven previously sequenced strains, has been termed the “100-genomes strains.” From these sequences and additional sequencing efforts, we defined the structures of five different classes of *CUP1* repeats as well as the sequence of a strain (DTY3) with only a single copy *of CUP1*. The comparison of these sequences suggests a simple mechanism by which a single *CUP1* gene is duplicated to form a tandem array by unequal non-homologous recombination.

We also compared the sizes of the *CUP1* arrays in 13 different strains that had at least two copies of *CUP1* and found a size range of between two and eighteen repeats per array. The size of the array roughly correlated with the ability of the strain to grow in high levels of copper. Finally, we showed that a strain with 14 *CUP1* repeats reverted to having only one *CUP1* gene at a rate of about 7.6 × 10^-7^/division.

## Materials and Methods

### Yeast strains

The genotypes of all strains used in our study are shown in Supporting Information, Table S1 and primers used in strain construction, polymerase chain reaction (PCR) analysis, or DNA sequencing are in Table S2. The genotypes of the haploid strains S288c (Engel *et al.* 2014), W303-1A (Thomas and Rothstein 1989), YJM789 (Wei *et al.* 2007), and DTY3 (Tamai *et al.* 1993) have been previously described. The diploid strains YJM189, YJM271, YJM456, YJM693, YJM969, YJM972, YJM978, YJM996, YJM1307, and YJM1549 were isolated from the wild (P. K. Strope, D. A. Skelly, S. G. Kozmin, G. Mahadevan, E. A. Stone, P. M. Magwene, F. S. Dietrich, and J. H. McCusker, personal communication); these diploids were generated by a mating type switch and have no polymorphisms other than at the *MAT* locus. The strain YJM799 is isogenic with YJM789 except for changes introduced by transformation and was obtained from J. McCusker (Duke University). JSC19-1 is also isogenic with YJM789 except for changes introduced by transformation (St. Charles and Petes 2013).

The strain JSC10-1 (St. Charles and Petes 2013) is isogenic with W303-1A except for changes introduced by transformation and has the genotype: *MAT***a** *leu2-3,112 his3-11,15 ura3-1 ade2-1 trp1-1 can1-100Δ*::*NAT RAD5*. We generated a PCR fragment containing a wild-type *URA3* gene by amplifying genomic DNA of the strain S288c with the primers VIII212898::URA3 F and VIII212898::URA3 R. This fragment was transformed into JSC10−1 to generate a derivative (YZ22) that had the *URA3* gene integrated within the *CUP1* cluster at position 212898 in the *RUF5* gene of one of the repeats; this allele is called *VIII212898*::*URA3*.

### Measurement of the sizes and sequences of *CUP1* repeats

Our first estimate of the sizes of the *CUP1* repeats in different strains was initially based on a PCR analysis. For this analysis, we used primers F1 and R1′ located within *CUP1* but oriented in different directions (Figure 2A). To generate a PCR product, the array needs to contain at least two tandem *CUP1* genes. The resulting PCR fragments were analyzed by gel electrophoresis. Some of the strains did not generate a PCR fragment with this procedure. We confirmed that these strains had only a single copy of *CUP1* by PCR analysis using the primers VIII211849 and VIII216603 (abbreviated 849 and 603 in Figure 2A). With these primers, strains that have a single copy of *CUP1* produce a PCR fragment of 2.7 kb.

Sequence analysis of various classes of repeats and their flanking sequences were determined by sequencing PCR fragments with the primers described in File S1. These sequences are displayed in Table S3, Table S4, Table S5, Table S6, Table S7, Table S8, Table S9, and Table S10.

### Analysis of the number of *CUP1* copies in tandem arrays

Genomic DNA isolated from different strains was isolated in plugs of low-melt agarose as described previously (McCulley and Petes 2010). The samples were treated overnight at 37° with *Eco*RI. The resulting DNA fragments were separated by CHEF (*i.e.*, contour-clamped homogeneous electric field) gel electrophoresis (McCulley and Petes 2010), followed by transfer of the separated fragments to nylon membranes. The hybridization probes were prepared using digoxygenin-dUTP (Roche); details of the hybridization conditions are in File S1. Sizes of the tandem arrays were estimated relative to DNA size standard (Bioline DNA Hyperladders I and VI).

### Measurements of copper resistance

For each strain, about 1000 cells were inoculated into 5 mL of SD complete medium (Guthrie and Fink 1991) containing levels of copper sulfate that varied between 0 and 2.4 mM, changing in 0.1 mM increments. After 2 d of growth at 30°, we measured the OD_660_ of each culture. If the OD_660_ of the undiluted culture was <0.1, we scored the concentration as inhibitory to growth of that strain. In the uninhibited cultures, the OD_660_ was between 1.8 and 3, the equivalent of about 2−4 × 10^7^ cells/mL.

### Measurements of the rate of loss of a *URA3* gene integrated within the *CUP1* repeats in YZ22

As described previously, the haploid strain YZ22 (isogenic with W303-1A) contains a *CUP1* array of approximately 14 repeats with a *URA3* gene integrated into the array. As explained in the text, an unequal crossover or various intrachromatid recombination events can result in loss of the insertion. Strains that lose the *URA3* insertion result in derivatives that are Ura^-^ and selectable on medium containing 5-fluoro-orotate (Boeke *et al*. 1987). We measured the frequency of 5-FOA^R^ derivatives and the total number of cells in about 60 independent cultures of YZ22. These frequencies were converted to rates using the method of the median (Lea and Coulson 1949). We screened 135 independent 5-FOA^R^ derivatives by PCR analysis with primers VII211849 F and VIII216603 to identify strains that contained only one *CUP1* gene. Six of the 135 strains had the 2.7 kb fragment expected for single *CUP1* genes.

## Results

### Identifying five different *CUP1* repeats

Until recently, the complete assembled sequence of the *CUP1* array was available only for S288c (Johnston *et al.* 1994). In S288c, the *CUP1* repeats are about 2.0 kb in size. In examining genomic sequences derived from YJM789 (Wei *et al.* 2007), we noticed that the partial sequence of the *CUP1* repeat in contig 18 was different from that of S288c. We decided, therefore, to examine the sequence of the *CUP1* repeats and flanking sequences in YJM789 and other yeast strains that had repeats different from S288c. In addition, we sequenced the *CUP1* gene in DTY3, a strain that has only one *CUP1* gene.

**Figure 3:**
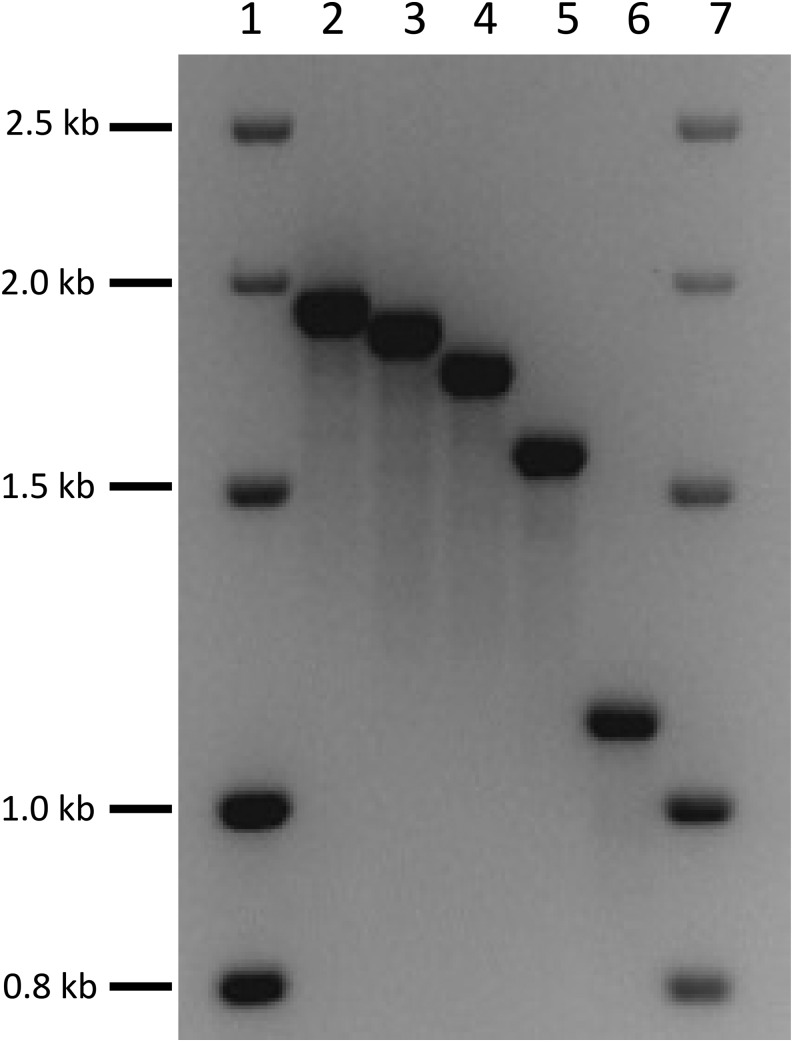
Analysis of PCR fragments representing various types of *CUP1* repeats. Using primers F1 and R1′, we PCR-amplified genomic DNA samples from five different yeast strains. Because of the locations of F1 and R1′ within the *CUP1* gene, the resulting fragments are about 40 bp shorter than the repeat length determined by DNA sequencing. Lanes 1 and 7 contain sizing ladders. The strain names, and the types and sizes of the repeats in the other lanes are: lane 2 (S288c, type 1, 2.0 kb); lane 3 (YJM271, type 4, 1.9 kb); lane 4 (YJM189, type 2, 1.8 kb); lane 5 (YJM456, type 5, 1.6 kb); lane 6 (YJM789, type 3, 1.2 kb).

The initial characterization of the repeats was done by PCR analysis using primers located within the *CUP1* gene (F1 and R1′, Table S2). The location and orientation of the primers are shown in Figure 2A. To produce a PCR product, there must be at least two tandem *CUP1* copies. Because these primers are located about 40 bp apart, the PCR product is about 40 bp smaller than the size of the repeats. In Figure 3, we show a PCR analysis of the five classes of repeats. Although we show the results for only a few of the strains from the 100-genomes sequencing project, all of these strains were examined by PCR.

These results and those obtained by deep sequencing (P. K. Strope, D. A. Skelly, S. G. Kozmin, G. Mahadevan, E. A. Stone, P. M. Magwene, F. S. Dietrich, and J. H. McCusker, personal communication) indicate that there are at least five types of *CUP1* repeats in *S. cerevisiae*. The approximate sizes of these repeats in kb are: Type 1 (2.0), Type 2 (1.8), Type 3 (1.2), Type 4 (1.9), and Type 5 (1.6). S288c and W303-1A have Type 1 repeats, whereas YJM789 has Type 3 repeats. From the deep-sequencing analysis, of the 66 strains with at least two *CUP1* genes, 57 had only one type of repeat per array, and 9 had more than one type. Of the “pure” arrays, the number of strains with each type are: Type 1 (8), Type 2 (14), Type 3 (18), Type 4 (4), and Type 5 (13).

About one third of the 100-genome strains examined by PCR failed to produce a product with primers F1 and R1′. Genomic DNA from these strains was re-examined using primers VIII211849 and VIII216603 (labeled as 849 and 603, respectively, in Figure 2A) that are located in the genes flanking the *CUP1* sequences. In strains that have only one copy of *CUP1*, we expect to see a PCR fragment of about 2.7 kb. A fragment of this size was observed in the 100-genomes strains that failed to generate a PCR product with primers F1 and R1′. We also observed the 2.7-kb fragment in DNA derived from the copper-sensitive strain DTY3. Deep-sequencing analysis also showed that 30 of the 100-genome strains had only one *CUP1* gene.

### Sequence analysis of *CUP1* repeats

**Figure 4:**
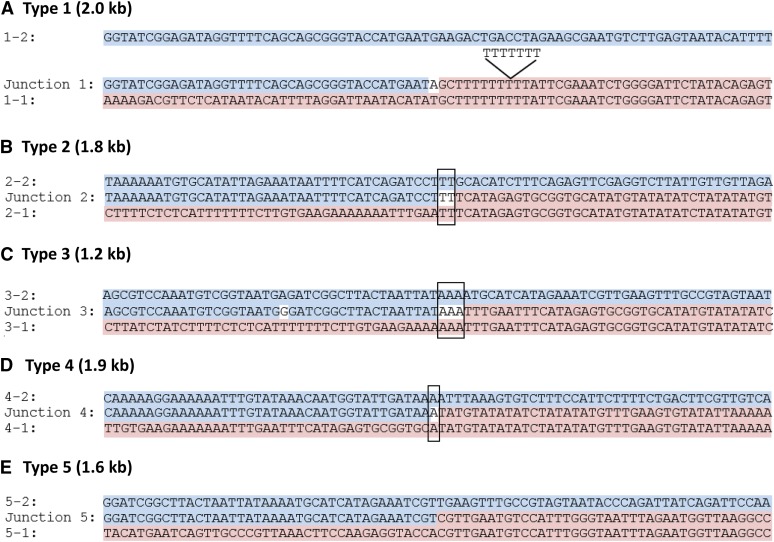
Sequences at the breakpoints for five different types of *CUP1* repeats. Breakpoint sequences of five different types of *CUP1* repeats are shown in comparison to the sequences flanking the repeats. The breakpoints of each repeat are labeled “Junctions 1−5,” and are the middle lines of each comparison. The top lines of each comparison (highlighted in blue) represent sequences from the centromere-distal flanking region, usually including a portion of the *RSC30* gene, and the bottom lines are from the centromere-proximal flanking region (highlighted in pink) usually containing a portion of the *CIC1-RUF5* intergenic region. For each comparison, the flanking sequences are from the same strain that contained the repeats. The locations of junctions J1-J5, as well as the locations of the flanking sequences, are shown in Figure 2. Sequence matches of the junction sequences to the centromere-distal and centromere-proximal flanking sequences are highlighted in blue and pink, respectively. Bases that are shared homologies of the flanking sequences are shown in boxes. Bases that do not match either flanking sequence or that match both flanking sequences are not highlighted. (A) J1 junction sequences of Type 1 repeats (S288c). As indicated, the T-tract is seven bp longer in the repeat than in the flanking sequences. There is an A/T base pair at the junction that is not derived from either of the flanking sequences. (B) J2 junction sequences (YJM189 and YJM996). (C) J3 junction sequences (YJM789). There is one SNP distinguishing the repeat sequence and that of the centromere-distal flanking sequence (base that is not highlighted within the highlighted region). (D) J4 junction sequences (YJM271, YJM1307). (E) J5 junction sequences (YJM456). The strain YJM769 had the identical sequence as YJM456 except for the presence of one SNP (Table S8 and Table S9).

The structure of the Type 1 repeats of S288c is shown in Figure 2A. The repeat can be defined by the sequence of the junction (labeled “J1” in Figure 2A) that has centromere-proximal sequences derived from *RSC30* (named *YHR054C*) fused to centromere-distal sequences derived from the region located 5′ to *RUF5*. As shown in Figure 4A, the junction sequence can be matched nearly perfectly to sequences derived from within the *RSC30* gene (line above J1, labeled 1−2) on the left side and to sequences from the *CIC1-RUF5* intergenic region (line below J1, labeled 1−1) on the right side; the locations of the 1−1 and 1−2 sequences are shown in Figure 2A. There are no sequence homologies at the breakpoints of the junctions. As will be explained in detail below, this observation is relevant to the mechanism by which the *CUP1* repeats were formed.

The J1 junction has only two sequence differences that are not predicted from a simple fusion of *RSC30* and the intergenic region of *CIC1-RUF5*: an A at the fusion breakpoint and seven T residues inserted into the poly T tract in the region upstream of *RUF5*. The length of the Type 1 repeat is 1998 bp (Johnston *et al.* 1994). It should be mentioned that it can be confusing to use SGD coordinates to describe the repeats, since the sequences within the repeats match to two sets of coordinates, and the coordinates of the flanking sequences are displaced by about two kb (the length of the Type 1 repeat).

We sequenced the *CUP1* repeats in eight strains including one Type 2 (YJM189), two Type 3 (YJM789, YJM969), two Type 4 (YJM271, YJM307), two Type 5 (YJM456, YJM996) strains, and one strain (DTY3) with a single copy of *CUP1*. The strain S288c, which has a Type 1 repeat, was used for sequence comparisons by BLAST analysis (http://www.yeastgenome.org/cgi-bin/seqTools). For those strains that were also examined by deep sequencing, the sequences were identical to those obtained from the PCR products (P. K. Strope, D. A. Skelly, S. G. Kozmin, G. Mahadevan, E. A. Stone, P. M. Magwene, F. S. Dietrich, and J. H. McCusker, personal communication). The details of the sequence analysis, and the resulting sequences are in File S1 and Table S3, Table S4, Table S5, Table S6, Table S7, Table S8, Table S9, and Table S10. In Table S3, Table S4, Table S5, Table S6, Table S7, Table S8, Table S9, and Table S10, we included about 600 bp of sequences flanking the repeats, in addition to the sequences of the repeats themselves.

Based on the sequence analysis, the repeats of each type are depicted in Figure 2. In Figure 4, B−E, we show the junction sequences (middle line) compared with sequences flanking the repeat near *RSC30* (top line) and *CIC1* (bottom line). As observed for the Type 1 repeat, the Type 2−5 junctions show little (≤ 3 bp) sequence homology at the breakpoints. This observation argues strongly that the mechanism that generated the repeats likely involves nonhomologous end-joining (details in Discussion).

We also compared the sequences of all eight strains described above to the sequence of S288c. There were very few differences, and these differences are summarized in Table S3, Table S4, Table S5, Table S6, Table S7, Table S8, Table S9, and Table S10. Two of the strains (YJM789 and DTY3) had a C to T change in the *CUP1* coding sequence (SGD coordinate 212556); this change does not alter an amino acid. In strains YJM789, YJM456, YJM969, and DTY3, the termination codon of *CUP1* was UAA instead of UGA, the termination codon for *CUP1* in S288c and the other strains.

### Variation in the number of *CUP1* repeats in different yeast strains

**Figure 5:**
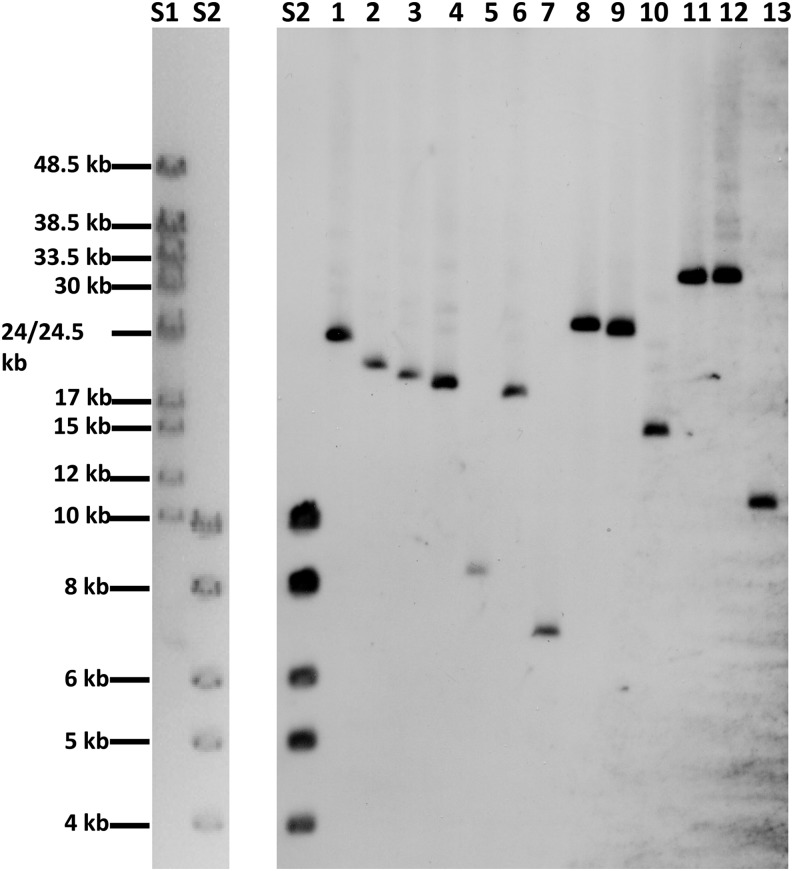
Southern analysis of the *CUP1* genes in 13 yeast strains. Genomic DNA from each strain was treated with *Eco*RI. There are no recognition sites for *Eco*RI within the *CUP1* repeats (Figure 2A). The fragments were separated by gel electrophoresis, transferred to a membrane, and hybridized to a probe containing the *CUP1* sequences. The lanes labeled S1 and S2 on the left side of the figure are ethidium bromide-stained fragments representing size standards (Hyperladders VI and I from Bioline). S2 in the gel on the right side of the figure is the same ladder hybridized to a ladder-specific probe. The samples in lanes 1−13 are: 1 (YJM189), 2 (YJM271), 3 (YJM456), 4 (YJM693), 5 (YJM969), 6 (YJM972), 7 (YJM978), 8 (YJM996), 9 (YJM1549), 10 (YJM1307), 11 (S288c), 12 (W303-1A), and 13 (YJM789). The fragment sizes in lanes 1−13 are in Table 1.

Variation in the number of *CUP1* repeats in tandem arrays of different yeast strains has been observed previously by Southern analysis (Welch *et al.* 1983). In addition, in the analysis of the 100-genome strains, estimates of the numbers of repeats per strain were made based on the number of times *CUP1* sequences were present relative to single-copy sequences (P. K. Strope, D. A. Skelly, S. G. Kozmin, G. Mahadevan, E. A. Stone, P. M. Magwene, F. S. Dietrich, and J. H. McCusker, personal communication). We used Southern analysis to determine the number of repeats in 10 strains that were part of the 100-genome strain analysis, as well as three other yeast strains (S288c, W303-1A, and YJM789). For this analysis, genomic DNA was treated with *Eco*RI which does not have a recognition sequence within the repeats (Figure 2A). Southern analysis of the strains is depicted in Figure 5.

**Figure 6:**
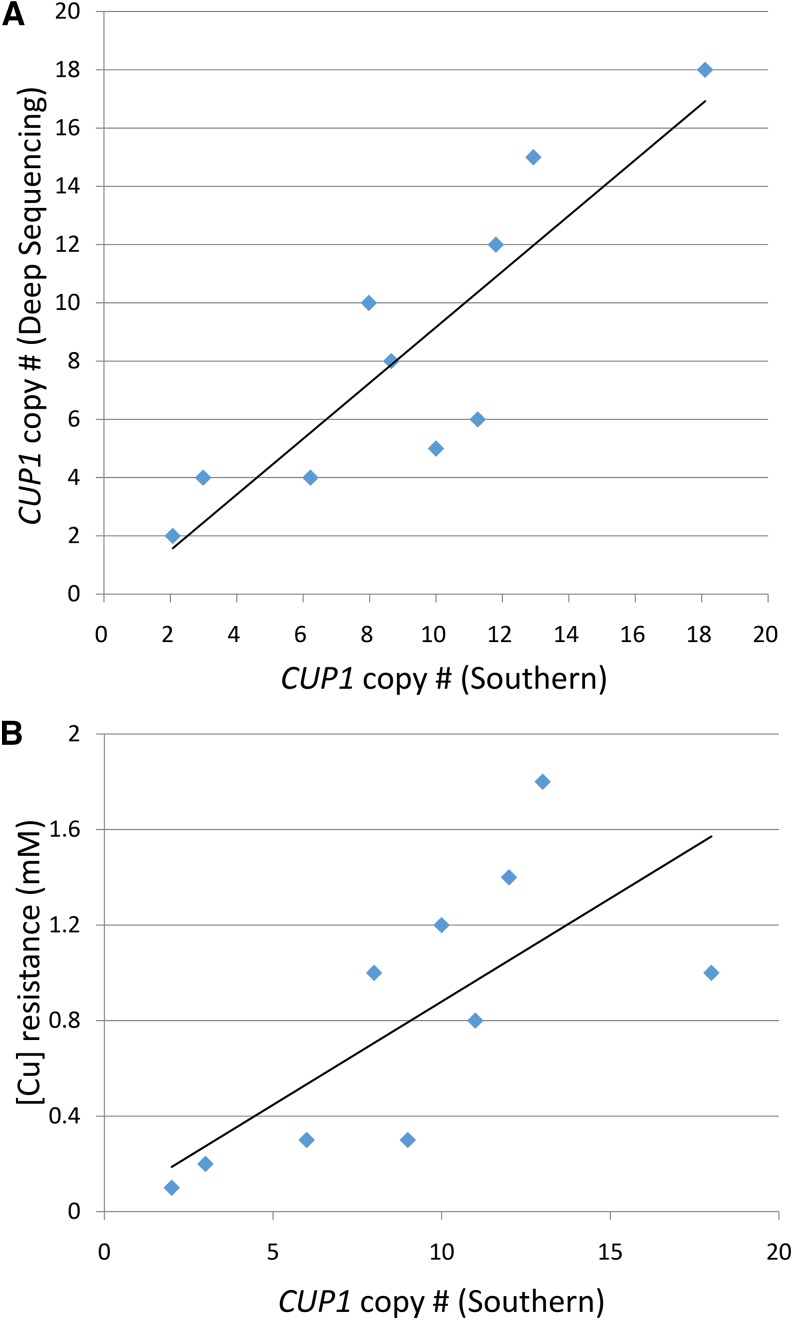
Correlations between different assays of *CUP1* copy-number and between *CUP1* copy-number and copper resistance. (A) Correlation between different assays of *CUP1* copy-number. From the information in Table 1, we found a strong correlation (R^2^ = 0.76) between copy-number as estimated by coverage in deep-sequencing experiments (Y-axis) *vs.* copy-number estimated by Southern analysis (X-axis). (B) Correlation between *CUP1* copy-number (Southern analysis) and the minimal concentration of copper that inhibits growth for diploid strains.

Based on the size of observed fragment, the sizes of the repeats, and the location of the flanking *Eco*RI sites, we calculated the number of repeats in each strain (Table 1). This number was based on three independent measurements. In the strains examined, the number of repeats varied between two copies (YJM978) and 18 copies (YJM1549). There is a strong correlation (R^2^ = 0.76, *P* = 0.001) between the copy numbers as determined by Southern analysis and the copy numbers as estimated by coverage in the deep-sequencing experiments (Table 1 and Figure 6A). Although the agreement in the estimates of the number of *CUP1* copies is reasonably good, for several of the strains, there were significant differences. These differences could reflect limitations of the methods used to estimate copy number or unselected alterations in the number of *CUP1* genes in independent isolates of the same strain.

**Table 1:** Analysis of *CUP1* gene tandem arrays in 14 yeast strains of *Saccharomyces cerevisiae*

Strain	Type of Repeat (size in kb)	Size of *Eco*RI fragment, kb*^a^*	*CUP1* Copy No. Southern	*CUP1* Copy No. (Deep Sequencing)*^b^*	[Cu^2+^] Inhibitory Concentration, mM
S288c	1 (2.0)	30.8	14	ND	2
W303-1A	1 (2.0)	31.3	14	ND	1.8
YJM189	2 (1.8)	23.3	11	6	0.8
YJM972	2 (1.8)	18.7	8	10	1
YJM996	2 (1.8)	25.4	12	12	1.4
YJM789	3 (1.2)	11.6	7	ND	0.4
YJM693	3 (1.2)	19.3	13	15	1.8
YJM1549	3 (1.2)	24.4	18	18	1.0
YJM271	4 (1.9)	20.7	9	8	0.3
YJM1307	4 (1.9)	15.5	6	4	0.3
YJM456	5 (1.6)	19.8	10	5	1.2
YJM969*^c^*	5 (1.6)	8.5	3	4	0.2
YJM978	5 (1.6)	6.5	2	2	0.1
DTY3	No repeat	5.2*^d^*	1	NR	<0.1

ND, not determined. NR, not relevant.

a Average size of *CUP1*-containing *EcoR*I fragment based on two to three independent experiments.

b Estimate of *CUP1* copy number based on coverage in deep-sequencing analysis (P. K. Strope, D. A. Skelly, S. G. Kozmin, G. Mahadevan, E. A. Stone, P. M. Magwene, F. S. Dietrich, and J. H. McCusker, personal communication).

c This strain had a mixture of Type 3 and Type 5 repeats by deep sequencing (P. K. Strope, D. A. Skelly, S. G. Kozmin, G. Mahadevan, E. A. Stone, P. M. Magwene, F. S. Dietrich, and J. H. McCusker, personal communication), but only Type 5 repeats by polymerase chain reaction analysis.

d This size is based on our sequencing results and on Southern analysis of a single-copy *CUP1* strain (Fogel *et al.* 1983).

### Copper resistance of strains with different types and different numbers of *CUP1* repeats

To determine whether there is a significant positive correlation between the number of *CUP1* genes in the tandem array and the copper resistance of different strains, we measured the minimal concentration of copper that prevented growth (details in the section *Materials and Methods*). The analysis of copper resistance was done in the same strains that were used for the Southern analysis (Table 1). We determined the correlation between copper resistance and the number of repeats for the ten diploid strains analyzed (Figure 6B). The correlation is significant (*P* < 0.02) with R^2^ = 0.52. Qualitatively similar results were observed previously (Fogel *et al.* 1983; Welch *et al.* 1983; P. K. Strope, D. A. Skelly, S. G. Kozmin, G. Mahadevan, E. A. Stone, P. M. Magwene, F. S. Dietrich, and J. H. McCusker, personal communication). When we included the haploid strains (S288c, W303-1A, YJM789, and DTY3) with the diploid strains, the correlation between copper resistance and copy number was higher (R^2^ = 0.64; *P* < 0.001).

### Rate of reduction of a tandem *CUP1* array to a single copy of *CUP1*

**Figure 7:**
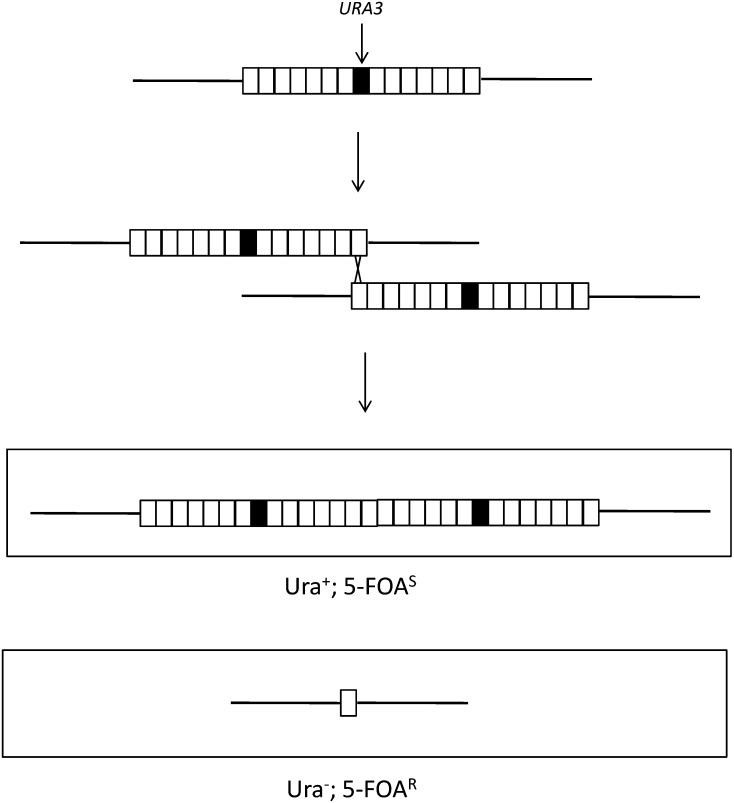
Loss of a *CUP1* tandem array by unequal crossing-over. We depict the *CUP1* array of the haploid strain YZ22 that contains an inserted *URA3* gene within the array. An unequal crossover between the terminal *CUP1* repeat of one array with opposite terminus of another array located on a sister chromatid results in one cell with an elongated array with two *URA3* insertions and another cell with a single *CUP1* repeat and no *URA3* insertion.

Although we are primarily concerned with the mechanisms by which single-copy genes become duplicated, a related issue is the mechanism by which a tandem array is reduced to a single-copy gene. We constructed a haploid YZ22 in which a tandem array of approximately 14 *CUP1* repeats has an integrated *URA3* gene. Unequal crossing (Figure 7) or several types of homologous recombination events not depicted (intrachromatid “pop-out” recombination, and single-strand annealing) can result in a single-copy *CUP1* locus. Derivatives of YZ22 that lose the *URA3* insertion can be identified by selection on solid medium containing 5-fluoro-orotate (details in Materials and Methods). From measurements of the frequency of 5-FOA^R^ derivatives in multiple independent cultures, we calculated the rate of appearance of 5-FOA^R^ derivatives to be 1.7 × 10^−5^/division.

To determine what fraction of these derivatives had reduced the array to a single copy, we examined 135 independent 5-FOA^R^ derivatives by PCR using primers from within the flanking *CIC1* and *RSC30* genes (primers VIII211849 F and VIII216603 R, respectively). In strains with a single copy of *CUP1*, a PCR fragment of 2.7 kb was observed. 6 of the 135 strains had a single copy of *CUP1*. Thus, the approximate rate for reduction of the tandem array of CUP1 genes to a single copy is 6/135 × 1.7 × 10^−5^ or 7.6 × 10^−7^/division.

## Discussion

Tandemly repeated genes are a common feature of eukaryotic genomes. These genes often encode proteins or RNA species (such as ribosomal RNA) that are required in large amounts. As discussed in the *Introduction*, the homologous recombination mechanisms that alter the number of repeats in a pre-existing tandem array are well characterized, and include unequal crossing-over, intra-chromatid crossovers, gene conversion events, and single-strand annealing. The events that generate a tandem duplication of a single-copy gene are less understood. From our analysis, we argue that this duplication event at the *CUP1* locus likely reflects the joining of two broken ends by a non-homologous end-joining event.

### Previous observations of gene duplications in yeast

In yeast, the number of repeats in the ribosomal RNA gene cluster alters at a high rate, 10^−1^/meiotic division (Petes 1980) and 10^−2^/mitotic division (Szostak and Wu 1980). In general, the rates of generating *de novo* duplications are much less, although the rates are variable depending on the context of the reporter gene and the details of the experimental system. If the reporter gene is flanked by repeated elements, the duplication often occurs by unequal crossovers between these elements. For example, Zhang *et al.* (2013) showed that duplication of a reporter construct located between two Ty elements on chromosome V in a haploid strain was a consequence of unequal crossovers, occurring at a frequency of about 10^−6^−10^−7^. No events in which the duplication was generated by non-homologous end-joining were observed. In strains in which re-replication of an origin was induced, gene duplications of sequences within the re-replicated region were efficiently generated (Green *et al.* 2010; Finn and Li 2013). These events usually involved nonallelic homologous recombination between Ty elements flanking the duplicated region.

Haploid yeast strains with a deletion of the ribosomal protein gene *RPL20A* grow slowly, allowing for selection of fast-growing derivatives that duplicate the *RPL20A*-related gene *RPL20B* (Koszul *et al.* 2004). These duplications involved both flanking repeated genes (delta and Ty elements) and microhomologies. The events involving recombination between large (>300 bp) regions of homology were Rad52p-dependent, and all classes of duplications had a requirement for Pol32p (Payen *et al.* 2008). Based on the genetic analysis, Payen *et al.* (2008) argued that many of the Rad52p-independent duplications were generated by microhomology-mediated BIR. The rates of duplications of *RPL20B* were estimated at between 10^−7^−10^−10^, depending on what correction factor was used to determine the relative growth rates of strains with and without the duplication (Koszul *et al.* 2004; Payen *et al.* 2008). The *de novo RPL20B* repeats were large, varying between 41 and 655 kb (Koszul *et al.* 2004). In experiments selecting for duplications of the *ADH4* gene, only one large (>100 kb) chromosomal duplication was observed, yielding a rate of about 10^−10^/cell division (Dorsey *et al.* 1992).

Although it is difficult to reach a definitive conclusion based on these data, most of the yeast observations suggest that duplications that arise as a consequence of homologous recombination between flanking repeated sequences occur more frequently than those generated by non-homologous end-joining events. In contrast, many duplications in mammalian cells are produced by nonhomologous end-joining or related mechanisms (Zhang *et al.* 2009). These differences are consistent with the relative importance of homologous recombination *vs.* nonhomologous end-joining in yeast compared with mammals (Jasin and Rothstein 2013).

### Duplications of *CUP1* in yeast

The *CUP1* repeats are located on the right arm of chromosome VIII, and there are no flanking Ty elements on this arm in S288c. In strains with tandemly repeated *CUP1* genes, Fogel *et al.* (1983) were readily able to select derivatives with longer arrays by growing the strains in high concentrations of copper. They were not, however, able to isolate strains with a *de novo* duplication of *CUP1* from a strain that had a single *CUP1* copy. Their results argue that such duplications are infrequent when *CUP1* is in its “normal” location. Our analysis of different types of *CUP1* repeats, however, suggest that such duplications occur during evolution.

**Figure 8:**
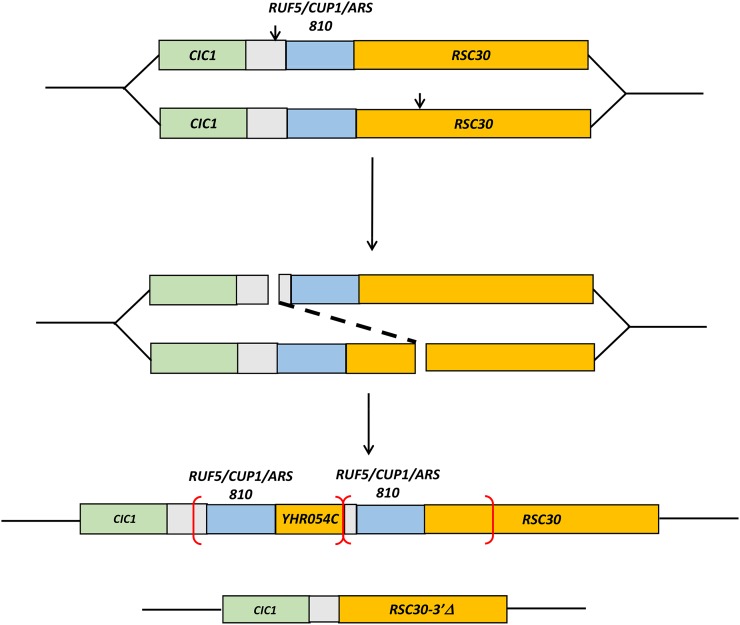
Generation of a *de novo* duplication of *CUP1* by unequal nonhomologous end-joining. A mechanism for the generation of a two-copy tandem array of *CUP1* from a single *CUP1* gene (similar to the duplication observed in S288c) is shown. We suggest that, during DNA replication, one fork is broken in the intergenic region between *CIC1* and *RUF5/CUP1/ARS810*. The second fork is broken within *RSC30*. The nonhomologous end-joining of one broken end to the other (indicated by the dashed line) would produce the duplication. If the other broken ends are also joined, a deletion of *RUF5/CUP1/ARS810* would be produced in the sister chromatid.

All five classes of repeats can be explained by the duplication mechanism shown in Figure 8. We suggest that, during replication of a chromosome with only one copy of *CUP1*, two breaks occur, one centromere-distal and one centromere-proximal to the *CUP1* locus. The joining of these broken ends by nonhomologous end-joining results in two products, one with a *CUP1* deletion and one with a tandem duplication. An alternative possibility is that the repeats were generated by a single break in the centromere-distal location, followed by a BIR event in which the centromere-proximal site was invaded. Because most BIR events involve either extensive sequence homology (Paques and Haber 1999) or 5−20 bp of microhomology (Payen *et al.* 2008), and most of the observed breakpoints in our study have very little or no homology, we favor the first alternative.

In a previous study, Haber and Louis (1998) noted that minisatellites in yeast were often flanked by 5−10 bp direct repeats, and suggested that these short repeats could be substrates for replication slippage or unequal crossovers. Although the repeated sequences at the *CUP1* junctions are three bp or less, it is possible that the initial duplication involved longer direct repeats but sequence alterations accumulated subsequent to the duplication. One argument against this possibility is that the sequences flanking the *CUP1* repeats are highly conserved. From Table S3, Table S4, Table S5, Table S6, Table S7, Table S8, and Table S9, we calculate that the average sequence divergence of the centromere-proximal 600 bp flanking the repeats (relative to the standard SGD sequence) is 0.7%. The centromere-distal sequences are similarly well conserved (0.4% divergence). Finally, the *CUP1* repeats show only 0.5% sequence divergence.

Although we favor the model in which the *CUP1* repeats are generated by “classic” NHEJ, this model does not explain the A/T base pair that is present at the junction of the Type 1 repeats. In an analysis of NHEJ in Drosophila, Yu and McVey (2010) observed joining events in which one or more bases were inserted at the junction. Their observations suggested that these insertions were a consequence of pairing interactions between short repeats, followed by limited DNA synthesis prior to the joining event. Although we do not need to invoke this model for most of the repeats, it is possible that the Type 1 repeats were generated by this mechanism (termed “synthesis-dependent microhomology-mediated end joining”). Alternatively, the A/T base pair may represent a mutation produced subsequent to the original NHEJ-generated duplication.

**Figure 9:**
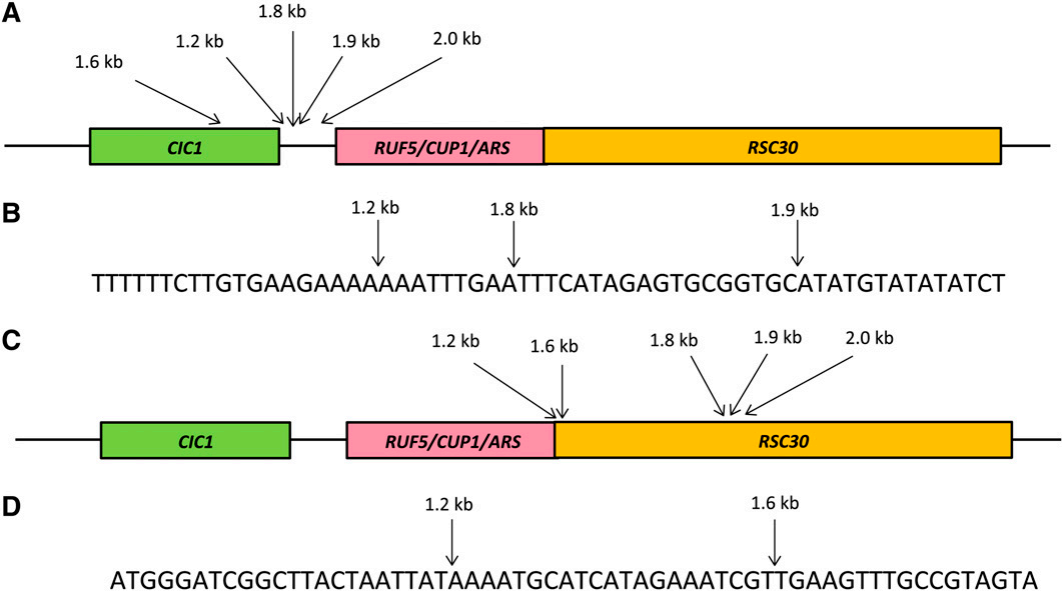
Clustered breakpoints of five different types of *CUP1* repeats. (A) Location of junction breakpoints in the *CIC1* gene and intergenic region for five types of *CUP1* repeats. (B) Sequence in the region of three tightly clustered breakpoints in the *CIC1-RUF5/CUP1/ARS* intergenic region. (C) Location of junction breakpoints in the *RSC30* gene for five types of repeats. (D) Sequence in the region of two tightly clustered breakpoints within *RSC30*.

The differences in the location of the breaks generated repeats that vary in size between about 1.2 kb and 2 kb. The centromere-proximal breaks occurred in two locations (Figure 9). Most were in the intergenic region between *CIC1* and *RUF5*, but one was within the *CIC1* gene. The centromere-distal breaks occurred either between *RUF5* and *RSC30*, or within the *RSC30* gene.

The locations of the presumptive DSBs required to generate the *CUP1* duplications could reflect chromosomal sequences that are intrinsically susceptible to breakage (fragile sites). In yeast, DNA sequences with the ability to form secondary structures (Voineagu *et al.* 2008) or to perturb the progression of replication forks (Cha and Kleckner 2002; Paeschke *et al.* 2011; Song *et al.* 2014) are hotspots for chromosome breakage.

The minimal size of the duplication is constrained by the sequences required for optimal *CUP1* expression. Aside from the *CUP1* coding sequence of about 200 bp, optimal expression of the gene and copper-induced transcription requires about 300 bp of upstream sequences (Thiele and Hamer 1986). Since the *CUP1* transcript is about 500 bp in size (Karin *et al.* 1984), the minimal size of a functional *CUP1* repeat is expected to be less than 600 bp. However, all of the repeats share sequences from the 3′ end of *RUF5* to the beginning of the *RSC30* gene, a region of about 920 bp. It is possible that these additional sequences are involved in regulating *CUP1* gene expression in some environments or enhancing the gene amplification process subsequent to the duplication. It is interesting that none of the duplications include an intact *CIC1* or *RSC30* gene. Koszul *et al.* (2004) found that duplications of the *RPL20B* gene often included many flanking genes. One obvious possibility is that yeast cells are intolerant of extra doses of either *CIC1* (encoding an essential protein associated with the proteasome; Jäger *et al.* 2001) or *RSC30* (encoding a protein involved in chromatin re-modeling; Angus-Hill *et al.* 2001).

### Alterations in the number of repeats within the *CUP1* tandem array

Several lines of evidence demonstrate that the number of repeats per tandem array alters at high frequency. First, as described previously, Fogel *et al.* (1983) showed that strains with longer arrays of *CUP1* could be readily isolated by growing the cells in high levels of copper. Second, isolates of yeast obtained from the wild have different numbers of *CUP1* genes in their arrays (Welch *et al.* 1983). In the strains examined in our study, the number of *CUP1* genes varied from one to eighteen (Table 1). Third, in several different mutant backgrounds, the rate of alterations in the number of repeats is very high, greater than 10^−3^/division (McCulley and Petes 2010; Song *et al.* 2014). Because loss or duplication of *CUP1* repeats occur in integral numbers of units, these events are presumably the result of homologous recombination (unequal crossovers, single-strand annealing, or gene conversion).

About 30% of the 100-genome strains had only one copy of *CUP1*. Such strains could represent the progenitor isolates from which the tandem arrays were derived. Alternatively, these isolates could represent strains in which all copies except one were lost as a consequence of homologous recombination. It is important to stress that the process by which a single *CUP1* repeat becomes duplicated is inherently different than the process by which a tandem array becomes deleted to a single copy. If the *CUP1* gene is not associated with repeated flanking elements, the duplication process likely occurs by some type of nonhomologous end-joining or a BIR event involving microhomologies. The latter type of event can occur by homologous recombination between the terminal repeats of the array.

Based on our study and a number of previous studies, we argue that *CUP1* tandem arrays arise as a consequence of two mechanisms: 1) a duplication of a single-copy *CUP1* gene by an infrequent nonhomologous recombination event and 2) increases in copy number from the duplication to multiple copies by a frequent homologous recombination event. In environments with high levels of copper, strains with larger numbers of *CUP1* repeats will be selected. Our observation that a tandem array of *CUP1* repeats can be reduced to a single *CUP1* copy emphasizes the necessity of a mechanism for generating *de novo* duplications.

## Supplementary Material

Supporting Information
