# Supplementary material for: Structures of Naturally Evolved CUP1 Tandem Arrays in Yeast Indicate That These Arrays Are Generated by Unequal Nonhomologous Recombination
Source: G3 (Bethesda). 2014 Sep 17;4(11):2259–69. doi: 10.1534/g3.114.012922 (PMC4232551; doi:10.1534/g3.114.012922)
Supplement: Supporting Information [file supp_4_11_2259__index.html]

Structures of Naturally Evolved CUP1 Tandem Arrays in Yeast Indicate That These Arrays Are Generated by Unequal Nonhomologous Recombination — Supporting Information 

# Structures of Naturally Evolved *CUP1* Tandem Arrays in Yeast Indicate That These Arrays Are Generated by Unequal Nonhomologous Recombination

## Supporting Information for Zhao *et al.*, 2014

**Files in this Data Supplement:**

- Supporting Information - File S1 and Tables S1-S10 (PDF, 304 KB)
- File S1 - Supplementary Materials and Methods (PDF, 105 KB)
- Table S1 - Strain genotypes. (PDF, 109 KB)
- Table S2 - Primer names and sequences used in strain constructions and analysis. (PDF, 75 KB)
- Table S3 - Sequence analysis of the *CUP1* repeats (Type 2, 1.8 kb) of YJM189. (PDF, 152 KB)
- Table S4 - Sequence analysis of the *CUP1* repeats (Type 2, 1.8 kb) of YJM996. (PDF, 151 KB)
- Table S5 - Sequence analysis of the *CUP1* repeats (Type 3, 1.2 kb) of YJM789. (PDF, 150 KB)
- Table S6 - Sequence analysis of the *CUP1* repeats (Type 4, 1.9 kb) of YJM271. (PDF, 152 KB)
- Table S7 - Sequence analysis of the *CUP1* repeats (Type 4, 1.9 kb) of YJM1307. (PDF, 152 KB)
- Table S8 - Sequence analysis of the *CUP1* repeats (Type 5, 1.6 kb) of YJM456. (PDF, 151 KB)
- Table S9 - Sequence analysis of the *CUP1* repeats (Type 5, 1.6 kb) of YJM969. (PDF, 151 KB)
- Table S10 - Sequence analysis of strain (DTY3) that has a single copy of *CUP1*. (PDF, 158 KB)
