# Supplementary material for: Structures of Naturally Evolved CUP1 Tandem Arrays in Yeast Indicate That These Arrays Are Generated by Unequal Nonhomologous Recombination
Source: G3 (Bethesda). 2014 Sep 17;4(11):2259–69. doi: 10.1534/g3.114.012922 (PMC4232551; doi:10.1534/g3.114.012922)
Supplement: Supporting Information [file supp_g3.114.012922_012922SI.pdf]

G3· Genes | Genomes | Genetics

Structures of naturally-evolved *CUP1* tandem arrays in yeast indicate that these arrays are generated by unequal non-homologous recombination

Ying Zhao[1], Pooja K. Strope[1], Stanislav G. Kozmin[1], John H. McCusker[1], Fred S. Dietrich[1], Robert J. Kokoska[2], and Thomas D. Petes[1]

[1]Department of Molecular Genetics and Microbiology and University Program in Genetics and Genomics, Duke University Medical Center, Durham, NC 27710

[2]Physical Sciences Directorate, U. S. Army Research Office, PO Box 12211, Research Triangle Park, NC 27709

DOI: 10.1534/g3.114.012922

File S1

Supplementary Materials and Methods

Southern analysis

The size of the *CUP1* tandem arrays was estimated by gel electrophoresis of *EcoR*I fragments derived from the various yeast strains. Following electrophoresis, the samples were transferred to Nylon membranes (Roche, Product # 11209272001). The 1 kb hybridization probe included sequences between coordinates 212534 and 213538, containing *CUP1* and flanking sequences within the repeat. The probe was synthesized to contain digoxygenin (DIG)-dUTP. Using the primers CUP1 amp5-2 and CUP1 amp3 (Table S2), we amplified genomic DNA of strain S288c using the PCR DIG probe synthesis kit of Roche (Product No. 11536090910). We used a concentration of DIG-dUTP of 21 μM in addition to the genomic DNA (10 ng), primers, and other constituents of the reaction. DIG-labeled probes for the DNA ladders (Bioline DNA Hyperladders I and VI (discontinued) were generated with DIG-High Prime DNA Labeling and Detection Starter Kit II (Roche, Product No. 11585614910) using the random-priming labeling technique.

Hybridization was performed according to the procedures described in the Roche DIG Application Manual for Filter Hybridization (http://lifescience.roche.com/wcsstore/RASCatalogAssetStore/Articles/05353149001_08.08.pdf). We used 45 μl of the DIG-labeled *CUP1* probe mixture in 25 ml of hybridization buffer (Roche DIG Easy Hyb Granules, Product No. 11796895001). Hybridization was conducted at 42^0 C. overnight, followed by two high-stringency washes done for 15 minutes at 65^0C. Chemiluminescent detection of the probes on the membranes was

done using Anti-Digoxigenin-AP, Fab fragments (Roche, Product No. 11093274910), and the DIG Wash and Block Buffer Set (Roche, Product No. 11585762001). The washing buffer, maleic acid buffer, and detection buffer were prepared as described in the DIG DNA Labeling and Detection Kit Version 19 protocol (https://cssportal.roche.com/LFR_PublicDocs/ras/11093657910_en_19.pdf).

DNA sequencing

For all strains containing repeated *CUP1* genes, we determined the sequence of the repeats, as well as the sequences that connected the repeats to single-copy sequences on the centromere-distal and centromere-proximal sides of the tandem array. The information about the flanking sequences was necessary to determine whether the tandem arrays were in the same chromosomal context on chromosome VIII in all strains. The results of this analysis are presented in Tables S3-S10. Sequences of all primers are in Table S2.

For the analysis of repeats, we first determined the sequences of the PCR fragments generated using the primers F1 and R1'. Since the fragment generated using these primers does not contains about 30 bp that separate the primer binding sites, we also determined the sequences of PCR fragments generated using the primers VIII212300 and VIII213031 that contains the sequences separating F1 and R1'. The specific primers used to sequence the repeats differed for different types of repeats, and are shown in boldface in Tables S3-S9.

The primers used to produce PCR fragments containing the centromere-proximal junctions (*CIC1-CUP1* region) were produced with the following primer pairs: 1) Type 2

(VIII211528 F and R1’), 2) Type 3 (VIII211528 F and R1’), 3) Type 4 (VIII211528 F and R1’), and 4) Type 5 (VIII211528 F and VIII212063 R). The primers used for sequencing are indicated in Tables S3-S9.

The primers used to produce PCR fragments containing the centromere-distal junctions (*CUP1-RSC30* region) were produced with the following primer pairs: 1) Type 2 (VIII213200 F and VIII216603), 2) Type 3 (F1 and VIII213537 R), 3) Type 4 (VIII213601 F and VIII216603 R), and 4) Type 5 (F1 and VIII214195). The primers used for sequencing are indicated in Tables S3-S9.

We also sequenced the *CUP1* region in a copper-sensitive strain DTY3 that contains a single copy of *CUP1*. Three overlapping PCR fragments were sequenced. One fragment (generated using primers VIII210632 F and VIII212063 R) contained the *CIC1* coding sequence and a portion of the *CIC1-CUP1* intergenic sequence. The second fragment (generated using primers VIII211849 F and VIII216603 R) extended from the 3’ region of *CIC1* to the 3’ region of *RSC30*. The third fragment (generated with primers VIII216314 F and VIII218008) contained the 5’ region of *RSC30*. The composite sequence and the primers used to produce the sequence are in Table S10.

Supplementary literature cited

Engel, S. R., F. S. Dietrich, D. G. Fisk, G. Binkley, R. Balakrishnan *et al.*, 2014 The reference genome sequence of *Saccharomyces cerevisiae*: then and now. G3 (Bethesda) 4:389-398.

St. Charles, J., and T. D. Petes, 2013 High-resolution mapping of spontaneous mitotic recombination hotspots on the 1.1 Mb arm of yeast chromosome IV. PLoS Genet. 9:e1003434.

Tamai, K. T., E. B. Gralla, L. M. Ellerby, J. S. Valentine, and D. J. Thiele, 1993 Yeast and mammalian metallothioneins functionally substitute for yeast copper-zinc superoxide dismutase. Proc Natl Acad Sci USA 90: 8013 -8017.

Thomas, B. J., and R. Rothstein, 1989 Elevated recombination rates in transcriptionally active DNA. Cell 56:619-630.

Wei, W., J. H. McCusker, R. W. Hyman, T. Jones, Y. Ning *et al.*, 2007 Genome sequencing and comparative analysis of *Saccharomyces cerevisiae* strain YJM789. Proc. Natl. Acad. Sci. USA 104:12825-12830.

Table S1 Strain genotypes.

Strain name	Genotype (reference)[1]
S288c	*MAT***a** *gal2* (Engel *et al.*, 2014)
W303-1A	*MAT***a** *leu2-3,112 his3-11,15 ura3-1 ade2-1 trp1-1 can1-100Δ::NAT RAD5* (Thomas and Rothstein, 1989)
YZ22	*MAT***a** *leu2-3,112 his3-11,15 ura3-1 ade2-1 trp1-1 can1-100Δ::NAT VIII212898::URA3 RAD5*
JSC10-1	*MAT***a** *leu2-3,112 his3-11,15 ura3-1 ade2-1 trp1-1 can1-100Δ::NAT ho::hisG RAD5* (St. Charles and Petes, 2013)
YJM789	*MATα lys2 gal2 ho::hisG* (Wei *et al.*, 2007)
YJM799	*MATα ura3 gal2 ho::hisG* (provided by J. McCusker, Duke Univ.)
JSC19-1	*MATα ade2-1 ura3 gal2 ho::hisG CAN1Δ::NAT* (St. Charles and Petes, 2013)
DTY3	*MATα trp1-1 leu2-3,112 gal1 ura3-50 his cup1*^{S} (Tamai *et al.*, 1993)
YJM189	Wild-type diploid from 100-genome strains (Strope *et al.*)
YJM271	Wild-type diploid from 100-genome strains (Strope *et al.*)
YJM456	Wild-type diploid from 100-genome strains (Strope *et al.*)
YJM693	Wild-type diploid from 100-genome strains (Strope *et al.*)
YJM969	Wild-type diploid from 100-genome strains (Strope *et al.*)
YJM972	Wild-type diploid from 100-genome strains (Strope *et al.*)
YJM978	Wild-type diploid from 100-genome strains (Strope *et al.*)
YJM996	Wild-type diploid from 100-genome strains (Strope *et al.*)

YJM1307	Wild-type diploid from 100-genome strains (Strope *et al.*)
YJM1549	Wild-type diploid from 100-genome strains (Strope *et al.*)
YZ22	*MAT***a** *leu2-3,112 his3-11,15 ura3-1 ade2-1 trp1-1 can1-100Δ::NAT VIII212898::URA3 RAD5*

[1]Strains from the 100-genome collection were provided by P. K. Strope, D. A. Skelly, S. G. Kozmin, G. Mahadevan, E. A. Stone, P. M. Magwene, F. S. Dietrich, and J. H. McCusker.

Table S2   Primer names and sequences used in strain constructions and analysis.

Primer name	Sequence
F1	CATTGGCACTCATGACCTTCA
R1	AATAAAGTATCTCCATATGTCGCC
R1'	AATCATGTAGCTGCCCAACGG
cup1 amp5-2	CGAGATGAAATGAATAGCAACGG
cup1 amp5-3	CTCCTTGTCTTGTATCAATTGCAT
cup1 amp3	TTCATTTCCCAGAGCAGCATGAC
VIII212898::URA3 F	TACAAGACAAGGAGTTATTTGCTTCTCTTTTATATG ATTCTGACAATCCATATTGCGTTGGTAGTCTTTTAA TGTGGCTGTGGTTTCAGG
VIII212898::URA3 R	ATTCTTTTGCTGGCATTTCTTCTAGAAGCAAAAAGA GCGATGCGTCTTTTCCGCTGAACCGTTCCAGCAAAG ATTCCCGGGTAATAACTG
VIII211849 F	GGAAATCGCCAATCCTTCCGAATT
VIII216603 R	TATCTCAACTGACCAAACTAGGCG
VIII212300 F	GAGTTGTAAGTTAGGCAAACTAGA
VIII213200 F	ATCATAGAAATCGTTGAAGTTTGC
VIII213031 R	CTGATATCTTAGCCTTGTTACTAG
VIII212063 R	TACATATGCACCGCACTCTATG
VIII210632 F	TGCTTCACCGTTGCGTCAATAA
VIII211528 F	AAAGGTTTACATGAATCAGTTGCC
VIII213234 F	CCCAGATTATCAGATTCCAAATCC

VIII213601 F	GCAATTGAACATTAATCTCCTCAT
VIII211185 F	CCAGTGCAACAGCGGTTAAG
VIII214195 R	GACATTCCTTTAATTGCTAACGAT
VIII213537 R	GAGATGAAATGAATAGCAACGGAAG
VIII210632 F	TGCTTCACCGTTGCGTCAATAA
VIII216314 F	GAAATCTTTTATCTGGAAGCTTAAC
VIII218008 R	TTTGAATATAACCTTGGCGTCCTA
VIII216763 F	TTGGTGGGAAGTTAACTTTGCAA
VIII216859 R	CACGATATCTGCCATATTAATCAG
VIII216894 R	GAGATCATTATCTTTTCAAGTTCTAT

Table S3 Sequence analysis of the *CUP1* repeats (Type 2, 1.8 kb) of YJM189.

In this table, we show genomic sequences of YJM189 in three regions: 1) the sequences that flank the *CUP1* repeats adjacent to *CIC1*, 2) the sequence of the *CUP1* repeat, and 3) the sequences that flank the *CUP1* tandem array adjacent to *RCS30*. The sequences of YJM189 (denoted “Query” below) were compared in a BLAST search with sequences of S288c (denoted “Sbject”). SNPs that distinguish YJM189 and S288c sequences are summarized at the end of the table. The *CUP1* coding sequences are shown in red. The names of the primers used in the sequence analysis are shown in boldface. Additional details about the sequencing are in Supporting Data File S1.

1. *CIC1-CUP1* (VIII211739-212339)

VIII211528 F

```
Query: 182    CTTGATGAACT 192
              |||||||||||
Sbjct: 211739 CTTGATGAACT 211749
```

```
Query: 193    TGAAGCTAAAAAGGACAAAATCGAAGAAACCCACGAAGATGACATGGTCACCAT 246
              ||||||||||||||||||||||||||||||||||||||||||||||||||||||
Sbjct: 211750 TGAAGCTAAAAAGGACAAAATCGAAGAAACCCACGAAGATGACATGGTCACCAT 211803
```

```
Query: 249    TGATGGTGTACAAGTTCATTTATCTACCTTCAACAAGGGTTTGATGGAAATCGCCAATCC
308
              ||||||||||||||||||||| ||||||||||||||||||||||||||||||||||||||
Sbjct: 211804 TGATGGTGTACAAGTTCATTTGTCTACCTTCAACAAGGGTTTGATGGAAATCGCCAATCC
211863
```

```
Query: 309    TTCCGAATTGGGTTCAATTTTCTCTAAACAAATTAACAATGCAAAAAAGAGATCTTCTAG
368
              ||||||||||||||||||||||||||||||||||||||||||||||||||||||||||||
Sbjct: 211864 TTCCGAATTGGGTTCAATTTTCTCTAAACAAATTAACAATGCAAAAAAGAGATCTTCTAG
211923
```

```
Query: 369    CGAGCTTGAAAAAGAATCTAGCGAGTCAGAAGCTGTCAAGAAGGCTAAAAGTTAATTTGT
428
              ||||||||||||||||||||||||||||||||||||||||||||||||||||||||||||
Sbjct: 211924 CGAGCTTGAAAAAGAATCTAGCGAGTCAGAAGCTGTCAAGAAGGCTAAAAGTTAATTTGT
211983


Query: 429    TTCCTCCTTATCTATCTTTTCTCTCATTTTTTTCTTGTGAAGAAAAAAATTTGAATTTCA
488
              ||||||||||||||||||||||||||||||||||||||||||||||||||||||||||||
Sbjct: 211984 TTCCTCCTTATCTATCTTTTCTCTCATTTTTTTCTTGTGAAGAAAAAAATTTGAATTTCA
212043


Query: 489    TAGAGTGCGGTGCATATGTATATATCTATATATGTTTGAAGTGTATATTAAAAATAAAGT
548
              ||||||||||||||||||||||||||||||||||||||||||||||||||||||||||||
Sbjct: 212044 TAGAGTGCGGTGCATATGTATATATCTATATATGTTTGAAGTGTATATTAAAAATAAAGT
212103
```

R1'

```
Query: 522    CATTATTTGAATATTGGTTTCTCGGTCTAAGAGCTTATACGTTTTAGACTG 454
              |||||||||||||||||||||||||||||||||||||||||||||||||||
Sbjct: 212104 CATTATTTGAATATTGGTTTCTCGGTCTAAGAGCTTATACGTTTTAGACTG 212154


Query: 453    ATCTGTTGTACTATCCGCTTCAAATAAATAGATCATTGAAAGTGACGGGGATAACAGCAT
394
              ||||||||||||||||||||||||||||||||||||||||||||||||||||||||||||
Sbjct: 212155 ATCTGTTGTACTATCCGCTTCAAATAAATAGATCATTGAAAGTGACGGGGATAACAGCAT
212214


Query: 393    TTTACCTTTAAAAGACGTTCTCATAATAGATTTTAGGATTAATACATATGCTTTTTTTTT
334
              |||||||||||||||||||||||||||| |||||||||||||||||||||||||||||||
Sbjct: 212215 TTTACCTTTAAAAGACGTTCTCATAATACATTTTAGGATTAATACATATGCTTTTTTTTT
212274


Query: 333    TATTCGAAATCTGGGGATTCTATACAGAGTTGTAAGTTAGGCAAACTAGAATTTGGTAAT
274
               |||||||||||||||||||||||||||||||||||||||||||||||||||||||||||
Sbjct: 212275 -ATTCGAAATCTGGGGATTCTATACAGAGTTGTAAGTTAGGCAAACTAGAATTTGGTAAT
212333


Query: 273    AATATT 268
              ||||||
Sbjct: 212334 AATATT 212339
```

<u>2. *CUP1* Repeat (VIII212039-213867)</u>

R1’

```
Query: 574    TTTCATAGAGTGCGGTGCATATGTATATATCTATATATGTTTGAAGTGTATATTAAAAAT
515
              ||||||||||||||||||||||||||||||||||||||||||||||||||||||||||||
Sbjct: 212039 TTTCATAGAGTGCGGTGCATATGTATATATCTATATATGTTTGAAGTGTATATTAAAAAT
212098


Query: 514    AAAGTCATTATTTGAATATTGGTTTCTCGGTCTAAGAGCTTATACGTTTTAGACTGATCT
455
              ||||||||||||||||||||||||||||||||||||||||||||||||||||||||||||
Sbjct: 212099 AAAGTCATTATTTGAATATTGGTTTCTCGGTCTAAGAGCTTATACGTTTTAGACTGATCT
212158


Query: 454    GTTGTACTATCCGCTTCAAATAAATAGATCATTGAAAGTGACGGGGATAACAGCATTTTA
395
              ||||||||||||||||||||||||||||||||||||||||||||||||||||||||||||
Sbjct: 212159 GTTGTACTATCCGCTTCAAATAAATAGATCATTGAAAGTGACGGGGATAACAGCATTTTA
212218


Query: 394    CCTTTAAAAGACGTTCTCATAATAGATTTTAGGATTAATACATATGCTTTTTTTTTTATT
335
              |||||||||||||||||||||||| ||||||||||||||||||||||||||||||| |||
Sbjct: 212219 CCTTTAAAAGACGTTCTCATAATACATTTTAGGATTAATACATATGCTTTTTTTTT-ATT
212277


Query: 334    CGAAATCTGGGGATTCTATACAGAGTTGTAAGTTAGGCAAACTAGAATTTGGTAATAATA
275
              ||||||||||||||||||||||||||||||||||||||||||||||||||||||||||||
Sbjct: 212278 CGAAATCTGGGGATTCTATACAGAGTTGTAAGTTAGGCAAACTAGAATTTGGTAATAATA
212337


Query: 274    TTTTATTCTTGGGGCGACATATGGAGATACTTTATTTCCTTTTCTTAATTATTAACGTAT
215
              ||||||||||||||||||||||||||||||||||||||||||||||||||||||||||||
Sbjct: 212338 TTTTATTCTTGGGGCGACATATGGAGATACTTTATTTCCTTTTCTTAATTATTAACGTAT
212397


Query: 214    ACCTATAAATTAACAAAGTATCTAAACAAAATACATAAGTGTACTCAAACTGAGTAGAAT
155
              ||||||||||||||||||||||||||||||||||||||||||||||||||||||||||||
Sbjct: 212398 ACCTATAAATTAACAAAGTATCTAAACAAAATACATAAGTGTACTCAAACTGAGTAGAAT
212457


Query: 154    CGTCGATTAAACTTCCTTCTCCTTTTAAAAATTAAAAACAGCAAATAGTTAGATGA 91
              ||||||||||||||||||||||||||||||||||||||||||||||||||||||||
Sbjct: 212458 CGTCGATTAAACTTCCTTCTCCTTTTAAAAATTAAAAACAGCAAATAGTTAGATGA 212513
```

VIII212300 F

```
Query: 187    ATATATTAAAGACTATTCGTTTCATTTCCCAGAGCAGCATGACTTCTTGGTTTCTTCAGA
246
              ||||||||||||||||||||||||||||||||||||||||||||||||||||||||||||
Sbjct: 212514 ATATATTAAAGACTATTCGTTTCATTTCCCAGAGCAGCATGACTTCTTGGTTTCTTCAGA
212573


Query: 247    CTTGTTACCGCAGGGGCATTTGTCGTCGCTGTTACACCCCGTTGGGCAGCTACATGATTT
306
              ||||||||||||||||||||||||||||||||||||||||||||||||||||||||||||
Sbjct: 212574 CTTGTTACCGCAGGGGCATTTGTCGTCGCTGTTACACCCCGTTGGGCAGCTACATGATTT
212633


Query: 307    TTGGCATTGTTCATTATTTTTGCAGCTACCACATTGGCATTGGCACTCATGACCTTCATT
366
              ||||||||||||||||||||||||||||||||||||||||||||||||||||||||||||
Sbjct: 212634 TTGGCATTGTTCATTATTTTTGCAGCTACCACATTGGCATTGGCACTCATGACCTTCATT
212693


Query: 367    TTGGAAGTTAATTAATTCGCTGAACATTTTATGTGATGATTGATTGATTG----TACAGT
422
              ||||||||||||||||||||||||||||||||||||||||||||||||||    ||||||
Sbjct: 212694 TTGGAAGTTAATTAATTCGCTGAACATTTTATGTGATGATTGATTGATTGATTGTACAGT
212753


Query: 423    TTGTTTTTCTTAATATCTATTTCGATGACTTCTATATGATATTGCACTAACAAGAAGATA
482
              ||||||||||||||||||||||||||||||||||||||||||||||||||||||||||||
Sbjct: 212754 TTGTTTTTCTTAATATCTATTTCGATGACTTCTATATGATATTGCACTAACAAGAAGATA
212813


Query: 483    TTATAATGCAATTGATACAAGACAAGGAGTTATTTGCTTCTCTTTTATATGATTCTGACA
542
              ||||||||||||||||||||||||||||||||||||||||||||||||||||||||||||
Sbjct: 212814 TTATAATGCAATTGATACAAGACAAGGAGTTATTTGCTTCTCTTTTATATGATTCTGACA
212873


Query: 543    ATCCATATTGCGTTGGTAGTCTTTTTTGCTGGAACGGTTCAGCGGAAAAGACGCATCGCT
602
              ||||||||||||||||||||||||||||||||||||||||||||||||||||||||||||
Sbjct: 212874 ATCCATATTGCGTTGGTAGTCTTTTTTGCTGGAACGGTTCAGCGGAAAAGACGCATCGCT
212933


Query: 603    CTTTTTGCTTCTAGAAGAAATGCCAGCAAAAGAATCTCTTGACAGTGACTGACAGCAAAA
662
              ||||||||||||||||||||||||||||||||||||||||||||||||||||||||||||
```

```
Sbjct: 212934 CTTTTTGCTTCTAGAAGAAATGCCAGCAAAAGAATCTCTTGACAGTGACTGACAGCAAAA
212993


Query: 663    ATGTCTT 669
              |||||||
Sbjct: 212994 ATGTCTT 213000
```

F1

```
Query: 297    TTTCTAACTAGTAACAAGGCTAAGATATCAGCCTGAAATAAAGGGTGGTGAAGTAATAAT
356
              ||||||||||||||||||||||||||||||||||||||||||||||||||||||||||||
Sbjct: 213001 TTTCTAACTAGTAACAAGGCTAAGATATCAGCCTGAAATAAAGGGTGGTGAAGTAATAAT
213060


Query: 357    TAAATCATCCGTATAAACCTATACACATATATGAGGAAAAATAATACAAAAGTGTTTTAA
416
              ||||||||||||||||||||||||||||||||||||||||||||||||||||||||||||
Sbjct: 213061 TAAATCATCCGTATAAACCTATACACATATATGAGGAAAAATAATACAAAAGTGTTTTAA
213120


Query: 417    ATACAGATACATACATGAACATATGCACGTATAGCGTCCAAATGTCGGTAATGGGATCGG
476
              |||||||||||||||||||||||||||||||||||| |||||||||||||||||||||||
Sbjct: 213121 ATACAGATACATACATGAACATATGCACGTATAGCGCCCAAATGTCGGTAATGGGATCGG
213180


Query: 477    CTTACTAATTATAAAATGCATCATAGAAATCGTTGAAGTTTGCCGTAGTAATACCCAGAT
536
              ||||||||||||||||||||||||||||||||||||||||||||||||||||||||||||
Sbjct: 213181 CTTACTAATTATAAAATGCATCATAGAAATCGTTGAAGTTTGCCGTAGTAATACCCAGAT
213240


Query: 537    TATCAGATTCCAAATCCTTGTCAATAATTATACTCCTTTGGAAAACTTCTCTTTCCATTA
596
              ||||||||||||||||||||||||||||||||||||||||||| ||||||||||||||||
Sbjct: 213241 TATCAGATTCCAAATCCTTGTCAATAATTATACTCCTTTGGACAACTTCTCTTTCCATTA
213300


Query: 597    AAAAATCTGAAATCTCCTTAAATTTTAAATAGATTCTGTTCAGTTCACTAACGGGGAATT
656
              ||||||||||||||||||||||||||||||||||||||||||||||||||||||||||||
Sbjct: 213301 AAAAATCTGAAATCTCCTTAAATTTTAAATAGATTCTGTTCAGTTCACTAACGGGGAATT
213360
```

VIII213200 F

```
Query: 116    TCAAGAGAACAT 127
              ||||||||||||
Sbjct: 213361 TCAAGAGAACAT 213372


Query: 128    TTTTGTTCTTCGCCGACTGACTATAATCTGTAACATTATTGTTATCAGAGTTTCTCGCAA
187
              ||||||||||||||||||||||||||||||||||||||||||||||||||||||||||||
Sbjct: 213373 TTTTGTTCTTCGCCGACTGACTATAATCTGTAACATTATTGTTATCAGAGTTTCTCGCAA
213432


Query: 188    AATTTTGTTTTTTCTTGCTAAATCTCAGCATATATTTAATCAGATTCAAAACCTTGTTGA
247
              ||||||||||||||||||||||||||||||||||||||||||||||||||||||||||||
Sbjct: 213433 AATTTTGTTTTTTCTTGCTAAATCTCAGCATATATTTAATCAGATTCAAAACCTTGTTGA
213492


Query: 248    AACCTTTAATAGATTTGAAATTTCCGTTGCTATTCATTTCATCTCGTAAAAAGGATACGA
307
              |||||||||||||||||||| |||||||||||||||||||||||||||||||||||||||
Sbjct: 213493 AACCTTTAATAGATTTGAAACTTCCGTTGCTATTCATTTCATCTCGTAAAAAGGATACGA
213552


Query: 308    TAATTTCTATTTTTTTTAAAATTTCCAAAATCTTGTCATGAATCAATAGCAATTGAACAT
367
              ||||||||||||||||||||||||||||||||||||||||||||||||||||||||||||
Sbjct: 213553 TAATTTCTATTTTTTTTAAAATTTCCAAAATCTTGTCATGAATCAATAGCAATTGAACAT
213612



Query: 368    TAATCTCCTCATTTGAAAGATTTTTGTAAAATTCGTCATATAATATTACTTCACAACGTT
427
              ||||||||||||||||||||||||||||||||||||||||||||||||||||||||||||
Sbjct: 213613 TAATCTCCTCATTTGAAAGATTTTTGTAAAATTCGTCATATAATATTACTTCACAACGTT
213672


Query: 428    GGAAAATAGCAAATGTGATTGCTATAAAATTCTGTAAGATTTCAATAAAATGATTTGCGA
487
              ||||||||||||||||||||||||||||||||||||||||||||||||||||||||||||
Sbjct: 213673 GGAAAATAGCAAATGTGATTGCTATAAAATTCTGTAAGATTTCAATAAAATGATTTGCGA
213732


Query: 488    ATAAAAATTCTTTACCATTAGAATGAAAGCGATTATTGCCGCTTGAAAATGACTTTATCG
547
              ||||||||||||||||||||||||||||||||||||||||||||||||||||||||||||
```

```
Sbjct: 213733 ATAAAAATTCTTTACCATTAGAATGAAAGCGATTATTGCCGCTTGAAAATGACTTTATCG
213792
```

R1’

```
Query: 649    ACTTTATGGGGAAGATAAAATTAA 626
              ||||||||||||||||||||||||
Sbjct: 213793 ACTTTATGGGGAAGATAAAATTAA 213816


Query: 625    ATGTTATTGAGTAAAAAATGTGCATATTAGAAATAATTTTCATCAGATCCT 561
              |||||||||||||||||||||||||||||||||||||||||||||||||||
Sbjct: 213817 ATGTTATTGAGTAAAAAATGTGCATATTAGAAATAATTTTCATCAGATCCT 213867
```

3. *CUP1-RSC30* (VIII213567-214167)

VIII213234 F

```
Query: 304    TTTAAAATTTCCAAAATCTTGTCATGAATCAATAGCAATTGAACATTAATCTCCTCATTT
363
              ||||||||||||||||||||||||||||||||||||||||||||||||||||||||||||
Sbjct: 213567 TTTAAAATTTCCAAAATCTTGTCATGAATCAATAGCAATTGAACATTAATCTCCTCATTT
213626


Query: 364    GAAAGATTTTTGTAAAATTCGTCATATAATATTACTTCACAACGTTGGAAAATAGCAAAT
423
              ||||||||||||||||||||||||||||||||||||||||||||||||||||||||||||
Sbjct: 213627 GAAAGATTTTTGTAAAATTCGTCATATAATATTACTTCACAACGTTGGAAAATAGCAAAT
213686


Query: 424    GTGATTGCTATAAAATTCTGTAAGATTTCAATAAAATGATTTGCGAATAAAAATTCTTTA
483
              ||||||||||||||||||||||||||||||||||||||||||||||||||||||||||||
Sbjct: 213687 GTGATTGCTATAAAATTCTGTAAGATTTCAATAAAATGATTTGCGAATAAAAATTCTTTA
213746


Query: 484    CCATTAGAATGAAAGCGATTATTGCCGCTTGAAAATGACTTTATCGACTTTATGGGGAAG
543
              ||||||||||||||||||||||||||||||||||||||||||||||||||||||||||||
Sbjct: 213747 CCATTAGAATGAAAGCGATTATTGCCGCTTGAAAATGACTTTATCGACTTTATGGGGAAG
213806


Query: 544    ATAAAATTAAATGTTACTGAGTAAAAAATGTGCATATTAGAAATAATTTTCATCAGATCC
603
              |||||||||||||||| |||||||||||||||||||||||||||||||||||||||||||
Sbjct: 213807 ATAAAATTAAATGTTATTGAGTAAAAAATGTGCATATTAGAAATAATTTTCATCAGATCC
213866
```

```
Query: 604    TTTGCACATCTTTCAGAGTTCGAGGTCTTATTGTTGTTAGAAGAATGTTGAACTGCCATG
663
              ||||||||||||||||||||||||||||||||||||||||||||||||||||||||||||
Sbjct: 213867 TTTGCACATCTTTCAGAGTTCGAGGTCTTATTGTTGTTAGAAGAATGTTGAACTGCCATG
213926


Query: 664    GACAAAGAGGATTCGTTTTGAACAAAAAGGAAAAAATTTGTATAAACAATGGTATTGATA
723
              ||||||||||||||||||||||||||||||||||||||||||||||||||||||||||||
Sbjct: 213927 GACAAAGAGGATTCGTTTTGAACAAAAAGGAAAAAATTTGTATAAACAATGGTATTGATA
213986
```

VIII216603 R

```
Query: 588    AAAT 585
              ||||
Sbjct: 213987 AAAT 213990


Query: 584    TTAAAGTGTCTTTCCATTCTTTTCTGACTTCGTTGTCATGAAAATATAAGTCTACTGTAT
525
              ||||||||||||||||||||||||||||||||||||||||||||||||||||||||||||
Sbjct: 213991 TTAAAGTGTCTTTCCATTCTTTTCTGACTTCGTTGTCATGAAAATATAAGTCTACTGTAT
214050


Query: 524    TACTCACGCCCATAGTCAAGGTTTCTAACAGACTTTCAATTTTGGTTAAATTTACTGGCA
465
              ||||||||||||||||||||||||||||||||||||||||||||||||||||||||||||
Sbjct: 214051 TACTCACGCCCATAGTCAAGGTTTCTAACAGACTTTCAATTTTGGTTAAATTTACTGGCA
214110


Query: 464    AGTAGAAAGGAACATCTTGCAGAATATTTATCAATTTTGCTTGCGTTTCCAGTAATT 402
              |||||||||||||| ||||||||||||||||||||||||||||||||||||||||||
Sbjct: 214111 AGTAGAAAGGAACACCTTGCAGAATATTTATCAATTTTGCTTGCGTTTCCAGTAATT
214167
```

SNPs between YJM189 and S288c

Sequenced interval	Coordinate(s)	SNP in YJM189	SNP in S288c
CIC1-CUP1 VIII211739-212339			
	211825	A	G
	212243	G	C
	212266-212274	10 T's	9 T's
CUP1 repeat VIII212039-213867			

	212243	G	C
	212266-212274	10 T's	9 T's
	212744-212747	4 bp deletion	ATTG
	213157	T	C
	213283	A	C
	213513	T	C
CUP1-RSC30<br>VIII213567-214167			
	213823	C	T
	214125	T	C

Table S4 Sequence analysis of the *CUP1* repeats (Type 2, 1.8 kb) of YJM996.

In this table, we show genomic sequences of YJM996 in three regions: 1) the sequences that flank the *CUP1* repeats adjacent to *CIC1*, 2) the sequence of the *CUP1* repeat, and 3) the sequences that flank the *CUP1* tandem array adjacent to *RCS30*. The sequences of YJM189 (denoted “Query” below) were compared in a BLAST search with sequences of S288c (denoted “Sbject”). SNPs that distinguish YJM189 and S288c sequences are summarized at the end of the table. The *CUP1* coding sequences are shown in red. The names of the primers used in the sequence analysis are shown in boldface. Additional details about the sequencing are in Supporting Data File S1.

1. *CIC1-CUP1* (VIII211739-212339)

R1’

```
Query: 863    CTTGATGAACTTGAAGCTAAA 843
              |||||||||||||||||||||
Sbjct: 211739 CTTGATGAACTTGAAGCTAAA 211759


Query: 842    AAGGACAAAATCGAAGAAACCCACGAAGATGACATGGTCACCATTGATGGTGTACAAGTT
783
              ||||||||||||||||||||||||||||||||||||||||||||||||||||||||||||
Sbjct: 211760 AAGGACAAAATCGAAGAAACCCACGAAGATGACATGGTCACCATTGATGGTGTACAAGTT
211819


Query: 782    CATTTATCTACCTTCAACAAGGGTTTGATGGAAATCGCCAATCCTTCCGAATTGGGTTCA
723
              ||||| ||||||||||||||||||||||||||||||||||||||||||||||||||||||
Sbjct: 211820 CATTTGTCTACCTTCAACAAGGGTTTGATGGAAATCGCCAATCCTTCCGAATTGGGTTCA
211879


Query: 722    ATTTTCTCTAAACAAATTAACAATGCAAAAAAGAGATCTTCTAGCGAGCTTGAAAAAGAA
663
              ||||||||||||||||||||||||||||||||||||||||||||||||||||||||||||
Sbjct: 211880 ATTTTCTCTAAACAAATTAACAATGCAAAAAAGAGATCTTCTAGCGAGCTTGAAAAAGAA
211939
```

```
Query: 662    TCTAGCGAGTCAGAAGCTGTCAAGAAGGCTAAAAGTTAATTTGTTTCCTCCTTATCTATC
603
              ||||||||||||||||||||||||||||||||||||||||||||||||||||||||||||
Sbjct: 211940 TCTAGCGAGTCAGAAGCTGTCAAGAAGGCTAAAAGTTAATTTGTTTCCTCCTTATCTATC
211999


Query: 602    TTTTCTCTCATTTTTTTCTTGTGAAGAAAAAAATTTGAATTTCATAGAGTGCGGTGCATA
543
              ||||||||||||||||||||||||||||||||||||||||||||||||||||||||||||
Sbjct: 212000 TTTTCTCTCATTTTTTTCTTGTGAAGAAAAAAATTTGAATTTCATAGAGTGCGGTGCATA
212059


Query: 542    TGTATATATCTATATATGTTTGAAGTGTATATTAAAAATAAAGTCATTATTTGAATATTG
483
              ||||||||||||||||||||||||||||||||||||||||||||||||||||||||||||
Sbjct: 212060 TGTATATATCTATATATGTTTGAAGTGTATATTAAAAATAAAGTCATTATTTGAATATTG
212119


Query: 482    GTTTCTCGGTCTAAGAGCTTATACGTTTTAGACTGATCTGTTGTACTATCCGCTTCAAAT
423
              ||||||||||||||||||||||||||||||||||||||||||||||||||||||||||||
Sbjct: 212120 GTTTCTCGGTCTAAGAGCTTATACGTTTTAGACTGATCTGTTGTACTATCCGCTTCAAAT
212179


Query: 422    AAATAGATCATTGAAAGTGACGGGGATAACAGCATTTTACCTTTAAAAGACGTTCTCATA
363
              ||||||||||||||||||||||||||||||||||||||||||||||||||||||||||||
Sbjct: 212180 AAATAGATCATTGAAAGTGACGGGGATAACAGCATTTTACCTTTAAAAGACGTTCTCATA
212239


Query: 362    ATAGATTTTAGGATTAATACATATGCTTTTTTTTTTATTCGAAATCTGGGGATTCTATAC
303
              ||| ||||||||||||||||||||||||||||||| ||||||||||||||||||||||||
Sbjct: 212240 ATACATTTTAGGATTAATACATATGCTTTTTTTTT-ATTCGAAATCTGGGGATTCTATAC
212298


Query: 302    AGAGTTGTAAGTTAGGCAAACTAGAATTTGGTAATAATATT 252
              |||||||||||||||||||||||||||||||||||||||||
Sbjct: 212299 AGAGTTGTAAGTTAGGCAAACTAGAATTTGGTAATAATATT 212339
```

2. *CUP1* repeat (VIII212039-213867)

R1’

```
Query: 572    TTTCATAGAGTGCGGTGCATATGTATATATCTATATATGTTTGAAGTGTATATTAAAAAT
513
              ||||||||||||||||||||||||||||||||||||||||||||||||||||||||||||
```

```
Sbjct: 212039 TTTCATAGAGTGCGGTGCATATGTATATATCTATATATGTTTGAAGTGTATATTAAAAAT
212098


Query: 512    AAAGTCATTATTTGAATATTGGTTTCTCGGTCTAAGAGCTTATACGTTTTAGACTGATCT
453
              ||||||||||||||||||||||||||||||||||||||||||||||||||||||||||||
Sbjct: 212099 AAAGTCATTATTTGAATATTGGTTTCTCGGTCTAAGAGCTTATACGTTTTAGACTGATCT
212158


Query: 452    GTTGTACTATCCGCTTCAAATAAATAGATCATTGAAAGTGACGGGGATAACAGCATTTTA
393
              ||||||||||||||||||||||||||||||||||||||||||||||||||||||||||||
Sbjct: 212159 GTTGTACTATCCGCTTCAAATAAATAGATCATTGAAAGTGACGGGGATAACAGCATTTTA
212218


Query: 392    CCTTTAAAAGACGTTCTCATAATAGATTTTAGGATTAATACATATGCTTTTTTTTTTATT
333
              |||||||||||||||||||||||| ||||||||||||||||||||||||||||||| |||
Sbjct: 212219 CCTTTAAAAGACGTTCTCATAATACATTTTAGGATTAATACATATGCTTTTTTTTT-ATT
212277


Query: 332    CGAAATCTGGGGATTCTATACAGAGTTGTAAGTTAGGCAAACTAGAATTTGGTAATAATA
273
              ||||||||||||||||||||||||||||||||||||||||||||||||||||||||||||
Sbjct: 212278 CGAAATCTGGGGATTCTATACAGAGTTGTAAGTTAGGCAAACTAGAATTTGGTAATAATA
212337


Query: 272    TTTTATTCTTGGGGCGACATATGGAGATACTTTATTTCCTTTTCTTAATTATTAACGTAT
213
              ||||||||||||||||||||||||||||||||||||||||||||||||||||||||||||
Sbjct: 212338 TTTTATTCTTGGGGCGACATATGGAGATACTTTATTTCCTTTTCTTAATTATTAACGTAT
212397


Query: 212    ACCTATAAATTAACAAAGTATCTAAACAAAATACATAAGTGTACTCAAACTGAGTAGAAT
153
              ||||||||||||||||||||||||||||||||||||||||||||||||||||||||||||
Sbjct: 212398 ACCTATAAATTAACAAAGTATCTAAACAAAATACATAAGTGTACTCAAACTGAGTAGAAT
212457
```

VIII212300 F

```
Query: 131    CGTCGATTAAACTTCCTTCTCCTTTTAAAAATTAAAAACAGCAAATAGTTAGATGA 186
              ||||||||||||||||||||||||||||||||||||||||||||||||||||||||
Sbjct: 212458 CGTCGATTAAACTTCCTTCTCCTTTTAAAAATTAAAAACAGCAAATAGTTAGATGA 212513


Query: 187    ATATATTAAAGACTATTCGTTTCATTTCCCAGAGCAGCATGACTTCTTGGTTTCTTCAGA
246
```

```
              ||||||||||||||||||||||||||||||||||||||||||||||||||||||||||||
Sbjct: 212514 ATATATTAAAGACTATTCGTTTCATTTCCCAGAGCAGCATGACTTCTTGGTTTCTTCAGA
212573

Query: 247    CTTGTTACCGCAGGGGCATTTGTCGTCGCTGTTACACCCCGTTGGGCAGCTACATGATTT
306
              ||||||||||||||||||||||||||||||||||||||||||||||||||||||||||||
Sbjct: 212574 CTTGTTACCGCAGGGGCATTTGTCGTCGCTGTTACACCCCGTTGGGCAGCTACATGATTT
212633

Query: 307    TTGGCATTGTTCATTATTTTTGCAGCTACCACATTGGCATTGGCACTCATGACCTTCATT
366
              ||||||||||||||||||||||||||||||||||||||||||||||||||||||||||||
Sbjct: 212634 TTGGCATTGTTCATTATTTTTGCAGCTACCACATTGGCATTGGCACTCATGACCTTCATT
212693

Query: 367    TTGGAAGTTAATTAATTCGCTGAACATTTTATGTGATGATTGATTGATTG----TACAGT
422
              ||||||||||||||||||||||||||||||||||||||||||||||||||    ||||||
Sbjct: 212694 TTGGAAGTTAATTAATTCGCTGAACATTTTATGTGATGATTGATTGATTGATTGTACAGT
212753

Query: 423    TTGTTTTTCTTAATATCTATTTCGATGACTTCTATATGATATTGCACTAACAAGAAGATA
482
              ||||||||||||||||||||||||||||||||||||||||||||||||||||||||||||
Sbjct: 212754 TTGTTTTTCTTAATATCTATTTCGATGACTTCTATATGATATTGCACTAACAAGAAGATA
212813

Query: 483    TTATAATGCAATTGATACAAGACAAGGAGTTATTTGCTTCTCTTTTATATGATTCTGACA
542
              ||||||||||||||||||||||||||||||||||||||||||||||||||||||||||||
Sbjct: 212814 TTATAATGCAATTGATACAAGACAAGGAGTTATTTGCTTCTCTTTTATATGATTCTGACA
212873
```

F1

```
Query: 171    ATCCATATTGCGTT 184
              ||||||||||||||
Sbjct: 212874 ATCCATATTGCGTT 212887

Query: 185    GGTAGTCTTTTTTGCTGGAACGGTTCAGCGGAAAAGACGCATCGCTCTTTTTGCTTCTAG
244
              ||||||||||||||||||||||||||||||||||||||||||||||||||||||||||||
Sbjct: 212888 GGTAGTCTTTTTTGCTGGAACGGTTCAGCGGAAAAGACGCATCGCTCTTTTTGCTTCTAG
212947

Query: 245    AAGAAATGCCAGCAAAAGAATCTCTTGACAGTGACTGACAGCAAAAATGTCTTTTTCTAA
304
              ||||||||||||||||||||||||||||||||||||||||||||||||||||||||||||
```

```
Sbjct: 212948 AAGAAATGCCAGCAAAAGAATCTCTTGACAGTGACTGACAGCAAAAATGTCTTTTTCTAA
213007


Query: 305    CTAGTAACAAGGCTAAGATATCAGCCTGAAATAAAGGGTGGTGAAGTAATAATTAAATCA
364
              ||||||||||||||||||||||||||||||||||||||||||||||||||||||||||||
Sbjct: 213008 CTAGTAACAAGGCTAAGATATCAGCCTGAAATAAAGGGTGGTGAAGTAATAATTAAATCA
213067


Query: 365    TCCGTATAAACCTATACACATATATGAGGAAAAATAATACAAAAGTGTTTTAAATACAGA
424
              ||||||||||||||||||||||||||||||||||||||||||||||||||||||||||||
Sbjct: 213068 TCCGTATAAACCTATACACATATATGAGGAAAAATAATACAAAAGTGTTTTAAATACAGA
213127


Query: 425    TACATACATGAACATATGCACGTATAGCGTCCAAATGTCGGTAATGGGATCGGCTTACTA
484
              ||||||||||||||||||||||||||||| ||||||||||||||||||||||||||||||
Sbjct: 213128 TACATACATGAACATATGCACGTATAGCGCCCAAATGTCGGTAATGGGATCGGCTTACTA
213187


Query: 485    ATTATAAAATGCATCATAGAAATCGTTGAAGTTTGCCGTAGTAATACCCAGATTATCAGA
544
              ||||||||||||||||||||||||||||||||||||||||||||||||||||||||||||
Sbjct: 213188 ATTATAAAATGCATCATAGAAATCGTTGAAGTTTGCCGTAGTAATACCCAGATTATCAGA
213247


Query: 545    TTCCAAATCCTTGTCAATAATTATACTCCTTTGGAAAACTTCTCTTTCCATTAAAAAATC
604
              ||||||||||||||||||||||||||||||||||| ||||||||||||||||||||||||
Sbjct: 213248 TTCCAAATCCTTGTCAATAATTATACTCCTTTGGACAACTTCTCTTTCCATTAAAAAATC
213307
```

VIII213200 F

```
Query: 84     TGAAATCTCCTTAAATTTTAAATAGATTCTGTTCAGTTCACTAAC 128
              |||||||||||||||||||||||||||||||||||||||||||||
Sbjct: 213308 TGAAATCTCCTTAAATTTTAAATAGATTCTGTTCAGTTCACTAAC 213352


Query: 129    GGGGAATTTCAAGAGAACATTTTTGTTCTTCGCCGACTGACTATAATCTGTAACATTATT
188
              ||||||||||||||||||||||||||||||||||||||||||||||||||||||||||||
Sbjct: 213353 GGGGAATTTCAAGAGAACATTTTTGTTCTTCGCCGACTGACTATAATCTGTAACATTATT
213412


Query: 189    GTTATCAGAGTTTCTCGCAAAATTTTGTTTTTTCTTGCTAAATCTCAGCATATATTTAAT
248
              ||||||||||||||||||||||||||||||||||||||||||||||||||||||||||||
```

```
Sbjct: 213413 GTTATCAGAGTTTCTCGCAAAATTTTGTTTTTTCTTGCTAAATCTCAGCATATATTTAAT
213472

Query: 249    CAGATTCAAAACCTTGTTGAAACCTTTAATAGATTTGAAATTTCCGTTGCTATTCATTTC
308
              ||||||||||||||||||||||||||||||||||||||||| |||||||||||||||||||
Sbjct: 213473 CAGATTCAAAACCTTGTTGAAACCTTTAATAGATTTGAAACTTCCGTTGCTATTCATTTC
213532

Query: 309    ATCTCGTAAAAAGGATACGATAATTTCTATTTTTTTTAAAATTTCCAAAATCTTGTCATG
368
              ||||||||||||||||||||||||||||||||||||||||||||||||||||||||||||
Sbjct: 213533 ATCTCGTAAAAAGGATACGATAATTTCTATTTTTTTTAAAATTTCCAAAATCTTGTCATG
213592

Query: 369    AATCAATAGCAATTGAACATTAATCTCCTCATTTGAAAGATTTTTGTAAAATTCGTCATA
428
              ||||||||||||||||||||||||||||||||||||||||||||||||||||||||||||
Sbjct: 213593 AATCAATAGCAATTGAACATTAATCTCCTCATTTGAAAGATTTTTGTAAAATTCGTCATA
213652

Query: 429    TAATATTACTTCACAACGTTGGAAAATAGCAAATGTGATTGCTATAAAATTCTGTAAGAT
488
              ||||||||||||||||||||||||||||||||||||||||||||||||||||||||||||
Sbjct: 213653 TAATATTACTTCACAACGTTGGAAAATAGCAAATGTGATTGCTATAAAATTCTGTAAGAT
213712

Query: 489    TTCAATAAAATGATTTGCGAATAAAAATTCTTTACCATTAGAATGAAAGCGATTATTGCC
548
              ||||||||||||||||||||||||||||||||||||||||||||||||||||||||||||
Sbjct: 213713 TTCAATAAAATGATTTGCGAATAAAAATTCTTTACCATTAGAATGAAAGCGATTATTGCC
213772

Query: 549    GCTTGAAAATGACTTTATCGACTTTATGGGGAAGATAAAATTAAATGTTATTGAGTAAAA
608
              ||||||||||||||||||||||||||||||||||||||||||||||||||||||||||||
Sbjct: 213773 GCTTGAAAATGACTTTATCGACTTTATGGGGAAGATAAAATTAAATGTTATTGAGTAAAA
213832

Query: 609    AATGTGCATATTAGAAATAATTTTCATCAGATCCT 643
              |||||||||||||||||||||||||||||||||||
Sbjct: 213833 AATGTGCATATTAGAAATAATTTTCATCAGATCCT 213867
```

3. *CUP1-RSC30* (VIII213567-214167)

VIII213200 F

```
Query: 342    TTTAAAATTTCCAAAATCTTGT 364
              ||||||||||||||||||||||
Sbjct: 213567 TTTAAAATTTCCAAAATCTTGT 213588


Query: 365    CATGAATCAATAGCAATTGAACATTAATCTCCTCATTTGAAAGATTTTTGTAAAATTCGT
424
              ||||||||||||||||||||||||||||||||||||||||||||||||||||||||||||
Sbjct: 213589 CATGAATCAATAGCAATTGAACATTAATCTCCTCATTTGAAAGATTTTTGTAAAATTCGT
213648


Query: 425    CATATAATATTACTTCACAACGTTGGAAAATAGCAAATGTGATTGCTATAAAATTCTGTA
484
              ||||||||||||||||||||||||||||||||||||||||||||||||||||||||||||
Sbjct: 213649 CATATAATATTACTTCACAACGTTGGAAAATAGCAAATGTGATTGCTATAAAATTCTGTA
213708


Query: 485    AGATTTCAATAAAATGATTTGCGAATAAAAATTCTTTACCATTAGAATGAAAGCGATTAT
544
              ||||||||||||||||||||||||||||||||||||||||||||||||||||||||||||
Sbjct: 213709 AGATTTCAATAAAATGATTTGCGAATAAAAATTCTTTACCATTAGAATGAAAGCGATTAT
213768


Query: 545    TGCCGCTTGAAAATGACTTTATCGACTTTATGGGGAAGATAAAATTAAATGTTATTGAGT
604
              ||||||||||||||||||||||||||||||||||||||||||||||||||||||||||||
Sbjct: 213769 TGCCGCTTGAAAATGACTTTATCGACTTTATGGGGAAGATAAAATTAAATGTTATTGAGT
213828


Query: 605    AAAAAATGTGCATATTAGAAATAATTTTCATCAGATCCTTTGCACATCTTTCAGAGTTCG
664
              ||||||||||||||||||||||||||||||||||||||||||||||||||||||||||||
Sbjct: 213829 AAAAAATGTGCATATTAGAAATAATTTTCATCAGATCCTTTGCACATCTTTCAGAGTTCG
213888


Query: 665    AGGTCTTATTGTTGTTAGAAGAATGTTGAACTGCCATGGACAAAGAGGATTCGTTTTGAA
724
              ||||||||||||||||||||||||||||||||||||||||||||||||||||||||||||
Sbjct: 213889 AGGTCTTATTGTTGTTAGAAGAATGTTGAACTGCCATGGACAAAGAGGATTCGTTTTGAA
213948
```

VIII216603 R

```
Query: 630    CAAAAAGGA 622
              |||||||||
Sbjct: 213949 CAAAAAGGA 213957
```

```
Query: 621    AAAAATTTGTATAAACAATGGTATTGATAAAATTTAAAGTGTCTTTCCATTCTTTTCTGA
562
              ||||||||||||||||||||||||||||||||||||||||||||||||||||||||||||
Sbjct: 213958 AAAAATTTGTATAAACAATGGTATTGATAAAATTTAAAGTGTCTTTCCATTCTTTTCTGA
214017


Query: 561    CTTCGTTGTCATGAAAATATAAGTCTACTGTATTACTCACGCCCATAGTCAAGGTTTCTA
502
              ||||||||||||||||||||||||||||||||||||||||||||||||||||||||||||
Sbjct: 214018 CTTCGTTGTCATGAAAATATAAGTCTACTGTATTACTCACGCCCATAGTCAAGGTTTCTA
214077


Query: 501    ACAGACTTTCAATTTTGGTTAAATTTACTGGCAAGTAGAAAGGAACATCTTGCAGAATAT
442
              ||||||||||||||||||||||||||||||||||||||||||||||| ||||||||||||
Sbjct: 214078 ACAGACTTTCAATTTTGGTTAAATTTACTGGCAAGTAGAAAGGAACACCTTGCAGAATAT
214137


Query: 441    TTATCAATTTTGCTTGCGTTTCCGGTAATTTTAAATCGTTAGCAATTAAA 392
              ||||||||||||||||||||||| ||||||||||||||||||||||||||
Sbjct: 214138 TTATCAATTTTGCTTGCGTTTCCAGTAATTTTAAATCGTTAGCAATTAAA 214187
```

SNPs between YJM996 and S288c

Sequenced interval	Coordinate(s)	SNP in YJM996	SNP in S288c
CIC1-CUP1 VIII211739-212339			
	211825	A	G
	212243	G	C
	212266-212274	10 T's	9 T's
CUP1 repeat VIII212039-213867			
	212243	G	C
	212266-212274	10 T's	9 T's
	212744-212747	4 bp deletion	ATTG
	213157	T	C
	213283	A	C
	213513	T	C
CUP1-RSC30 VIII213567-214167			
	214125	T	C
	214161	G	A

Table S5 Sequence analysis of the *CUP1* repeats (Type 3, 1.2 kb) of YJM789.

In this table, we show genomic sequences of YJM789 in three regions: 1) the sequences that flank the *CUP1* repeats adjacent to *CIC1*, 2) the sequence of the *CUP1* repeat, and 3) the sequences that flank the *CUP1* tandem array adjacent to *RCS30*. The sequences of YJM789 (denoted "Query" below) were compared in a BLAST search with sequences of S288c (denoted "Sbject"). SNPs that distinguish YJM789 and S288c sequences are summarized at the end of the table. The *CUP1* coding sequences are shown in red. The names of the primers used in the sequence analysis are shown in boldface. Additional details about the sequencing are in Supporting Data File S1.

1. *CIC1-CUP1* (VIII211730-212330)

VIII211528 F

```
Query: 167    CAGGACGTTCTTGAT 181
              |||||||||||||||
Sbjct: 211730 CAGGACGTTCTTGAT 211744


Query: 182    GAACTTGAAGCTAAAAAGGACAAAATCGAAGAAACCCACGAAGATGACATGGTCACCATT
241
              ||||||||||||||||||||||||||||||||||||||||||||||||||||||||||||
Sbjct: 211745 GAACTTGAAGCTAAAAAGGACAAAATCGAAGAAACCCACGAAGATGACATGGTCACCATT
211804


Query: 242    GATGGTGTACAAGTTCATTTGTCTACCTTCAACAAGGGTTTGATGGAAATCGCCAATCCT
301
              ||||||||||||||||||||||||||||||||||||||||||||||||||||||||||||
Sbjct: 211805 GATGGTGTACAAGTTCATTTGTCTACCTTCAACAAGGGTTTGATGGAAATCGCCAATCCT
211864


Query: 302    TCCGAATTGGGTTCAATTTTCTCTAAACAAATTAACAATGCAAAAAAGAGATCTTCTAGC
361
              ||||||||||||||||||||||||||||||||||||||||||||||||||||||||||||
Sbjct: 211865 TCCGAATTGGGTTCAATTTTCTCTAAACAAATTAACAATGCAAAAAAGAGATCTTCTAGC
211924


Query: 362    GAGCTTGAAAAAGAATCTAGCGAGTCAGAAGCTGTCAAGAAGGCTAAAAGTTAATTTGTT
421
```

```
               ||||||||||||||||||||||||||||||||||||||||||||||||||||||||||||
Sbjct: 211925 GAGCTTGAAAAAGAATCTAGCGAGTCAGAAGCTGTCAAGAAGGCTAAAAGTTAATTTGTT
211984


Query: 422    TCCTCCTTATCTATCTTTTCTCTCATTTTTTTCTTGTGAAGAAAAAAATTTGAATTTCAT
481
              ||||||||||||||||||||||||||||||||||||||||||||||||||||||||||||
Sbjct: 211985 TCCTCCTTATCTATCTTTTCTCTCATTTTTTTCTTGTGAAGAAAAAAATTTGAATTTCAT
212044


Query: 482    AGAGTGCGGTGCATATGTATATATCTATATATGTTTGAAGTGTATATTAAAAATAAAGTC
541
              ||||||||||||||||||||||||||||||||||||||||||||||||||||||||||||
Sbjct: 212045 AGAGTGCGGTGCATATGTATATATCTATATATGTTTGAAGTGTATATTAAAAATAAAGTC
212104




Query: 542    ATTATTTGAATATTGGTTTCTCGGTCTAAGAGCTTATACGTTTTAGACTGATCTGTTGTA
601
              ||||||||||||||||||||||||||||||||||||||||||||||||||||||||||||
Sbjct: 212105 ATTATTTGAATATTGGTTTCTCGGTCTAAGAGCTTATACGTTTTAGACTGATCTGTTGTA
212164


Query: 602    CTATCCGCTTCAAATAAATAGATCATTGAAAGTGACGGGGATAACAGCATTTTACCTTTA
661
              ||||||||||||||||||||||||||||||||||||||||||||||||||||||||||||
Sbjct: 212165 CTATCCGCTTCAAATAAATAGATCATTGAAAGTGACGGGGATAACAGCATTTTACCTTTA
212224


Query: 378    AAAGACGTTCTCATAATACATTTTAGGATTAATACATATGCTTTTTTTTT 328
              ||||||||||||||||||||||||||||||||||||||||||||||||||
Sbjct: 212225 AAAGACGTTCTCATAATACATTTTAGGATTAATACATATGCTTTTTTTT- 212274


Query: 327    ATTCGAAATCTGGGGATTTTATACAGAGTTGTAAGTTAGGCAAACTAGAATTTGGT 276
              |||||||||||||||||| |||||||||||||||||||||||||||||||||||||
Sbjct: 212275 ATTCGAAATCTGGGGATTCTATACAGAGTTGTAAGTTAGGCAAACTAGAATTTGGT 212330
```

2. *CUP1* repeat (VIII212030-213192)

F1

```
Query: 489    AAATTTGAATTTCATAGAGTGCGGTGCATATGTATATATCTATATATGTTTGAAGTGT 546
              ||||||||||||||||||||||||||||||||||||||||||||||||||||||||||
Sbjct: 212030 AAATTTGAATTTCATAGAGTGCGGTGCATATGTATATATCTATATATGTTTGAAGTGT
212087
```

```
Query: 547    ATATTAAAAATAAAGTCATTATTTGAATATTGGTTTCTCGGTCTAAGAGCTTATACGTTT
606
              ||||||||||||||||||||||||||||||||||||||||||||||||||||||||||||
Sbjct: 212088 ATATTAAAAATAAAGTCATTATTTGAATATTGGTTTCTCGGTCTAAGAGCTTATACGTTT
212147
```

R1’

```
Query: 456    TAGACTGATCTGTTGTACTATCCGCTTCAAATAAATAGATCATTGAAAGTGACGGGGATA
397
              ||||||||||||||||||||||||||||||||||||||||||||||||||||||||||||
Sbjct: 212148 TAGACTGATCTGTTGTACTATCCGCTTCAAATAAATAGATCATTGAAAGTGACGGGGATA
212207


Query: 396    ACAGCATTTTACCTTTAAAAGACGTTCTCATAATACATTTTAGGATTAATACATATGCTT
337
              ||||||||||||||||||||||||||||||||||||||||||||||||||||||||||||
Sbjct: 212208 ACAGCATTTTACCTTTAAAAGACGTTCTCATAATACATTTTAGGATTAATACATATGCTT
212267


Query: 336    TTTTTTTTATTCGAAATCTGGGGATTTTATACAGAGTTGTAAGTTAGGCAAACTAGAATT
277
              ||||||| |||||||||||||||||| |||||||||||||||||||||||||||||||||
Sbjct: 212268 TTTTTTT-ATTCGAAATCTGGGGATTCTATACAGAGTTGTAAGTTAGGCAAACTAGAATT
212326


Query: 276    TGGTAATAATATTTTATTCTTGGGGCGACATATGGAGATACTTTATTTCCTTTTCTTAAT
217
              ||||||||||||||||||||||||||||||||||||||||||||||||||||||||||||
Sbjct: 212327 TGGTAATAATATTTTATTCTTGGGGCGACATATGGAGATACTTTATTTCCTTTTCTTAAT
212386


Query: 216    TATTAACGTATACCTATAAATTAACAAAGTATCTAAACAAAATACATAAGTGTACTCAAA
157
              ||||||||||||||||||||||||||||||||||||||||||||||||||||||||||||
Sbjct: 212387 TATTAACGTATACCTATAAATTAACAAAGTATCTAAACAAAATACATAAGTGTACTCAAA
212446


Query: 156    CTGAGTAGAATCGTCGATTAAACTTCCTTCTCCTTTTAAAAATTAAAAACAGTAAATAGT 97
              |||||||||||||||||||||||||||||||||||||||||||||||||||| |||||||
Sbjct: 212447 CTGAGTAGAATCGTCGATTAAACTTCCTTCTCCTTTTAAAAATTAAAAACAGCAAATAGT
212506
```

VIII212300 F

```
Query: 180    TAAATGAA 187
              || |||||
Sbjct: 212507 TAGATGAA 212514
```

```
Query: 188    TATATTAAAGACTATTCGTTTTATTTCCCAGAGCAGCATGATTTCTTGGTTTCTTCAGAC
247
              ||||||||||||||||||||| ||||||||||||||||||| ||||||||||||||||||
Sbjct: 212515 TATATTAAAGACTATTCGTTTCATTTCCCAGAGCAGCATGACTTCTTGGTTTCTTCAGAC
212574

Query: 248    TTGTTACCGCAGGGGCATTTGTCGTCGCTGTTACACCCCGTTGGGCAGCTACATGATTTT
307
              ||||||||||||||||||||||||||||||||||||||||||||||||||||||||||||
Sbjct: 212575 TTGTTACCGCAGGGGCATTTGTCGTCGCTGTTACACCCCGTTGGGCAGCTACATGATTTT
212634

Query: 308    TGGCATTGTTCATTATTTTTGCAGCTACCACATTGGCATTGGCACTCATGACCTTCATTT
367
              ||||||||||||||||||||||||||||||||||||||||||||||||||||||||||||
Sbjct: 212635 TGGCATTGTTCATTATTTTTGCAGCTACCACATTGGCATTGGCACTCATGACCTTCATTT
212694

Query: 368    TGGAAGTTAATTAATTCGCTGAACATTTTATGTGATGATTGATTGATTG----TACGGTT
423
              |||||||||||||||||||||||||||||||||||||||||||||||||    ||| |||
Sbjct: 212695 TGGAAGTTAATTAATTCGCTGAACATTTTATGTGATGATTGATTGATTGATTGTACAGTT
212754

Query: 424    TGTTTTTGTTAATATCTATTTCGATGACTTCTATATGATATTGCACTAACAAGAAGATAT
483
              ||||||| ||||||||||||||||||||||||||||||||||||||||||||||||||||
Sbjct: 212755 TGTTTTTCTTAATATCTATTTCGATGACTTCTATATGATATTGCACTAACAAGAAGATAT
212814

Query: 484    TATAATGCAATTGGTACAAGACAAGGAGTTATTTGCTTCTCTTTTATATGATTCTGACAA
543
              ||||||||||||| ||||||||||||||||||||||||||||||||||||||||||||||
Sbjct: 212815 TATAATGCAATTGATACAAGACAAGGAGTTATTTGCTTCTCTTTTATATGATTCTGACAA
212874

Query: 544    TCCATATTGCGTTGGTAGTCTTTTTTGCTGGAACGGTTCAGCGGAAAAGACGCATCGCTC
603
              ||||||||||||||||||||||||||||||||||||||||||||||||||||||||||||
Sbjct: 212875 TCCATATTGCGTTGGTAGTCTTTTTTGCTGGAACGGTTCAGCGGAAAAGACGCATCGCTC
212934

Query: 604    TTTTTGCTTCTAGAAGAAATGCCAGCAAAAGAATCTCTTGACAGTGACTGACAGCAAAAA
663
              ||||||||||||||||||||||||||||||||||||||||||||||||||||||||||||
Sbjct: 212935 TTTTTGCTTCTAGAAGAAATGCCAGCAAAAGAATCTCTTGACAGTGACTGACAGCAAAAA
212994
```

F1

```
Query: 290    TGTCTTTTT 298
              |||||||||
Sbjct: 212995 TGTCTTTTT 213003
```

```
Query: 299    CTAACTAGTAACAAGGCTAAGATATCAGCCTGAAATAAAGGGTGGTGAAGTAATAATTAA
358
              ||||||||||||||||||||||||||||||||||||||||||||||||||||||||||||
Sbjct: 213004 CTAACTAGTAACAAGGCTAAGATATCAGCCTGAAATAAAGGGTGGTGAAGTAATAATTAA
213063
```

```
Query: 359    ATCATCCGTATAAACCTATACACATATATGAGGAAAAAATAATACAAAGTGTTTTAAAT
418
              ||||||||||||||||||||||||||||||||||||||| ||||||||||||||||||||
Sbjct: 213064 ATCATCCGTATAAACCTATACACATATATGAGGAAAAA-TAATACAAAGTGTTTTAAAT
213122
```

```
Query: 419    ACAGATACATACATGAACATATGCACGTATAGCGTCCAAATGTCGGTAATGGGATCGGCT
478
              |||||||||||||||||||||||||||||||||| |||||||||||||||||||||||||
Sbjct: 213123 ACAGATACATACATGAACATATGCACGTATAGCGCCCAAATGTCGGTAATGGGATCGGCT
213182
```

```
Query: 479    TACTAATTAT 522
              ||||||||||
Sbjct: 213183 TACTAATTAT 213192
```

3. *CUP1-RSC30* (VIII212892-213492)

F1

```
Query: 188    GTCTTTTTTGCTGGAACGGTTCAGCGGAAAAGACGCATCGCTCTTTTTGCTT 239
              ||||||||||||||||||||||||||||||||||||||||||||||||||||
Sbjct: 212892 GTCTTTTTTGCTGGAACGGTTCAGCGGAAAAGACGCATCGCTCTTTTTGCTT 212943
```

```
Query: 240    CTAGAAGAAATGCCAGCAAAAGAATCTCTTGACAGTGACTGACAGCAAAAATGTCTTTTT
299
              ||||||||||||||||||||||||||||||||||||||||||||||||||||||||||||
Sbjct: 212944 CTAGAAGAAATGCCAGCAAAAGAATCTCTTGACAGTGACTGACAGCAAAAATGTCTTTTT
213003
```

```
Query: 300    CTAACTAGTAACAAGGCTAAGATATCAGCCTGAAATAAAGGGTGGTGAAGTAATAATTAA
359
              ||||||||||||||||||||||||||||||||||||||||||||||||||||||||||||
```

```
Sbjct: 213004 CTAACTAGTAACAAGGCTAAGATATCAGCCTGAAATAAAGGGTGGTGAAGTAATAATTAA
213063

Query: 360    ATCATCCGTATAAACCTATACACATATATGAGGAAAAAATAATACAAAAGTGTTTTAAAT
419
              ||||||||||||||||||||||||||||||||||||||| |||||||||||||||||||||
Sbjct: 213064 ATCATCCGTATAAACCTATACACATATATGAGGAAAAA-TAATACAAAAGTGTTTTAAAT
213122

Query: 420    ACAGATACATACATGAACATATGCACGTATAGCGTCCAAATGTCGGTAATGAGATCGGCT
479
              |||||||||||||||||||||||||||||||||| |||||||||||||||| ||||||||
Sbjct: 213123 ACAGATACATACATGAACATATGCACGTATAGCGCCCAAATGTCGGTAATGGGATCGGCT
213182

Query: 480    TACTAATTATAAAATGCATCATAGAAATCGTTGAAGTTTGCCGTAGTAATACCCAGATTA
539
              ||||||||||||||||||||||||||||||||||||||||||||||||||||||||||||
Sbjct: 213183 TACTAATTATAAAATGCATCATAGAAATCGTTGAAGTTTGCCGTAGTAATACCCAGATTA
213242

Query: 540    TCAGATTCCAAATCCTTGTCAATAATTATACTCCTTTGGAAAACTTCTCTTTCCATTAAA
599
              ||||||||||||||||||||||||||||||||||||||||| ||||||||||||||||||
Sbjct: 213243 TCAGATTCCAAATCCTTGTCAATAATTATACTCCTTTGGACAACTTCTCTTTCCATTAAA
213302


Query: 600    AAATCTGAAATCTCCTTAAATTTTAAATAGATTCTGTTCAGTTCACTAACGGGGAATTTC
659
              ||||||||||||||||||||||||||||||||||||||||||||||||||||||||||||
Sbjct: 213303 AAATCTGAAATCTCCTTAAATTTTAAATAGATTCTGTTCAGTTCACTAACGGGGAATTTC
213362

Query: 660    AAGAGAACATTTTTGTTCTTCGCCGACTGACTATAATCTGTAACATTATTATTATCAGAG
719
              |||||||||||||||||||||||||||||||||||||||||||||||||| |||||||||
Sbjct: 213363 AAGAGAACATTTTTGTTCTTCGCCGACTGACTATAATCTGTAACATTATTGTTATCAGAG
213422

Query: 720    TTTCTCGCAAAATTTTGTTTTTTCTTGCTAAATCTCAGCATATATTTAATCAGATTCAAA
779
              ||||||||||||||||||||||||||||||||||||||||||||||||||||||||||||
Sbjct: 213423 TTTCTCGCAAAATTTTGTTTTTTCTTGCTAAATCTCAGCATATATTTAATCAGATTCAAA
213482


Query: 780    ACCTTGTTGA 789
              ||||||||||
```

```
Sbjct: 213483 ACCTTGTTGA 213492
```

SNPs between YJM789 and S288c

Sequenced interval	Coordinate(s)	SNP in YJM789	SNP in S288c
CIC1-CUP1 VIII211728-212328			
	212266-212274	10 T's	9 T's
	212293	T	C
CUP1 repeat VIII212028-213190			
	212266-212274	10 T's	9 T's
	212293	T	C
	212499	T	C
	212509	A	G
	212536	T	C
	212556	T	C
	212744-212747	4 bp deletion	ATTG
	212751	G	A
	212762	G	C
	212828	G	A
	213097-213101	6 A's	5 A's
	213157	T	C
CUP1-RSC30 VIII212890-213490			
	213097-213101	6 A's	5 A's
	213157	T	C
	213174	A	G
	213283	A	C
	213413	A	G

Table S6 Sequence analysis of the *CUP1* repeats (Type 4, 1.9 kb) of YJM271.

In this table, we show genomic sequences of YJM271 in three regions: 1) the sequences that flank the *CUP1* repeats adjacent to *CIC1*, 2) the sequence of the *CUP1* repeat, and 3) the sequences that flank the *CUP1* tandem array adjacent to *RCS30*. The sequences of YJM271 (denoted "Query" below) were compared in a BLAST search with sequences of S288c (denoted "Sbject"). SNPs that distinguish YJM271 and S288c sequences are summarized at the end of the table. The *CUP1* coding sequences are shown in red. The names of the primers used in the sequence analysis are shown in boldface. Additional details about the sequencing are in Supporting Data File S1.

1. ***CIC1-CUP1*** (VIII211758-212358)

VIII211528 F

```
Query: 202    AAAAGGACAAAATCGAAGAAACCCACGAAGATGACATGGTCACCAT 247
              ||||||||||||||||||||||||||||||||||||||||||||||
Sbjct: 211758 AAAAGGACAAAATCGAAGAAACCCACGAAGATGACATGGTCACCAT 211803


Query: 248    TGATGGTGTACAAGTTCATTTATCTACCTTCAACAAGGGTTTGATGGAAATCGCCAATCC
307
              ||||||||||||||||||||| ||||||||||||||||||||||||||||||||||||||
Sbjct: 211804 TGATGGTGTACAAGTTCATTTGTCTACCTTCAACAAGGGTTTGATGGAAATCGCCAATCC
211863


Query: 308    TTCCGAATTGGGTTCAATTTTCTCTAAACAAATTAACAATGCAAAAAAGAGATCTTCTAG
367
              ||||||||||||||||||||||||||||||||||||||||||||||||||||||||||||
Sbjct: 211864 TTCCGAATTGGGTTCAATTTTCTCTAAACAAATTAACAATGCAAAAAAGAGATCTTCTAG
211923


Query: 368    CGAGCTTGAAAAAGAATCTAGCGAGTCAGAAGCTGTCAAGAAGGCTAAAAGTTAATTTGT
427
              ||||||||||||||||||||||||||||||||||||||||||||||||||||||||||||
Sbjct: 211924 CGAGCTTGAAAAAGAATCTAGCGAGTCAGAAGCTGTCAAGAAGGCTAAAAGTTAATTTGT
211983
```

```
Query: 428    TTCCTCCTTATCTATCTTTTCTCTCATTTTTTTCTTGTGAAGAAAAAAATTTGAATTTCA
487
              ||||||||||||||||||||||||||||||||||||||||||||||||||||||||||||
Sbjct: 211984 TTCCTCCTTATCTATCTTTTCTCTCATTTTTTTCTTGTGAAGAAAAAAATTTGAATTTCA
212043


Query: 488    TAGAGTGCGGTGCATATGTATATATCTATATATGTTTGAAGTGTATATTAAAAATAAAGT
547
              ||||||||||||||||||||||||||||||||||||||||||||||||||||||||||||
Sbjct: 212044 TAGAGTGCGGTGCATATGTATATATCTATATATGTTTGAAGTGTATATTAAAAATAAAGT
212103


Query: 548    CATTATTTGAATATTGGTTTCTCGGTCTAAGAGCTTATACGTTTTAGACTGATCTGTTGT
607
              ||||||||||||||||||||||||||||||||||||||||||||||||||||||||||||
Sbjct: 212104 CATTATTTGAATATTGGTTTCTCGGTCTAAGAGCTTATACGTTTTAGACTGATCTGTTGT
212163
```

R1’

```
Query: 444    ACTATCCGCTTCAAATAAATAGATCAT 418
              |||||||||||||||||||||||||||
Sbjct: 212164 ACTATCCGCTTCAAATAAATAGATCAT 212190


Query: 417    TGAAAGTGACGGGGATAACAGCATTTTACCTTTAAAAGACGTTCTCATAATAGATTTTAG
358
              |||||||||||||||||||||||||||||||||||||||||||||||||||| |||||||
Sbjct: 212191 TGAAAGTGACGGGGATAACAGCATTTTACCTTTAAAAGACGTTCTCATAATACATTTTAG
212250


Query: 357    GATTAATACATATGCTTTTTTTTTTATTCGAAATCTGGGGATTCTATACAGAGTTGTAAG
298
              |||||||||||||||||||||||| |||||||||||||||||||||||||||||||||||
Sbjct: 212251 GATTAATACATATGCTTTTTTTTT-ATTCGAAATCTGGGGATTCTATACAGAGTTGTAAG
212309


Query: 297    TTAGGCAAACTAGAATTTGGTAATAATATTTTATTCTTGGGGCGACATA 249
              |||||||||||||||||||||||||||||||||||||||||||||||||
Sbjct: 212310 TTAGGCAAACTAGAATTTGGTAATAATATTTTATTCTTGGGGCGACATA 212358
```

2*. CUP1* repeat (VIII212058-213988)

R1’

```
Query: 555    TATGTATATATCTATATATGTTT 533
              |||||||||||||||||||||||
Sbjct: 212058 TATGTATATATCTATATATGTTT 212080
```

```
Query: 532    GAAGTGTATATTAAAAATAAAGTCATTATTTGAATATTGGTTTCTCGGTCTAAGAGCTTA
473
              ||||||||||||||||||||||||||||||||||||||||||||||||||||||||||||
Sbjct: 212081 GAAGTGTATATTAAAAATAAAGTCATTATTTGAATATTGGTTTCTCGGTCTAAGAGCTTA
212140

Query: 472    TACGTTTTAGACTGATCTGTTGTACTATCCGCTTCAAATAAATAGATCATTGAAAGTGAC
413
              ||||||||||||||||||||||||||||||||||||||||||||||||||||||||||||
Sbjct: 212141 TACGTTTTAGACTGATCTGTTGTACTATCCGCTTCAAATAAATAGATCATTGAAAGTGAC
212200

Query: 412    GGGGATAACAGCATTTTACCTTTAAAAGACGTTCTCATAATAGATTTTAGGATTAATACA
353
              |||||||||||||||||||||||||||||||||||||||||| |||||||||||||||||
Sbjct: 212201 GGGGATAACAGCATTTTACCTTTAAAAGACGTTCTCATAATACATTTTAGGATTAATACA
212260

Query: 352    TATGCTTTTTTTTTTATTCGAAATCTGGGGATTCTATACAGAGTTGTAAGTTAGGCAAAC
293
              |||||||||||||| |||||||||||||||||||||||||||||||||||||||||||||
Sbjct: 212261 TATGCTTTTTTTTT-ATTCGAAATCTGGGGATTCTATACAGAGTTGTAAGTTAGGCAAAC
212319

Query: 292    TAGAATTTGGTAATAATATTTTATTCTTGGGGCGACATATGGAGATACTTTATTTCCTTT
233
              ||||||||||||||||||||||||||||||||||||||||||||||||||||||||||||
Sbjct: 212320 TAGAATTTGGTAATAATATTTTATTCTTGGGGCGACATATGGAGATACTTTATTTCCTTT
212379

Query: 232    TCTTAATTATTAACGTATACCTATAAATTAACAAAGTATCTAAACAAAATACATAAGTGT
173
              ||||||||||||||||||||||||||||||||||||||||||||||||||||||||||||
Sbjct: 212380 TCTTAATTATTAACGTATACCTATAAATTAACAAAGTATCTAAACAAAATACATAAGTGT
212439

Query: 172    ACTCAAACTGAGTAGAATCGTCGATTAAACTTCCTTCTCCTTTTAAAAATTAAAAACAGC
113
              ||||||||||||||||||||||||||||||||||||||||||||||||||||||||||||
Sbjct: 212440 ACTCAAACTGAGTAGAATCGTCGATTAAACTTCCTTCTCCTTTTAAAAATTAAAAACAGC
212499
```

VIII212300 F

```
Query: 173    AAATAGTTAGATGAA 187
              |||||||||||||||
```

```
Sbjct: 212500 AAATAGTTAGATGAA 212514


Query: 188    TATATTAAAGACTATTCGTTTCATTTCCCAGAGCAGCATGACTTCTTGGTTTCTTCAGAC
247
              ||||||||||||||||||||||||||||||||||||||||||||||||||||||||||||
Sbjct: 212515 TATATTAAAGACTATTCGTTTCATTTCCCAGAGCAGCATGACTTCTTGGTTTCTTCAGAC
212574


Query: 248    TTGTTACCGCAGGGGCATTTGTCGTCGCTGTTACACCCCGTTGGGCAGCTACATGATTTT
307
              ||||||||||||||||||||||||||||||||||||||||||||||||||||||||||||
Sbjct: 212575 TTGTTACCGCAGGGGCATTTGTCGTCGCTGTTACACCCCGTTGGGCAGCTACATGATTTT
212634


Query: 308    TGGCATTGTTCATTATTTTTGCAGCTACCACATTGGCATTGGCACTCATGACCTTCATTT
367
              ||||||||||||||||||||||||||||||||||||||||||||||||||||||||||||
Sbjct: 212635 TGGCATTGTTCATTATTTTTGCAGCTACCACATTGGCATTGGCACTCATGACCTTCATTT
212694


Query: 368    TGGAAGTTAATTAATTCGCTGAACATTTTATGTGATGATTGATTGATTG----TACGGTT
423
              |||||||||||||||||||||||||||||||||||||||||||||||||    ||| |||
Sbjct: 212695 TGGAAGTTAATTAATTCGCTGAACATTTTATGTGATGATTGATTGATTGATTGTACAGTT
212754


Query: 424    TGTTTTTCTTAATATCTATTTCGATGACTTCTATATGATATTGCACTAACAAGAAGATAT
483
              ||||||||||||||||||||||||||||||||||||||||||||||||||||||||||||
Sbjct: 212755 TGTTTTTCTTAATATCTATTTCGATGACTTCTATATGATATTGCACTAACAAGAAGATAT
212814


Query: 484    TATAATGCAATTGATACAAGACAAGGAGTTATTTGCTTCTCTTTTATATGATTCTGACAA
543
              ||||||||||||||||||||||||||||||||||||||||||||||||||||||||||||
Sbjct: 212815 TATAATGCAATTGATACAAGACAAGGAGTTATTTGCTTCTCTTTTATATGATTCTGACAA
212874


Query: 544    TCCATATTGCGTTGGTAGTCTTTTTTGCTGGAACGGTTCAGCGGAAAAGACGCATCGCTC
603
              ||||||||||||||||||||||||||||||||||||||||||||||||||||||||||||
Sbjct: 212875 TCCATATTGCGTTGGTAGTCTTTTTTGCTGGAACGGTTCAGCGGAAAAGACGCATCGCTC
212934


Query: 604    TTTTTGCTTCTAGAAGAAATGCCAGCAAAAGAATCTCTTGACAGTGACTGACAGCAAAAA
663
              ||||||||||||||||||||||||||||||||||||||||||||||||||||||||||||
Sbjct: 212935 TTTTTGCTTCTAGAAGAAATGCCAGCAAAAGAATCTCTTGACAGTGACTGACAGCAAAAA
212994
```

F1

```
Query: 292    TGTCTT 297
              ||||||
Sbjct: 212995 TGTCTT 213000


Query: 298    TTTCTAACTAGTAACAAGGCTAAGATATCAGCCTGAAATAAAGGGTGGTGAAGTAATAAT
357
              ||||||||||||||||||||||||||||||||||||||||||||||||||||||||||||
Sbjct: 213001 TTTCTAACTAGTAACAAGGCTAAGATATCAGCCTGAAATAAAGGGTGGTGAAGTAATAAT
213060


Query: 358    TAAATCATCCGTATAAACCTATACACATATATGAGGAAAAATAATACAAAAGTGTTTTAA
417
              ||||||||||||||||||||||||||||||||||||||||||||||||||||||||||||
Sbjct: 213061 TAAATCATCCGTATAAACCTATACACATATATGAGGAAAAATAATACAAAAGTGTTTTAA
213120


Query: 418    ATACAGATACATACATGAACATATGCACGTATAGCGTCCAAATGTCGGTAATGGGATCGG
477
              ||||||||||||||||||||||||||||||||||| ||||||||||||||||||||||||
Sbjct: 213121 ATACAGATACATACATGAACATATGCACGTATAGCGCCCAAATGTCGGTAATGGGATCGG
213180


Query: 478    CTTACTAATTATAAAATGCATCATAGAAATCGTTGAAGTTTGCCGTAGTAATACCCAGAT
537
              ||||||||||||||||||||||||||||||||||||||||||||||||||||||||||||
Sbjct: 213181 CTTACTAATTATAAAATGCATCATAGAAATCGTTGAAGTTTGCCGTAGTAATACCCAGAT
213240


Query: 538    TATCAGATTCCAAATCCTTGTCAATAATTATACTCCTTTGGAAAACTTCTCTTTCCATTA
597
              |||||||||||||||||||||||||||||||||||||||||| |||||||||||||||||
Sbjct: 213241 TATCAGATTCCAAATCCTTGTCAATAATTATACTCCTTTGGACAACTTCTCTTTCCATTA
213300


Query: 598    AAAAATCTGAAATCTCCTTAAATTTTAAATAGATTCTGTTCAGTTCACTAACGGGGAATT
657
              ||||||||||||||||||||||||||||||||||||||||||||||||||||||||||||
Sbjct: 213301 AAAAATCTGAAATCTCCTTAAATTTTAAATAGATTCTGTTCAGTTCACTAACGGGGAATT
213360
```

VIII213200 F

```
Query: 135    TCAAGAGAACATTTTTGTTCTTCGCCGACTGAGTATAATCTGTAACATTATT 186
              ||||||||||||||||||||||||||||||||||||||||||||||||||||
Sbjct: 213361 TCAAGAGAACATTTTTGTTCTTCGCCGACTGACTATAATCTGTAACATTATT 213412


Query: 187    ATTATCAGAGTTTCTCGCAAAATTTTGTTTTTTCTTGCTAAATCTCAGCATATATTTAAT
246
               |||||||||||||||||||||||||||||||||||||||||||||||||||||||||||
Sbjct: 213413 GTTATCAGAGTTTCTCGCAAAATTTTGTTTTTTCTTGCTAAATCTCAGCATATATTTAAT
213472


Query: 247    CAGATTCAAAACCTTGTTGAAACCTTTAATAGATTTGAAATTTCCGTTGCTATTCATTTC
306
              |||||||||||||||||||||||||||||||||||||||| |||||||||||||||||||
Sbjct: 213473 CAGATTCAAAACCTTGTTGAAACCTTTAATAGATTTGAAACTTCCGTTGCTATTCATTTC
213532


Query: 307    ATCTCGTAAAAAGGATACGATAATTTCTATTTTTTTTAAAATTTCCAAAATCTTGTCATG
366
              ||||||||||||||||||||||||||||||||||||||||||||||||||||||||||||
Sbjct: 213533 ATCTCGTAAAAAGGATACGATAATTTCTATTTTTTTTAAAATTTCCAAAATCTTGTCATG
213592


Query: 367    AATCAATAGCAATTGAACATTAATCTCCTCATTTGAAAGATTTTTGTAAAATTCGTCATA
426
              ||||||||||||||||||||||||||||||||||||||||||||||||||||||||||||
Sbjct: 213593 AATCAATAGCAATTGAACATTAATCTCCTCATTTGAAAGATTTTTGTAAAATTCGTCATA
213652


Query: 427    TAATATTACTTCACAACGTTGGAAAATAGCAAATGTGATTGCTATAAAATTCTGTAAGAT
486
              ||||||||||||||||||||||||||||||||||||||||||||||||||||||||||||
Sbjct: 213653 TAATATTACTTCACAACGTTGGAAAATAGCAAATGTGATTGCTATAAAATTCTGTAAGAT
213712


Query: 487    TTCAATAAAATGATTTGCGAATAAAAATTCTTTACCATTAGAATGAAAGCGATTATTGCC
546
              ||||||||||||||||||||||||||||||||||||||||||||||||||||||||||||
Sbjct: 213713 TTCAATAAAATGATTTGCGAATAAAAATTCTTTACCATTAGAATGAAAGCGATTATTGCC
213772


Query: 547    GCTTGAAAATGACTTTATCGACTTTATGGGGAAGATAAAATTAAATGTTACTGAGTAAAA
606
              |||||||||||||||||||||||||||||||||||||||||||||||||| |||||||||
Sbjct: 213773 GCTTGAAAATGACTTTATCGACTTTATGGGGAAGATAAAATTAAATGTTATTGAGTAAAA
213832


Query: 607    AATGTGCATATTAGAAATAATTTTCATCAGATCCTTTGCACATCTTTCAGAGTTCGAGGT
666
              ||||||||||||||||||||||||||||||||||||||||||||||||||||||||||||
```

```
Sbjct: 213833 AATGTGCATATTAGAAATAATTTTCATCAGATCCTTTGCACATCTTTCAGAGTTCGAGGT
213892


Query: 667    CTTATTGTTGTTAGAAGAATGTTGAACTGCCATGGACAAAGAGGATTCGTTTTGAACAAA
726
              ||||||||||||||||||||||||||||||||||||||||||||||||||||||||||||
Sbjct: 213893 CTTATTGTTGTTAGAAGAATGTTGAACTGCCATGGACAAAGAGGATTCGTTTTGAACAAA
213952
```

R1'

```
Query: 595    AAGGAAAAAATTTGTATAAAC 575
              |||||||||||||||||||||
Sbjct: 213953 AAGGAAAAAATTTGTATAAAC 213973


Query: 574    AATGGTATTGATAAA 560
              |||||||||||||||
Sbjct: 213974 AATGGTATTGATAAA 213988
```

3. *CUP1-RSC30* (VIII213688-214288)

VIII213601 F

```
Query: 61     TGA 63
              |||
Sbjct: 213688 TGA 213690


Query: 64     TTGCTATAAAATTCTGTAAGATTTCAATAAAATGATTTGCGAATAAAAATTCTTTACCAT
123
              ||||||||||||||||||||||||||||||||||||||||||||||||||||||||||||
Sbjct: 213691 TTGCTATAAAATTCTGTAAGATTTCAATAAAATGATTTGCGAATAAAAATTCTTTACCAT
213750


Query: 124    TAGAATGAAAGCGATTATTGCCGCTTGAAAATGACTTTATCGACTTTATGGGGAAGATAA
183
              ||||||||||||||||||||||||||||||||||||||||||||||||||||||||||||
Sbjct: 213751 TAGAATGAAAGCGATTATTGCCGCTTGAAAATGACTTTATCGACTTTATGGGGAAGATAA
213810


Query: 184    AATTAAATGTTACTGAGTAAAAAATGTGCATATTAGAAATAATTTTCATCAGATCCTTTG
243
              |||||||||||| |||||||||||||||||||||||||||||||||||||||||||||||
Sbjct: 213811 AATTAAATGTTATTGAGTAAAAAATGTGCATATTAGAAATAATTTTCATCAGATCCTTTG
213870
```

```
Query: 244    CACATCTTTCAGAGTTCGAGGTCTTATTGTTGTTAGAAGAATGTTGAACTGCCATGGACA
303
              ||||||||||||||||||||||||||||||||||||||||||||||||||||||||||||
Sbjct: 213871 CACATCTTTCAGAGTTCGAGGTCTTATTGTTGTTAGAAGAATGTTGAACTGCCATGGACA
213930


Query: 304    AAGAGGATTCGTTTTGAACAAAAAGGAAAAAATTTGTATAAACAATGGTATTGATAAAAT
363
              ||||||||||||||||||||||||||||||||||||||||||||||||||||||||||||
Sbjct: 213931 AAGAGGATTCGTTTTGAACAAAAAGGAAAAAATTTGTATAAACAATGGTATTGATAAAAT
213990


Query: 364    TTAAAGTGTCTTTCCATTCTTTTCTGACTTCGTTGTCATGAAAATATAAGTCTACTGTAT
423
              ||||||||||||||||||||||||||||||||||||||||||||||||||||||||||||
Sbjct: 213991 TTAAAGTGTCTTTCCATTCTTTTCTGACTTCGTTGTCATGAAAATATAAGTCTACTGTAT
214050


Query: 424    TACTCACGCCCATAGTCAAGGTTTCTAACAGACTTTCAATTTTGGTTAAATTTACTGGCA
483
              ||||||||||||||||||||||||||||||||||||||||||||||||||||||||||||
Sbjct: 214051 TACTCACGCCCATAGTCAAGGTTTCTAACAGACTTTCAATTTTGGTTAAATTTACTGGCA
214110
```

VIII216603 R

```
Query: 468    AGTAGAAAGGAACATCTTGCAGAATATTTATCAATT 433
              ||||||||||||||||||||||||||||||||||||
Sbjct: 214111 AGTAGAAAGGAACACCTTGCAGAATATTTATCAATT 214146


Query: 432    TTGCTTGCGTTTCCAGTAATTTTAAATCGTTAGCAATTAAAGGAATGTCGTTCGTATCAA
373
              ||||||||||||||||||||||||||||||||||||||||||||||||||||||||||||
Sbjct: 214147 TTGCTTGCGTTTCCAGTAATTTTAAATCGTTAGCAATTAAAGGAATGTCGTTCGTATCAA
214206


Query: 372    TAGAGGCAGGTATCGGAGATAGGTTTTCAGCAGCGGGTACCATGAAT 326
              |||||||||||||||||||||||||||||||||||||||||||||||
Sbjct: 214207 TAGAGGCAGGTATCGGAGATAGGTTTTCAGCAGCGGGTACCATGAAT 214253


Query: 325    GAAGACTGACCTA 313
              |||||||||||||
Sbjct: 216252 GAAGACTGACCTA 216264


Query: 312    GAAGCGAATGTCTTGAGTAATA 277
              ||||||||||||||||||||||
Sbjct: 216265 GAAGCGAATGTCTTGAGTAATA 216286
```

SNPs between YJM271 and S288c

Sequenced interval	Coordinate(s)	SNP in YJM271	SNP in S288c
CIC1-CUP1 VIII211758-212358			
	211825	A	G
	212243	G	C
	212266-212274	10 T's	9 T's
CUP1 repeat VIII212058-213988			
	212243	G	C
	212266-212274	10 T's	9 T's
	212744-212747	4 bp deletion	ATTG
	212751	G	A
	213157	T	C
	213283	A	C
	213413	A	G
	213513	T	C
	213823	C	T
CUP1-RSC30 VIII213688-214288			
	213823	C	T

Table S7 Sequence analysis of the *CUP1* repeats (Type 4, 1.9 kb) of YJM1307.

In this table, we show genomic sequences of YJM1307 in three regions: 1) the sequences that flank the *CUP1* repeats adjacent to *CIC1*, 2) the sequence of the *CUP1* repeat, and 3) the sequences that flank the *CUP1* tandem array adjacent to *RCS30*. The sequences of YJM1307 (denoted "Query" below) were compared in a BLAST search with sequences of S288c (denoted "Sbject"). SNPs that distinguish YJM1307 and S288c sequences are summarized at the end of the table. The *CUP1* coding sequences are shown in red. The names of the primers used in the sequence analysis are shown in boldface. Additional details about the sequencing are in Supporting Data File S1.

1. ***CIC1-CUP1*** **(VIII211758-212358)**

VIII211528 F

```
Query: 202    AAAGGACAAAATCGAAGAAACCCACGAAGATGACATGGTCAC 244
              ||||||||||||||||||||||||||||||||||||||||||
Sbjct: 211758 AAAGGACAAAATCGAAGAAACCCACGAAGATGACATGGTCAC 211800


Query: 245    CATTGATGGTGTACAAGTTCATTTGTCTACCTTCAACAAGGGTTTGATGGAAATCGCCAA
304
              ||||||||||||||||||||||||||||||||||||||||||||||||||||||||||||
Sbjct: 211801 CATTGATGGTGTACAAGTTCATTTGTCTACCTTCAACAAGGGTTTGATGGAAATCGCCAA
211860


Query: 305    TCCTTCCGAATTGGGTTCAATTTTCTCTAAACAAATTAACAATGCAAAAAAGAGATCTTC
364
              ||||||||||||||||||||||||||||||||||||||||||||||||||||||||||||
Sbjct: 211861 TCCTTCCGAATTGGGTTCAATTTTCTCTAAACAAATTAACAATGCAAAAAAGAGATCTTC
211920


Query: 365    TAGCGAGCTTGAAAAAGAATCTAGCGAGTCAGAAGCTGTCAAGAAGGCTAAAAGTTAATT
424
              ||||||||||||||||||||||||||||||||||||||||||||||||||||||||||||
Sbjct: 211921 TAGCGAGCTTGAAAAAGAATCTAGCGAGTCAGAAGCTGTCAAGAAGGCTAAAAGTTAATT
211980


Query: 425    TGTTTCCTCCTTATCTATCTTTTCTCTCATTTTTTTCTTGTGAAGAAAAAAATTTGAATT
484
              ||||||||||||||||||||||||||||||||||||||||||||||||||||||||||||
Sbjct: 211981 TGTTTCCTCCTTATCTATCTTTTCTCTCATTTTTTTCTTGTGAAGAAAAAAATTTGAATT
212040
```

```
Query: 485    TCATAGAGTGCGGTGCATATGTATATATCTATATATGTTTGAAGTGTATATTAAAAATAA
544
              ||||||||||||||||||||||||||||||||||||||||||||||||||||||||||||
Sbjct: 212041 TCATAGAGTGCGGTGCATATGTATATATCTATATATGTTTGAAGTGTATATTAAAAATAA
212100
```

R1'

```
Query: 506    AGTCATTATTTGAATATTGGTTTCTCGGTCTAAGAGCTTATACGTTTTA 457
              |||||||||||||||||||||||||||||||||||||||||||||||||
Sbjct: 212100 AGTCATTATTTGAATATTGGTTTCTCGGTCTAAGAGCTTATACGTTTTA 212149

Query: 456    GACTGATCTGTTGTACTATCCGCTTCAAATAAATAGATCATTGAAAGTGACGGGGATAAC
397
              ||||||||||||||||||||||||||||||||||||||||||||||||||||||||||||
Sbjct: 212150 GACTGATCTGTTGTACTATCCGCTTCAAATAAATAGATCATTGAAAGTGACGGGGATAAC
212209

Query: 396    AGCATTTTACCTTTAAAAGACGTTCTCATAATAGATTTTAGGATTAATACATATGCTTTT
337
              |||||||||||||||||||||||||||||||||| |||||||||||||||||||||||||
Sbjct: 212210 AGCATTTTACCTTTAAAAGACGTTCTCATAATACATTTTAGGATTAATACATATGCTTTT
212269

Query: 336    TTTTTTATTCGAAATCTGGGGATTCTATACAGAGTTGTAAGTTAGGCAAACTAGAATTTG
277
              ||||| ||||||||||||||||||||||||||||||||||||||||||||||||||||||
Sbjct: 212270 TTTTT-ATTCGAAATCTGGGGATTCTATACAGAGTTGTAAGTTAGGCAAACTAGAATTTG
212328

Query: 276    GTAATAATATTTTATTCTTGGGGCGACATA 247
              ||||||||||||||||||||||||||||||
Sbjct: 212329 GTAATAATATTTTATTCTTGGGGCGACATA 212358
```

2. *CUP1* repeat (VIII212058-213988)

R1'

```
Query: 630    TATGTATATATCTATATATGTTT 537
              |||||||||||||||||||||||
Sbjct: 212058 TATGTATATATCTATATATGTTT 212080
```

```
Query: 536    GAAGTGTATATTAAAAATAAAGTCATTATTTGAATATTGGTTTCTCGGTCTAAGAGCTTA
477
              ||||||||||||||||||||||||||||||||||||||||||||||||||||||||||||
Sbjct: 212081 GAAGTGTATATTAAAAATAAAGTCATTATTTGAATATTGGTTTCTCGGTCTAAGAGCTTA
212140


Query: 476    TACGTTTTAGACTGATCTGTTGTACTATCCGCTTCAAATAAATAGATCATTGAAAGTGAC
417
              ||||||||||||||||||||||||||||||||||||||||||||||||||||||||||||
Sbjct: 212141 TACGTTTTAGACTGATCTGTTGTACTATCCGCTTCAAATAAATAGATCATTGAAAGTGAC
212200


Query: 416    GGGGATAACAGCATTTTACCTTTAAAAGACGTTCTCATAATAGATTTTAGGATTAATACA
357
              |||||||||||||||||||||||||||||||||||||||||| |||||||||||||||||
Sbjct: 212201 GGGGATAACAGCATTTTACCTTTAAAAGACGTTCTCATAATACATTTTAGGATTAATACA
212260


Query: 356    TATGCTTTTTTTTTTATTCGAAATCTGGGGATTCTATACAGAGTTGTAAGTTAGGCAAAC
297
              |||||||||||||| |||||||||||||||||||||||||||||||||||||||||||||
Sbjct: 212261 TATGCTTTTTTTTT-ATTCGAAATCTGGGGATTCTATACAGAGTTGTAAGTTAGGCAAAC
212319


Query: 296    TAGAATTTGGTAATAATATTTTATTCTTGGGGCGACATATGGAGATACTTTATTTCCTTT
237
              ||||||||||||||||||||||||||||||||||||||||||||||||||||||||||||
Sbjct: 212320 TAGAATTTGGTAATAATATTTTATTCTTGGGGCGACATATGGAGATACTTTATTTCCTTT
212379


Query: 236    TCTTAATTATTAACGTATACCTATAAATTAACAAAGTATCTAAACAAAATACATAAGTGT
177
              ||||||||||||||||||||||||||||||||||||||||||||||||||||||||||||
Sbjct: 212380 TCTTAATTATTAACGTATACCTATAAATTAACAAAGTATCTAAACAAAATACATAAGTGT
212439


Query: 176    ACTCAAACTGAGTAGAATCGTCGATTAAACTTCCTTCTCCTTTTAAAAATTAAAAACAGC
117
              ||||||||||||||||||||||||||||||||||||||||||||||||||||||||||||
Sbjct: 212440 ACTCAAACTGAGTAGAATCGTCGATTAAACTTCCTTCTCCTTTTAAAAATTAAAAACAGC
212499
```

VIII212300 F

```
Query: 174    AAATAGTTAGATGA 187
              ||||||||||||||
Sbjct: 212500 AAATAGTTAGATGA 212513
```

```
Query: 188    ATATATTAAAGACTATTCGTTTCATTTCCCAGAGCAGCATGACTTCTTGGTTTCTTCAGA
247
              ||||||||||||||||||||||||||||||||||||||||||||||||||||||||||||
Sbjct: 212514 ATATATTAAAGACTATTCGTTTCATTTCCCAGAGCAGCATGACTTCTTGGTTTCTTCAGA
212573

Query: 248    CTTGTTACCGCAGGGGCATTTGTCGTCGCTGTTACACCCCGTTGGGCAGCTACATGATTT
307
              ||||||||||||||||||||||||||||||||||||||||||||||||||||||||||||
Sbjct: 212574 CTTGTTACCGCAGGGGCATTTGTCGTCGCTGTTACACCCCGTTGGGCAGCTACATGATTT
212633

Query: 308    TTGGCATTGTTCATTATTTTTGCAGCTACCACATTGGCATTGGCACTCATGACCTTCATT
367
              ||||||||||||||||||||||||||||||||||||||||||||||||||||||||||||
Sbjct: 212634 TTGGCATTGTTCATTATTTTTGCAGCTACCACATTGGCATTGGCACTCATGACCTTCATT
212693

Query: 368    TTGGAAGTTAATTAATTCGCTGAACATTTTATGTGATGATTGATTGATTG----TACGGT
423
              ||||||||||||||||||||||||||||||||||||||||||||||||||    ||| ||
Sbjct: 212694 TTGGAAGTTAATTAATTCGCTGAACATTTTATGTGATGATTGATTGATTGATTGTACAGT
212753

Query: 424    TTGTTTTTCTTAATATCTATTTCGATGACTTCTATATGATATTGCACTAACAAGAAGATA
483
              ||||||||||||||||||||||||||||||||||||||||||||||||||||||||||||
Sbjct: 212754 TTGTTTTTCTTAATATCTATTTCGATGACTTCTATATGATATTGCACTAACAAGAAGATA
212813

Query: 484    TTATAATGCAATTGATACAAGACAAGGAGTTATTTGCTTCTCTTTTATATGATTCTGACA
543
              ||||||||||||||||||||||||||||||||||||||||||||||||||||||||||||
Sbjct: 212814 TTATAATGCAATTGATACAAGACAAGGAGTTATTTGCTTCTCTTTTATATGATTCTGACA
212873

Query: 544    ATCCATATTGCGTTGGTAGTCTTTTTTGCTGGAACGGTTCAGCGGAAAAGACGCATCGCT
603
              ||||||||||||||||||||||||||||||||||||||||||||||||||||||||||||
Sbjct: 212874 ATCCATATTGCGTTGGTAGTCTTTTTTGCTGGAACGGTTCAGCGGAAAAGACGCATCGCT
212933

Query: 604    CTTTTTGCTTCTAGAAGAAATGCCAGCAAAAGAATCTCTTGACAGTGACTGACAGCAAAA
663
              ||||||||||||||||||||||||||||||||||||||||||||||||||||||||||||
Sbjct: 212934 CTTTTTGCTTCTAGAAGAAATGCCAGCAAAAGAATCTCTTGACAGTGACTGACAGCAAAA
212993
```

F1

```
Query: 289    ATGTCTTT 296
              ||||||||
Sbjct: 212994 ATGTCTTT 213001


Query: 297    TTCTAACTAGTAACAAGGCTAAGATATCAGCCTGAAATAAAGGGTGGTGAAGTAATAATT
356
              ||||||||||||||||||||||||||||||||||||||||||||||||||||||||||||
Sbjct: 213002 TTCTAACTAGTAACAAGGCTAAGATATCAGCCTGAAATAAAGGGTGGTGAAGTAATAATT
213061


Query: 357    AAATCATCCGTATAAACCTATACACATATATGAGGAAAAATAATACAAAAGTGTTTTAAA
416
              ||||||||||||||||||||||||||||||||||||||||||||||||||||||||||||
Sbjct: 213062 AAATCATCCGTATAAACCTATACACATATATGAGGAAAAATAATACAAAAGTGTTTTAAA
213121


Query: 417    TACAGATACATACATGAACATATGCACGTATAGCGTCCAAATGTCGGTAATGGGATCGGC
476
              ||||||||||||||||||||||||||||||||||| ||||||||||||||||||||||||
Sbjct: 213122 TACAGATACATACATGAACATATGCACGTATAGCGCCCAAATGTCGGTAATGGGATCGGC
213181


Query: 477    TTACTAATTATAAAATGCATCATAGAAATCGTTGAAGTTTGCCGTAGTAATACCCAGATT
536
              ||||||||||||||||||||||||||||||||||||||||||||||||||||||||||||
Sbjct: 213182 TTACTAATTATAAAATGCATCATAGAAATCGTTGAAGTTTGCCGTAGTAATACCCAGATT
213241


Query: 537    ATCAGATTCCAAATCCTTGTCAATAATTATACTCCTTTGGAAAACTTCTCTTTCCATTAA
596
              ||||||||||||||||||||||||||||||||||||||||| ||||||||||||||||||
Sbjct: 213242 ATCAGATTCCAAATCCTTGTCAATAATTATACTCCTTTGGACAACTTCTCTTTCCATTAA
213301


Query: 597    AAAATCTGAAATCTCCTTAAATTTTAAATAGATTCTGTTCAGTTCACTAACGGGGAATTT
656
              ||||||||||||||||||||||||||||||||||||||||||||||||||||||||||||
Sbjct: 213302 AAAATCTGAAATCTCCTTAAATTTTAAATAGATTCTGTTCAGTTCACTAACGGGGAATTT
213361
```

VIII213200 F

```
Query: 134    CAAGAGAACATTTTTGTTCTTCGCCGACTGAGTATAATCTGTAACATTAT 183
              ||||||||||||||||||||||||||||||| ||||||||||||||||||
Sbjct: 213362 CAAGAGAACATTTTTGTTCTTCGCCGACTGACTATAATCTGTAACATTAT 213411
```

```
Query: 184    TATTATCAGAGTTTCTCGCAAAATTTTGTTTTTTCTTGCTAAATCTCAGCATATATTTAA
243
              | ||||||||||||||||||||||||||||||||||||||||||||||||||||||||||
Sbjct: 213412 TGTTATCAGAGTTTCTCGCAAAATTTTGTTTTTTCTTGCTAAATCTCAGCATATATTTAA
213471


Query: 244    TCAGATTCAAAACCTTGTTGAAACCTTTAATAGATTTGAAATTTCCGTTGCTATTCATTT
303
              ||||||||||||||||||||||||||||||||||||||||| ||||||||||||||||||
Sbjct: 213472 TCAGATTCAAAACCTTGTTGAAACCTTTAATAGATTTGAAACTTCCGTTGCTATTCATTT
213531


Query: 304    CATCTCGTAAAAAGGATACGATAATTTCTATTTTTTTTAAAATTTCCAAAATCTTGTCAT
363
              ||||||||||||||||||||||||||||||||||||||||||||||||||||||||||||
Sbjct: 213532 CATCTCGTAAAAAGGATACGATAATTTCTATTTTTTTTAAAATTTCCAAAATCTTGTCAT
213591


Query: 364    GAATCAATAGCAATTGAACATTAATCTCCTCATTTGAAAGATTTTTGTAAAATTCGTCAT
423
              ||||||||||||||||||||||||||||||||||||||||||||||||||||||||||||
Sbjct: 213592 GAATCAATAGCAATTGAACATTAATCTCCTCATTTGAAAGATTTTTGTAAAATTCGTCAT
213651


Query: 424    ATAATATTACTTCACAACGTTGGAAAATAGCAAATGTGATTGCTATAAAATTCTGTAAGA
483
              ||||||||||||||||||||||||||||||||||||||||||||||||||||||||||||
Sbjct: 213652 ATAATATTACTTCACAACGTTGGAAAATAGCAAATGTGATTGCTATAAAATTCTGTAAGA
213711


Query: 484    TTTCAATAAAATGATTTGCGAATAAAAATTCTTTACCATTAGAATGAAAGCGATTATTGC
543
              ||||||||||||||||||||||||||||||||||||||||||||||||||||||||||||
Sbjct: 213712 TTTCAATAAAATGATTTGCGAATAAAAATTCTTTACCATTAGAATGAAAGCGATTATTGC
213771


Query: 544    CGCTTGAAAATGACTTTATCGACTTTATGGGGAAGATAAAATTAAATGTTACTGAGTAAA
603
              ||||||||||||||||||||||||||||||||||||||||||||||||||| ||||||||
Sbjct: 213772 CGCTTGAAAATGACTTTATCGACTTTATGGGGAAGATAAAATTAAATGTTATTGAGTAAA
213831
```

R1’

```
Query: 754    AAATGTGCATATTAGAAATAAT 695
              ||||||||||||||||||||||
Sbjct: 213832 AAATGTGCATATTAGAAATAAT 213853
```

```
Query: 694    TTTCATCAGATCCTTTGCACATCTTTCAGAGTTCGAGGTCTTATTGTTGTTAGAAGAATG
635
              ||||||||||||||||||||||||||||||||||||||||||||||||||||||||||||
Sbjct: 213854 TTTCATCAGATCCTTTGCACATCTTTCAGAGTTCGAGGTCTTATTGTTGTTAGAAGAATG
213913


Query: 634    TTGAACTGCCATGGACAAAGAGGATTCGTTTTGAACAAAAAGGAAAAAATTTGTATAAAC
575
              ||||||||||||||||||||||||||||||||||||||||||||||||||||||||||||
Sbjct: 213914 TTGAACTGCCATGGACAAAGAGGATTCGTTTTGAACAAAAAGGAAAAAATTTGTATAAAC
213973


Query: 574    AATGGTATTGATAAA 560
              |||||||||||||||
Sbjct: 213974 AATGGTATTGATAAA 213988
```

3. *CUP1-RSC30* (VIII213688-214288)

VIII213601 F

```
Query: 63     GATTGCTATA 73
              ||||||||||
Sbjct: 213688 GATTGCTATA 213698


Query: 74     AAATTCTGTAAGATTTCAATAAAATGATTTGCGAATAAAAATTCTTTACCATTAGAATG 133
              |||||||||||||||||||||||||||||||||||||||||||||||||||||||||||
Sbjct: 213699 AAATTCTGTAAGATTTCAATAAAATGATTTGCGAATAAAAATTCTTTACCATTAGAATG
213757


Query: 134    AAAGCGATTATTGCCGCTTGAAAATGACTTTATCGACTTTATGGGGAAGATAAAATTAAA
193
              ||||||||||||||||||||||||||||||||||||||||||||||||||||||||||||
Sbjct: 213758 AAAGCGATTATTGCCGCTTGAAAATGACTTTATCGACTTTATGGGGAAGATAAAATTAAA
213817


Query: 194    TGTTACTGAGTAAAAAATGTGCATATTAGAAATAATTTTCATCAGATCCTTTGCACATCT
253
              ||||| ||||||||||||||||||||||||||||||||||||||||||||||||||||||
Sbjct: 213818 TGTTATTGAGTAAAAAATGTGCATATTAGAAATAATTTTCATCAGATCCTTTGCACATCT
213877


Query: 254    TTCAGAGTTCGAGGTCTTATTGTTGTTAGAAGAATGTTGAACTGCCATGGACAAAGAGGA
313
              ||||||||||||||||||||||||||||||||||||||||||||||||||||||||||||
Sbjct: 213878 TTCAGAGTTCGAGGTCTTATTGTTGTTAGAAGAATGTTGAACTGCCATGGACAAAGAGGA
213937
```

```
Query: 314    TTCGTTTTGAACAAAAAGGAAAAAATTTGTATAAACAATGGTATTGATAAAATTTAAAGT
373
              ||||||||||||||||||||||||||||||||||||||||||||||||||||||||||||
Sbjct: 213938 TTCGTTTTGAACAAAAAGGAAAAAATTTGTATAAACAATGGTATTGATAAAATTTAAAGT
213997


Query: 374    GTCTTTCCATTCTTTTCTGACTTCGTTGTCATGAAAATATAAGTCTACTGTATTACTCAC
433
              ||||||||||||||||||||||||||||||||||||||||||||||||||||||||||||
Sbjct: 213998 GTCTTTCCATTCTTTTCTGACTTCGTTGTCATGAAAATATAAGTCTACTGTATTACTCAC
214057


Query: 434    GCCCATAGTCAAGGTTTCTAACAGACTTTCAATTTTGGTTAAATTTACTGGCAAGTAGAA
493
              ||||||||||||||||||||||||||||||||||||||||||||||||||||||||||||
Sbjct: 214058 GCCCATAGTCAAGGTTTCTAACAGACTTTCAATTTTGGTTAAATTTACTGGCAAGTAGAA
214117


Query: 494    AGGAACATCTTGCAGAATATTTATCAATTTTGCTTGCGTTTCCAGTAATTTTAAATCGTT
553
              ||||||| ||||||||||||||||||||||||||||||||||||||||||||||||||||
Sbjct: 214118 AGGAACACCTTGCAGAATATTTATCAATTTTGCTTGCGTTTCCAGTAATTTTAAATCGTT
214177


Query: 554    AGCAATTAAAGGAATGTCGTTCGTATCAATAGAGGCAGGTATCGGAGATAGGTTTTCAGC
613
              ||||||||||||||||||||||||||||||||||||||||||||||||||||||||||||
Sbjct: 214178 AGCAATTAAAGGAATGTCGTTCGTATCAATAGAGGCAGGTATCGGAGATAGGTTTTCAGC
214237


Query: 614    AGCGGGTACCATGAAT 629
              ||||||||||||||||
Sbjct: 214238 AGCGGGTACCATGAAT 214253
```

VIII216603 R

```
Query: 325    GAAGACTGAC 316
              ||||||||||
Sbjct: 216252 GAAGACTGAC 216261


Query: 315    CTAGAAGCGAATGTCTTGAGTAATA 291
              |||||||||||||||||||||||||
Sbjct: 216262 CTAGAAGCGAATGTCTTGAGTAATA 216286
```

SNPs between YJM1307 and S288c

Sequenced interval	Coordinate(s)	SNP in YJM1307	SNP in S288c
CIC1-CUP1 VIII211758-212358			
	212243	G	C
	212266-212274	10 T's	9 T's
CUP1 repeat VIII212058-213988			
	212243	G	C
	212266-212274	10 T's	9 T's
	212744-212747	4 bp deletion	ATTG
	212751	G	A
	213157	T	C
	213283	A	C
	213393	G	C
	213413	A	G
	213513	T	C
	213823	C	T
CUP1-RSC30 VIII213688-214288			
	213823	C	T
	214125	T	C

Table S8 Sequence analysis of the *CUP1* repeats (Type 5, 1.6 kb) of YJM456.

In this table, we show genomic sequences of YJM456 in three regions: 1) the sequences that flank the *CUP1* repeats adjacent to *CIC1*, 2) the sequence of the *CUP1* repeat, and 3) the sequences that flank the *CUP1* tandem array adjacent to *RCS30*. The sequences of YJM456 (denoted "Query" below) were compared in a BLAST search with sequences of S288c (denoted "Sbject"). SNPs that distinguish YJM456 and S288c sequences are summarized at the end of the table. The *CUP1* coding sequences are shown in red. The names of the primers used in the sequence analysis are shown in boldface. Additional details about the sequencing are in Supporting Data File S1.

1. *CIC1-CUP1* (VIII211275-211875)

VIII211185 F

```
Query: 62     AAT 64
              |||
Sbjct: 211275 AAT 211277


Query: 65     TAGATTCAGAAGGAATCAAGGTTGATGAAATCATTTGCGGGAAAGACTTAAAGACCGTTT
124
              ||||||||||||||||||||||||||||||||||||||||||||||||||||||||||||
Sbjct: 211278 TAGATTCAGAAGGAATCAAGGTTGATGAAATCATTTGCGGGAAAGACTTAAAGACCGTTT
211337


Query: 125    ACAAGGCATATGAGACTAGAAACGCTTTTATATCTCAGTTTTCTTTGATTTTGGCTGACG
184
              |||||||||||||| |||||||||||||||||||||||||||||||||||||||||||||
Sbjct: 211338 ACAAGGCATATGAGGCTAGAAACGCTTTTATATCTCAGTTTTCTTTGATTTTGGCTGACG
211397


Query: 185    ACAGTATAGTTACATCTTTGCCAAAACTTATGGGAGGCAAAGCCTACAACAAAGTAGAAA
244
              ||||||||||||||||||||||||||||||||||||||||||||||||||||||||||||
Sbjct: 211398 ACAGTATAGTTACATCTTTGCCAAAACTTATGGGAGGCAAAGCCTACAACAAAGTAGAAA
211457


Query: 245    CTACCCCTATATCAATTAGAACACATGCAAATAAGGAATTTTCCTTGACCACTTTGACGA
304
```

```
              |||| |||||||||||||||||||||||||||||||||||||||||||||||||||||||
Sbjct: 211458 CTACTCCTATATCAATTAGAACACATGCAAATAAGGAATTTTCCTTGACCACTTTGACGA
211517

Query: 305    ACAATATCAAAAAGGTATACATGAATCAGTTGCCCGTTAAACTTCCAAGAGGTACCACGT
364
              |||||||||||||||| |||||||||||||||||||||||||||||||||||||||||||
Sbjct: 211518 ACAATATCAAAAAGGTTTACATGAATCAGTTGCCCGTTAAACTTCCAAGAGGTACCACGT
211577

Query: 365    TGAATGTCCATTTGGGTAATTTAGAATGGTTAAGGCCAGAAGAGTTTGTAGATAACGTTG
424
              ||||||||||||||||||||||||||||||||||||||||||||||||||||||||||||
Sbjct: 211578 TGAATGTCCATTTGGGTAATTTAGAATGGTTAAGGCCAGAAGAGTTTGTAGATAACGTTG
211637


Query: 425    AATTAATTTCTGAACAGGTAATCAAAGCATACCAAATCAGATCCATTTTTATCAAAACCA
484
              ||||||||||||||||| ||||||||||||||||||||||||||||||||||||| ||||
Sbjct: 211638 AATTAATTTCTGAACAGTTAATCAAAGCATACCAAATCAGATCCATTTTTATCAAGACCA
211697

Query: 485    ATAAGTCACCCGTATTGCCATTATACTATAACCAGGACGTTCTTGATGAACTTGAAGCTA
544
              ||| ||| ||||||||||||||||||||||||||||||||||||||||||||||||||||
Sbjct: 211698 ATAGGTCGCCCGTATTGCCATTATACTATAACCAGGACGTTCTTGATGAACTTGAAGCTA
211757

Query: 545    AAAAGGACAAAATCGAAGAAACCCACGAAGATGACATGGTCACCATTGATGGTGTACAAG
604
              ||||||||||||||||||||||||||||||||||||||||||||||||||||||||||||
Sbjct: 211758 AAAAGGACAAAATCGAAGAAACCCACGAAGATGACATGGTCACCATTGATGGTGTACAAG
211817

Query: 605    TTCATTTATCTACCTTCAACAAGGGTTTGATGGAAATCGCCAATCCTTCCGAATTGGG 662
              ||||||| ||||||||||||||||||||||||||||||||||||||||||||||||||
Sbjct: 211818 TTCATTTGTCTACCTTCAACAAGGGTTTGATGGAAATCGCCAATCCTTCCGAATTGGG
211875
```

2. *CUP1* repeat (VIII211575-213213)

VIII212063 R

```
Query: 455    CGTTGAATGTCCATTTGGGTAAT 433
              |||||||||||||||||||||||
Sbjct: 211575 CGTTGAATGTCCATTTGGGTAAT 211597
```

```
Query: 432    TTAGAATGGTTAAGGCCAGAAGAGTTTGTAGATAACGTTGAATTAATTTCTGAACAGGTA
373
              ||||||||||||||||||||||||||||||||||||||||||||||||||||||||| ||
Sbjct: 211598 TTAGAATGGTTAAGGCCAGAAGAGTTTGTAGATAACGTTGAATTAATTTCTGAACAGTTA
211657


Query: 372    ATCAAAGCATACCAAATCAGATCCATTTTTATCAAAACCAATAAGTCACCCGTATTGCCA
313
              ||||||||||||||||||||||||||||||||||| ||||||| ||| ||||||||||||
Sbjct: 211658 ATCAAAGCATACCAAATCAGATCCATTTTTATCAAGACCAATAGGTCGCCCGTATTGCCA
211717


Query: 312    TTATACTATAACCAGGACGTTCTTGATGAACTTGAAGCTAAAAAGGACAAAATCGAAGAA
253
              ||||||||||||||||||||||||||||||||||||||||||||||||||||||||||||
Sbjct: 211718 TTATACTATAACCAGGACGTTCTTGATGAACTTGAAGCTAAAAAGGACAAAATCGAAGAA
211777


Query: 252    ACCCACGAAGATGACATGGTCACCATTGATGGTGTACAAGTTCATTTATCTACCTTCAAC
193
              ||||||||||||||||||||||||||||||||||||||||||||||| ||||||||||||
Sbjct: 211778 ACCCACGAAGATGACATGGTCACCATTGATGGTGTACAAGTTCATTTGTCTACCTTCAAC
211837


Query: 192    AAGGGTTTGATGGAAATCGCCAATCCTTCCGAATTGGGTTCAATTTTCTCTAAACAAATT
133
              ||||||||||||||||||||||||||||||||||||||||||||||||||||||||||||
Sbjct: 211838 AAGGGTTTGATGGAAATCGCCAATCCTTCCGAATTGGGTTCAATTTTCTCTAAACAAATT
211897
```

R1

```
Query: 450    AACAATGCAAAAAAGAGATCTTCTAGCGAGCTTGAAAAAGAATCTAGC 403
              ||||||||||||||||||||||||||||||||||||||||||||||||
Sbjct: 211898 AACAATGCAAAAAAGAGATCTTCTAGCGAGCTTGAAAAAGAATCTAGC 211945


Query: 402    GAGTCAGAAGCTGTCAAGAAGGCTAAAAGTTAATTTGTTTCCTCCTTATCTATCTTTTCT
343
              ||||||||||||||||||||||||||||||||||||||||||||||||||||||||||||
Sbjct: 211946 GAGTCAGAAGCTGTCAAGAAGGCTAAAAGTTAATTTGTTTCCTCCTTATCTATCTTTTCT
212005


Query: 342    CTCATTTTTTTCTTGTGAAGAAAAAAATTTGAATTTCATAGAGTGCGGTGCATATGTATA
283
              ||||||||||||||||||||||||||||||||||||||||||||||||||||||||||||
Sbjct: 212006 CTCATTTTTTTCTTGTGAAGAAAAAAATTTGAATTTCATAGAGTGCGGTGCATATGTATA
212065
```

```
Query: 282    TATCTATATATGTTTGAAGTGTATATTAAAAATAAAGTCATTATTTGAATATTGGTTTCT
223
              ||||||||||||||||||||||||||||||||||||||||||||||||||||||||||||
Sbjct: 212066 TATCTATATATGTTTGAAGTGTATATTAAAAATAAAGTCATTATTTGAATATTGGTTTCT
212125


Query: 222    CGGTCTAAGAGCTTATACGTTTTAGACTGATCTGTTGTACTATCCGCTTCAAATAAATAG
163
              ||||||||||||||||||||||||||||||||||||||||||||||||||||||||||||
Sbjct: 212126 CGGTCTAAGAGCTTATACGTTTTAGACTGATCTGTTGTACTATCCGCTTCAAATAAATAG
212185


Query: 162    ATCATTGAAAGTGACGGGGATAACAGCATTTTACCTTTAAAAGACGTTCTCATAATAGAT
103
              ||||||||||||||||||||||||||||||||||||||||||||||||||||||||| ||
Sbjct: 212186 ATCATTGAAAGTGACGGGGATAACAGCATTTTACCTTTAAAAGACGTTCTCATAATACAT
212245
```

R1’

```
Query: 363    TTTAGGATTAATACAT 348
              ||||||||||||||||
Sbjct: 212246 TTTAGGATTAATACAT 212261


Query: 347    ATGCTTTTTTTTTTATTCGAAATCTGGGGATTCTATACAGAGTTGTAAGTTAGGCAAACT
288
              ||||||||||||| ||||||||||||||||||||||||||||||||||||||||||||||
Sbjct: 212262 ATGCTTTTTTTTT-ATTCGAAATCTGGGGATTCTATACAGAGTTGTAAGTTAGGCAAACT
212320


Query: 287    AGAATTTGGTAATAATATTTTATTCTTGGGGCGACATATGGAGATACTTTATTTCCTTTT
228
              ||||||||||||||||||||||||||||||||||||||||||||||||||||||||||||
Sbjct: 212321 AGAATTTGGTAATAATATTTTATTCTTGGGGCGACATATGGAGATACTTTATTTCCTTTT
212380


Query: 227    CTTAATTATTAACGTATACCTATAAATTAACAAAGTATCTAAACAAAATACATAAGTGTA
168
              ||||||||||||||||||||||||||||||||||||||||||||||||||||||||||||
Sbjct: 212381 CTTAATTATTAACGTATACCTATAAATTAACAAAGTATCTAAACAAAATACATAAGTGTA
212440
```

VIII212300 F

```
Query: 115    CTCAAACTGAGTA 127
              |||||||||||||
```

```
Sbjct: 212441 CTCAAACTGAGTA 212453


Query: 128    GAATCGTCGATTAAACTTCCTTCTCCTTTTAAAAATTAAAAACAGCAAATAGTTAGATGA
187
              ||||||||||||||||||||||||||||||||||||||||||||||||||||||||||||
Sbjct: 212454 GAATCGTCGATTAAACTTCCTTCTCCTTTTAAAAATTAAAAACAGCAAATAGTTAGATGA
212513



Query: 188    ATATATTAAAGACTATTCGTTTTATTTCCCAGAGCAGCATGACTTCTTGGTTTCTTCAGA
247
              |||||||||||||||||||||| |||||||||||||||||||||||||||||||||||||
Sbjct: 212514 ATATATTAAAGACTATTCGTTTCATTTCCCAGAGCAGCATGACTTCTTGGTTTCTTCAGA
212573


Query: 248    CTTGTTACCGCAGGGGCATTTGTCGTCGCTGTTACACCCCGTTGGGCAGCTACATGATTT
307
              ||||||||||||||||||||||||||||||||||||||||||||||||||||||||||||
Sbjct: 212574 CTTGTTACCGCAGGGGCATTTGTCGTCGCTGTTACACCCCGTTGGGCAGCTACATGATTT
212633


Query: 308    TTGGCATTGTTCATTATTTTTGCAGCTACCACATTGGCATTGGCACTCATGACCTTCATT
367
              ||||||||||||||||||||||||||||||||||||||||||||||||||||||||||||
Sbjct: 212634 TTGGCATTGTTCATTATTTTTGCAGCTACCACATTGGCATTGGCACTCATGACCTTCATT
212693


Query: 368    TTGGAAGTTAATTAATTCGCTGAACATTTTATGTGATGATTGATTGATTG----TACGGT
423
              ||||||||||||||||||||||||||||||||||||||||||||||||||    ||| ||
Sbjct: 212694 TTGGAAGTTAATTAATTCGCTGAACATTTTATGTGATGATTGATTGATTGATTGTACAGT
212753


Query: 424    TTGTTTTTCTTAATATCTATTTCGATGACTTCTATATGATATTGCACTAACAAGAAGATA
483
              ||||||||||||||||||||||||||||||||||||||||||||||||||||||||||||
Sbjct: 212754 TTGTTTTTCTTAATATCTATTTCGATGACTTCTATATGATATTGCACTAACAAGAAGATA
212813


Query: 484    TTATAATGCAATTGATACAAGACAAGGAGTTATTTGCTTCTCTTTTATATGATTCTGACA
543
              ||||||||||||||||||||||||||||||||||||||||||||||||||||||||||||
Sbjct: 212814 TTATAATGCAATTGATACAAGACAAGGAGTTATTTGCTTCTCTTTTATATGATTCTGACA
212873


Query: 544    ATCCATATTGCGTTGGTAGTCTTTTTTGCTGGAACGGTTCAGCGGAAAAGACGCATCGCT
603
              ||||||||||||||||||||||||||||||||||||||||||||||||||||||||||||
```

```
Sbjct: 212874 ATCCATATTGCGTTGGTAGTCTTTTTTGCTGGAACGGTTCAGCGGAAAAGACGCATCGCT
212933
```

```
Query: 604    CTTTTTGCTTCTAGAAGAAATGCCAGCAAAAGAATCTCTTGACAGTGACTGACAGCAAAA
663
              ||||||||||||||||||||||||||||||||||||||||||||||||||||||||||||
Sbjct: 212934 CTTTTTGCTTCTAGAAGAAATGCCAGCAAAAGAATCTCTTGACAGTGACTGACAGCAAAA
212993
```

F1

```
Query: 291    ATGTCTTTTT 300
              ||||||||||
Sbjct: 212994 ATGTCTTTTT 213003
```

```
Query: 301    CTAACTAGTAACAAGGCTAAGATATCAGCCTGAAATAAAGGGTGGTGAAGTAATAATTAA
360
              ||||||||||||||||||||||||||||||||||||||||||||||||||||||||||||
Sbjct: 213004 CTAACTAGTAACAAGGCTAAGATATCAGCCTGAAATAAAGGGTGGTGAAGTAATAATTAA
213063
```

```
Query: 361    ATCATCCGTATAAACCTATACACATATATGAGGAAAAATAATACAAAAGTGTTTTAAATA
420
              ||||||||||||||||||||||||||||||||||||||||||||||||||||||||||||
Sbjct: 213064 ATCATCCGTATAAACCTATACACATATATGAGGAAAAATAATACAAAAGTGTTTTAAATA
213123
```

```
Query: 421    CAGATACATACATGAACATATGCACGTATAGCGTCCAAATGTCGGTAATGGGATCGGCTT
480
              |||||||||||||||||||||||||||||||||| |||||||||||||||||||||||||
Sbjct: 213124 CAGATACATACATGAACATATGCACGTATAGCGCCCAAATGTCGGTAATGGGATCGGCTT
213183
```

```
Query: 481    ACTAATTATAAAATGCATCATAGAAATCGT 510
              ||||||||||||||||||||||||||||||
Sbjct: 213184 ACTAATTATAAAATGCATCATAGAAATCGT 213213
```

3. *CUP1-RSC30* (VIII212913-213513)

F1

```
Query: 214    CAGCGGAAAAGACGCATCGCTCTTTTTGCTTCTAGAAGAA 253
              ||||||||||||||||||||||||||||||||||||||||
Sbjct: 212913 CAGCGGAAAAGACGCATCGCTCTTTTTGCTTCTAGAAGAA 212952
```

```
Query: 254    ATGCCAGCAAAAGAATCTCTTGACAGTGACTGACAGCAAAAATGTCTTTTTCTAACTAGT
313
              ||||||||||||||||||||||||||||||||||||||||||||||||||||||||||||
Sbjct: 212953 ATGCCAGCAAAAGAATCTCTTGACAGTGACTGACAGCAAAAATGTCTTTTTCTAACTAGT
213012


Query: 314    AACAAGGCTAAGATATCAGCCTGAAATAAAGGGTGGTGAAGTAATAATTAAATCATCCGT
373
              ||||||||||||||||||||||||||||||||||||||||||||||||||||||||||||
Sbjct: 213013 AACAAGGCTAAGATATCAGCCTGAAATAAAGGGTGGTGAAGTAATAATTAAATCATCCGT
213072


Query: 374    ATAAACCTATACACATATATGAGGAAAAATAATACAAAAGTGTTTTAAATACAGATACAT
433
              ||||||||||||||||||||||||||||||||||||||||||||||||||||||||||||
Sbjct: 213073 ATAAACCTATACACATATATGAGGAAAAATAATACAAAAGTGTTTTAAATACAGATACAT
213132


Query: 434    ACATGAACATATGCACGTATAGCGTCCAAATGTCGGTAATGGGATCGGCTTACTAATTAT
493
              ||||||||||||||||||||||||| ||||||||||||||||||||||||||||||||||
Sbjct: 213133 ACATGAACATATGCACGTATAGCGCCCAAATGTCGGTAATGGGATCGGCTTACTAATTAT
213192


Query: 494    AAAATGCATCATAGAAATCGTTGAAGTTTGCCGTAGTAATACCCAGATTATCAGATTCCA
553
              ||||||||||||||||||||||||||||||||||||||||||||||||||||||||||||
Sbjct: 213193 AAAATGCATCATAGAAATCGTTGAAGTTTGCCGTAGTAATACCCAGATTATCAGATTCCA
213252


Query: 554    AATCCTTGTCAATAATTATACTCCTTTGGAAAACTTCTCTTTCCATTAAAAAATCTGAAA
613
              ||||||||||||||||||||||||||||||| ||||||||||||||||||||||||||||
Sbjct: 213253 AATCCTTGTCAATAATTATACTCCTTTGGACAACTTCTCTTTCCATTAAAAAATCTGAAA
213312


Query: 614    TCTCCTTAAATTTTAAATAGATTCTGTTCAGTTCACTAACGGGGAATTTCAAGAGAACAT
673
              ||||||||||||||||||||||||||||||||||||||||||||||||||||||||||||
Sbjct: 213313 TCTCCTTAAATTTTAAATAGATTCTGTTCAGTTCACTAACGGGGAATTTCAAGAGAACAT
213372
```

VIII213200 F

```
Query: 146    TTTTGTTCTTCGCCGACTGACTATAATCTGTAACATTA 183
              ||||||||||||||||||||||||||||||||||||||
Sbjct: 213373 TTTTGTTCTTCGCCGACTGACTATAATCTGTAACATTA 213410
```

```
Query: 184    TTGTTATCAGAGTTTCTCGCAAAATTTTGTTTTTTCTTGCTAAATCTCAGCATATATTTA
243
              ||||||||||||||||||||||||||||||||||||||||||||||||||||||||||||
Sbjct: 213411 TTGTTATCAGAGTTTCTCGCAAAATTTTGTTTTTTCTTGCTAAATCTCAGCATATATTTA
213470


Query: 244    ATCAGATTCAAAACCTTGTTGAAACCTTTAATAGATTTGAAAT 286
              ||||||||||||||||||||||||||||||||||||||||||
Sbjct: 213471 ATCAGATTCAAAACCTTGTTGAAACCTTTAATAGATTTGAAAC 213513
```

SNPs between YJM456 and S288c

Sequenced interval	Coordinate(s)	SNP in YJM456	SNP in S288c
CIC1-CUP1 VIII211275-211875			
	211352	A	G
	211462	C	T
	211534	A	T
	211655	G	T
	211693	A	G
	211701	A	G
	211705	A	G
	211825	A	G
CUP1 repeat VIII211575-213213			
	211655	G	T
	211693	A	G
	211701	A	G
	211705	A	G
	211825	A	G
	212243	G	C
	212266-212274	10 poly(T)	9 poly(T)
	212536	T	C
	212744-212747	4 bp deletion	ATTG
	212751	G	A
	213157	T	C
CUP1-RSC30 VIII212913-213513			
	213157	T	C
	213283	A	C

Table S9 Sequence analysis of the *CUP1* repeats (Type 5, 1.6 kb) of YJM969.

In this table, we show genomic sequences of YJM969 in three regions: 1) the sequences that flank the *CUP1* repeats adjacent to *CIC1*, 2) the sequence of the *CUP1* repeat, and 3) the sequences that flank the *CUP1* tandem array adjacent to *RCS30*. The sequences of YJM969 (denoted “Query” below) were compared in a BLAST search with sequences of S288c (denoted “Sbject”). SNPs that distinguish YJM969 and S288c sequences are summarized at the end of the table. The *CUP1* coding sequences are shown in red. The names of the primers used in the sequence analysis are shown in boldface. Additional details about the sequencing are in Supporting Data File S1.

1. ***<u>CIC1-CUP1</u>* <u>(VIII211275-211875)</u>**

VIII211185 F

```
Query: 1      AATTAGATTCAGAAGGAATCAAGGTTGATGAAATCATTTGCGGGAAAGACTTAAAGACCG 60
              ||||||||||||||||||||||||||||||||||||||||||||||||||||||||||||
Sbjct: 211275 AATTAGATTCAGAAGGAATCAAGGTTGATGAAATCATTTGCGGGAAAGACTTAAAGACCG
211334


Query: 61     TTTACAAGGCATATGAGGCTAGAAACGCTTTTATATCTCAGTTTTCTTTGATTTTGGCTG
120
              ||||||||||||||||||||||||||||||||||||||||||||||||||||||||||||
Sbjct: 211335 TTTACAAGGCATATGAGGCTAGAAACGCTTTTATATCTCAGTTTTCTTTGATTTTGGCTG
211394


Query: 121    ACGACAGTATAGTTACATCTTTGCCAAAACTTATGGGAGGCAAAGCCTACAACAAAGTAG
180
              ||||||||||||||||||||||||||||||||||||||||||||||||||||||||||||
Sbjct: 211395 ACGACAGTATAGTTACATCTTTGCCAAAACTTATGGGAGGCAAAGCCTACAACAAAGTAG
211454


Query: 181    AAACTACCCCTATATCAATTAGAACACATGCAAATAAGGAATTTTCCTTGACCACTTTGA
240
              ||||||| ||||||||||||||||||||||||||||||||||||||||||||||||||||
Sbjct: 211455 AAACTACTCCTATATCAATTAGAACACATGCAAATAAGGAATTTTCCTTGACCACTTTGA
211514


Query: 241    CGAACAATATCAAAAAGGTATACATGAATCAGTTGGCCGTTAAACTTCCAAGAGGTACCA
300
              ||||||||||||||||||| ||||||||||||||| ||||||||||||||||||||||||
```

```
Sbjct: 211515 CGAACAATATCAAAAAGGTTTACATGAATCAGTTGCCCGTTAAACTTCCAAGAGGTACCA
211574


Query: 301    CGTTGAATGTCCATTTGGGTAATTTAGAATGGTTAAGGCCAGAAGAGTTTGTAGATAACG
360
              ||||||||||||||||||||||||||||||||||||||||||||||||||||||||||||
Sbjct: 211575 CGTTGAATGTCCATTTGGGTAATTTAGAATGGTTAAGGCCAGAAGAGTTTGTAGATAACG
211634


Query: 361    TTGAATTAATTTCTGAACAGGTAATCAAAGCATACCAAATCAGATCCATTTTTATCAAAA
420
              |||||||||||||||||||| |||||||||||||||||||||||||||||||||||||| |
Sbjct: 211635 TTGAATTAATTTCTGAACAGTTAATCAAAGCATACCAAATCAGATCCATTTTTATCAAGA
211694


Query: 421    CCAATAAGTCGCCCGTATTGCCATTATACTATAACCAGGACGTTCTTGATGAACTTGAAG
480
              |||||| |||||||||||||||||||||||||||||||||||||||||||||||||||||
Sbjct: 211695 CCAATAGGTCGCCCGTATTGCCATTATACTATAACCAGGACGTTCTTGATGAACTTGAAG
211754


Query: 481    CTAAAAAGGACAAAATCGAAGAAACCCACGAAGATGACATGGTCACCATTGATGGTGTAC
540
              ||||||||||||||||||||||||||||||||||||||||||||||||||||||||||||
Sbjct: 211755 CTAAAAAGGACAAAATCGAAGAAACCCACGAAGATGACATGGTCACCATTGATGGTGTAC
211814


Query: 541    AAGTTCATTTATCTACCTTCAACAAGGGTTTGATGGAAATCGCCAATCCTTCCGAATTGG
600
              |||||||||| |||||||||||||||||||||||||||||||||||||||||||||||||
Sbjct: 211815 AAGTTCATTTGTCTACCTTCAACAAGGGTTTGATGGAAATCGCCAATCCTTCCGAATTGG
211874


Query: 601    G
              |
Sbjct: 211875 G
```

2. *CUP1* Repeat (VIII211575-213213)

F1

```
Query: 514    CGTTGAATGTCCATTTGGGTAAT 536
              |||||||||||||||||||||||
Sbjct: 211575 CGTTGAATGTCCATTTGGGTAAT 211597


Query: 537    TTAGAATGGTTAAGGCCAGAAGAGTTTGTAGATAACGTTGAATTAATTTCTGAACAGGTA
596
```

```
                |||||||||||||||||||||||||||||||||||||||||||||||||||||||||| ||
Sbjct: 211598 TTAGAATGGTTAAGGCCAGAAGAGTTTGTAGATAACGTTGAATTAATTTCTGAACAGTTA
211657


Query: 597    ATCAAAGCATACCAAATCAGATCCATTTTTATCAAAACCAATAAGTCGCCCGTATTGCCA
656
              ||||||||||||||||||||||||||||||||||| ||||||| ||||||||||||||||
Sbjct: 211658 ATCAAAGCATACCAAATCAGATCCATTTTTATCAAGACCAATAGGTCGCCCGTATTGCCA
211717


Query: 657    TTATACTATAACCAGGACGTTCTTGATGAACTTGAAGCTAAAAAGGACAAAATCGAAGAA
716
              ||||||||||||||||||||||||||||||||||||||||||||||||||||||||||||
Sbjct: 211718 TTATACTATAACCAGGACGTTCTTGATGAACTTGAAGCTAAAAAGGACAAAATCGAAGAA
211777


Query: 717    ACCCA 721
              |||||
Sbjct: 211778 ACCCA 211782
```

VIII212063 R

```
Query: 249    CGAAGATGACATGGTCACCATTGATGGTGTACAAGTTCATTTATCTACCTTCAACAAGGG
190
              |||||||||||||||||||||||||||||||||||||||||| |||||||||||||||||
Sbjct: 211783 CGAAGATGACATGGTCACCATTGATGGTGTACAAGTTCATTTGTCTACCTTCAACAAGGG
211842


Query: 189    TTTGATGGAAATCGCCAATCCTTCCGAATTGGGTTCAATTTTCTCTAAACAAATTAACAA
130
              ||||||||||||||||||||||||||||||||||||||||||||||||||||||||||||
Sbjct: 211843 TTTGATGGAAATCGCCAATCCTTCCGAATTGGGTTCAATTTTCTCTAAACAAATTAACAA
211902


Query: 129    TGCAAAAAAGAGATCTTCTAGCGAGCTTGAAAAAGAATCTAGCGAGTCAGAAGCTGTCAA 70
              ||||||||||||||||||||||||||||||||||||||||||||||||||||||||||||
Sbjct: 211903 TGCAAAAAAGAGATCTTCTAGCGAGCTTGAAAAAGAATCTAGCGAGTCAGAAGCTGTCAA
211962
```

R1’

```
Query: 665    GAAGGCTAAAAGTTAATTTGTTTCCTCCTTATCTATCTTTTCTCTCAT 594
              ||||||||||||||||||||||||||||||||||||||||||||||||
Sbjct: 211963 GAAGGCTAAAAGTTAATTTGTTTCCTCCTTATCTATCTTTTCTCTCAT 212010


Query: 593    TTTTTTCTTGTGAAGAAAAAAATTTGAATTTCATAGAGTGCGGTGCATATGTATATATCT
534
              ||||||||||||||||||||||||||||||||||||||||||||||||||||||||||||
```

```
Sbjct: 212011 TTTTTTCTTGTGAAGAAAAAAATTTGAATTTCATAGAGTGCGGTGCATATGTATATATCT
212070


Query: 533    ATATATGTTTGAAGTGTATATTAAAAATAAAGTCATTATTTGAATATTGGTTTCTCGGTC
474
              ||||||||||||||||||||||||||||||||||||||||||||||||||||||||||||
Sbjct: 212071 ATATATGTTTGAAGTGTATATTAAAAATAAAGTCATTATTTGAATATTGGTTTCTCGGTC
212130


Query: 473    TAAGAGCTTATACGTTTTAGACTGATCTGTTGTACTATCCGCTTCAAATAAATAGATCAT
414
              ||||||||||||||||||||||||||||||||||||||||||||||||||||||||||||
Sbjct: 212131 TAAGAGCTTATACGTTTTAGACTGATCTGTTGTACTATCCGCTTCAAATAAATAGATCAT
212190


Query: 413    TGAAAGTGACGGGGATAACAGCATTTTACCTTTAAAAGACGTTCTCATAATAGATTTTAG
354
              |||||||||||||||||||||||||||||||||||||||||||||||||||| |||||||
Sbjct: 212191 TGAAAGTGACGGGGATAACAGCATTTTACCTTTAAAAGACGTTCTCATAATACATTTTAG
212250


Query: 353    GATTAATACATATGCTTTTTTTTTTATTCGAAATCTGGGGATTCTATACAGAGTTGTAAG
294
              |||||||||||||||||||||||| |||||||||||||||||||||||||||||||||||
Sbjct: 212251 GATTAATACATATGCTTTTTTTTT-ATTCGAAATCTGGGGATTCTATACAGAGTTGTAAG
212309


Query: 293    TTAGGCAAACTAGAATTTGGTAATAATATTTTATTCTTGGGGCGACATATGGAGATACTT
234
              ||||||||||||||||||||||||||||||||||||||||||||||||||||||||||||
Sbjct: 212310 TTAGGCAAACTAGAATTTGGTAATAATATTTTATTCTTGGGGCGACATATGGAGATACTT
212369
```

VIII212300 F

```
Query: 42     TATTTCCTTTTCTTAATTATTAAC 65
              ||||||||||||||||||||||||
Sbjct: 212370 TATTTCCTTTTCTTAATTATTAAC 212393


Query: 66     GTATACCTATAAATTAACAAAGTATCTAAACAAAATACATAAGTGTACTCAAACTGAGTA
125
              ||||||||||||||||||||||||||||||||||||||||||||||||||||||||||||
Sbjct: 212394 GTATACCTATAAATTAACAAAGTATCTAAACAAAATACATAAGTGTACTCAAACTGAGTA
212453


Query: 126    GAATCGTCGATTAAACTTCCTTCTCCTTTTAAAAATTAAAAACAGCAAATAGTTAGATGA
185
```

```
              ||||||||||||||||||||||||||||||||||||||||||||||||||||||||||||
Sbjct: 212454 GAATCGTCGATTAAACTTCCTTCTCCTTTTAAAAATTAAAAACAGCAAATAGTTAGATGA
212513

Query: 186    ATATATTAAAGACTATTCGTTTTATTTCCCAGAGCAGCATGACTTCTTGGTTTCTTCAGA
245
              |||||||||||||||||||||| |||||||||||||||||||||||||||||||||||||
Sbjct: 212514 ATATATTAAAGACTATTCGTTTCATTTCCCAGAGCAGCATGACTTCTTGGTTTCTTCAGA
212573


Query: 246    CTTGTTACCGCAGGGGCATTTGTCGTCGCTGTTACACCCCGTTGGGCAGCTACATGATTT
305
              ||||||||||||||||||||||||||||||||||||||||||||||||||||||||||||
Sbjct: 212574 CTTGTTACCGCAGGGGCATTTGTCGTCGCTGTTACACCCCGTTGGGCAGCTACATGATTT
212633

Query: 306    TTGGCATTGTTCATTATTTTTGCAGCTACCACATTGGCATTGGCACTCATGACCTTCATT
365
              ||||||||||||||||||||||||||||||||||||||||||||||||||||||||||||
Sbjct: 212634 TTGGCATTGTTCATTATTTTTGCAGCTACCACATTGGCATTGGCACTCATGACCTTCATT
212693

Query: 366    TTGGAAGTTAATTAATTCGCTGAACATTTTATGTGATGATTGATTGATTG----TACGGT
421
              ||||||||||||||||||||||||||||||||||||||||||||||||||    ||| ||
Sbjct: 212694 TTGGAAGTTAATTAATTCGCTGAACATTTTATGTGATGATTGATTGATTGATTGTACAGT
212753

Query: 422    TTGTTTTTCTTAATATCTATTTCGATGACTTCTATATGATATTGCACTAACAAGAAGATA
481
              ||||||||||||||||||||||||||||||||||||||||||||||||||||||||||||
Sbjct: 212754 TTGTTTTTCTTAATATCTATTTCGATGACTTCTATATGATATTGCACTAACAAGAAGATA
212813

Query: 482    TTATAATGCAATTGATACAAGACAAGGAGTTATTTGCTTCTCTTTTATATGATTCTGACA
541
              ||||||||||||||||||||||||||||||||||||||||||||||||||||||||||||
Sbjct: 212814 TTATAATGCAATTGATACAAGACAAGGAGTTATTTGCTTCTCTTTTATATGATTCTGACA
212873

Query: 542    ATCCATATTGCGTTGGTAGTCTTTTTTGCTGGAACGGTTCAGCGGAAAAGACGCATCGCT
601
              ||||||||||||||||||||||||||||||||||||||||||||||||||||||||||||
Sbjct: 212874 ATCCATATTGCGTTGGTAGTCTTTTTTGCTGGAACGGTTCAGCGGAAAAGACGCATCGCT
212933
```

F1

```
Query: 234    CTTTTTGCTTCTA 246
              |||||||||||||
Sbjct: 212934 CTTTTTGCTTCTA 212946


Query: 247    GAAGAAATGCCAGCAAAAGAATCTCTCGACAGTGACTGACAGCAAAAATGTCTTTTTCTA
306
              |||||||||||||||||||||||||| ||||||||||||||||||||||||||||||||||
Sbjct: 212947 GAAGAAATGCCAGCAAAAGAATCTCTTGACAGTGACTGACAGCAAAAATGTCTTTTTCTA
213006


Query: 307    ACTAGTAACAAGGCTAAGATATCAGCCTGAAATAAAGGGTGGTGAAGTAATAATTAAATC
366
              ||||||||||||||||||||||||||||||||||||||||||||||||||||||||||||
Sbjct: 213007 ACTAGTAACAAGGCTAAGATATCAGCCTGAAATAAAGGGTGGTGAAGTAATAATTAAATC
213066


Query: 367    ATCCGTATAAACCTATACACATATATGAGGAAAAATAATACAAAAGTGTTTTAAATACAG
426
              ||||||||||||||||||||||||||||||||||||||||||||||||||||||||||||
Sbjct: 213067 ATCCGTATAAACCTATACACATATATGAGGAAAAATAATACAAAAGTGTTTTAAATACAG
213126


Query: 427    ATACATACATGAACATATGCACGTATAGCGTCCAAATGTCGGTAATGGGATCGGCTTACT
486
              |||||||||||||||||||||||||||||| |||||||||||||||||||||||||||||
Sbjct: 213127 ATACATACATGAACATATGCACGTATAGCGCCCAAATGTCGGTAATGGGATCGGCTTACT
213186


Query: 487    AATTATAAAATGCATCATAGAAATCGT 513
              |||||||||||||||||||||||||||
Sbjct: 213187 AATTATAAAATGCATCATAGAAATCGT 213213
```

<u>3. *CUP1-RSC30* (VIII212913-213513)</u>

VIII212300 F

```
Query: 581    CAGCGGAAAAGACGCATCGCT 601
              |||||||||||||||||||||
Sbjct: 212913 CAGCGGAAAAGACGCATCGCT 212933
```

F1

```
Query: 234    CTTTTTGCTTCTA 246
              |||||||||||||
Sbjct: 212934 CTTTTTGCTTCTA 212946
```

```
Query: 247    GAAGAAATGCCAGCAAAAGAATCTCTCGACAGTGACTGACAGCAAAAATGTCTTTTTCTA
306
              |||||||||||||||||||||||||| |||||||||||||||||||||||||||||||||
Sbjct: 212947 GAAGAAATGCCAGCAAAAGAATCTCTTGACAGTGACTGACAGCAAAAATGTCTTTTTCTA
213006


Query: 307    ACTAGTAACAAGGCTAAGATATCAGCCTGAAATAAAGGGTGGTGAAGTAATAATTAAATC
366
              ||||||||||||||||||||||||||||||||||||||||||||||||||||||||||||
Sbjct: 213007 ACTAGTAACAAGGCTAAGATATCAGCCTGAAATAAAGGGTGGTGAAGTAATAATTAAATC
213066


Query: 367    ATCCGTATAAACCTATACACATATATGAGGAAAAATAATACAAAAGTGTTTTAAATACAG
426
              ||||||||||||||||||||||||||||||||||||||||||||||||||||||||||||
Sbjct: 213067 ATCCGTATAAACCTATACACATATATGAGGAAAAATAATACAAAAGTGTTTTAAATACAG
213126


Query: 425    ATACATACATGAACATATGCACGTATAGCGTCCAAATGTCGGTAATGGGATCGGCTTA 482
              |||||||||||||||||||||||||||||| |||||||||||||||||||||||||||
Sbjct: 213127 ATACATACATGAACATATGCACGTATAGCGCCCAAATGTCGGTAATGGGATCGGCTTA
213184


Query: 483    CTAATTATAAAATGCATCATAGAAATCGTTGAAGTTTGCCGTAGTAATACCCAGATTATC
542
              ||||||||||||||||||||||||||||||||||||||||||||||||||||||||||||
Sbjct: 213185 CTAATTATAAAATGCATCATAGAAATCGTTGAAGTTTGCCGTAGTAATACCCAGATTATC
213244


Query: 543    AGATTCCAAATCCTTGTCAATAATTATACTCCTTTGGAAAACTTCTCTTTCCATTAAAAA
602
              ||||||||||||||||||||||||||||||||||||||| ||||||||||||||||||||
Sbjct: 213245 AGATTCCAAATCCTTGTCAATAATTATACTCCTTTGGACAACTTCTCTTTCCATTAAAAA
213304


Query: 79     ATCTGAAATCTCCTTAAATTTTAAATAGATTCTGTTCAGTTCACTAACGG 128
              ||||||||||||||||||||||||||||||||||||||||||||||||||
Sbjct: 213305 ATCTGAAATCTCCTTAAATTTTAAATAGATTCTGTTCAGTTCACTAACGG 213354


Query: 129    GGAATTTCAAGAGAACATTTTTGTTCTTCGCCGACTGACTATAATCTGTAACATTATTGT
188
              ||||||||||||||||||||||||||||||||||||||||||||||||||||||||||||
Sbjct: 213355 GGAATTTCAAGAGAACATTTTTGTTCTTCGCCGACTGACTATAATCTGTAACATTATTGT
213414


Query: 189    TATCAGAGTTTCTCGCAAAATTTTGTTTTTTCTTGCTAAATCTCAGCATATATTTAATCA
248
              ||||||||||||||||||||||||||||||||||||||||||||||||||||||||||||
```

```
Sbjct: 213415 TATCAGAGTTTCTCGCAAAATTTTGTTTTTTCTTGCTAAATCTCAGCATATATTTAATCA
213474


Query: 249    GATTCAAAACCTTGTTGAAACCTTTAATAGATTTGAAAT 287
              |||||||||||||||||||||||||||||||||||||||
Sbjct: 213475 GATTCAAAACCTTGTTGAAACCTTTAATAGATTTGAAAC 213513
```

SNPs between YJM969 and S288c

Sequenced interval	Coordinate(s)	SNP in YJM969	SNP in S288c
CIC1-CUP1 VIII211275-211875			
	211462	C	T
	211534	A	T
	211550	G	C
	211655	G	C
	211693	A	G
	211701	A	G
	211825	A	G
CUP1 repeat VIII211575-213213			
	211655	G	T
	211693	A	G
	211701	A	G
	211825	A	G
	212243	G	C
	212266-212274	10 T’s	9 T’s
	212536	T	C
	212744-212747	4 bp deletion	ATTG
	212751	G	A
	212973	C	T
	213157	T	C
CUP1-RSC30 VIII212913-213513			
	212973	C	T
	213157	T	C
	213283	A	C

Table S10 Sequence analysis of strain (DTY3) that has a single copy of *CUP1*.

To determine the sequence of the single-copy *CUP1* gene and its flanking sequences, we generated three PCR fragments that had overlapping sequences as described in Supporting Data File S1. These fragments were sequenced using the primers shown in boldface below. The region between the 5' end of *CIC1* and the 5' end of *RSC30*, including the *CUP1* gene, is shown as two continuous sequences. One sequence matches with SGD coordinates 210848 (5' end of *CIC1*) to 213868 (in the 3' region of *RSC30*). The other sequence matches between 215867 (3' region of *RSC30*) to 217834 (the 5' end of *RSC30*). The discontinuity in the comparison between the DTY3 sequences and those of S288c arises because the S288c sequence contains two copies of the 2.0 kb Type 1 *CUP1* repeat instead of the single *CUP1* gene present in DTY3.

1. VIII210848-VIII213868

VIII210632 F

```
Query: 189    ATGGCTAAAAAGAGTAACTCAAAGAAATCTACGCCTGTAAGTACACCAAGCAAAG 243
              |||||||||||||||||||||||||||||||||||||||||||||||||||||||
Sbjct: 210848 ATGGCTAAAAAGAGTAACTCAAAGAAATCTACGCCTGTAAGTACACCAAGCAAAG 210902


Query: 244    AAAAGAAGAAGGTTATTGAAAAGAAATCTTCCACAGCCATTCCTAGGGAAAGAGTTATTA
303
              ||||||||||||||||||||||||||||||||||||||||||||||||||||||||||||
Sbjct: 210903 AAAAGAAGAAGGTTATTGAAAAGAAATCTTCCACAGCCATTCCTAGGGAAAGAGTTATTA
210962


Query: 304    AAGCTGTCAACGAGCTTATAAAATTCACTTCCAAGCCACAAGATGAAAATAATGAAGAAG
363
              ||||||||||||||||||||||||||||||||||||||||||||||||||||||||||||
Sbjct: 210963 AAGCTGTCAACGAGCTTATAAAATTCACTTCCAAGCCACAAGATGAAAATAATGAAGAAG
211022


Query: 364    GAAATAACGGTAAGAAAAACCTATTGGAAGATGATGAAGAAGAATTGAAGAAAGATCTGC
423
              ||||||||||||||||||||||||||||||||||||||||||||||||||||||||||||
Sbjct: 211023 GAAATAACGGTAAGAAAAACCTATTGGAAGATGATGAAGAAGAATTGAAGAAAGATCTGC
211082


Query: 424    AATTAATCGTAGTAAATAATAAATCATTCACCGGTACTTCCAAATCATTCAAATTGAAAT
483
              ||||||||||||||||||||||||||||||||||||||||||||||||||||||||||||
```

```
Sbjct: 211083 AATTAATCGTAGTAAATAATAAATCATTCACCGGTACTTCCAAATCATTCAAATTGAAAT
211142

Query: 484    TACTAAATGTCAAACATTCGTTTTACAAGCCTTGGAAAGAAGCCAGTGCAACAGCGGTTA
543
              ||||||||||||||||||||||||||||||||||||||||||||||||||||||||||||
Sbjct: 211143 TACTAAATGTCAAACATTCGTTTTACAAGCCTTGGAAAGAAGCCAGTGCAACAGCGGTTA
211202

Query: 544    AGGATTTCAAAGTTTTATTAATTTTGAAGGATTCTGATATTAAGAAAGTTTCAGAAGATG
603
              ||||||||||||||||||||||||||||||||||||||||||||||||||||||||||||
Sbjct: 211203 AGGATTTCAAAGTTTTATTAATTTTGAAGGATTCTGATATTAAGAAAGTTTCAGAAGATG
211262

Query: 604    ATTTATTTGATCAATTAGATTCAGAAGGAATCAAGGTTGATGAAATCATTTGCGGGAAAG
663
              ||||||||||||||||||||||||||||||||||||||||||||||||||||||||||||
Sbjct: 211263 ATTTATTTGATCAATTAGATTCAGAAGGAATCAAGGTTGATGAAATCATTTGCGGGAAAG
211322

Query: 664    ACTTAAAGACCGTTTACAAGGCATATGAGGCTAGAAACGCTTTTATATCTCAGTT 718
              |||||||||||||||||||||||||||||||||||||||||||||||||||||||
Sbjct: 211323 ACTTAAAGACCGTTTACAAGGCATATGAGGCTAGAAACGCTTTTATATCTCAGTT 211377
```

VIII212063 R

```
Query: 658    TTCTTTGATTTTGGCTGACGACAGTATAGTTACATCTTTGCCAAAACTTATGGGAGGCAA
599
              ||||||||||||||||||||||||||||||||||||||||||||||||||||||||||||
Sbjct: 211378 TTCTTTGATTTTGGCTGACGACAGTATAGTTACATCTTTGCCAAAACTTATGGGAGGCAA
211437

Query: 598    AGCCTACAACAAAGTAGAAACTACTCCTATATCAATTAGAACACATGCAAATAAGGAATT
539
              ||||||||||||||||||||||||||||||||||||||||||||||||||||||||||||
Sbjct: 211438 AGCCTACAACAAAGTAGAAACTACTCCTATATCAATTAGAACACATGCAAATAAGGAATT
211497

Query: 538    TTCCTTGACCACTTTGACGAACAATATCAAAAAGGTTTACATGAATCAGTTGCCCGTTAA
479
              ||||||||||||||||||||||||||||||||||||||||||||||||||||||||||||
Sbjct: 211498 TTCCTTGACCACTTTGACGAACAATATCAAAAAGGTTTACATGAATCAGTTGCCCGTTAA
211557

Query: 478    ACTTCCAAGAGGTACCACGTTGAATGTCCATTTGGGTAATTTAGAATGGTTAAGGCCAGA
419
              ||||||||||||||||||||||||||||||||||||||||||||||||||||||||||||
```

```
Sbjct: 211558 ACTTCCAAGAGGTACCACGTTGAATGTCCATTTGGGTAATTTAGAATGGTTAAGGCCAGA
211617

Query: 418    AGAGTTTGTAGATAACGTTGAATTAATTTCTGAACAGTTAATCAAAGCATACCAAATCAG
359
              ||||||||||||||||||||||||||||||||||||||||||||||||||||||||||||
Sbjct: 211618 AGAGTTTGTAGATAACGTTGAATTAATTTCTGAACAGTTAATCAAAGCATACCAAATCAG
211677

Query: 358    ATCCATTTTTATCAAAACCAATAAGTCGCCCGTATTGCCATTATACTATAACCAGGACGT
299
              ||||||||||||||| ||||||| ||||||||||||||||||||||||||||||||||||
Sbjct: 211678 ATCCATTTTTATCAAGACCAATAGGTCGCCCGTATTGCCATTATACTATAACCAGGACGT
211737

Query: 298    TCTTGATGAACTTGAAGCTAAAAAGGACAAAATCGAAGAAACCCACGAAGATGACATGGT
239
              ||||||||||||||||||||||||||||||||||||||||||||||||||||||||||||
Sbjct: 211738 TCTTGATGAACTTGAAGCTAAAAAGGACAAAATCGAAGAAACCCACGAAGATGACATGGT
211797

Query: 238    CACCATTGATGGTGTACAAGTTCATTTATCTACCTTCAACAAGGGTTTGATGGAAATCGC
179
              |||||||||||||||||||||||||||| ||||||||||||||||||||||||||||||||
Sbjct: 211798 CACCATTGATGGTGTACAAGTTCATTTGTCTACCTTCAACAAGGGTTTGATGGAAATCGC
211857

Query: 178    CAATCCTTCCGAATTGGGTTCAATTTTCTCTAAACAAATTAACAATGCAAAAAA 112
              ||||||||||||||||||||||||||||||||||||||||||||||||||||||
Sbjct: 211858 CAATCCTTCCGAATTGGGTTCAATTTTCTCTAAACAAATTAACAATGCAAAAAA 211910
```

VIII211849 F

```
Query: 33     GAGATCTTCTAGCGAGCTTGAAAAAGAATCTA 64
              ||||||||||||||||||||||||||||||||
Sbjct: 211911 GAGATCTTCTAGCGAGCTTGAAAAAGAATCTA 211943

Query: 65     GCGAGTCAGAAGCTGTCAAGAAGACTAAAAGTTAATTTGTGTCCTCCTTATCTATCTTTT
124
              ||||||||||||||||||||||| |||||||||||||||| |||||||||||||||||||
Sbjct: 211944 GCGAGTCAGAAGCTGTCAAGAAGGCTAAAAGTTAATTTGTTTCCTCCTTATCTATCTTTT
212003

Query: 125    CTCTCATTTTTTTCTTGTGAAGAAAAAAATTTGAATTTCATAGAGTGCGGTGCATATGTA
184
              ||||||||||||||||||||||||||||||||||||||||||||||||||||||||||||
Sbjct: 212004 CTCTCATTTTTTTCTTGTGAAGAAAAAAATTTGAATTTCATAGAGTGCGGTGCATATGTA
212063
```

```
Query: 185    TATATCTATATATGTTTGAAGTGTATATTAAAAATAAAGTCATTATTTGAATATTGGTTT
244
              ||||||||||||||||||||||||||||||||||||||||||||||||||||||||||||
Sbjct: 212064 TATATCTATATATGTTTGAAGTGTATATTAAAAATAAAGTCATTATTTGAATATTGGTTT
212123


Query: 245    CTCGGTCTAAGAGCTTATACGTTTTAGACTGATCTGTTGTACTATCCGCTTCAAATAAAT
304
              ||||||||||||||||||||||||||||||||||||||||||||||||||||||||||||
Sbjct: 212124 CTCGGTCTAAGAGCTTATACGTTTTAGACTGATCTGTTGTACTATCCGCTTCAAATAAAT
212183


Query: 305    AGATCATTGAAAGTGACGGGGATAACAGCATTTTACCTTTAAAAGACGTTCTCATAATAC
364
              ||||||||||||||||||||||||||||||||||||||||||||||||||||||||||||
Sbjct: 212184 AGATCATTGAAAGTGACGGGGATAACAGCATTTTACCTTTAAAAGACGTTCTCATAATAC
212243


Query: 365    ATTTTAGGATTAATACATATGCTTTTTTTTTATTCAAAATCTGGGGATTTTATACAGAGT
424
              ||||||||||||||||||||||||||||||||||||| ||||||||||||| ||||||||||
Sbjct: 212244 ATTTTAGGATTAATACATATGCTTTTTTTTTATTCGAAATCTGGGGATTCTATACAGAGT
212303


Query: 425    TGTAAGTTAGGCAAACTAGAATTTGGTAATAATATTTTATTCTTGGGGCGACATATGGAG
484
              ||||||||||||||||||||||||||||||||||||||||||||||||||||||||||||
Sbjct: 212304 TGTAAGTTAGGCAAACTAGAATTTGGTAATAATATTTTATTCTTGGGGCGACATATGGAG
212363


Query: 485    ATACTTTATTTCCTTTTCTTAATTATTAACGTATACCTATAAATTAACAAAGTATCTAAA
544
              ||||||||||||||||||||||||||||||||||||||||||||||||||||||||||||
Sbjct: 212364 ATACTTTATTTCCTTTTCTTAATTATTAACGTATACCTATAAATTAACAAAGTATCTAAA
212423


Query: 545    CAAAATACATAAGTGTACTCAAACTGAGTAGAATCGTCGATTAAACTTCCTTCTCCTTTT
604
              ||||||||||||||||||||||||||||||||||||||||||||||||||||||||||||
Sbjct: 212424 CAAAATACATAAGTGTACTCAAACTGAGTAGAATCGTCGATTAAACTTCCTTCTCCTTTT
212483
```

VIII212300 F

```
Query: 157    AAAAATTAAAAACAGCAAATAGTTAAATG 185
              ||||||||||||||||||||||||| |||
Sbjct: 212484 AAAAATTAAAAACAGCAAATAGTTAGATG 212512
```

```
Query: 186    AATATATTAAAGACTATTCGTTTTATTTCCCAGAGCAGCATGATTTCTTGGTTTCTTCAG
245
              ||||||||||||||||||||||| ||||||||||||||||||| ||||||||||||||||
Sbjct: 212513 AATATATTAAAGACTATTCGTTTCATTTCCCAGAGCAGCATGACTTCTTGGTTTCTTCAG
212572

Query: 246    ACTTGTTACCGCAGGGGCATTTGTCGTCGCTGTTACACCCCGTTGGGCAGCTACATGATT
305
              ||||||||||||||||||||||||||||||||||||||||||||||||||||||||||||
Sbjct: 212573 ACTTGTTACCGCAGGGGCATTTGTCGTCGCTGTTACACCCCGTTGGGCAGCTACATGATT
212632

Query: 306    TTTGGCATTGTTCATTATTTTTGCAGCTACCACATTGGCATTGGCACTCATGACCTTCAT
365
              ||||||||||||||||||||||||||||||||||||||||||||||||||||||||||||
Sbjct: 212633 TTTGGCATTGTTCATTATTTTTGCAGCTACCACATTGGCATTGGCACTCATGACCTTCAT
212692

Query: 366    TTTGGAAGTTAATTAATTCGCTGAACATTTTATGTGATGATTGATTGATTG----TACGG
421
              |||||||||||||||||||||||||||||||||||||||||||||||||||    ||| |
Sbjct: 212693 TTTGGAAGTTAATTAATTCGCTGAACATTTTATGTGATGATTGATTGATTGATTGTACAG
212752

Query: 422    TTTGTTTTTGTTAATATCTATTTCGATGACTTCTATATGATATTGCACTAACAAGAAGAT
481
              ||||||||| ||||||||||||||||||||||||||||||||||||||||||||||||||
Sbjct: 212753 TTTGTTTTTCTTAATATCTATTTCGATGACTTCTATATGATATTGCACTAACAAGAAGAT
212812

Query: 482    ATTATAATGCAATTGGTACAAGACAAGGAGTTATTTGCTTCTCTTTTATATGATTCTGAC
541
              ||||||||||||||| ||||||||||||||||||||||||||||||||||||||||||||
Sbjct: 212813 ATTATAATGCAATTGATACAAGACAAGGAGTTATTTGCTTCTCTTTTATATGATTCTGAC
212872

Query: 542    AATCCATATTGCGTTGGTAGTCTTTTTTGCTGGAACGGTTCAGCGGAAAAGACGCATCGC
601
              ||||||||||||||||||||||||||||||||||||||||||||||||||||||||||||
Sbjct: 212873 AATCCATATTGCGTTGGTAGTCTTTTTTGCTGGAACGGTTCAGCGGAAAAGACGCATCGC
212932
```

F1

```
Query: 242    TCTTTTTGCTTCTAGAAGAAATGCCAGCAAAAGAATCTCTTGACAGTGACTGACAGCAAA
301
              ||||||||||||||||||||||||||||||||||||||||||||||||||||||||||||
```

```
Sbjct: 212933 TCTTTTTGCTTCTAGAAGAAATGCCAGCAAAAGAATCTCTTGACAGTGACTGACAGCAAA
212992


Query: 302    AATGTCTTTTTCTAACTAGTAACAAGGCTAAGATATCAGCCTGAAATAAAGGGTGGTGAA
361
              ||||||||||||||||||||||||||||||||||||||||||||||||||||||||||||
Sbjct: 212993 AATGTCTTTTTCTAACTAGTAACAAGGCTAAGATATCAGCCTGAAATAAAGGGTGGTGAA
213052


Query: 362    GTAATAATTAAATCATCCGTATAAACCTATACACATATATGAGGAAAAAATAATACAAAA
421
              ||||||||||||||||||||||||||||||||||||||||||||||||| ||||||||||
Sbjct: 213053 GTAATAATTAAATCATCCGTATAAACCTATACACATATATGAGGAAAAA-TAATACAAAA
213111


Query: 422    GTGTTTTAAATACAGATACATACATGAACATATGCACGTATAGCGTCCAAATGTCGGTAA
481
              ||||||||||||||||||||||||||||||||||||||||||||| ||||||||||||||
Sbjct: 213112 GTGTTTTAAATACAGATACATACATGAACATATGCACGTATAGCGCCCAAATGTCGGTAA
213171


Query: 482    TGGGATCGGCTTACTAATTATAAAATGCATCATAGAAATCGTTGAAGTTTGCCGTAGTAA
541
              ||||||||||||||||||||||||||||||||||||||||||||||||||||||||||||
Sbjct: 213172 TGGGATCGGCTTACTAATTATAAAATGCATCATAGAAATCGTTGAAGTTTGCCGTAGTAA
213231


Query: 542    TACCCAGATTATCAGATTCCAAATCCTTGTCAATAATTATACTCCTTTGGAAAACTTCTC
601
              ||||||||||||||||||||||||||||||||||||||||||||||||||| ||||||||
Sbjct: 213232 TACCCAGATTATCAGATTCCAAATCCTTGTCAATAATTATACTCCTTTGGACAACTTCTC
213291

Query: 602    TTTCCATTAAAAAATCTGAAATCTCCTTAAATTTTAAATAGATTCTGTTCAGTTCACTAA
661
              ||||||||||||||||||||||||||||||||||||||||||||||||||||||||||||
Sbjct: 213292 TTTCCATTAAAAAATCTGAAATCTCCTTAAATTTTAAATAGATTCTGTTCAGTTCACTAA
213351
```

VIII213234 F

```
Query: 87     CGGGGAATTTCAAGAGAACATTTTTGTTCTTCGCCGA 123
              |||||||||||||||||||||||||||||||||||||
Sbjct: 213352 CGGGGAATTTCAAGAGAACATTTTTGTTCTTCGCCGA 213388


Query: 124    CTGACTATAATCTGTAACATTATTATTATCAGAGTTTCTCGCAAAATTTTGTTTTTTCTT
183
              |||||||||||||||||||||||| |||||||||||||||||||||||||||||||||||
```

```
Sbjct: 213389 CTGACTATAATCTGTAACATTATTGTTATCAGAGTTTCTCGCAAAATTTTGTTTTTTCTT
213448


Query: 184    GCTAAATCTCAGCATATATTTAATCAGATTCAAAACCTTGTTGAAACCTTTAATAGATTT
243
              ||||||||||||||||||||||||||||||||||||||||||||||||||||||||||||
Sbjct: 213449 GCTAAATCTCAGCATATATTTAATCAGATTCAAAACCTTGTTGAAACCTTTAATAGATTT
213508


Query: 244    GAAATTTCCGTTGCTATTCATTTCATCCCGTAAAAAGGATACGATAATTTCTATTTTTTT
303
              |||| |||||||||||||||||||||| ||||||||||||||||||||||||||||||||
Sbjct: 213509 GAAACTTCCGTTGCTATTCATTTCATCTCGTAAAAAGGATACGATAATTTCTATTTTTTT
213568


Query: 304    TAAAATTTCCAAAATCTTGTCATGAATCAATAGCAATTGAACATTAATCTCCTCATTTGA
363
              ||||||||||||||||||||||||||||||||||||||||||||||||||||||||||||
Sbjct: 213569 TAAAATTTCCAAAATCTTGTCATGAATCAATAGCAATTGAACATTAATCTCCTCATTTGA
213628


Query: 364    AAGATTTTTGTAAAATTCGTCATATAATATTACTTCACAACGTTGGAAAATAGCAAATGT
423
              ||||||||||||||||||||||||||||||||||||||||||||||||||||||||||||
Sbjct: 213629 AAGATTTTTGTAAAATTCGTCATATAATATTACTTCACAACGTTGGAAAATAGCAAATGT
213688


Query: 424    GATTGCTATAAAATTCTGTAAGATTTCAATAAAATGATTTGCGAATAAAAATTCTTTACC
483
              ||||||||||||||||||||||||||||||||||||||||||||||||||||||||||||
Sbjct: 213689 GATTGCTATAAAATTCTGTAAGATTTCAATAAAATGATTTGCGAATAAAAATTCTTTACC
213748


Query: 484    ATTAGAATGAAAGCGATTATTGCCGCTTGAAAATGACTTTATCGACTTTATGGGGAAGAT
543
              ||||||||||||||||||||||||||||||||||||||||||||||||||||||||||||
Sbjct: 213749 ATTAGAATGAAAGCGATTATTGCCGCTTGAAAATGACTTTATCGACTTTATGGGGAAGAT
213808


Query: 544    AAAATTAAATGTTACTGAGTAAAAAATGTGCATATTAGAAATAATTTTCATCAGATCCTT
603
              |||||||||||||| |||||||||||||||||||||||||||||||||||||||||||||
Sbjct: 213809 AAAATTAAATGTTATTGAGTAAAAAATGTGCATATTAGAAATAATTTTCATCAGATCCTT
213868
```

2. VIII215871-VIII217834

VIII213601 F

```
Query: 240    TGCA 243
              ||||
Sbjct: 215867 TGCA 215870


Query: 244    CATCTTTCAGAGTTCGAGGTCTTATTGTTGTTAGAAGAATGCTGAACTGCCATGGACAAA
303
              ||||||||||||||||||||||||||||||||||||||||| ||||||||||||||||||
Sbjct: 215871 CATCTTTCAGAGTTCGAGGTCTTATTGTTGTTAGAAGAATGTTGAACTGCCATGGACAAA
215930


Query: 304    GAGGATTCGTTTTGAACAAAAAGGAAAAAATTTGTATAAACAATGGTATTGATAAAATTT
363
              ||||||||||||||||||||||||||||||||||||||||||||||||||||||||||||
Sbjct: 215931 GAGGATTCGTTTTGAACAAAAAGGAAAAAATTTGTATAAACAATGGTATTGATAAAATTT
215990


Query: 364    AAAGTGTCTTTCCATTCTTTTCTGACTTCGTTGTCATGAAAATATAAGTCTACTGTATTA
423
              ||||||||||||||||||||||||||||||||||||||||||||||||||||||||||||
Sbjct: 215991 AAAGTGTCTTTCCATTCTTTTCTGACTTCGTTGTCATGAAAATATAAGTCTACTGTATTA
216050


Query: 424    CTCACGCCCATAGTCAAGGTTTCTAACAGACTTTCAATTTTGGTTAAATTTACTGGCAAG
483
              ||||||||||||||||||||||||||||||||||||||||||||||||||||||||||||
Sbjct: 216051 CTCACGCCCATAGTCAAGGTTTCTAACAGACTTTCAATTTTGGTTAAATTTACTGGCAAG
216110


Query: 484    TAGAAAGGAACATCTTGCAGAATATTTATCAATTTTGCTTGCGTTTCCAGTAATTTTAAA
543
              |||||||||||| |||||||||||||||||||||||||||||||||||||||||||||||
Sbjct: 216111 TAGAAAGGAACACCTTGCAGAATATTTATCAATTTTGCTTGCGTTTCCAGTAATTTTAAA
216170


Query: 544    TCGTTAGCAATTAAAGGAATGTCGTTCGTATCAATAGAGGCAGGTATCGGAGATAGGTTT
603
              ||||||||||||||||||||||||||||||||||||||||||||||||||||||||||||
Sbjct: 216171 TCGTTAGCAATTAAAGGAATGTCGTTCGTATCAATAGAGGCAGGTATCGGAGATAGGTTT
216230


Query: 604    TCAGTAGCGGGTACCATGAATGAAGACTGACCTAGAAGCGAATGTCTTGAGTAATACATT
663
              |||| |||||||||||||||||||||||||||||||||||||||||||||||||||||||
Sbjct: 216231 TCAGCAGCGGGTACCATGAATGAAGACTGACCTAGAAGCGAATGTCTTGAGTAATACATT
216290
```

```
Query: 664    TTTTTCAAAAAATGGAATAACTTGAAATCTTTTATCTGGAAGCTTAA 710
              |||||||||||||||||||||||||||||||||||||||||||||||
Sbjct: 216291 TTTTTCAAAAAATGGAATAACTTGAAATCTTTTATCTGGAAGCTTAA 216337
```

VIII216859 R

```
Query: 489    GAGACAGCTCAAATCTTCG 471
               ||||||||||||||||||
Sbjct: 216338 CAGACAGCTCAAATCTTCG 216356


Query: 470    TCTAAACTTGATTTATGATCATCATGCATGTACAGAGATTCATAAAATTTTGTGATGGCA
411
              ||||||||||||||||||||||||||||||||||||||||||||||||||||||||||||
Sbjct: 216357 TCTAAACTTGATTTATGATCATCATGCATGTACAGAGATTCATAAAATTTTGTGATGGCA
216416


Query: 410    ATAAATTTGATTGTCTCTTGGTCATAATATTCCAGGTTTATCAATGTGATTTGGCTTCGA
351
              ||||||||||||||||||||||||||||||||||||||||||||||||||||||||||||
Sbjct: 216417 ATAAATTTGATTGTCTCTTGGTCATAATATTCCAGGTTTATCAATGTGATTTGGCTTCGA
216476


Query: 350    ATCAACCTCAAATTATTCATTAATGCCGAAACATGGTTATTAATAGCCTGCTTATTGAAT
291
              ||||||||||||||||||||||||||||||||||||||||||||||||||||||||||||
Sbjct: 216477 ATCAACCTCAAATTATTCATTAATGCCGAAACATGGTTATTAATAGCCTGCTTATTGAAT
216536


Query: 290    AGGGTCATTGAATCGTTTAACAACAGCAGAAGCACAGTTAATTCGCCTAGTTTGGTCAGT
231
              |||||||||||||||||||||||||| |||||||||||||||||||||||||||||||||
Sbjct: 216537 AGGGTCATTGAATCGTTTAACAACAGTAGAAGCACAGTTAATTCGCCTAGTTTGGTCAGT
216596


Query: 230    TGAGATAAAGGAAGATCGACCGTTGAGAATGAAGGACAGGTATCACCATTTGGAAAAGAG
171
              ||||||||||||||||||||||||||||||||||||||||||||||||||||||||||||
Sbjct: 216597 TGAGATAAAGGAAGATCGACCGTTGAGAATGAAGGACAGGTATCACCATTTGGAAAAGAG
216656


Query: 170    TTATGAACAAATTCTAGTAAGAGGGTTTTGTCAAAGATTGGCAAAAATTCCTCAATATTC
111
              |||||||||||||||||||||||||||||||||||||||||||||||||| |||||||||
Sbjct: 216657 TTATGAACAAATTCTAGTAAGAGGGTTTTGTCAAAGATTGGCAAAAATTCTTCAATATTC
216716


Query: 110    AGATTGTTAAGGGATTCAGAGTTTAGTGTCTCAATAATTATTGCTTTTGGTGGGAAGTTA 51
```

```
              ||||||||||||||||||||||||||||||||||||||||||||||||||||||||||||
Sbjct: 216717 AGATTGTTAAGGGATTCAGAGTTTAGTGTCTCAATAATTATTGCTTTTGGTGGGAAGTTA
216776
```

VIII216894 R

```
Query: 89     ACTTTGCAAACTTCCATATATTGTTGGTGATTTTTATCGTTAGGAGGACT 40
              |||||||||||||||||||||||||||||||||||||| ||||||||||||
Sbjct: 216777 ACTTTGCAAACTTCCATATATTGTTGGTGATTTTTATTGTTAGGAGGACT 216826
```

VIII216763 F

```
Query: 38     TACTTTGAACTGATTAATATGGC 60
              |||||||||||||||||||||||
Sbjct: 216827 TACTTTGAACTGATTAATATGGC 216849

Query: 61     AGATATCGTGCAAAATATTATAGAACTTGAAAAGATAATGATCTCTAAAAATGATGAAGT
120
              ||||||||||||||||||||||||||||||||||||||||||||||||||||||||||||
Sbjct: 216850 AGATATCGTGCAAAATATTATAGAACTTGAAAAGATAATGATCTCTAAAAATGATGAAGT
216909

Query: 121    TTTTAGTGAAAAGGTAGTTGGGGGTATCCTTCAACGGTTTCACTTGGACTAAACTTTGCA
180
              ||||||||||||||||||||||||||||||||||||||||||||||||||||||||||||
Sbjct: 216910 TTTTAGTGAAAAGGTAGTTGGGGGTATCCTTCAACGGTTTCACTTGGACTAAACTTTGCA
216969

Query: 181    CATCTGTTATTGTGAGCGCTGTAAAGTATTTTTGTGAGGACTGAGAAGTCCTTGAAATCT
240
              |||||||||||||||||||||||||||||||||||||||||||||||||||||||| |||
Sbjct: 216970 CATCTGTTATTGTGAGCGCTGTAAAGTATTTTTGTGAGGACTGAGAAGTCCTTGAAGTCT
217029

Query: 241    GATGATCAAATCCGGCTTTTTTACCGTCAACCTTGCCTTTCTTAGAATTCTCACCTGTTT
300
              |||||||||||||||| |||||||||||||||||||||||||||||||||||||||||||
Sbjct: 217030 GATGATCAAATCCGGCTCTTTTACCGTCAACCTTGCCTTTCTTAGAATTCTCACCTGTTT
217089

Query: 301    TGTTAATTTTGTTTGCCGTGGTATTGTCGGCATTTAAATTCGATTTTTCTTTCAAACTTT
360
              ||||||||||||||||||||||||||||||||||||||||||||||||||||||||||||
Sbjct: 217090 TGTTAATTTTGTTTGCCGTGGTATTGTCGGCATTTAAATTCGATTTTTCTTTCAAACTTT
217149
```

```
Query: 361    TCAGTTGCAAGCTTTGTAAATCAAGCAATTTGTTTTTTAAAAAGTCTATCTCCTTGATTA
420
              ||||||||||||||||||||||||||||||||||||||||||||||||||||||||||||
Sbjct: 217150 TCAGTTGCAAGCTTTGTAAATCAAGCAATTTGTTTTTTAAAAAGTCTATCTCCTTGATTA
217209


Query: 421    ATACTTCCTCTTGAGTAAACGGAGAAGTCATTAGATCGAAAACGGCCGAGTCTTGATTGA
480
              ||||||||||||||||||||||||||||||||||||||||||||||||||||||||||||
Sbjct: 217210 ATACTTCCTCTTGAGTAAACGGAGAAGTCATTAGATCGAAAACGGCCGAGTCTTGATTGA
217269


Query: 481    TACCAACATTAAAAGAGTATGGGTTATCTGTATAGTCAAAACTTTGAGCGTTAACCAGTT
540
              ||||||||||||||||||||||||||||||||||||||||||||||||||||||||||||
Sbjct: 217270 TACCAACATTAAAAGAGTATGGGTTATCTGTATAGTCAAAACTTTGAGCGTTAACCAGTT
217329


Query: 541    GTAATCGTGTGTTATATTCTCTAATCTGATCCATGGAGGGCACATCTGGACTATCGCTTC
600
              ||||||||||||||||||||||||||||||||||||||||||||||||||||||||||||
Sbjct: 217330 GTAATCGTGTGTTATATTCTCTAATCTGATCCATGGAGGGCACATCTGGACTATCGCTTC
217389


Query: 601    TCACAATAGTGTTTCCATGAACATTACTACTAACGGTACTACTATTCTGACGAGGAGCAG
660
              ||||||||||||||||||||||||||||||||||||||||||||||||||||||||||||
Sbjct: 217390 TCACAATAGTGTTTCCATGAACATTACTACTAACGGTACTACTATTCTGACGAGGAGCAG
217449
```

VIII218008 R

```
Query: 551    TATTATT 545
              |||||||
Sbjct: 217450 TATTATT 217456


Query: 544    ATTGGTGTTATTATTCTGGTAGCTAGCGGCCTTGGTCCACTGCATCGCAGTATCCACAGA
485
              ||||||||||||||||||||||||||||||||||||||||||||||||||||||||||||
Sbjct: 217457 ATTGGTGTTATTATTCTGGTAGCTAGCGGCCTTGGTCCACTGCATCGCAGTATCCACAGA
217516


Query: 484    GGGGTTGAAAGAGAAGTTGGATGCCTCTATCGACGTTTGCATAATCGGATACTGCGTTTG
425
              ||||||||| ||||||||| ||||||||||||||||||||||||||||||||||||||||
Sbjct: 217517 GGGGTTGAACGAGAAGTTGAATGCCTCTATCGACGTTTGCATAATCGGATACTGCGTTTG
217576
```

```
Query: 424    AATCATTACGTTTTTCTGATTTACACCATTTCCCTGACTAAAATGGTTGGAACCTTGGCC
365
              |||||||||||||||||||||||||||| ||||||||||||||||||||||||||||||||
Sbjct: 217577 AATCATTACGTTTTTCTGATTTACACCGTTTCCCTGACTAAAATGGTTGGAACCTTGGCC
217636


Query: 364    ATTGCCGTGCGTGGACATCCCGGACGCAGAGGGCACAGCGACCATCTTACCAGGTCCATC
305
              ||||||||||||||||||||||||||||||||||||||||||||||||||||||||||||
Sbjct: 217637 ATTGCCGTGCGTGGACATCCCGGACGCAGAGGGCACAGCGACCATCTTACCAGGTCCATC
217696


Query: 304    TGGATAAAAACAGTCCGGCTTGTTATACTTGACGCAATTCCCACATATCGGTTTTGCCCT
245
              ||||||||||||||||||||||||||||||||||||||||||||||||||||||||||||
Sbjct: 217697 TGGATAAAAACAGTCCGGCTTGTTATACTTGACGCAATTCCCACATATCGGTTTTGCCCT
217756


Query: 244    GTCGCACCCGATCTTTCTCTTCCTGCATTGGGTGCAAGCAGGCGGCTTCCTCACTTTTCT
185
              ||||||||||||||||||||||||||||||||||||||||||||||||||||||||||||
Sbjct: 217757 GTCGCACCCGATCTTTCTCTTCCTGCATTGGGTGCAAGCAGGCGGCTTCCTCACTTTTCT
217816


Query: 184    CACTTGCATGTCCATCAT 167
              ||||||||||||||||||
Sbjct: 217817 CACTTGCATGTCCATCAT 217834
```

SNPs between DTY3 and S288c

Sequenced interval	Coordinate(s)	SNP in DTY3	SNP in S288c
VIII210848-VIII213868			
	211693	A	G
	211701	A	G
	211825	A	G
	211967	A	G
	211984	G	T
	212279	A	G
	212293	T	C

	212509	A	G
	212536	T	C
	212556	T	C
	212744-212747	4 bp deletion	ATTG
	212751	G	A
	212762	G	C
	212828	G	A
	213097-213101	6 A’s	5 A’s
	213157	T	C
	213283	A	C
	213413	A	G
	213513	T	C
	213536	C	T
	213823	C	T
VIII215871-VIII217834			
	215192	C	T
	216123	T	C
	216235	T	C
	216563	C	T
	216707	C	T
	216814	C	T
	217026	A	G
	217047	T	C
	217626	A	C
	217536	G	A
	217604	A	G
