# Supplementary material for: Structures of Naturally Evolved CUP1 Tandem Arrays in Yeast Indicate That These Arrays Are Generated by Unequal Nonhomologous Recombination
Source: G3 (Bethesda). 2014 Sep 17;4(11):2259–69. doi: 10.1534/g3.114.012922 (PMC4232551; doi:10.1534/g3.114.012922)
Supplement: Supporting Information [file supp_g3.114.012922_FileS1.pdf]

File S1

Supplementary Materials and Methods

Southern analysis

The size of the *CUP1* tandem arrays was estimated by gel electrophoresis of *EcoR*I fragments derived from the various yeast strains. Following electrophoresis, the samples were transferred to Nylon membranes (Roche, Product # 11209272001). The 1 kb hybridization probe included sequences between coordinates 212534 and 213538, containing *CUP1* and flanking sequences within the repeat. The probe was synthesized to contain digoxygenin (DIG)-dUTP. Using the primers CUP1 amp5-2 and CUP1 amp3 (Table S2), we amplified genomic DNA of strain S288c using the PCR DIG probe synthesis kit of Roche (Product No. 11536090910). We used a concentration of DIG-dUTP of 21 μM in addition to the genomic DNA (10 ng), primers, and other constituents of the reaction. DIG-labeled probes for the DNA ladders (Bioline DNA Hyperladders I and VI (discontinued) were generated with DIG-High Prime DNA Labeling and Detection Starter Kit II (Roche, Product No. 11585614910) using the random-priming labeling technique.

Hybridization was performed according to the procedures described in the Roche DIG Application Manual for Filter Hybridization (http://lifescience.roche.com/wcsstore/RASCatalogAssetStore/Articles/05353149001_08.08.pdf). We used 45 μl of the DIG-labeled *CUP1* probe mixture in 25 ml of hybridization buffer (Roche DIG Easy Hyb Granules, Product No. 11796895001). Hybridization was conducted at 42^0 C. overnight, followed by two high-stringency washes done for 15 minutes at 65^0C. Chemiluminescent detection of the probes on the membranes was

done using Anti-Digoxigenin-AP, Fab fragments (Roche, Product No. 11093274910), and the DIG Wash and Block Buffer Set (Roche, Product No. 11585762001). The washing buffer, maleic acid buffer, and detection buffer were prepared as described in the DIG DNA Labeling and Detection Kit Version 19 protocol (https://cssportal.roche.com/LFR_PublicDocs/ras/11093657910_en_19.pdf).

DNA sequencing

For all strains containing repeated *CUP1* genes, we determined the sequence of the repeats, as well as the sequences that connected the repeats to single-copy sequences on the centromere-distal and centromere-proximal sides of the tandem array. The information about the flanking sequences was necessary to determine whether the tandem arrays were in the same chromosomal context on chromosome VIII in all strains. The results of this analysis are presented in Tables S3-S10. Sequences of all primers are in Table S2.

For the analysis of repeats, we first determined the sequences of the PCR fragments generated using the primers F1 and R1'. Since the fragment generated using these primers does not contains about 30 bp that separate the primer binding sites, we also determined the sequences of PCR fragments generated using the primers VIII212300 and VIII213031 that contains the sequences separating F1 and R1'. The specific primers used to sequence the repeats differed for different types of repeats, and are shown in boldface in Tables S3-S9.

The primers used to produce PCR fragments containing the centromere-proximal junctions (*CIC1-CUP1* region) were produced with the following primer pairs: 1) Type 2

(VIII211528 F and R1’), 2) Type 3 (VIII211528 F and R1’), 3) Type 4 (VIII211528 F and R1’), and 4) Type 5 (VIII211528 F and VIII212063 R). The primers used for sequencing are indicated in Tables S3-S9.

The primers used to produce PCR fragments containing the centromere-distal junctions (*CUP1-RSC30* region) were produced with the following primer pairs: 1) Type 2 (VIII213200 F and VIII216603), 2) Type 3 (F1 and VIII213537 R), 3) Type 4 (VIII213601 F and VIII216603 R), and 4) Type 5 (F1 and VIII214195). The primers used for sequencing are indicated in Tables S3-S9.

We also sequenced the *CUP1* region in a copper-sensitive strain DTY3 that contains a single copy of *CUP1*. Three overlapping PCR fragments were sequenced. One fragment (generated using primers VIII210632 F and VIII212063 R) contained the *CIC1* coding sequence and a portion of the *CIC1-CUP1* intergenic sequence. The second fragment (generated using primers VIII211849 F and VIII216603 R) extended from the 3’ region of *CIC1* to the 3’ region of *RSC30*. The third fragment (generated with primers VIII216314 F and VIII218008) contained the 5’ region of *RSC30*. The composite sequence and the primers used to produce the sequence are in Table S10.

Supplementary literature cited

Engel, S. R., F. S. Dietrich, D. G. Fisk, G. Binkley, R. Balakrishnan *et al.*, 2014 The reference genome sequence of *Saccharomyces cerevisiae*: then and now. G3 (Bethesda) 4:389-398.

St. Charles, J., and T. D. Petes, 2013 High-resolution mapping of spontaneous mitotic recombination hotspots on the 1.1 Mb arm of yeast chromosome IV. PLoS Genet. 9:e1003434.

Tamai, K. T., E. B. Gralla, L. M. Ellerby, J. S. Valentine, and D. J. Thiele, 1993 Yeast and mammalian metallothioneins functionally substitute for yeast copper-zinc superoxide dismutase. Proc Natl Acad Sci USA 90: 8013 -8017.

Thomas, B. J., and R. Rothstein, 1989 Elevated recombination rates in transcriptionally active DNA. Cell 56:619-630.

Wei, W., J. H. McCusker, R. W. Hyman, T. Jones, Y. Ning *et al.*, 2007 Genome sequencing and comparative analysis of *Saccharomyces cerevisiae* strain YJM789. Proc. Natl. Acad. Sci. USA 104:12825-12830.
