# Supplementary material for: Structures of Naturally Evolved CUP1 Tandem Arrays in Yeast Indicate That These Arrays Are Generated by Unequal Nonhomologous Recombination
Source: G3 (Bethesda). 2014 Sep 17;4(11):2259–69. doi: 10.1534/g3.114.012922 (PMC4232551; doi:10.1534/g3.114.012922)
Supplement: Supporting Information [file supp_g3.114.012922_TableS1.pdf]

Table S1 Strain genotypes.

Strain name	Genotype (reference)[1]
S288c	*MAT***a** *gal2* (Engel *et al.*, 2014)
W303-1A	*MAT***a** *leu2-3,112 his3-11,15 ura3-1 ade2-1 trp1-1 can1-100Δ::NAT RAD5* (Thomas and Rothstein, 1989)
YZ22	*MAT***a** *leu2-3,112 his3-11,15 ura3-1 ade2-1 trp1-1 can1-100Δ::NAT VIII212898::URA3 RAD5*
JSC10-1	*MAT***a** *leu2-3,112 his3-11,15 ura3-1 ade2-1 trp1-1 can1-100Δ::NAT ho::hisG RAD5* (St. Charles and Petes, 2013)
YJM789	*MATα lys2 gal2 ho::hisG* (Wei *et al.*, 2007)
YJM799	*MATα ura3 gal2 ho::hisG* (provided by J. McCusker, Duke Univ.)
JSC19-1	*MATα ade2-1 ura3 gal2 ho::hisG CAN1Δ::NAT* (St. Charles and Petes, 2013)
DTY3	*MATα trp1-1 leu2-3,112 gal1 ura3-50 his* $cup1^S$ (Tamai *et al.*, 1993)
YJM189	Wild-type diploid from 100-genome strains (Strope *et al.*)
YJM271	Wild-type diploid from 100-genome strains (Strope *et al.*)
YJM456	Wild-type diploid from 100-genome strains (Strope *et al.*)
YJM693	Wild-type diploid from 100-genome strains (Strope *et al.*)
YJM969	Wild-type diploid from 100-genome strains (Strope *et al.*)
YJM972	Wild-type diploid from 100-genome strains (Strope *et al.*)
YJM978	Wild-type diploid from 100-genome strains (Strope *et al.*)
YJM996	Wild-type diploid from 100-genome strains (Strope *et al.*)

YJM1307	Wild-type diploid from 100-genome strains (Strope *et al.*)
YJM1549	Wild-type diploid from 100-genome strains (Strope *et al.*)
YZ22	*MAT***a** *leu2-3,112 his3-11,15 ura3-1 ade2-1 trp1-1 can1-100Δ::NAT VIII212898::URA3 RAD5*

[1]Strains from the 100-genome collection were provided by P. K. Strope, D. A. Skelly, S. G. Kozmin, G. Mahadevan, E. A. Stone, P. M. Magwene, F. S. Dietrich, and J. H. McCusker.
