# Supplementary material for: Structures of Naturally Evolved CUP1 Tandem Arrays in Yeast Indicate That These Arrays Are Generated by Unequal Nonhomologous Recombination
Source: G3 (Bethesda). 2014 Sep 17;4(11):2259–69. doi: 10.1534/g3.114.012922 (PMC4232551; doi:10.1534/g3.114.012922)
Supplement: Supporting Information [file supp_g3.114.012922_TableS2.pdf]

Table S2 Primer names and sequences used in strain constructions and analysis.

Primer name	Sequence
F1	CATTGGCACTCATGACCTTCA
R1	AATAAAGTATCTCCATATGTCGCC
R1'	AATCATGTAGCTGCCCAACGG
cup1 amp5-2	CGAGATGAAATGAATAGCAACGG
cup1 amp5-3	CTCCTTGTCTTGTATCAATTGCAT
cup1 amp3	TTCATTTCCCAGAGCAGCATGAC
VIII212898::URA3 F	TACAAGACAAGGAGTTATTTGCTTCTCTTTTATATG ATTCTGACAATCCATATTGCGTTGGTAGTCTTTTAA TGTGGCTGTGGTTTCAGG
VIII212898::URA3 R	ATTCTTTTGCTGGCATTTCTTCTAGAAGCAAAAAGA GCGATGCGTCTTTTCCGCTGAACCGTTCCAGCAAAG ATTCCCGGGTAATAACTG
VIII211849 F	GGAAATCGCCAATCCTTCCGAATT
VIII216603 R	TATCTCAACTGACCAAACTAGGCG
VIII212300 F	GAGTTGTAAGTTAGGCAAACTAGA
VIII213200 F	ATCATAGAAATCGTTGAAGTTTGC
VIII213031 R	CTGATATCTTAGCCTTGTTACTAG
VIII212063 R	TACATATGCACCGCACTCTATG
VIII210632 F	TGCTTCACCGTTGCGTCAATAA
VIII211528 F	AAAGGTTTACATGAATCAGTTGCC
VIII213234 F	CCCAGATTATCAGATTCCAAATCC

VIII213601 F	GCAATTGAACATTAATCTCCTCAT
VIII211185 F	CCAGTGCAACAGCGGTTAAG
VIII214195 R	GACATTCCTTTAATTGCTAACGAT
VIII213537 R	GAGATGAAATGAATAGCAACGGAAG
VIII210632 F	TGCTTCACCGTTGCGTCAATAA
VIII216314 F	GAAATCTTTTATCTGGAAGCTTAAC
VIII218008 R	TTTGAATATAACCTTGGCGTCCTA
VIII216763 F	TTGGTGGGAAGTTAACTTTGCAA
VIII216859 R	CACGATATCTGCCATATTAATCAG
VIII216894 R	GAGATCATTATCTTTTCAAGTTCTAT
