# Supplementary material for: Structures of Naturally Evolved CUP1 Tandem Arrays in Yeast Indicate That These Arrays Are Generated by Unequal Nonhomologous Recombination
Source: G3 (Bethesda). 2014 Sep 17;4(11):2259–69. doi: 10.1534/g3.114.012922 (PMC4232551; doi:10.1534/g3.114.012922)
Supplement: Supporting Information [file supp_g3.114.012922_TableS3.pdf]

Table S3 Sequence analysis of the *CUP1* repeats (Type 2, 1.8 kb) of YJM189.

In this table, we show genomic sequences of YJM189 in three regions: 1) the sequences that flank the *CUP1* repeats adjacent to *CIC1*, 2) the sequence of the *CUP1* repeat, and 3) the sequences that flank the *CUP1* tandem array adjacent to *RCS30*. The sequences of YJM189 (denoted “Query” below) were compared in a BLAST search with sequences of S288c (denoted “Sbject”). SNPs that distinguish YJM189 and S288c sequences are summarized at the end of the table. The *CUP1* coding sequences are shown in red. The names of the primers used in the sequence analysis are shown in boldface. Additional details about the sequencing are in Supporting Data File S1.

1. *CIC1-CUP1* (VIII211739-212339)

VIII211528 F

```
Query: 182    CTTGATGAACT 192
              |||||||||||
Sbjct: 211739 CTTGATGAACT 211749


Query: 193    TGAAGCTAAAAAGGACAAAATCGAAGAAACCCACGAAGATGACATGGTCACCAT 246
              ||||||||||||||||||||||||||||||||||||||||||||||||||||||
Sbjct: 211750 TGAAGCTAAAAAGGACAAAATCGAAGAAACCCACGAAGATGACATGGTCACCAT 211803


Query: 249    TGATGGTGTACAAGTTCATTTATCTACCTTCAACAAGGGTTTGATGGAAATCGCCAATCC
308
              ||||||||||||||||||||| ||||||||||||||||||||||||||||||||||||||
Sbjct: 211804 TGATGGTGTACAAGTTCATTTGTCTACCTTCAACAAGGGTTTGATGGAAATCGCCAATCC
211863


Query: 309    TTCCGAATTGGGTTCAATTTTCTCTAAACAAATTAACAATGCAAAAAAGAGATCTTCTAG
368
              ||||||||||||||||||||||||||||||||||||||||||||||||||||||||||||
Sbjct: 211864 TTCCGAATTGGGTTCAATTTTCTCTAAACAAATTAACAATGCAAAAAAGAGATCTTCTAG
211923
```

```
Query: 369    CGAGCTTGAAAAAGAATCTAGCGAGTCAGAAGCTGTCAAGAAGGCTAAAAGTTAATTTGT
428
              ||||||||||||||||||||||||||||||||||||||||||||||||||||||||||||
Sbjct: 211924 CGAGCTTGAAAAAGAATCTAGCGAGTCAGAAGCTGTCAAGAAGGCTAAAAGTTAATTTGT
211983


Query: 429    TTCCTCCTTATCTATCTTTTCTCTCATTTTTTTCTTGTGAAGAAAAAAATTTGAATTTCA
488
              ||||||||||||||||||||||||||||||||||||||||||||||||||||||||||||
Sbjct: 211984 TTCCTCCTTATCTATCTTTTCTCTCATTTTTTTCTTGTGAAGAAAAAAATTTGAATTTCA
212043


Query: 489    TAGAGTGCGGTGCATATGTATATATCTATATATGTTTGAAGTGTATATTAAAAATAAAGT
548
              ||||||||||||||||||||||||||||||||||||||||||||||||||||||||||||
Sbjct: 212044 TAGAGTGCGGTGCATATGTATATATCTATATATGTTTGAAGTGTATATTAAAAATAAAGT
212103
```

R1’

```
Query: 522    CATTATTTGAATATTGGTTTCTCGGTCTAAGAGCTTATACGTTTTAGACTG 454
              |||||||||||||||||||||||||||||||||||||||||||||||||||
Sbjct: 212104 CATTATTTGAATATTGGTTTCTCGGTCTAAGAGCTTATACGTTTTAGACTG 212154


Query: 453    ATCTGTTGTACTATCCGCTTCAAATAAATAGATCATTGAAAGTGACGGGGATAACAGCAT
394
              ||||||||||||||||||||||||||||||||||||||||||||||||||||||||||||
Sbjct: 212155 ATCTGTTGTACTATCCGCTTCAAATAAATAGATCATTGAAAGTGACGGGGATAACAGCAT
212214


Query: 393    TTTACCTTTAAAAGACGTTCTCATAATAGATTTTAGGATTAATACATATGCTTTTTTTTT
334
              |||||||||||||||||||||||||||| |||||||||||||||||||||||||||||||
Sbjct: 212215 TTTACCTTTAAAAGACGTTCTCATAATACATTTTAGGATTAATACATATGCTTTTTTTTT
212274


Query: 333    TATTCGAAATCTGGGGATTCTATACAGAGTTGTAAGTTAGGCAAACTAGAATTTGGTAAT
274
               |||||||||||||||||||||||||||||||||||||||||||||||||||||||||||
Sbjct: 212275 -ATTCGAAATCTGGGGATTCTATACAGAGTTGTAAGTTAGGCAAACTAGAATTTGGTAAT
212333


Query: 273    AATATT 268
              ||||||
Sbjct: 212334 AATATT 212339
```

2. *CUP1* Repeat (VIII212039-213867)

R1’

```
Query: 574    TTTCATAGAGTGCGGTGCATATGTATATATCTATATATGTTTGAAGTGTATATTAAAAAT
515
              ||||||||||||||||||||||||||||||||||||||||||||||||||||||||||||
Sbjct: 212039 TTTCATAGAGTGCGGTGCATATGTATATATCTATATATGTTTGAAGTGTATATTAAAAAT
212098


Query: 514    AAAGTCATTATTTGAATATTGGTTTCTCGGTCTAAGAGCTTATACGTTTTAGACTGATCT
455
              ||||||||||||||||||||||||||||||||||||||||||||||||||||||||||||
Sbjct: 212099 AAAGTCATTATTTGAATATTGGTTTCTCGGTCTAAGAGCTTATACGTTTTAGACTGATCT
212158


Query: 454    GTTGTACTATCCGCTTCAAATAAATAGATCATTGAAAGTGACGGGGATAACAGCATTTTA
395
              ||||||||||||||||||||||||||||||||||||||||||||||||||||||||||||
Sbjct: 212159 GTTGTACTATCCGCTTCAAATAAATAGATCATTGAAAGTGACGGGGATAACAGCATTTTA
212218


Query: 394    CCTTTAAAAGACGTTCTCATAATAGATTTTAGGATTAATACATATGCTTTTTTTTTTATT
335
              |||||||||||||||||||||||| ||||||||||||||||||||||||||||||| |||
Sbjct: 212219 CCTTTAAAAGACGTTCTCATAATACATTTTAGGATTAATACATATGCTTTTTTTTT-ATT
212277


Query: 334    CGAAATCTGGGGATTCTATACAGAGTTGTAAGTTAGGCAAACTAGAATTTGGTAATAATA
275
              ||||||||||||||||||||||||||||||||||||||||||||||||||||||||||||
Sbjct: 212278 CGAAATCTGGGGATTCTATACAGAGTTGTAAGTTAGGCAAACTAGAATTTGGTAATAATA
212337


Query: 274    TTTTATTCTTGGGGCGACATATGGAGATACTTTATTTCCTTTTCTTAATTATTAACGTAT
215
              ||||||||||||||||||||||||||||||||||||||||||||||||||||||||||||
Sbjct: 212338 TTTTATTCTTGGGGCGACATATGGAGATACTTTATTTCCTTTTCTTAATTATTAACGTAT
212397


Query: 214    ACCTATAAATTAACAAAGTATCTAAACAAAATACATAAGTGTACTCAAACTGAGTAGAAT
155
              ||||||||||||||||||||||||||||||||||||||||||||||||||||||||||||
Sbjct: 212398 ACCTATAAATTAACAAAGTATCTAAACAAAATACATAAGTGTACTCAAACTGAGTAGAAT
212457


Query: 154    CGTCGATTAAACTTCCTTCTCCTTTTAAAAATTAAAAACAGCAAATAGTTAGATGA 91
              ||||||||||||||||||||||||||||||||||||||||||||||||||||||||
Sbjct: 212458 CGTCGATTAAACTTCCTTCTCCTTTTAAAAATTAAAAACAGCAAATAGTTAGATGA 212513
```

VIII212300 F

```
Query: 187    ATATATTAAAGACTATTCGTTTCATTTCCCAGAGCAGCATGACTTCTTGGTTTCTTCAGA
246
              ||||||||||||||||||||||||||||||||||||||||||||||||||||||||||||
Sbjct: 212514 ATATATTAAAGACTATTCGTTTCATTTCCCAGAGCAGCATGACTTCTTGGTTTCTTCAGA
212573

Query: 247    CTTGTTACCGCAGGGGCATTTGTCGTCGCTGTTACACCCCGTTGGGCAGCTACATGATTT
306
              ||||||||||||||||||||||||||||||||||||||||||||||||||||||||||||
Sbjct: 212574 CTTGTTACCGCAGGGGCATTTGTCGTCGCTGTTACACCCCGTTGGGCAGCTACATGATTT
212633

Query: 307    TTGGCATTGTTCATTATTTTTGCAGCTACCACATTGGCATTGGCACTCATGACCTTCATT
366
              ||||||||||||||||||||||||||||||||||||||||||||||||||||||||||||
Sbjct: 212634 TTGGCATTGTTCATTATTTTTGCAGCTACCACATTGGCATTGGCACTCATGACCTTCATT
212693

Query: 367    TTGGAAGTTAATTAATTCGCTGAACATTTTATGTGATGATTGATTGATTG----TACAGT
422
              ||||||||||||||||||||||||||||||||||||||||||||||||||    ||||||
Sbjct: 212694 TTGGAAGTTAATTAATTCGCTGAACATTTTATGTGATGATTGATTGATTGATTGTACAGT
212753

Query: 423    TTGTTTTTCTTAATATCTATTTCGATGACTTCTATATGATATTGCACTAACAAGAAGATA
482
              ||||||||||||||||||||||||||||||||||||||||||||||||||||||||||||
Sbjct: 212754 TTGTTTTTCTTAATATCTATTTCGATGACTTCTATATGATATTGCACTAACAAGAAGATA
212813

Query: 483    TTATAATGCAATTGATACAAGACAAGGAGTTATTTGCTTCTCTTTTATATGATTCTGACA
542
              ||||||||||||||||||||||||||||||||||||||||||||||||||||||||||||
Sbjct: 212814 TTATAATGCAATTGATACAAGACAAGGAGTTATTTGCTTCTCTTTTATATGATTCTGACA
212873

Query: 543    ATCCATATTGCGTTGGTAGTCTTTTTTGCTGGAACGGTTCAGCGGAAAAGACGCATCGCT
602
              ||||||||||||||||||||||||||||||||||||||||||||||||||||||||||||
Sbjct: 212874 ATCCATATTGCGTTGGTAGTCTTTTTTGCTGGAACGGTTCAGCGGAAAAGACGCATCGCT
212933

Query: 603    CTTTTTGCTTCTAGAAGAAATGCCAGCAAAAGAATCTCTTGACAGTGACTGACAGCAAAA
662
              ||||||||||||||||||||||||||||||||||||||||||||||||||||||||||||
```

```
Sbjct: 212934 CTTTTTGCTTCTAGAAGAAATGCCAGCAAAAGAATCTCTTGACAGTGACTGACAGCAAAA
212993


Query: 663    ATGTCTT 669
              |||||||
Sbjct: 212994 ATGTCTT 213000
```

F1

```
Query: 297    TTTCTAACTAGTAACAAGGCTAAGATATCAGCCTGAAATAAAGGGTGGTGAAGTAATAAT
356
              ||||||||||||||||||||||||||||||||||||||||||||||||||||||||||||
Sbjct: 213001 TTTCTAACTAGTAACAAGGCTAAGATATCAGCCTGAAATAAAGGGTGGTGAAGTAATAAT
213060


Query: 357    TAAATCATCCGTATAAACCTATACACATATATGAGGAAAAATAATACAAAAGTGTTTTAA
416
              ||||||||||||||||||||||||||||||||||||||||||||||||||||||||||||
Sbjct: 213061 TAAATCATCCGTATAAACCTATACACATATATGAGGAAAAATAATACAAAAGTGTTTTAA
213120


Query: 417    ATACAGATACATACATGAACATATGCACGTATAGCGTCCAAATGTCGGTAATGGGATCGG
476
              |||||||||||||||||||||||||||||||||||| |||||||||||||||||||||||
Sbjct: 213121 ATACAGATACATACATGAACATATGCACGTATAGCGCCCAAATGTCGGTAATGGGATCGG
213180


Query: 477    CTTACTAATTATAAAATGCATCATAGAAATCGTTGAAGTTTGCCGTAGTAATACCCAGAT
536
              ||||||||||||||||||||||||||||||||||||||||||||||||||||||||||||
Sbjct: 213181 CTTACTAATTATAAAATGCATCATAGAAATCGTTGAAGTTTGCCGTAGTAATACCCAGAT
213240


Query: 537    TATCAGATTCCAAATCCTTGTCAATAATTATACTCCTTTGGAAAACTTCTCTTTCCATTA
596
              |||||||||||||||||||||||||||||||||||||||||| |||||||||||||||||
Sbjct: 213241 TATCAGATTCCAAATCCTTGTCAATAATTATACTCCTTTGGACAACTTCTCTTTCCATTA
213300


Query: 597    AAAAATCTGAAATCTCCTTAAATTTTAAATAGATTCTGTTCAGTTCACTAACGGGGAATT
656
              ||||||||||||||||||||||||||||||||||||||||||||||||||||||||||||
Sbjct: 213301 AAAAATCTGAAATCTCCTTAAATTTTAAATAGATTCTGTTCAGTTCACTAACGGGGAATT
213360
```

VIII213200 F

```
Query: 116    TCAAGAGAACAT 127
              ||||||||||||
Sbjct: 213361 TCAAGAGAACAT 213372


Query: 128    TTTTGTTCTTCGCCGACTGACTATAATCTGTAACATTATTGTTATCAGAGTTTCTCGCAA
187
              ||||||||||||||||||||||||||||||||||||||||||||||||||||||||||||
Sbjct: 213373 TTTTGTTCTTCGCCGACTGACTATAATCTGTAACATTATTGTTATCAGAGTTTCTCGCAA
213432


Query: 188    AATTTTGTTTTTTCTTGCTAAATCTCAGCATATATTTAATCAGATTCAAAACCTTGTTGA
247
              ||||||||||||||||||||||||||||||||||||||||||||||||||||||||||||
Sbjct: 213433 AATTTTGTTTTTTCTTGCTAAATCTCAGCATATATTTAATCAGATTCAAAACCTTGTTGA
213492


Query: 248    AACCTTTAATAGATTTGAAATTTCCGTTGCTATTCATTTCATCTCGTAAAAAGGATACGA
307
              |||||||||||||||||||| |||||||||||||||||||||||||||||||||||||||
Sbjct: 213493 AACCTTTAATAGATTTGAAACTTCCGTTGCTATTCATTTCATCTCGTAAAAAGGATACGA
213552


Query: 308    TAATTTCTATTTTTTTTAAAATTTCCAAAATCTTGTCATGAATCAATAGCAATTGAACAT
367
              ||||||||||||||||||||||||||||||||||||||||||||||||||||||||||||
Sbjct: 213553 TAATTTCTATTTTTTTTAAAATTTCCAAAATCTTGTCATGAATCAATAGCAATTGAACAT
213612



Query: 368    TAATCTCCTCATTTGAAAGATTTTTGTAAAATTCGTCATATAATATTACTTCACAACGTT
427
              ||||||||||||||||||||||||||||||||||||||||||||||||||||||||||||
Sbjct: 213613 TAATCTCCTCATTTGAAAGATTTTTGTAAAATTCGTCATATAATATTACTTCACAACGTT
213672


Query: 428    GGAAAATAGCAAATGTGATTGCTATAAAATTCTGTAAGATTTCAATAAAATGATTTGCGA
487
              ||||||||||||||||||||||||||||||||||||||||||||||||||||||||||||
Sbjct: 213673 GGAAAATAGCAAATGTGATTGCTATAAAATTCTGTAAGATTTCAATAAAATGATTTGCGA
213732


Query: 488    ATAAAAATTCTTTACCATTAGAATGAAAGCGATTATTGCCGCTTGAAAATGACTTTATCG
547
              ||||||||||||||||||||||||||||||||||||||||||||||||||||||||||||
```

```
Sbjct: 213733 ATAAAAATTCTTTACCATTAGAATGAAAGCGATTATTGCCGCTTGAAAATGACTTTATCG
213792
```

R1’

```
Query: 649    ACTTTATGGGGAAGATAAAATTAA 626
              ||||||||||||||||||||||||
Sbjct: 213793 ACTTTATGGGGAAGATAAAATTAA 213816


Query: 625    ATGTTATTGAGTAAAAAATGTGCATATTAGAAATAATTTTCATCAGATCCT 561
              |||||||||||||||||||||||||||||||||||||||||||||||||||
Sbjct: 213817 ATGTTATTGAGTAAAAAATGTGCATATTAGAAATAATTTTCATCAGATCCT 213867
```

3. *CUP1-RSC30* (VIII213567-214167)

VIII213234 F

```
Query: 304    TTTAAAATTTCCAAAATCTTGTCATGAATCAATAGCAATTGAACATTAATCTCCTCATTT
363
              ||||||||||||||||||||||||||||||||||||||||||||||||||||||||||||
Sbjct: 213567 TTTAAAATTTCCAAAATCTTGTCATGAATCAATAGCAATTGAACATTAATCTCCTCATTT
213626


Query: 364    GAAAGATTTTTGTAAAATTCGTCATATAATATTACTTCACAACGTTGGAAAATAGCAAAT
423
              ||||||||||||||||||||||||||||||||||||||||||||||||||||||||||||
Sbjct: 213627 GAAAGATTTTTGTAAAATTCGTCATATAATATTACTTCACAACGTTGGAAAATAGCAAAT
213686


Query: 424    GTGATTGCTATAAAATTCTGTAAGATTTCAATAAAATGATTTGCGAATAAAAATTCTTTA
483
              ||||||||||||||||||||||||||||||||||||||||||||||||||||||||||||
Sbjct: 213687 GTGATTGCTATAAAATTCTGTAAGATTTCAATAAAATGATTTGCGAATAAAAATTCTTTA
213746


Query: 484    CCATTAGAATGAAAGCGATTATTGCCGCTTGAAAATGACTTTATCGACTTTATGGGGAAG
543
              ||||||||||||||||||||||||||||||||||||||||||||||||||||||||||||
Sbjct: 213747 CCATTAGAATGAAAGCGATTATTGCCGCTTGAAAATGACTTTATCGACTTTATGGGGAAG
213806


Query: 544    ATAAAATTAAATGTTACTGAGTAAAAAATGTGCATATTAGAAATAATTTTCATCAGATCC
603
              |||||||||||||||| |||||||||||||||||||||||||||||||||||||||||||
Sbjct: 213807 ATAAAATTAAATGTTATTGAGTAAAAAATGTGCATATTAGAAATAATTTTCATCAGATCC
213866
```

```
Query: 604    TTTGCACATCTTTCAGAGTTCGAGGTCTTATTGTTGTTAGAAGAATGTTGAACTGCCATG
663
              ||||||||||||||||||||||||||||||||||||||||||||||||||||||||||||
Sbjct: 213867 TTTGCACATCTTTCAGAGTTCGAGGTCTTATTGTTGTTAGAAGAATGTTGAACTGCCATG
213926


Query: 664    GACAAAGAGGATTCGTTTTGAACAAAAAGGAAAAAATTTGTATAAACAATGGTATTGATA
723
              ||||||||||||||||||||||||||||||||||||||||||||||||||||||||||||
Sbjct: 213927 GACAAAGAGGATTCGTTTTGAACAAAAAGGAAAAAATTTGTATAAACAATGGTATTGATA
213986
```

VIII216603 R

```
Query: 588    AAAT 585
              ||||
Sbjct: 213987 AAAT 213990


Query: 584    TTAAAGTGTCTTTCCATTCTTTTCTGACTTCGTTGTCATGAAAATATAAGTCTACTGTAT
525
              ||||||||||||||||||||||||||||||||||||||||||||||||||||||||||||
Sbjct: 213991 TTAAAGTGTCTTTCCATTCTTTTCTGACTTCGTTGTCATGAAAATATAAGTCTACTGTAT
214050


Query: 524    TACTCACGCCCATAGTCAAGGTTTCTAACAGACTTTCAATTTTGGTTAAATTTACTGGCA
465
              ||||||||||||||||||||||||||||||||||||||||||||||||||||||||||||
Sbjct: 214051 TACTCACGCCCATAGTCAAGGTTTCTAACAGACTTTCAATTTTGGTTAAATTTACTGGCA
214110


Query: 464    AGTAGAAAGGAACATCTTGCAGAATATTTATCAATTTTGCTTGCGTTTCCAGTAATT 402
              |||||||||||||| ||||||||||||||||||||||||||||||||||||||||||
Sbjct: 214111 AGTAGAAAGGAACACCTTGCAGAATATTTATCAATTTTGCTTGCGTTTCCAGTAATT
214167
```

SNPs between YJM189 and S288c

Sequenced interval	Coordinate(s)	SNP in YJM189	SNP in S288c
CIC1-CUP1 VIII211739-212339			
	211825	A	G
	212243	G	C
	212266-212274	10 T’s	9 T’s
CUP1 repeat VIII212039-213867			

	212243	G	C
	212266-212274	10 T's	9 T's
	212744-212747	4 bp deletion	ATTG
	213157	T	C
	213283	A	C
	213513	T	C
CUP1-RSC30<br>VIII213567-214167			
	213823	C	T
	214125	T	C
