# Supplementary material for: Structures of Naturally Evolved CUP1 Tandem Arrays in Yeast Indicate That These Arrays Are Generated by Unequal Nonhomologous Recombination
Source: G3 (Bethesda). 2014 Sep 17;4(11):2259–69. doi: 10.1534/g3.114.012922 (PMC4232551; doi:10.1534/g3.114.012922)
Supplement: Supporting Information [file supp_g3.114.012922_TableS4.pdf]

Table S4 Sequence analysis of the *CUP1* repeats (Type 2, 1.8 kb) of YJM996.

In this table, we show genomic sequences of YJM996 in three regions: 1) the sequences that flank the *CUP1* repeats adjacent to *CIC1*, 2) the sequence of the *CUP1* repeat, and 3) the sequences that flank the *CUP1* tandem array adjacent to *RCS30*. The sequences of YJM189 (denoted “Query” below) were compared in a BLAST search with sequences of S288c (denoted “Sbject”). SNPs that distinguish YJM189 and S288c sequences are summarized at the end of the table. The *CUP1* coding sequences are shown in red. The names of the primers used in the sequence analysis are shown in boldface. Additional details about the sequencing are in Supporting Data File S1.

1. *CIC1-CUP1* (VIII211739-212339)

R1’

```
Query: 863    CTTGATGAACTTGAAGCTAAA 843
              |||||||||||||||||||||
Sbjct: 211739 CTTGATGAACTTGAAGCTAAA 211759


Query: 842    AAGGACAAAATCGAAGAAACCCACGAAGATGACATGGTCACCATTGATGGTGTACAAGTT
783
              ||||||||||||||||||||||||||||||||||||||||||||||||||||||||||||
Sbjct: 211760 AAGGACAAAATCGAAGAAACCCACGAAGATGACATGGTCACCATTGATGGTGTACAAGTT
211819


Query: 782    CATTTATCTACCTTCAACAAGGGTTTGATGGAAATCGCCAATCCTTCCGAATTGGGTTCA
723
              ||||| ||||||||||||||||||||||||||||||||||||||||||||||||||||||
Sbjct: 211820 CATTTGTCTACCTTCAACAAGGGTTTGATGGAAATCGCCAATCCTTCCGAATTGGGTTCA
211879


Query: 722    ATTTTCTCTAAACAAATTAACAATGCAAAAAAGAGATCTTCTAGCGAGCTTGAAAAAGAA
663
              ||||||||||||||||||||||||||||||||||||||||||||||||||||||||||||
Sbjct: 211880 ATTTTCTCTAAACAAATTAACAATGCAAAAAAGAGATCTTCTAGCGAGCTTGAAAAAGAA
211939
```

```
Query: 662    TCTAGCGAGTCAGAAGCTGTCAAGAAGGCTAAAAGTTAATTTGTTTCCTCCTTATCTATC
603
              ||||||||||||||||||||||||||||||||||||||||||||||||||||||||||||
Sbjct: 211940 TCTAGCGAGTCAGAAGCTGTCAAGAAGGCTAAAAGTTAATTTGTTTCCTCCTTATCTATC
211999


Query: 602    TTTTCTCTCATTTTTTTCTTGTGAAGAAAAAAATTTGAATTTCATAGAGTGCGGTGCATA
543
              ||||||||||||||||||||||||||||||||||||||||||||||||||||||||||||
Sbjct: 212000 TTTTCTCTCATTTTTTTCTTGTGAAGAAAAAAATTTGAATTTCATAGAGTGCGGTGCATA
212059


Query: 542    TGTATATATCTATATATGTTTGAAGTGTATATTAAAAATAAAGTCATTATTTGAATATTG
483
              ||||||||||||||||||||||||||||||||||||||||||||||||||||||||||||
Sbjct: 212060 TGTATATATCTATATATGTTTGAAGTGTATATTAAAAATAAAGTCATTATTTGAATATTG
212119


Query: 482    GTTTCTCGGTCTAAGAGCTTATACGTTTTAGACTGATCTGTTGTACTATCCGCTTCAAAT
423
              ||||||||||||||||||||||||||||||||||||||||||||||||||||||||||||
Sbjct: 212120 GTTTCTCGGTCTAAGAGCTTATACGTTTTAGACTGATCTGTTGTACTATCCGCTTCAAAT
212179


Query: 422    AAATAGATCATTGAAAGTGACGGGGATAACAGCATTTTACCTTTAAAAGACGTTCTCATA
363
              ||||||||||||||||||||||||||||||||||||||||||||||||||||||||||||
Sbjct: 212180 AAATAGATCATTGAAAGTGACGGGGATAACAGCATTTTACCTTTAAAAGACGTTCTCATA
212239


Query: 362    ATAGATTTTAGGATTAATACATATGCTTTTTTTTTTATTCGAAATCTGGGGATTCTATAC
303
              ||| |||||||||||||||||||||||||||||||| |||||||||||||||||||||||
Sbjct: 212240 ATACATTTTAGGATTAATACATATGCTTTTTTTTT-ATTCGAAATCTGGGGATTCTATAC
212298


Query: 302    AGAGTTGTAAGTTAGGCAAACTAGAATTTGGTAATAATATT 252
              |||||||||||||||||||||||||||||||||||||||||
Sbjct: 212299 AGAGTTGTAAGTTAGGCAAACTAGAATTTGGTAATAATATT 212339
```

2. *CUP1* repeat (VIII212039-213867)

R1’

```
Query: 572    TTTCATAGAGTGCGGTGCATATGTATATATCTATATATGTTTGAAGTGTATATTAAAAAT
513
              ||||||||||||||||||||||||||||||||||||||||||||||||||||||||||||
```

```
Sbjct: 212039 TTTCATAGAGTGCGGTGCATATGTATATATCTATATATGTTTGAAGTGTATATTAAAAAT
212098


Query: 512    AAAGTCATTATTTGAATATTGGTTTCTCGGTCTAAGAGCTTATACGTTTTAGACTGATCT
453
              ||||||||||||||||||||||||||||||||||||||||||||||||||||||||||||
Sbjct: 212099 AAAGTCATTATTTGAATATTGGTTTCTCGGTCTAAGAGCTTATACGTTTTAGACTGATCT
212158


Query: 452    GTTGTACTATCCGCTTCAAATAAATAGATCATTGAAAGTGACGGGGATAACAGCATTTTA
393
              ||||||||||||||||||||||||||||||||||||||||||||||||||||||||||||
Sbjct: 212159 GTTGTACTATCCGCTTCAAATAAATAGATCATTGAAAGTGACGGGGATAACAGCATTTTA
212218


Query: 392    CCTTTAAAAGACGTTCTCATAATAGATTTTAGGATTAATACATATGCTTTTTTTTTTATT
333
              |||||||||||||||||||||||| ||||||||||||||||||||||||||||||| |||
Sbjct: 212219 CCTTTAAAAGACGTTCTCATAATACATTTTAGGATTAATACATATGCTTTTTTTTT-ATT
212277


Query: 332    CGAAATCTGGGGATTCTATACAGAGTTGTAAGTTAGGCAAACTAGAATTTGGTAATAATA
273
              ||||||||||||||||||||||||||||||||||||||||||||||||||||||||||||
Sbjct: 212278 CGAAATCTGGGGATTCTATACAGAGTTGTAAGTTAGGCAAACTAGAATTTGGTAATAATA
212337


Query: 272    TTTTATTCTTGGGGCGACATATGGAGATACTTTATTTCCTTTTCTTAATTATTAACGTAT
213
              ||||||||||||||||||||||||||||||||||||||||||||||||||||||||||||
Sbjct: 212338 TTTTATTCTTGGGGCGACATATGGAGATACTTTATTTCCTTTTCTTAATTATTAACGTAT
212397


Query: 212    ACCTATAAATTAACAAAGTATCTAAACAAAATACATAAGTGTACTCAAACTGAGTAGAAT
153
              ||||||||||||||||||||||||||||||||||||||||||||||||||||||||||||
Sbjct: 212398 ACCTATAAATTAACAAAGTATCTAAACAAAATACATAAGTGTACTCAAACTGAGTAGAAT
212457
```

VIII212300 F

```
Query: 131    CGTCGATTAAACTTCCTTCTCCTTTTAAAAATTAAAAACAGCAAATAGTTAGATGA 186
              ||||||||||||||||||||||||||||||||||||||||||||||||||||||||
Sbjct: 212458 CGTCGATTAAACTTCCTTCTCCTTTTAAAAATTAAAAACAGCAAATAGTTAGATGA 212513


Query: 187    ATATATTAAAGACTATTCGTTTCATTTCCCAGAGCAGCATGACTTCTTGGTTTCTTCAGA
246
```

```
                  ||||||||||||||||||||||||||||||||||||||||||||||||||||||||||||
Sbjct: 212514 ATATATTAAAGACTATTCGTTTCATTTCCCAGAGCAGCATGACTTCTTGGTTTCTTCAGA
212573


Query: 247     CTTGTTACCGCAGGGGCATTTGTCGTCGCTGTTACACCCCGTTGGGCAGCTACATGATTT
306
                ||||||||||||||||||||||||||||||||||||||||||||||||||||||||||||
Sbjct: 212574 CTTGTTACCGCAGGGGCATTTGTCGTCGCTGTTACACCCCGTTGGGCAGCTACATGATTT
212633


Query: 307     TTGGCATTGTTCATTATTTTTGCAGCTACCACATTGGCATTGGCACTCATGACCTTCATT
366
                ||||||||||||||||||||||||||||||||||||||||||||||||||||||||||||
Sbjct: 212634 TTGGCATTGTTCATTATTTTTGCAGCTACCACATTGGCATTGGCACTCATGACCTTCATT
212693


Query: 367     TTGGAAGTTAATTAATTCGCTGAACATTTTATGTGATGATTGATTGATTG----TACAGT
422
                ||||||||||||||||||||||||||||||||||||||||||||||||||    ||||||
Sbjct: 212694 TTGGAAGTTAATTAATTCGCTGAACATTTTATGTGATGATTGATTGATTGATTGTACAGT
212753


Query: 423     TTGTTTTTCTTAATATCTATTTCGATGACTTCTATATGATATTGCACTAACAAGAAGATA
482
                ||||||||||||||||||||||||||||||||||||||||||||||||||||||||||||
Sbjct: 212754 TTGTTTTTCTTAATATCTATTTCGATGACTTCTATATGATATTGCACTAACAAGAAGATA
212813


Query: 483     TTATAATGCAATTGATACAAGACAAGGAGTTATTTGCTTCTCTTTTATATGATTCTGACA
542
                ||||||||||||||||||||||||||||||||||||||||||||||||||||||||||||
Sbjct: 212814 TTATAATGCAATTGATACAAGACAAGGAGTTATTTGCTTCTCTTTTATATGATTCTGACA
212873
```

F1

```
Query: 171     ATCCATATTGCGTT 184
                ||||||||||||||
Sbjct: 212874 ATCCATATTGCGTT 212887


Query: 185     GGTAGTCTTTTTTGCTGGAACGGTTCAGCGGAAAAGACGCATCGCTCTTTTTGCTTCTAG
244
                ||||||||||||||||||||||||||||||||||||||||||||||||||||||||||||
Sbjct: 212888 GGTAGTCTTTTTTGCTGGAACGGTTCAGCGGAAAAGACGCATCGCTCTTTTTGCTTCTAG
212947


Query: 245     AAGAAATGCCAGCAAAAGAATCTCTTGACAGTGACTGACAGCAAAAATGTCTTTTTCTAA
304
                ||||||||||||||||||||||||||||||||||||||||||||||||||||||||||||
```

```
Sbjct: 212948 AAGAAATGCCAGCAAAAGAATCTCTTGACAGTGACTGACAGCAAAAATGTCTTTTTCTAA
213007


Query: 305    CTAGTAACAAGGCTAAGATATCAGCCTGAAATAAAGGGTGGTGAAGTAATAATTAAATCA
364
              ||||||||||||||||||||||||||||||||||||||||||||||||||||||||||||
Sbjct: 213008 CTAGTAACAAGGCTAAGATATCAGCCTGAAATAAAGGGTGGTGAAGTAATAATTAAATCA
213067


Query: 365    TCCGTATAAACCTATACACATATATGAGGAAAAATAATACAAAAGTGTTTTAAATACAGA
424
              ||||||||||||||||||||||||||||||||||||||||||||||||||||||||||||
Sbjct: 213068 TCCGTATAAACCTATACACATATATGAGGAAAAATAATACAAAAGTGTTTTAAATACAGA
213127


Query: 425    TACATACATGAACATATGCACGTATAGCGTCCAAATGTCGGTAATGGGATCGGCTTACTA
484
              ||||||||||||||||||||||||||||| ||||||||||||||||||||||||||||||
Sbjct: 213128 TACATACATGAACATATGCACGTATAGCGCCCAAATGTCGGTAATGGGATCGGCTTACTA
213187


Query: 485    ATTATAAAATGCATCATAGAAATCGTTGAAGTTTGCCGTAGTAATACCCAGATTATCAGA
544
              ||||||||||||||||||||||||||||||||||||||||||||||||||||||||||||
Sbjct: 213188 ATTATAAAATGCATCATAGAAATCGTTGAAGTTTGCCGTAGTAATACCCAGATTATCAGA
213247


Query: 545    TTCCAAATCCTTGTCAATAATTATACTCCTTTGGAAAACTTCTCTTTCCATTAAAAAATC
604
              ||||||||||||||||||||||||||||||||||| ||||||||||||||||||||||||
Sbjct: 213248 TTCCAAATCCTTGTCAATAATTATACTCCTTTGGACAACTTCTCTTTCCATTAAAAAATC
213307
```

VIII213200 F

```
Query: 84     TGAAATCTCCTTAAATTTTAAATAGATTCTGTTCAGTTCACTAAC 128
              |||||||||||||||||||||||||||||||||||||||||||||
Sbjct: 213308 TGAAATCTCCTTAAATTTTAAATAGATTCTGTTCAGTTCACTAAC 213352


Query: 129    GGGGAATTTCAAGAGAACATTTTTGTTCTTCGCCGACTGACTATAATCTGTAACATTATT
188
              ||||||||||||||||||||||||||||||||||||||||||||||||||||||||||||
Sbjct: 213353 GGGGAATTTCAAGAGAACATTTTTGTTCTTCGCCGACTGACTATAATCTGTAACATTATT
213412


Query: 189    GTTATCAGAGTTTCTCGCAAAATTTTGTTTTTTCTTGCTAAATCTCAGCATATATTTAAT
248
              ||||||||||||||||||||||||||||||||||||||||||||||||||||||||||||
```

```
Sbjct: 213413 GTTATCAGAGTTTCTCGCAAAATTTTGTTTTTTCTTGCTAAATCTCAGCATATATTTAAT
213472


Query: 249    CAGATTCAAAACCTTGTTGAAACCTTTAATAGATTTGAAATTTCCGTTGCTATTCATTTC
308
              |||||||||||||||||||||||||||||||||||||||| |||||||||||||||||||
Sbjct: 213473 CAGATTCAAAACCTTGTTGAAACCTTTAATAGATTTGAAACTTCCGTTGCTATTCATTTC
213532


Query: 309    ATCTCGTAAAAAGGATACGATAATTTCTATTTTTTTTAAAATTTCCAAAATCTTGTCATG
368
              ||||||||||||||||||||||||||||||||||||||||||||||||||||||||||||
Sbjct: 213533 ATCTCGTAAAAAGGATACGATAATTTCTATTTTTTTTAAAATTTCCAAAATCTTGTCATG
213592


Query: 369    AATCAATAGCAATTGAACATTAATCTCCTCATTTGAAAGATTTTTGTAAAATTCGTCATA
428
              ||||||||||||||||||||||||||||||||||||||||||||||||||||||||||||
Sbjct: 213593 AATCAATAGCAATTGAACATTAATCTCCTCATTTGAAAGATTTTTGTAAAATTCGTCATA
213652


Query: 429    TAATATTACTTCACAACGTTGGAAAATAGCAAATGTGATTGCTATAAAATTCTGTAAGAT
488
              ||||||||||||||||||||||||||||||||||||||||||||||||||||||||||||
Sbjct: 213653 TAATATTACTTCACAACGTTGGAAAATAGCAAATGTGATTGCTATAAAATTCTGTAAGAT
213712


Query: 489    TTCAATAAAATGATTTGCGAATAAAAATTCTTTACCATTAGAATGAAAGCGATTATTGCC
548
              ||||||||||||||||||||||||||||||||||||||||||||||||||||||||||||
Sbjct: 213713 TTCAATAAAATGATTTGCGAATAAAAATTCTTTACCATTAGAATGAAAGCGATTATTGCC
213772


Query: 549    GCTTGAAAATGACTTTATCGACTTTATGGGGAAGATAAAATTAAATGTTATTGAGTAAAA
608
              ||||||||||||||||||||||||||||||||||||||||||||||||||||||||||||
Sbjct: 213773 GCTTGAAAATGACTTTATCGACTTTATGGGGAAGATAAAATTAAATGTTATTGAGTAAAA
213832


Query: 609    AATGTGCATATTAGAAATAATTTTCATCAGATCCT 643
              |||||||||||||||||||||||||||||||||||
Sbjct: 213833 AATGTGCATATTAGAAATAATTTTCATCAGATCCT 213867
```

3. *CUP1-RSC30* (VIII213567-214167)

VIII213200 F

```
Query: 342    TTTAAAATTTCCAAAATCTTGT 364
              ||||||||||||||||||||||
Sbjct: 213567 TTTAAAATTTCCAAAATCTTGT 213588


Query: 365    CATGAATCAATAGCAATTGAACATTAATCTCCTCATTTGAAAGATTTTTGTAAAATTCGT
424
              ||||||||||||||||||||||||||||||||||||||||||||||||||||||||||||
Sbjct: 213589 CATGAATCAATAGCAATTGAACATTAATCTCCTCATTTGAAAGATTTTTGTAAAATTCGT
213648


Query: 425    CATATAATATTACTTCACAACGTTGGAAAATAGCAAATGTGATTGCTATAAAATTCTGTA
484
              ||||||||||||||||||||||||||||||||||||||||||||||||||||||||||||
Sbjct: 213649 CATATAATATTACTTCACAACGTTGGAAAATAGCAAATGTGATTGCTATAAAATTCTGTA
213708


Query: 485    AGATTTCAATAAAATGATTTGCGAATAAAAATTCTTTACCATTAGAATGAAAGCGATTAT
544
              ||||||||||||||||||||||||||||||||||||||||||||||||||||||||||||
Sbjct: 213709 AGATTTCAATAAAATGATTTGCGAATAAAAATTCTTTACCATTAGAATGAAAGCGATTAT
213768


Query: 545    TGCCGCTTGAAAATGACTTTATCGACTTTATGGGGAAGATAAAATTAAATGTTATTGAGT
604
              ||||||||||||||||||||||||||||||||||||||||||||||||||||||||||||
Sbjct: 213769 TGCCGCTTGAAAATGACTTTATCGACTTTATGGGGAAGATAAAATTAAATGTTATTGAGT
213828


Query: 605    AAAAAATGTGCATATTAGAAATAATTTTCATCAGATCCTTTGCACATCTTTCAGAGTTCG
664
              ||||||||||||||||||||||||||||||||||||||||||||||||||||||||||||
Sbjct: 213829 AAAAAATGTGCATATTAGAAATAATTTTCATCAGATCCTTTGCACATCTTTCAGAGTTCG
213888


Query: 665    AGGTCTTATTGTTGTTAGAAGAATGTTGAACTGCCATGGACAAAGAGGATTCGTTTTGAA
724
              ||||||||||||||||||||||||||||||||||||||||||||||||||||||||||||
Sbjct: 213889 AGGTCTTATTGTTGTTAGAAGAATGTTGAACTGCCATGGACAAAGAGGATTCGTTTTGAA
213948
```

VIII216603 R

```
Query: 630    CAAAAAGGA 622
              |||||||||
Sbjct: 213949 CAAAAAGGA 213957
```

```
Query: 621    AAAAATTTGTATAAACAATGGTATTGATAAAATTTAAAGTGTCTTTCCATTCTTTTCTGA
562
              ||||||||||||||||||||||||||||||||||||||||||||||||||||||||||||
Sbjct: 213958 AAAAATTTGTATAAACAATGGTATTGATAAAATTTAAAGTGTCTTTCCATTCTTTTCTGA
214017


Query: 561    CTTCGTTGTCATGAAAATATAAGTCTACTGTATTACTCACGCCCATAGTCAAGGTTTCTA
502
              ||||||||||||||||||||||||||||||||||||||||||||||||||||||||||||
Sbjct: 214018 CTTCGTTGTCATGAAAATATAAGTCTACTGTATTACTCACGCCCATAGTCAAGGTTTCTA
214077


Query: 501    ACAGACTTTCAATTTTGGTTAAATTTACTGGCAAGTAGAAAGGAACATCTTGCAGAATAT
442
              ||||||||||||||||||||||||||||||||||||||||||||||| ||||||||||||
Sbjct: 214078 ACAGACTTTCAATTTTGGTTAAATTTACTGGCAAGTAGAAAGGAACACCTTGCAGAATAT
214137


Query: 441    TTATCAATTTTGCTTGCGTTTCCGGTAATTTTAAATCGTTAGCAATTAAA 392
              ||||||||||||||||||||||| ||||||||||||||||||||||||||
Sbjct: 214138 TTATCAATTTTGCTTGCGTTTCCAGTAATTTTAAATCGTTAGCAATTAAA 214187
```

SNPs between YJM996 and S288c

Sequenced interval	Coordinate(s)	SNP in YJM996	SNP in S288c
CIC1-CUP1 VIII211739-212339			
	211825	A	G
	212243	G	C
	212266-212274	10 T's	9 T's
CUP1 repeat VIII212039-213867			
	212243	G	C
	212266-212274	10 T's	9 T's
	212744-212747	4 bp deletion	ATTG
	213157	T	C
	213283	A	C
	213513	T	C
CUP1-RSC30 VIII213567-214167			
	214125	T	C
	214161	G	A
