# Supplementary material for: Structures of Naturally Evolved CUP1 Tandem Arrays in Yeast Indicate That These Arrays Are Generated by Unequal Nonhomologous Recombination
Source: G3 (Bethesda). 2014 Sep 17;4(11):2259–69. doi: 10.1534/g3.114.012922 (PMC4232551; doi:10.1534/g3.114.012922)
Supplement: Supporting Information [file supp_g3.114.012922_TableS5.pdf]

Table S5 Sequence analysis of the *CUP1* repeats (Type 3, 1.2 kb) of YJM789.

In this table, we show genomic sequences of YJM789 in three regions: 1) the sequences that flank the *CUP1* repeats adjacent to *CIC1*, 2) the sequence of the *CUP1* repeat, and 3) the sequences that flank the *CUP1* tandem array adjacent to *RCS30*. The sequences of YJM789 (denoted “Query” below) were compared in a BLAST search with sequences of S288c (denoted “Sbject”). SNPs that distinguish YJM789 and S288c sequences are summarized at the end of the table. The *CUP1* coding sequences are shown in red. The names of the primers used in the sequence analysis are shown in boldface. Additional details about the sequencing are in Supporting Data File S1.

1. *CIC1-CUP1* (VIII211730-212330)

VIII211528 F

```
Query: 167    CAGGACGTTCTTGAT 181
              |||||||||||||||
Sbjct: 211730 CAGGACGTTCTTGAT 211744


Query: 182    GAACTTGAAGCTAAAAAGGACAAAATCGAAGAAACCCACGAAGATGACATGGTCACCATT
241
              ||||||||||||||||||||||||||||||||||||||||||||||||||||||||||||
Sbjct: 211745 GAACTTGAAGCTAAAAAGGACAAAATCGAAGAAACCCACGAAGATGACATGGTCACCATT
211804


Query: 242    GATGGTGTACAAGTTCATTTGTCTACCTTCAACAAGGGTTTGATGGAAATCGCCAATCCT
301
              ||||||||||||||||||||||||||||||||||||||||||||||||||||||||||||
Sbjct: 211805 GATGGTGTACAAGTTCATTTGTCTACCTTCAACAAGGGTTTGATGGAAATCGCCAATCCT
211864


Query: 302    TCCGAATTGGGTTCAATTTTCTCTAAACAAATTAACAATGCAAAAAAGAGATCTTCTAGC
361
              ||||||||||||||||||||||||||||||||||||||||||||||||||||||||||||
Sbjct: 211865 TCCGAATTGGGTTCAATTTTCTCTAAACAAATTAACAATGCAAAAAAGAGATCTTCTAGC
211924


Query: 362    GAGCTTGAAAAAGAATCTAGCGAGTCAGAAGCTGTCAAGAAGGCTAAAAGTTAATTTGTT
421
```

```
             ||||||||||||||||||||||||||||||||||||||||||||||||||||||||||||
Sbjct: 211925 GAGCTTGAAAAAGAATCTAGCGAGTCAGAAGCTGTCAAGAAGGCTAAAAGTTAATTTGTT
211984

Query: 422    TCCTCCTTATCTATCTTTTCTCTCATTTTTTTCTTGTGAAGAAAAAAATTTGAATTTCAT
481
             ||||||||||||||||||||||||||||||||||||||||||||||||||||||||||||
Sbjct: 211985 TCCTCCTTATCTATCTTTTCTCTCATTTTTTTCTTGTGAAGAAAAAAATTTGAATTTCAT
212044

Query: 482    AGAGTGCGGTGCATATGTATATATCTATATATGTTTGAAGTGTATATTAAAAATAAAGTC
541
             ||||||||||||||||||||||||||||||||||||||||||||||||||||||||||||
Sbjct: 212045 AGAGTGCGGTGCATATGTATATATCTATATATGTTTGAAGTGTATATTAAAAATAAAGTC
212104


Query: 542    ATTATTTGAATATTGGTTTCTCGGTCTAAGAGCTTATACGTTTTAGACTGATCTGTTGTA
601
             ||||||||||||||||||||||||||||||||||||||||||||||||||||||||||||
Sbjct: 212105 ATTATTTGAATATTGGTTTCTCGGTCTAAGAGCTTATACGTTTTAGACTGATCTGTTGTA
212164

Query: 602    CTATCCGCTTCAAATAAATAGATCATTGAAAGTGACGGGGATAACAGCATTTTACCTTTA
661
             ||||||||||||||||||||||||||||||||||||||||||||||||||||||||||||
Sbjct: 212165 CTATCCGCTTCAAATAAATAGATCATTGAAAGTGACGGGGATAACAGCATTTTACCTTTA
212224

Query: 378    AAAGACGTTCTCATAATACATTTTAGGATTAATACATATGCTTTTTTTTTT 328
             ||||||||||||||||||||||||||||||||||||||||||||||||||
Sbjct: 212225 AAAGACGTTCTCATAATACATTTTAGGATTAATACATATGCTTTTTTTTT- 212274

Query: 327    ATTCGAAATCTGGGGATTTTATACAGAGTTGTAAGTTAGGCAAACTAGAATTTGGT 276
             |||||||||||||||||| |||||||||||||||||||||||||||||||||||||
Sbjct: 212275 ATTCGAAATCTGGGGATTCTATACAGAGTTGTAAGTTAGGCAAACTAGAATTTGGT 212330
```

2. *CUP1* repeat (VIII212030-213192)

F1

```
Query: 489    AAATTTGAATTTCATAGAGTGCGGTGCATATGTATATATCTATATATGTTTGAAGTGT 546
             ||||||||||||||||||||||||||||||||||||||||||||||||||||||||||
Sbjct: 212030 AAATTTGAATTTCATAGAGTGCGGTGCATATGTATATATCTATATATGTTTGAAGTGT
212087
```

```
Query: 547    ATATTAAAAATAAAGTCATTATTTGAATATTGGTTTCTCGGTCTAAGAGCTTATACGTTT
606
              ||||||||||||||||||||||||||||||||||||||||||||||||||||||||||||
Sbjct: 212088 ATATTAAAAATAAAGTCATTATTTGAATATTGGTTTCTCGGTCTAAGAGCTTATACGTTT
212147
```

R1'

```
Query: 456    TAGACTGATCTGTTGTACTATCCGCTTCAAATAAATAGATCATTGAAAGTGACGGGGATA
397
              ||||||||||||||||||||||||||||||||||||||||||||||||||||||||||||
Sbjct: 212148 TAGACTGATCTGTTGTACTATCCGCTTCAAATAAATAGATCATTGAAAGTGACGGGGATA
212207


Query: 396    ACAGCATTTTACCTTTAAAAGACGTTCTCATAATACATTTTAGGATTAATACATATGCTT
337
              ||||||||||||||||||||||||||||||||||||||||||||||||||||||||||||
Sbjct: 212208 ACAGCATTTTACCTTTAAAAGACGTTCTCATAATACATTTTAGGATTAATACATATGCTT
212267


Query: 336    TTTTTTTTATTCGAAATCTGGGGATTTTATACAGAGTTGTAAGTTAGGCAAACTAGAATT
277
              ||||||| |||||||||||||||||| ||||||||||||||||||||||||||||||||||
Sbjct: 212268 TTTTTTT-ATTCGAAATCTGGGGATTCTATACAGAGTTGTAAGTTAGGCAAACTAGAATT
212326


Query: 276    TGGTAATAATATTTTATTCTTGGGGCGACATATGGAGATACTTTATTTCCTTTTCTTAAT
217
              ||||||||||||||||||||||||||||||||||||||||||||||||||||||||||||
Sbjct: 212327 TGGTAATAATATTTTATTCTTGGGGCGACATATGGAGATACTTTATTTCCTTTTCTTAAT
212386


Query: 216    TATTAACGTATACCTATAAATTAACAAAGTATCTAAACAAAATACATAAGTGTACTCAAA
157
              ||||||||||||||||||||||||||||||||||||||||||||||||||||||||||||
Sbjct: 212387 TATTAACGTATACCTATAAATTAACAAAGTATCTAAACAAAATACATAAGTGTACTCAAA
212446


Query: 156    CTGAGTAGAATCGTCGATTAAACTTCCTTCTCCTTTTAAAAATTAAAAACAGTAAATAGT 97
              |||||||||||||||||||||||||||||||||||||||||||||||||||| |||||||
Sbjct: 212447 CTGAGTAGAATCGTCGATTAAACTTCCTTCTCCTTTTAAAAATTAAAAACAGCAAATAGT
212506
```

VIII212300 F

```
Query: 180    TAAATGAA 187
              || |||||
Sbjct: 212507 TAGATGAA 212514
```

```
Query: 188    TATATTAAAGACTATTCGTTTTATTTCCCAGAGCAGCATGATTTCTTGGTTTCTTCAGAC
247
              ||||||||||||||||||||| ||||||||||||||||||| ||||||||||||||||||
Sbjct: 212515 TATATTAAAGACTATTCGTTTCATTTCCCAGAGCAGCATGACTTCTTGGTTTCTTCAGAC
212574


Query: 248    TTGTTACCGCAGGGGCATTTGTCGTCGCTGTTACACCCCGTTGGGCAGCTACATGATTTT
307
              ||||||||||||||||||||||||||||||||||||||||||||||||||||||||||||
Sbjct: 212575 TTGTTACCGCAGGGGCATTTGTCGTCGCTGTTACACCCCGTTGGGCAGCTACATGATTTT
212634


Query: 308    TGGCATTGTTCATTATTTTTGCAGCTACCACATTGGCATTGGCACTCATGACCTTCATTT
367
              ||||||||||||||||||||||||||||||||||||||||||||||||||||||||||||
Sbjct: 212635 TGGCATTGTTCATTATTTTTGCAGCTACCACATTGGCATTGGCACTCATGACCTTCATTT
212694


Query: 368    TGGAAGTTAATTAATTCGCTGAACATTTTATGTGATGATTGATTGATTG----TACGGTT
423
              |||||||||||||||||||||||||||||||||||||||||||||||||    ||| |||
Sbjct: 212695 TGGAAGTTAATTAATTCGCTGAACATTTTATGTGATGATTGATTGATTGATTGTACAGTT
212754


Query: 424    TGTTTTTGTTAATATCTATTTCGATGACTTCTATATGATATTGCACTAACAAGAAGATAT
483
              ||||||| ||||||||||||||||||||||||||||||||||||||||||||||||||||
Sbjct: 212755 TGTTTTTCTTAATATCTATTTCGATGACTTCTATATGATATTGCACTAACAAGAAGATAT
212814


Query: 484    TATAATGCAATTGGTACAAGACAAGGAGTTATTTGCTTCTCTTTTATATGATTCTGACAA
543
              ||||||||||||| ||||||||||||||||||||||||||||||||||||||||||||||
Sbjct: 212815 TATAATGCAATTGATACAAGACAAGGAGTTATTTGCTTCTCTTTTATATGATTCTGACAA
212874


Query: 544    TCCATATTGCGTTGGTAGTCTTTTTTGCTGGAACGGTTCAGCGGAAAAGACGCATCGCTC
603
              ||||||||||||||||||||||||||||||||||||||||||||||||||||||||||||
Sbjct: 212875 TCCATATTGCGTTGGTAGTCTTTTTTGCTGGAACGGTTCAGCGGAAAAGACGCATCGCTC
212934


Query: 604    TTTTTGCTTCTAGAAGAAATGCCAGCAAAAGAATCTCTTGACAGTGACTGACAGCAAAAA
663
              ||||||||||||||||||||||||||||||||||||||||||||||||||||||||||||
Sbjct: 212935 TTTTTGCTTCTAGAAGAAATGCCAGCAAAAGAATCTCTTGACAGTGACTGACAGCAAAAA
212994
```

F1

```
Query: 290    TGTCTTTTT 298
              |||||||||
Sbjct: 212995 TGTCTTTTT 213003


Query: 299    CTAACTAGTAACAAGGCTAAGATATCAGCCTGAAATAAAGGGTGGTGAAGTAATAATTAA
358
              ||||||||||||||||||||||||||||||||||||||||||||||||||||||||||||
Sbjct: 213004 CTAACTAGTAACAAGGCTAAGATATCAGCCTGAAATAAAGGGTGGTGAAGTAATAATTAA
213063


Query: 359    ATCATCCGTATAAACCTATACACATATATGAGGAAAAAATAATACAAAAGTGTTTTAAAT
418
              |||||||||||||||||||||||||||||||||||||| |||||||||||||||||||||
Sbjct: 213064 ATCATCCGTATAAACCTATACACATATATGAGGAAAAA-TAATACAAAAGTGTTTTAAAT
213122


Query: 419    ACAGATACATACATGAACATATGCACGTATAGCGTCCAAATGTCGGTAATGGGATCGGCT
478
              |||||||||||||||||||||||||||||||||| |||||||||||||||||||||||||| 
Sbjct: 213123 ACAGATACATACATGAACATATGCACGTATAGCGCCCAAATGTCGGTAATGGGATCGGCT
213182


Query: 479    TACTAATTAT 522
              ||||||||||
Sbjct: 213183 TACTAATTAT 213192
```

<u>3. ***CUP1-RSC30*** **(VIII212892-213492)**</u>

F1

```
Query: 188    GTCTTTTTTGCTGGAACGGTTCAGCGGAAAAGACGCATCGCTCTTTTTGCTT 239
              ||||||||||||||||||||||||||||||||||||||||||||||||||||
Sbjct: 212892 GTCTTTTTTGCTGGAACGGTTCAGCGGAAAAGACGCATCGCTCTTTTTGCTT 212943


Query: 240    CTAGAAGAAATGCCAGCAAAAGAATCTCTTGACAGTGACTGACAGCAAAAATGTCTTTTT
299
              ||||||||||||||||||||||||||||||||||||||||||||||||||||||||||||
Sbjct: 212944 CTAGAAGAAATGCCAGCAAAAGAATCTCTTGACAGTGACTGACAGCAAAAATGTCTTTTT
213003


Query: 300    CTAACTAGTAACAAGGCTAAGATATCAGCCTGAAATAAAGGGTGGTGAAGTAATAATTAA
359
              ||||||||||||||||||||||||||||||||||||||||||||||||||||||||||||
```

```
Sbjct: 213004 CTAACTAGTAACAAGGCTAAGATATCAGCCTGAAATAAAGGGTGGTGAAGTAATAATTAA
213063

Query: 360    ATCATCCGTATAAACCTATACACATATATGAGGAAAAAATAATACAAAAGTGTTTTAAAT
419
              |||||||||||||||||||||||||||||||||||||| |||||||||||||||||||||
Sbjct: 213064 ATCATCCGTATAAACCTATACACATATATGAGGAAAAA-TAATACAAAAGTGTTTTAAAT
213122

Query: 420    ACAGATACATACATGAACATATGCACGTATAGCGTCCAAATGTCGGTAATGAGATCGGCT
479
              |||||||||||||||||||||||||||||||||| |||||||||||||||| ||||||||
Sbjct: 213123 ACAGATACATACATGAACATATGCACGTATAGCGCCCAAATGTCGGTAATGGGATCGGCT
213182

Query: 480    TACTAATTATAAAATGCATCATAGAAATCGTTGAAGTTTGCCGTAGTAATACCCAGATTA
539
              ||||||||||||||||||||||||||||||||||||||||||||||||||||||||||||
Sbjct: 213183 TACTAATTATAAAATGCATCATAGAAATCGTTGAAGTTTGCCGTAGTAATACCCAGATTA
213242

Query: 540    TCAGATTCCAAATCCTTGTCAATAATTATACTCCTTTGGAAAACTTCTCTTTCCATTAAA
599
              |||||||||||||||||||||||||||||||||||||||| |||||||||||||||||||
Sbjct: 213243 TCAGATTCCAAATCCTTGTCAATAATTATACTCCTTTGGACAACTTCTCTTTCCATTAAA
213302

Query: 600    AAATCTGAAATCTCCTTAAATTTTAAATAGATTCTGTTCAGTTCACTAACGGGGAATTTC
659
              ||||||||||||||||||||||||||||||||||||||||||||||||||||||||||||
Sbjct: 213303 AAATCTGAAATCTCCTTAAATTTTAAATAGATTCTGTTCAGTTCACTAACGGGGAATTTC
213362

Query: 660    AAGAGAACATTTTTGTTCTTCGCCGACTGACTATAATCTGTAACATTATTATTATCAGAG
719
              |||||||||||||||||||||||||||||||||||||||||||||||||| |||||||||
Sbjct: 213363 AAGAGAACATTTTTGTTCTTCGCCGACTGACTATAATCTGTAACATTATTGTTATCAGAG
213422

Query: 720    TTTCTCGCAAAATTTTGTTTTTTCTTGCTAAATCTCAGCATATATTTAATCAGATTCAAA
779
              ||||||||||||||||||||||||||||||||||||||||||||||||||||||||||||
Sbjct: 213423 TTTCTCGCAAAATTTTGTTTTTTCTTGCTAAATCTCAGCATATATTTAATCAGATTCAAA
213482

Query: 780    ACCTTGTTGA 789
              ||||||||||
```

```
Sbjct: 213483 ACCTTGTTGA 213492
```

SNPs between YJM789 and S288c

Sequenced interval	Coordinate(s)	SNP in YJM789	SNP in S288c
CIC1-CUP1 VIII211728-212328			
	212266-212274	10 T's	9 T's
	212293	T	C
CUP1 repeat<br>VIII212028-213190			
	212266-212274	10 T's	9 T's
	212293	T	C
	212499	T	C
	212509	A	G
	212536	T	C
	212556	T	C
	212744-212747	4 bp deletion	ATTG
	212751	G	A
	212762	G	C
	212828	G	A
	213097-213101	6 A's	5 A's
	213157	T	C
CUP1-RSC30<br>VIII212890-213490			
	213097-213101	6 A's	5 A's
	213157	T	C
	213174	A	G
	213283	A	C
	213413	A	G
