# Supplementary material for: Structures of Naturally Evolved CUP1 Tandem Arrays in Yeast Indicate That These Arrays Are Generated by Unequal Nonhomologous Recombination
Source: G3 (Bethesda). 2014 Sep 17;4(11):2259–69. doi: 10.1534/g3.114.012922 (PMC4232551; doi:10.1534/g3.114.012922)
Supplement: Supporting Information [file supp_g3.114.012922_TableS6.pdf]

Table S6 Sequence analysis of the *CUP1* repeats (Type 4, 1.9 kb) of YJM271.

In this table, we show genomic sequences of YJM271 in three regions: 1) the sequences that flank the *CUP1* repeats adjacent to *CIC1*, 2) the sequence of the *CUP1* repeat, and 3) the sequences that flank the *CUP1* tandem array adjacent to *RCS30*. The sequences of YJM271 (denoted “Query” below) were compared in a BLAST search with sequences of S288c (denoted “Sbject”). SNPs that distinguish YJM271 and S288c sequences are summarized at the end of the table. The *CUP1* coding sequences are shown in red. The names of the primers used in the sequence analysis are shown in boldface. Additional details about the sequencing are in Supporting Data File S1.

1. *CIC1-CUP1* (VIII211758-212358)

VIII211528 F

```
Query: 202    AAAAGGACAAAATCGAAGAAACCCACGAAGATGACATGGTCACCAT 247
              ||||||||||||||||||||||||||||||||||||||||||||||
Sbjct: 211758 AAAAGGACAAAATCGAAGAAACCCACGAAGATGACATGGTCACCAT 211803


Query: 248    TGATGGTGTACAAGTTCATTTATCTACCTTCAACAAGGGTTTGATGGAAATCGCCAATCC
307
              ||||||||||||||||||||| ||||||||||||||||||||||||||||||||||||||
Sbjct: 211804 TGATGGTGTACAAGTTCATTTGTCTACCTTCAACAAGGGTTTGATGGAAATCGCCAATCC
211863


Query: 308    TTCCGAATTGGGTTCAATTTTCTCTAAACAAATTAACAATGCAAAAAAGAGATCTTCTAG
367
              ||||||||||||||||||||||||||||||||||||||||||||||||||||||||||||
Sbjct: 211864 TTCCGAATTGGGTTCAATTTTCTCTAAACAAATTAACAATGCAAAAAAGAGATCTTCTAG
211923


Query: 368    CGAGCTTGAAAAAGAATCTAGCGAGTCAGAAGCTGTCAAGAAGGCTAAAAGTTAATTTGT
427
              ||||||||||||||||||||||||||||||||||||||||||||||||||||||||||||
Sbjct: 211924 CGAGCTTGAAAAAGAATCTAGCGAGTCAGAAGCTGTCAAGAAGGCTAAAAGTTAATTTGT
211983
```

```
Query: 428    TTCCTCCTTATCTATCTTTTCTCTCATTTTTTTCTTGTGAAGAAAAAAATTTGAATTTCA
487
              ||||||||||||||||||||||||||||||||||||||||||||||||||||||||||||
Sbjct: 211984 TTCCTCCTTATCTATCTTTTCTCTCATTTTTTTCTTGTGAAGAAAAAAATTTGAATTTCA
212043


Query: 488    TAGAGTGCGGTGCATATGTATATATCTATATATGTTTGAAGTGTATATTAAAAATAAAGT
547
              ||||||||||||||||||||||||||||||||||||||||||||||||||||||||||||
Sbjct: 212044 TAGAGTGCGGTGCATATGTATATATCTATATATGTTTGAAGTGTATATTAAAAATAAAGT
212103


Query: 548    CATTATTTGAATATTGGTTTCTCGGTCTAAGAGCTTATACGTTTTAGACTGATCTGTTGT
607
              ||||||||||||||||||||||||||||||||||||||||||||||||||||||||||||
Sbjct: 212104 CATTATTTGAATATTGGTTTCTCGGTCTAAGAGCTTATACGTTTTAGACTGATCTGTTGT
212163
```

R1’

```
Query: 444    ACTATCCGCTTCAAATAAATAGATCAT 418
              |||||||||||||||||||||||||||
Sbjct: 212164 ACTATCCGCTTCAAATAAATAGATCAT 212190


Query: 417    TGAAAGTGACGGGGATAACAGCATTTTACCTTTAAAAGACGTTCTCATAATAGATTTTAG
358
              |||||||||||||||||||||||||||||||||||||||||||||||||||| |||||||
Sbjct: 212191 TGAAAGTGACGGGGATAACAGCATTTTACCTTTAAAAGACGTTCTCATAATACATTTTAG
212250


Query: 357    GATTAATACATATGCTTTTTTTTTTATTCGAAATCTGGGGATTCTATACAGAGTTGTAAG
298
              |||||||||||||||||||||||| |||||||||||||||||||||||||||||||||||
Sbjct: 212251 GATTAATACATATGCTTTTTTTTT-ATTCGAAATCTGGGGATTCTATACAGAGTTGTAAG
212309


Query: 297    TTAGGCAAACTAGAATTTGGTAATAATATTTTATTCTTGGGGCGACATA 249
              |||||||||||||||||||||||||||||||||||||||||||||||||
Sbjct: 212310 TTAGGCAAACTAGAATTTGGTAATAATATTTTATTCTTGGGGCGACATA 212358
```

2. *CUP1* repeat (VIII212058-213988)

R1’

```
Query: 555    TATGTATATATCTATATATGTTT 533
              |||||||||||||||||||||||
Sbjct: 212058 TATGTATATATCTATATATGTTT 212080
```

```
Query: 532    GAAGTGTATATTAAAAATAAAGTCATTATTTGAATATTGGTTTCTCGGTCTAAGAGCTTA
473
              ||||||||||||||||||||||||||||||||||||||||||||||||||||||||||||
Sbjct: 212081 GAAGTGTATATTAAAAATAAAGTCATTATTTGAATATTGGTTTCTCGGTCTAAGAGCTTA
212140


Query: 472    TACGTTTTAGACTGATCTGTTGTACTATCCGCTTCAAATAAATAGATCATTGAAAGTGAC
413
              ||||||||||||||||||||||||||||||||||||||||||||||||||||||||||||
Sbjct: 212141 TACGTTTTAGACTGATCTGTTGTACTATCCGCTTCAAATAAATAGATCATTGAAAGTGAC
212200


Query: 412    GGGGATAACAGCATTTTACCTTTAAAAGACGTTCTCATAATAGATTTTAGGATTAATACA
353
              |||||||||||||||||||||||||||||||||||||||||||| ||||||||||||||||
Sbjct: 212201 GGGGATAACAGCATTTTACCTTTAAAAGACGTTCTCATAATACATTTTAGGATTAATACA
212260


Query: 352    TATGCTTTTTTTTTATTCGAAATCTGGGGATTCTATACAGAGTTGTAAGTTAGGCAAAC
293
              |||||||||||||| ||||||||||||||||||||||||||||||||||||||||||||
Sbjct: 212261 TATGCTTTTTTTTT-ATTCGAAATCTGGGGATTCTATACAGAGTTGTAAGTTAGGCAAAC
212319


Query: 292    TAGAATTTGGTAATAATATTTTATTCTTGGGGCGACATATGGAGATACTTTATTTCCTTT
233
              ||||||||||||||||||||||||||||||||||||||||||||||||||||||||||||
Sbjct: 212320 TAGAATTTGGTAATAATATTTTATTCTTGGGGCGACATATGGAGATACTTTATTTCCTTT
212379


Query: 232    TCTTAATTATTAACGTATACCTATAAATTAACAAAGTATCTAAACAAAATACATAAGTGT
173
              ||||||||||||||||||||||||||||||||||||||||||||||||||||||||||||
Sbjct: 212380 TCTTAATTATTAACGTATACCTATAAATTAACAAAGTATCTAAACAAAATACATAAGTGT
212439


Query: 172    ACTCAAACTGAGTAGAATCGTCGATTAAACTTCCTTCTCCTTTTAAAAATTAAAAACAGC
113
              ||||||||||||||||||||||||||||||||||||||||||||||||||||||||||||
Sbjct: 212440 ACTCAAACTGAGTAGAATCGTCGATTAAACTTCCTTCTCCTTTTAAAAATTAAAAACAGC
212499
```

VIII212300 F

```
Query: 173    AAATAGTTAGATGAA 187
              |||||||||||||||
```

```
Sbjct: 212500 AAATAGTTAGATGAA 212514

Query: 188    TATATTAAAGACTATTCGTTTCATTTCCCAGAGCAGCATGACTTCTTGGTTTCTTCAGAC
247
              ||||||||||||||||||||||||||||||||||||||||||||||||||||||||||||
Sbjct: 212515 TATATTAAAGACTATTCGTTTCATTTCCCAGAGCAGCATGACTTCTTGGTTTCTTCAGAC
212574

Query: 248    TTGTTACCGCAGGGGCATTTGTCGTCGCTGTTACACCCCGTTGGGCAGCTACATGATTTT
307
              ||||||||||||||||||||||||||||||||||||||||||||||||||||||||||||
Sbjct: 212575 TTGTTACCGCAGGGGCATTTGTCGTCGCTGTTACACCCCGTTGGGCAGCTACATGATTTT
212634

Query: 308    TGGCATTGTTCATTATTTTTGCAGCTACCACATTGGCATTGGCACTCATGACCTTCATTT
367
              ||||||||||||||||||||||||||||||||||||||||||||||||||||||||||||
Sbjct: 212635 TGGCATTGTTCATTATTTTTGCAGCTACCACATTGGCATTGGCACTCATGACCTTCATTT
212694

Query: 368    TGGAAGTTAATTAATTCGCTGAACATTTTATGTGATGATTGATTGATTG----TACGGTT
423
              ||||||||||||||||||||||||||||||||||||||||||||||||    ||| |||
Sbjct: 212695 TGGAAGTTAATTAATTCGCTGAACATTTTATGTGATGATTGATTGATTGATTGTACAGTT
212754

Query: 424    TGTTTTTCTTAATATCTATTTCGATGACTTCTATATGATATTGCACTAACAAGAAGATAT
483
              ||||||||||||||||||||||||||||||||||||||||||||||||||||||||||||
Sbjct: 212755 TGTTTTTCTTAATATCTATTTCGATGACTTCTATATGATATTGCACTAACAAGAAGATAT
212814

Query: 484    TATAATGCAATTGATACAAGACAAGGAGTTATTTGCTTCTCTTTTATATGATTCTGACAA
543
              ||||||||||||||||||||||||||||||||||||||||||||||||||||||||||||
Sbjct: 212815 TATAATGCAATTGATACAAGACAAGGAGTTATTTGCTTCTCTTTTATATGATTCTGACAA
212874

Query: 544    TCCATATTGCGTTGGTAGTCTTTTTTGCTGGAACGGTTCAGCGGAAAAGACGCATCGCTC
603
              ||||||||||||||||||||||||||||||||||||||||||||||||||||||||||||
Sbjct: 212875 TCCATATTGCGTTGGTAGTCTTTTTTGCTGGAACGGTTCAGCGGAAAAGACGCATCGCTC
212934

Query: 604    TTTTTGCTTCTAGAAGAAATGCCAGCAAAAGAATCTCTTGACAGTGACTGACAGCAAAAA
663
              ||||||||||||||||||||||||||||||||||||||||||||||||||||||||||||
Sbjct: 212935 TTTTTGCTTCTAGAAGAAATGCCAGCAAAAGAATCTCTTGACAGTGACTGACAGCAAAAA
212994
```

F1

```
Query: 292    TGTCTT 297
              ||||||
Sbjct: 212995 TGTCTT 213000


Query: 298    TTTCTAACTAGTAACAAGGCTAAGATATCAGCCTGAAATAAAGGGTGGTGAAGTAATAAT
357
              ||||||||||||||||||||||||||||||||||||||||||||||||||||||||||||
Sbjct: 213001 TTTCTAACTAGTAACAAGGCTAAGATATCAGCCTGAAATAAAGGGTGGTGAAGTAATAAT
213060


Query: 358    TAAATCATCCGTATAAACCTATACACATATATGAGGAAAAATAATACAAAAGTGTTTTAA
417
              ||||||||||||||||||||||||||||||||||||||||||||||||||||||||||||
Sbjct: 213061 TAAATCATCCGTATAAACCTATACACATATATGAGGAAAAATAATACAAAAGTGTTTTAA
213120


Query: 418    ATACAGATACATACATGAACATATGCACGTATAGCGTCCAAATGTCGGTAATGGGATCGG
477
              |||||||||||||||||||||||||||||||||||| |||||||||||||||||||||||
Sbjct: 213121 ATACAGATACATACATGAACATATGCACGTATAGCGCCCAAATGTCGGTAATGGGATCGG
213180


Query: 478    CTTACTAATTATAAAATGCATCATAGAAATCGTTGAAGTTTGCCGTAGTAATACCCAGAT
537
              ||||||||||||||||||||||||||||||||||||||||||||||||||||||||||||
Sbjct: 213181 CTTACTAATTATAAAATGCATCATAGAAATCGTTGAAGTTTGCCGTAGTAATACCCAGAT
213240


Query: 538    TATCAGATTCCAAATCCTTGTCAATAATTATACTCCTTTGGAAAACTTCTCTTTCCATTA
597
              |||||||||||||||||||||||||||||||||||||||||| |||||||||||||||||
Sbjct: 213241 TATCAGATTCCAAATCCTTGTCAATAATTATACTCCTTTGGACAACTTCTCTTTCCATTA
213300


Query: 598    AAAAATCTGAAATCTCCTTAAATTTTAAATAGATTCTGTTCAGTTCACTAACGGGGAATT
657
              ||||||||||||||||||||||||||||||||||||||||||||||||||||||||||||
Sbjct: 213301 AAAAATCTGAAATCTCCTTAAATTTTAAATAGATTCTGTTCAGTTCACTAACGGGGAATT
213360
```

VIII213200 F

```
Query: 135    TCAAGAGAACATTTTTGTTCTTCGCCGACTGAGTATAATCTGTAACATTATT 186
              ||||||||||||||||||||||||||||||||||||||||||||||||||||
Sbjct: 213361 TCAAGAGAACATTTTTGTTCTTCGCCGACTGACTATAATCTGTAACATTATT 213412


Query: 187    ATTATCAGAGTTTCTCGCAAAATTTTGTTTTTTCTTGCTAAATCTCAGCATATATTTAAT
246
               |||||||||||||||||||||||||||||||||||||||||||||||||||||||||||
Sbjct: 213413 GTTATCAGAGTTTCTCGCAAAATTTTGTTTTTTCTTGCTAAATCTCAGCATATATTTAAT
213472


Query: 247    CAGATTCAAAACCTTGTTGAAACCTTTAATAGATTTGAAATTTCCGTTGCTATTCATTTC
306
              ||||||||||||||||||||||||||||||||||||||| ||||||||||||||||||||
Sbjct: 213473 CAGATTCAAAACCTTGTTGAAACCTTTAATAGATTTGAAACTTCCGTTGCTATTCATTTC
213532


Query: 307    ATCTCGTAAAAAGGATACGATAATTTCTATTTTTTTTAAAATTTCCAAAATCTTGTCATG
366
              ||||||||||||||||||||||||||||||||||||||||||||||||||||||||||||
Sbjct: 213533 ATCTCGTAAAAAGGATACGATAATTTCTATTTTTTTTAAAATTTCCAAAATCTTGTCATG
213592


Query: 367    AATCAATAGCAATTGAACATTAATCTCCTCATTTGAAAGATTTTTGTAAAATTCGTCATA
426
              ||||||||||||||||||||||||||||||||||||||||||||||||||||||||||||
Sbjct: 213593 AATCAATAGCAATTGAACATTAATCTCCTCATTTGAAAGATTTTTGTAAAATTCGTCATA
213652


Query: 427    TAATATTACTTCACAACGTTGGAAAATAGCAAATGTGATTGCTATAAAATTCTGTAAGAT
486
              ||||||||||||||||||||||||||||||||||||||||||||||||||||||||||||
Sbjct: 213653 TAATATTACTTCACAACGTTGGAAAATAGCAAATGTGATTGCTATAAAATTCTGTAAGAT
213712


Query: 487    TTCAATAAAATGATTTGCGAATAAAAATTCTTTACCATTAGAATGAAAGCGATTATTGCC
546
              ||||||||||||||||||||||||||||||||||||||||||||||||||||||||||||
Sbjct: 213713 TTCAATAAAATGATTTGCGAATAAAAATTCTTTACCATTAGAATGAAAGCGATTATTGCC
213772


Query: 547    GCTTGAAAATGACTTTATCGACTTTATGGGGAAGATAAAATTAAATGTTACTGAGTAAAA
606
              |||||||||||||||||||||||||||||||||||||||||||||||||| |||||||||
Sbjct: 213773 GCTTGAAAATGACTTTATCGACTTTATGGGGAAGATAAAATTAAATGTTATTGAGTAAAA
213832


Query: 607    AATGTGCATATTAGAAATAATTTTCATCAGATCCTTTGCACATCTTTCAGAGTTCGAGGT
666
              ||||||||||||||||||||||||||||||||||||||||||||||||||||||||||||
```

```
Sbjct: 213833 AATGTGCATATTAGAAATAATTTTCATCAGATCCTTTGCACATCTTTCAGAGTTCGAGGT
213892
```

```
Query: 667    CTTATTGTTGTTAGAAGAATGTTGAACTGCCATGGACAAAGAGGATTCGTTTTGAACAAA
726
              ||||||||||||||||||||||||||||||||||||||||||||||||||||||||||||
Sbjct: 213893 CTTATTGTTGTTAGAAGAATGTTGAACTGCCATGGACAAAGAGGATTCGTTTTGAACAAA
213952
```

R1’

```
Query: 595    AAGGAAAAAATTTGTATAAAC 575
              |||||||||||||||||||||
Sbjct: 213953 AAGGAAAAAATTTGTATAAAC 213973
```

```
Query: 574    AATGGTATTGATAAA 560
              |||||||||||||||
Sbjct: 213974 AATGGTATTGATAAA 213988
```

3. *CUP1-RSC30* (VIII213688-214288)

VIII213601 F

```
Query: 61     TGA 63
              |||
Sbjct: 213688 TGA 213690
```

```
Query: 64     TTGCTATAAAATTCTGTAAGATTTCAATAAAATGATTTGCGAATAAAAATTCTTTACCAT
123
              ||||||||||||||||||||||||||||||||||||||||||||||||||||||||||||
Sbjct: 213691 TTGCTATAAAATTCTGTAAGATTTCAATAAAATGATTTGCGAATAAAAATTCTTTACCAT
213750
```

```
Query: 124    TAGAATGAAAGCGATTATTGCCGCTTGAAAATGACTTTATCGACTTTATGGGGAAGATAA
183
              ||||||||||||||||||||||||||||||||||||||||||||||||||||||||||||
Sbjct: 213751 TAGAATGAAAGCGATTATTGCCGCTTGAAAATGACTTTATCGACTTTATGGGGAAGATAA
213810
```

```
Query: 184    AATTAAATGTTACTGAGTAAAAAATGTGCATATTAGAAATAATTTTCATCAGATCCTTTG
243
              |||||||||||| |||||||||||||||||||||||||||||||||||||||||||||||
Sbjct: 213811 AATTAAATGTTATTGAGTAAAAAATGTGCATATTAGAAATAATTTTCATCAGATCCTTTG
213870
```

```
Query: 244    CACATCTTTCAGAGTTCGAGGTCTTATTGTTGTTAGAAGAATGTTGAACTGCCATGGACA
303
              ||||||||||||||||||||||||||||||||||||||||||||||||||||||||||||
Sbjct: 213871 CACATCTTTCAGAGTTCGAGGTCTTATTGTTGTTAGAAGAATGTTGAACTGCCATGGACA
213930


Query: 304    AAGAGGATTCGTTTTGAACAAAAAGGAAAAAATTTGTATAAACAATGGTATTGATAAAAT
363
              ||||||||||||||||||||||||||||||||||||||||||||||||||||||||||||
Sbjct: 213931 AAGAGGATTCGTTTTGAACAAAAAGGAAAAAATTTGTATAAACAATGGTATTGATAAAAT
213990


Query: 364    TTAAAGTGTCTTTCCATTCTTTTCTGACTTCGTTGTCATGAAAATATAAGTCTACTGTAT
423
              ||||||||||||||||||||||||||||||||||||||||||||||||||||||||||||
Sbjct: 213991 TTAAAGTGTCTTTCCATTCTTTTCTGACTTCGTTGTCATGAAAATATAAGTCTACTGTAT
214050


Query: 424    TACTCACGCCCATAGTCAAGGTTTCTAACAGACTTTCAATTTTGGTTAAATTTACTGGCA
483
              ||||||||||||||||||||||||||||||||||||||||||||||||||||||||||||
Sbjct: 214051 TACTCACGCCCATAGTCAAGGTTTCTAACAGACTTTCAATTTTGGTTAAATTTACTGGCA
214110
```

VIII216603 R

```
Query: 468    AGTAGAAAGGAACATCTTGCAGAATATTTATCAATT 433
              ||||||||||||||||||||||||||||||||||||
Sbjct: 214111 AGTAGAAAGGAACACCTTGCAGAATATTTATCAATT 214146


Query: 432    TTGCTTGCGTTTCCAGTAATTTTAAATCGTTAGCAATTAAAGGAATGTCGTTCGTATCAA
373
              ||||||||||||||||||||||||||||||||||||||||||||||||||||||||||||
Sbjct: 214147 TTGCTTGCGTTTCCAGTAATTTTAAATCGTTAGCAATTAAAGGAATGTCGTTCGTATCAA
214206


Query: 372    TAGAGGCAGGTATCGGAGATAGGTTTTCAGCAGCGGGTACCATGAAT 326
              |||||||||||||||||||||||||||||||||||||||||||||||
Sbjct: 214207 TAGAGGCAGGTATCGGAGATAGGTTTTCAGCAGCGGGTACCATGAAT 214253


Query: 325    GAAGACTGACCTA 313
              |||||||||||||
Sbjct: 216252 GAAGACTGACCTA 216264


Query: 312    GAAGCGAATGTCTTGAGTAATA 277
              ||||||||||||||||||||||
Sbjct: 216265 GAAGCGAATGTCTTGAGTAATA 216286
```

SNPs between YJM271 and S288c

Sequenced interval	Coordinate(s)	SNP in YJM271	SNP in S288c
CIC1-CUP1 VIII211758-212358			
	211825	A	G
	212243	G	C
	212266-212274	10 T's	9 T's
CUP1 repeat VIII212058-213988			
	212243	G	C
	212266-212274	10 T's	9 T's
	212744-212747	4 bp deletion	ATTG
	212751	G	A
	213157	T	C
	213283	A	C
	213413	A	G
	213513	T	C
	213823	C	T
CUP1-RSC30 VIII213688-214288			
	213823	C	T
