# Supplementary material for: Structures of Naturally Evolved CUP1 Tandem Arrays in Yeast Indicate That These Arrays Are Generated by Unequal Nonhomologous Recombination
Source: G3 (Bethesda). 2014 Sep 17;4(11):2259–69. doi: 10.1534/g3.114.012922 (PMC4232551; doi:10.1534/g3.114.012922)
Supplement: Supporting Information [file supp_g3.114.012922_TableS7.pdf]

Table S7 Sequence analysis of the *CUP1* repeats (Type 4, 1.9 kb) of YJM1307.

In this table, we show genomic sequences of YJM1307 in three regions: 1) the sequences that flank the *CUP1* repeats adjacent to *CIC1*, 2) the sequence of the *CUP1* repeat, and 3) the sequences that flank the *CUP1* tandem array adjacent to *RCS30*. The sequences of YJM1307 (denoted “Query” below) were compared in a BLAST search with sequences of S288c (denoted “Sbject”). SNPs that distinguish YJM1307 and S288c sequences are summarized at the end of the table. The *CUP1* coding sequences are shown in red. The names of the primers used in the sequence analysis are shown in boldface. Additional details about the sequencing are in Supporting Data File S1.

1. ***CIC1-CUP1*** **(VIII211758-212358)**

VIII211528 F

```
Query: 202    AAAGGACAAAATCGAAGAAACCCACGAAGATGACATGGTCAC 244
              |||||||||||||||||||||||||||||||||||||||||||
Sbjct: 211758 AAAGGACAAAATCGAAGAAACCCACGAAGATGACATGGTCAC 211800


Query: 245    CATTGATGGTGTACAAGTTCATTTGTCTACCTTCAACAAGGGTTTGATGGAAATCGCCAA
304
              ||||||||||||||||||||||||||||||||||||||||||||||||||||||||||||
Sbjct: 211801 CATTGATGGTGTACAAGTTCATTTGTCTACCTTCAACAAGGGTTTGATGGAAATCGCCAA
211860


Query: 305    TCCTTCCGAATTGGGTTCAATTTTCTCTAAACAAATTAACAATGCAAAAAAGAGATCTTC
364
              ||||||||||||||||||||||||||||||||||||||||||||||||||||||||||||
Sbjct: 211861 TCCTTCCGAATTGGGTTCAATTTTCTCTAAACAAATTAACAATGCAAAAAAGAGATCTTC
211920


Query: 365    TAGCGAGCTTGAAAAAGAATCTAGCGAGTCAGAAGCTGTCAAGAAGGCTAAAAGTTAATT
424
              ||||||||||||||||||||||||||||||||||||||||||||||||||||||||||||
Sbjct: 211921 TAGCGAGCTTGAAAAAGAATCTAGCGAGTCAGAAGCTGTCAAGAAGGCTAAAAGTTAATT
211980


Query: 425    TGTTTCCTCCTTATCTATCTTTTCTCTCATTTTTTTCTTGTGAAGAAAAAAATTTGAATT
484
              ||||||||||||||||||||||||||||||||||||||||||||||||||||||||||||
Sbjct: 211981 TGTTTCCTCCTTATCTATCTTTTCTCTCATTTTTTTCTTGTGAAGAAAAAAATTTGAATT
212040
```

```
Query: 485    TCATAGAGTGCGGTGCATATGTATATATCTATATATGTTTGAAGTGTATATTAAAAATAA
544
              ||||||||||||||||||||||||||||||||||||||||||||||||||||||||||||
Sbjct: 212041 TCATAGAGTGCGGTGCATATGTATATATCTATATATGTTTGAAGTGTATATTAAAAATAA
212100
```

R1'

```
Query: 506    AGTCATTATTTGAATATTGGTTTCTCGGTCTAAGAGCTTATACGTTTTA 457
              ||||||||||||||||||||||||||||||||||||||||||||||||||
Sbjct: 212100 AGTCATTATTTGAATATTGGTTTCTCGGTCTAAGAGCTTATACGTTTTA 212149

Query: 456    GACTGATCTGTTGTACTATCCGCTTCAAATAAATAGATCATTGAAAGTGACGGGGATAAC
397
              ||||||||||||||||||||||||||||||||||||||||||||||||||||||||||||
Sbjct: 212150 GACTGATCTGTTGTACTATCCGCTTCAAATAAATAGATCATTGAAAGTGACGGGGATAAC
212209

Query: 396    AGCATTTTACCTTTAAAAGACGTTCTCATAATAGATTTTAGGATTAATACATATGCTTTT
337
              ||||||||||||||||||||||||||||||||| ||||||||||||||||||||||||||
Sbjct: 212210 AGCATTTTACCTTTAAAAGACGTTCTCATAATACATTTTAGGATTAATACATATGCTTTT
212269

Query: 336    TTTTTTATTCGAAATCTGGGGATTCTATACAGAGTTGTAAGTTAGGCAAACTAGAATTTG
277
              ||||| ||||||||||||||||||||||||||||||||||||||||||||||||||||||
Sbjct: 212270 TTTTT-ATTCGAAATCTGGGGATTCTATACAGAGTTGTAAGTTAGGCAAACTAGAATTTG
212328

Query: 276    GTAATAATATTTTATTCTTGGGGCGACATA 247
              ||||||||||||||||||||||||||||||
Sbjct: 212329 GTAATAATATTTTATTCTTGGGGCGACATA 212358
```

2. *CUP1* repeat (VIII212058-213988)

R1'

```
Query: 630    TATGTATATATCTATATATGTTT 537
              |||||||||||||||||||||||
Sbjct: 212058 TATGTATATATCTATATATGTTT 212080
```

```
Query: 536    GAAGTGTATATTAAAAATAAAGTCATTATTTGAATATTGGTTTCTCGGTCTAAGAGCTTA
477
              ||||||||||||||||||||||||||||||||||||||||||||||||||||||||||||
Sbjct: 212081 GAAGTGTATATTAAAAATAAAGTCATTATTTGAATATTGGTTTCTCGGTCTAAGAGCTTA
212140


Query: 476    TACGTTTTAGACTGATCTGTTGTACTATCCGCTTCAAATAAATAGATCATTGAAAGTGAC
417
              ||||||||||||||||||||||||||||||||||||||||||||||||||||||||||||
Sbjct: 212141 TACGTTTTAGACTGATCTGTTGTACTATCCGCTTCAAATAAATAGATCATTGAAAGTGAC
212200


Query: 416    GGGGATAACAGCATTTTACCTTTAAAAGACGTTCTCATAATAGATTTTAGGATTAATACA
357
              ||||||||||||||||||||||||||||||||||||||||||| ||||||||||||||||
Sbjct: 212201 GGGGATAACAGCATTTTACCTTTAAAAGACGTTCTCATAATACATTTTAGGATTAATACA
212260


Query: 356    TATGCTTTTTTTTTATTCGAAATCTGGGGATTCTATACAGAGTTGTAAGTTAGGCAAAC
297
              |||||||||||||| |||||||||||||||||||||||||||||||||||||||||||||
Sbjct: 212261 TATGCTTTTTTTTT-ATTCGAAATCTGGGGATTCTATACAGAGTTGTAAGTTAGGCAAAC
212319


Query: 296    TAGAATTTGGTAATAATATTTTATTCTTGGGGCGACATATGGAGATACTTTATTTCCTTT
237
              ||||||||||||||||||||||||||||||||||||||||||||||||||||||||||||
Sbjct: 212320 TAGAATTTGGTAATAATATTTTATTCTTGGGGCGACATATGGAGATACTTTATTTCCTTT
212379


Query: 236    TCTTAATTATTAACGTATACCTATAAATTAACAAAGTATCTAAACAAAATACATAAGTGT
177
              ||||||||||||||||||||||||||||||||||||||||||||||||||||||||||||
Sbjct: 212380 TCTTAATTATTAACGTATACCTATAAATTAACAAAGTATCTAAACAAAATACATAAGTGT
212439


Query: 176    ACTCAAACTGAGTAGAATCGTCGATTAAACTTCCTTCTCCTTTTAAAAATTAAAAACAGC
117
              ||||||||||||||||||||||||||||||||||||||||||||||||||||||||||||
Sbjct: 212440 ACTCAAACTGAGTAGAATCGTCGATTAAACTTCCTTCTCCTTTTAAAAATTAAAAACAGC
212499
```

VIII212300 F

```
Query: 174    AAATAGTTAGATGA 187
              ||||||||||||||
Sbjct: 212500 AAATAGTTAGATGA 212513
```

```
Query: 188    ATATATTAAAGACTATTCGTTTCATTTCCCAGAGCAGCATGACTTCTTGGTTTCTTCAGA
247
              ||||||||||||||||||||||||||||||||||||||||||||||||||||||||||||
Sbjct: 212514 ATATATTAAAGACTATTCGTTTCATTTCCCAGAGCAGCATGACTTCTTGGTTTCTTCAGA
212573


Query: 248    CTTGTTACCGCAGGGGCATTTGTCGTCGCTGTTACACCCCGTTGGGCAGCTACATGATTT
307
              ||||||||||||||||||||||||||||||||||||||||||||||||||||||||||||
Sbjct: 212574 CTTGTTACCGCAGGGGCATTTGTCGTCGCTGTTACACCCCGTTGGGCAGCTACATGATTT
212633


Query: 308    TTGGCATTGTTCATTATTTTTGCAGCTACCACATTGGCATTGGCACTCATGACCTTCATT
367
              ||||||||||||||||||||||||||||||||||||||||||||||||||||||||||||
Sbjct: 212634 TTGGCATTGTTCATTATTTTTGCAGCTACCACATTGGCATTGGCACTCATGACCTTCATT
212693


Query: 368    TTGGAAGTTAATTAATTCGCTGAACATTTTATGTGATGATTGATTGATTG----TACGGT
423
              ||||||||||||||||||||||||||||||||||||||||||||||||||    ||| ||
Sbjct: 212694 TTGGAAGTTAATTAATTCGCTGAACATTTTATGTGATGATTGATTGATTGATTGTACAGT
212753


Query: 424    TTGTTTTTCTTAATATCTATTTCGATGACTTCTATATGATATTGCACTAACAAGAAGATA
483
              ||||||||||||||||||||||||||||||||||||||||||||||||||||||||||||
Sbjct: 212754 TTGTTTTTCTTAATATCTATTTCGATGACTTCTATATGATATTGCACTAACAAGAAGATA
212813


Query: 484    TTATAATGCAATTGATACAAGACAAGGAGTTATTTGCTTCTCTTTTATATGATTCTGACA
543
              ||||||||||||||||||||||||||||||||||||||||||||||||||||||||||||
Sbjct: 212814 TTATAATGCAATTGATACAAGACAAGGAGTTATTTGCTTCTCTTTTATATGATTCTGACA
212873


Query: 544    ATCCATATTGCGTTGGTAGTCTTTTTTGCTGGAACGGTTCAGCGGAAAAGACGCATCGCT
603
              ||||||||||||||||||||||||||||||||||||||||||||||||||||||||||||
Sbjct: 212874 ATCCATATTGCGTTGGTAGTCTTTTTTGCTGGAACGGTTCAGCGGAAAAGACGCATCGCT
212933


Query: 604    CTTTTTGCTTCTAGAAGAAATGCCAGCAAAAGAATCTCTTGACAGTGACTGACAGCAAAA
663
              ||||||||||||||||||||||||||||||||||||||||||||||||||||||||||||
Sbjct: 212934 CTTTTTGCTTCTAGAAGAAATGCCAGCAAAAGAATCTCTTGACAGTGACTGACAGCAAAA
212993
```

F1

```
Query: 289    ATGTCTTT 296
              ||||||||
Sbjct: 212994 ATGTCTTT 213001


Query: 297    TTCTAACTAGTAACAAGGCTAAGATATCAGCCTGAAATAAAGGGTGGTGAAGTAATAATT
356
              ||||||||||||||||||||||||||||||||||||||||||||||||||||||||||||
Sbjct: 213002 TTCTAACTAGTAACAAGGCTAAGATATCAGCCTGAAATAAAGGGTGGTGAAGTAATAATT
213061


Query: 357    AAATCATCCGTATAAACCTATACACATATATGAGGAAAAATAATACAAAAGTGTTTTAAA
416
              ||||||||||||||||||||||||||||||||||||||||||||||||||||||||||||
Sbjct: 213062 AAATCATCCGTATAAACCTATACACATATATGAGGAAAAATAATACAAAAGTGTTTTAAA
213121


Query: 417    TACAGATACATACATGAACATATGCACGTATAGCGTCCAAATGTCGGTAATGGGATCGGC
476
              |||||||||||||||||||||||||||||||||| |||||||||||||||||||||||||
Sbjct: 213122 TACAGATACATACATGAACATATGCACGTATAGCGCCCAAATGTCGGTAATGGGATCGGC
213181


Query: 477    TTACTAATTATAAAATGCATCATAGAAATCGTTGAAGTTTGCCGTAGTAATACCCAGATT
536
              ||||||||||||||||||||||||||||||||||||||||||||||||||||||||||||
Sbjct: 213182 TTACTAATTATAAAATGCATCATAGAAATCGTTGAAGTTTGCCGTAGTAATACCCAGATT
213241


Query: 537    ATCAGATTCCAAATCCTTGTCAATAATTATACTCCTTTGGAAAACTTCTCTTTCCATTAA
596
              ||||||||||||||||||||||||||||||||||||||||| ||||||||||||||||||
Sbjct: 213242 ATCAGATTCCAAATCCTTGTCAATAATTATACTCCTTTGGACAACTTCTCTTTCCATTAA
213301


Query: 597    AAAATCTGAAATCTCCTTAAATTTTAAATAGATTCTGTTCAGTTCACTAACGGGGAATTT
656
              ||||||||||||||||||||||||||||||||||||||||||||||||||||||||||||
Sbjct: 213302 AAAATCTGAAATCTCCTTAAATTTTAAATAGATTCTGTTCAGTTCACTAACGGGGAATTT
213361
```

VIII213200 F

```
Query: 134    CAAGAGAACATTTTTGTTCTTCGCCGACTGAGTATAATCTGTAACATTAT 183
              ||||||||||||||||||||||||||||||| ||||||||||||||||||
Sbjct: 213362 CAAGAGAACATTTTTGTTCTTCGCCGACTGACTATAATCTGTAACATTAT 213411
```

```
Query: 184    TATTATCAGAGTTTCTCGCAAAATTTTGTTTTTTCTTGCTAAATCTCAGCATATATTTAA
243
              | ||||||||||||||||||||||||||||||||||||||||||||||||||||||||||
Sbjct: 213412 TGTTATCAGAGTTTCTCGCAAAATTTTGTTTTTTCTTGCTAAATCTCAGCATATATTTAA
213471


Query: 244    TCAGATTCAAAACCTTGTTGAAACCTTTAATAGATTTGAAATTTCCGTTGCTATTCATTT
303
              ||||||||||||||||||||||||||||||||||||||||| ||||||||||||||||||
Sbjct: 213472 TCAGATTCAAAACCTTGTTGAAACCTTTAATAGATTTGAAACTTCCGTTGCTATTCATTT
213531


Query: 304    CATCTCGTAAAAAGGATACGATAATTTCTATTTTTTTTAAAATTTCCAAAATCTTGTCAT
363
              ||||||||||||||||||||||||||||||||||||||||||||||||||||||||||||
Sbjct: 213532 CATCTCGTAAAAAGGATACGATAATTTCTATTTTTTTTAAAATTTCCAAAATCTTGTCAT
213591


Query: 364    GAATCAATAGCAATTGAACATTAATCTCCTCATTTGAAAGATTTTTGTAAAATTCGTCAT
423
              ||||||||||||||||||||||||||||||||||||||||||||||||||||||||||||
Sbjct: 213592 GAATCAATAGCAATTGAACATTAATCTCCTCATTTGAAAGATTTTTGTAAAATTCGTCAT
213651


Query: 424    ATAATATTACTTCACAACGTTGGAAAATAGCAAATGTGATTGCTATAAAATTCTGTAAGA
483
              ||||||||||||||||||||||||||||||||||||||||||||||||||||||||||||
Sbjct: 213652 ATAATATTACTTCACAACGTTGGAAAATAGCAAATGTGATTGCTATAAAATTCTGTAAGA
213711


Query: 484    TTTCAATAAAATGATTTGCGAATAAAAATTCTTTACCATTAGAATGAAAGCGATTATTGC
543
              ||||||||||||||||||||||||||||||||||||||||||||||||||||||||||||
Sbjct: 213712 TTTCAATAAAATGATTTGCGAATAAAAATTCTTTACCATTAGAATGAAAGCGATTATTGC
213771


Query: 544    CGCTTGAAAATGACTTTATCGACTTTATGGGGAAGATAAAATTAAATGTTACTGAGTAAA
603
              ||||||||||||||||||||||||||||||||||||||||||||||||||| ||||||||
Sbjct: 213772 CGCTTGAAAATGACTTTATCGACTTTATGGGGAAGATAAAATTAAATGTTATTGAGTAAA
213831
```

R1’

```
Query: 754    AAATGTGCATATTAGAAATAAT 695
              ||||||||||||||||||||||
Sbjct: 213832 AAATGTGCATATTAGAAATAAT 213853
```

```
Query: 694    TTTCATCAGATCCTTTGCACATCTTTCAGAGTTCGAGGTCTTATTGTTGTTAGAAGAATG
635
              ||||||||||||||||||||||||||||||||||||||||||||||||||||||||||||
Sbjct: 213854 TTTCATCAGATCCTTTGCACATCTTTCAGAGTTCGAGGTCTTATTGTTGTTAGAAGAATG
213913


Query: 634    TTGAACTGCCATGGACAAAGAGGATTCGTTTTGAACAAAAAGGAAAAAATTTGTATAAAC
575
              ||||||||||||||||||||||||||||||||||||||||||||||||||||||||||||
Sbjct: 213914 TTGAACTGCCATGGACAAAGAGGATTCGTTTTGAACAAAAAGGAAAAAATTTGTATAAAC
213973


Query: 574    AATGGTATTGATAAA 560
              |||||||||||||||
Sbjct: 213974 AATGGTATTGATAAA 213988
```

3. ***CUP1-RSC30*** (VIII213688-214288)

VIII213601 F

```
Query: 63     GATTGCTATA 73
              ||||||||||
Sbjct: 213688 GATTGCTATA 213698


Query: 74     AAATTCTGTAAGATTTCAATAAAATGATTTGCGAATAAAAATTCTTTACCATTAGAATG 133
              |||||||||||||||||||||||||||||||||||||||||||||||||||||||||||
Sbjct: 213699 AAATTCTGTAAGATTTCAATAAAATGATTTGCGAATAAAAATTCTTTACCATTAGAATG
213757


Query: 134    AAAGCGATTATTGCCGCTTGAAAATGACTTTATCGACTTTATGGGGAAGATAAAATTAAA
193
              ||||||||||||||||||||||||||||||||||||||||||||||||||||||||||||
Sbjct: 213758 AAAGCGATTATTGCCGCTTGAAAATGACTTTATCGACTTTATGGGGAAGATAAAATTAAA
213817


Query: 194    TGTTACTGAGTAAAAAATGTGCATATTAGAAATAATTTTCATCAGATCCTTTGCACATCT
253
              ||||| ||||||||||||||||||||||||||||||||||||||||||||||||||||||
Sbjct: 213818 TGTTATTGAGTAAAAAATGTGCATATTAGAAATAATTTTCATCAGATCCTTTGCACATCT
213877


Query: 254    TTCAGAGTTCGAGGTCTTATTGTTGTTAGAAGAATGTTGAACTGCCATGGACAAAGAGGA
313
              ||||||||||||||||||||||||||||||||||||||||||||||||||||||||||||
Sbjct: 213878 TTCAGAGTTCGAGGTCTTATTGTTGTTAGAAGAATGTTGAACTGCCATGGACAAAGAGGA
213937
```

```
Query: 314    TTCGTTTTGAACAAAAAGGAAAAAATTTGTATAAACAATGGTATTGATAAAATTTAAAGT
373
              ||||||||||||||||||||||||||||||||||||||||||||||||||||||||||||
Sbjct: 213938 TTCGTTTTGAACAAAAAGGAAAAAATTTGTATAAACAATGGTATTGATAAAATTTAAAGT
213997


Query: 374    GTCTTTCCATTCTTTTCTGACTTCGTTGTCATGAAAATATAAGTCTACTGTATTACTCAC
433
              ||||||||||||||||||||||||||||||||||||||||||||||||||||||||||||
Sbjct: 213998 GTCTTTCCATTCTTTTCTGACTTCGTTGTCATGAAAATATAAGTCTACTGTATTACTCAC
214057


Query: 434    GCCCATAGTCAAGGTTTCTAACAGACTTTCAATTTTGGTTAAATTTACTGGCAAGTAGAA
493
              ||||||||||||||||||||||||||||||||||||||||||||||||||||||||||||
Sbjct: 214058 GCCCATAGTCAAGGTTTCTAACAGACTTTCAATTTTGGTTAAATTTACTGGCAAGTAGAA
214117


Query: 494    AGGAACATCTTGCAGAATATTTATCAATTTTGCTTGCGTTTCCAGTAATTTTAAATCGTT
553
              ||||||| ||||||||||||||||||||||||||||||||||||||||||||||||||||
Sbjct: 214118 AGGAACACCTTGCAGAATATTTATCAATTTTGCTTGCGTTTCCAGTAATTTTAAATCGTT
214177


Query: 554    AGCAATTAAAGGAATGTCGTTCGTATCAATAGAGGCAGGTATCGGAGATAGGTTTTCAGC
613
              ||||||||||||||||||||||||||||||||||||||||||||||||||||||||||||
Sbjct: 214178 AGCAATTAAAGGAATGTCGTTCGTATCAATAGAGGCAGGTATCGGAGATAGGTTTTCAGC
214237


Query: 614    AGCGGGTACCATGAAT 629
              ||||||||||||||||
Sbjct: 214238 AGCGGGTACCATGAAT 214253
```

VIII216603 R

```
Query: 325    GAAGACTGAC 316
              ||||||||||
Sbjct: 216252 GAAGACTGAC 216261


Query: 315    CTAGAAGCGAATGTCTTGAGTAATA 291
              |||||||||||||||||||||||||
Sbjct: 216262 CTAGAAGCGAATGTCTTGAGTAATA 216286
```

SNPs between YJM1307 and S288c

Sequenced interval	Coordinate(s)	SNP in YJM1307	SNP in S288c
CIC1-CUP1 VIII211758-212358			
	212243	G	C
	212266-212274	10 T's	9 T's
CUP1 repeat VIII212058-213988			
	212243	G	C
	212266-212274	10 T's	9 T's
	212744-212747	4 bp deletion	ATTG
	212751	G	A
	213157	T	C
	213283	A	C
	213393	G	C
	213413	A	G
	213513	T	C
	213823	C	T
CUP1-RSC30 VIII213688-214288			
	213823	C	T
	214125	T	C
