# Supplementary material for: Structures of Naturally Evolved CUP1 Tandem Arrays in Yeast Indicate That These Arrays Are Generated by Unequal Nonhomologous Recombination
Source: G3 (Bethesda). 2014 Sep 17;4(11):2259–69. doi: 10.1534/g3.114.012922 (PMC4232551; doi:10.1534/g3.114.012922)
Supplement: Supporting Information [file supp_g3.114.012922_TableS8.pdf]

Table S8 Sequence analysis of the *CUP1* repeats (Type 5, 1.6 kb) of YJM456.

In this table, we show genomic sequences of YJM456 in three regions: 1) the sequences that flank the *CUP1* repeats adjacent to *CIC1*, 2) the sequence of the *CUP1* repeat, and 3) the sequences that flank the *CUP1* tandem array adjacent to *RCS30*. The sequences of YJM456 (denoted "Query" below) were compared in a BLAST search with sequences of S288c (denoted "Sbject"). SNPs that distinguish YJM456 and S288c sequences are summarized at the end of the table. The *CUP1* coding sequences are shown in red. The names of the primers used in the sequence analysis are shown in boldface. Additional details about the sequencing are in Supporting Data File S1.

1. *CIC1-CUP1* (VIII211275-211875)

VIII211185 F

```
Query: 62     AAT 64
              |||
Sbjct: 211275 AAT 211277


Query: 65     TAGATTCAGAAGGAATCAAGGTTGATGAAATCATTTGCGGGAAAGACTTAAAGACCGTTT
124
              ||||||||||||||||||||||||||||||||||||||||||||||||||||||||||||
Sbjct: 211278 TAGATTCAGAAGGAATCAAGGTTGATGAAATCATTTGCGGGAAAGACTTAAAGACCGTTT
211337


Query: 125    ACAAGGCATATGAGACTAGAAACGCTTTTATATCTCAGTTTTCTTTGATTTTGGCTGACG
184
              |||||||||||||| |||||||||||||||||||||||||||||||||||||||||||||
Sbjct: 211338 ACAAGGCATATGAGGCTAGAAACGCTTTTATATCTCAGTTTTCTTTGATTTTGGCTGACG
211397


Query: 185    ACAGTATAGTTACATCTTTGCCAAAACTTATGGGAGGCAAAGCCTACAACAAAGTAGAAA
244
              ||||||||||||||||||||||||||||||||||||||||||||||||||||||||||||
Sbjct: 211398 ACAGTATAGTTACATCTTTGCCAAAACTTATGGGAGGCAAAGCCTACAACAAAGTAGAAA
211457


Query: 245    CTACCCCTATATCAATTAGAACACATGCAAATAAGGAATTTTCCTTGACCACTTTGACGA
304
```

```
               |||| |||||||||||||||||||||||||||||||||||||||||||||||||||||||
Sbjct: 211458 CTACTCCTATATCAATTAGAACACATGCAAATAAGGAATTTTCCTTGACCACTTTGACGA
211517


Query: 305    ACAATATCAAAAAGGTATACATGAATCAGTTGCCCGTTAAACTTCCAAGAGGTACCACGT
364
              |||||||||||||||| |||||||||||||||||||||||||||||||||||||||||||
Sbjct: 211518 ACAATATCAAAAAGGTTTACATGAATCAGTTGCCCGTTAAACTTCCAAGAGGTACCACGT
211577


Query: 365    TGAATGTCCATTTGGGTAATTTAGAATGGTTAAGGCCAGAAGAGTTTGTAGATAACGTTG
424
              ||||||||||||||||||||||||||||||||||||||||||||||||||||||||||||
Sbjct: 211578 TGAATGTCCATTTGGGTAATTTAGAATGGTTAAGGCCAGAAGAGTTTGTAGATAACGTTG
211637




Query: 425    AATTAATTTCTGAACAGGTAATCAAAGCATACCAAATCAGATCCATTTTTATCAAAACCA
484
              ||||||||||||||||| ||||||||||||||||||||||||||||||||||||| ||||
Sbjct: 211638 AATTAATTTCTGAACAGTTAATCAAAGCATACCAAATCAGATCCATTTTTATCAAGACCA
211697


Query: 485    ATAAGTCACCCGTATTGCCATTATACTATAACCAGGACGTTCTTGATGAACTTGAAGCTA
544
              ||| ||| ||||||||||||||||||||||||||||||||||||||||||||||||||||
Sbjct: 211698 ATAGGTCGCCCGTATTGCCATTATACTATAACCAGGACGTTCTTGATGAACTTGAAGCTA
211757


Query: 545    AAAAGGACAAAATCGAAGAAACCCACGAAGATGACATGGTCACCATTGATGGTGTACAAG
604
              ||||||||||||||||||||||||||||||||||||||||||||||||||||||||||||
Sbjct: 211758 AAAAGGACAAAATCGAAGAAACCCACGAAGATGACATGGTCACCATTGATGGTGTACAAG
211817


Query: 605    TTCATTTATCTACCTTCAACAAGGGTTTGATGGAAATCGCCAATCCTTCCGAATTGGG 662
              ||||||| ||||||||||||||||||||||||||||||||||||||||||||||||||
Sbjct: 211818 TTCATTTGTCTACCTTCAACAAGGGTTTGATGGAAATCGCCAATCCTTCCGAATTGGG
211875
```

2. *CUP1* repeat (VIII211575-213213)

VIII212063 R

```
Query: 455    CGTTGAATGTCCATTTGGGTAAT 433
              |||||||||||||||||||||||
Sbjct: 211575 CGTTGAATGTCCATTTGGGTAAT 211597
```

```
Query: 432    TTAGAATGGTTAAGGCCAGAAGAGTTTGTAGATAACGTTGAATTAATTTCTGAACAGGTA
373
              ||||||||||||||||||||||||||||||||||||||||||||||||||||||||| ||
Sbjct: 211598 TTAGAATGGTTAAGGCCAGAAGAGTTTGTAGATAACGTTGAATTAATTTCTGAACAGTTA
211657


Query: 372    ATCAAAGCATACCAAATCAGATCCATTTTTATCAAAACCAATAAGTCACCCGTATTGCCA
313
              ||||||||||||||||||||||||||||||||||| ||||||| ||| ||||||||||||
Sbjct: 211658 ATCAAAGCATACCAAATCAGATCCATTTTTATCAAGACCAATAGGTCGCCCGTATTGCCA
211717


Query: 312    TTATACTATAACCAGGACGTTCTTGATGAACTTGAAGCTAAAAAGGACAAAATCGAAGAA
253
              ||||||||||||||||||||||||||||||||||||||||||||||||||||||||||||
Sbjct: 211718 TTATACTATAACCAGGACGTTCTTGATGAACTTGAAGCTAAAAAGGACAAAATCGAAGAA
211777


Query: 252    ACCCACGAAGATGACATGGTCACCATTGATGGTGTACAAGTTCATTTATCTACCTTCAAC
193
              ||||||||||||||||||||||||||||||||||||||||||||||| ||||||||||||
Sbjct: 211778 ACCCACGAAGATGACATGGTCACCATTGATGGTGTACAAGTTCATTTGTCTACCTTCAAC
211837


Query: 192    AAGGGTTTGATGGAAATCGCCAATCCTTCCGAATTGGGTTCAATTTTCTCTAAACAAATT
133
              ||||||||||||||||||||||||||||||||||||||||||||||||||||||||||||
Sbjct: 211838 AAGGGTTTGATGGAAATCGCCAATCCTTCCGAATTGGGTTCAATTTTCTCTAAACAAATT
211897
```

R1

```
Query: 450    AACAATGCAAAAAAGAGATCTTCTAGCGAGCTTGAAAAAGAATCTAGC 403
              ||||||||||||||||||||||||||||||||||||||||||||||||
Sbjct: 211898 AACAATGCAAAAAAGAGATCTTCTAGCGAGCTTGAAAAAGAATCTAGC 211945


Query: 402    GAGTCAGAAGCTGTCAAGAAGGCTAAAAGTTAATTTGTTTCCTCCTTATCTATCTTTTCT
343
              ||||||||||||||||||||||||||||||||||||||||||||||||||||||||||||
Sbjct: 211946 GAGTCAGAAGCTGTCAAGAAGGCTAAAAGTTAATTTGTTTCCTCCTTATCTATCTTTTCT
212005


Query: 342    CTCATTTTTTTCTTGTGAAGAAAAAAATTTGAATTTCATAGAGTGCGGTGCATATGTATA
283
              ||||||||||||||||||||||||||||||||||||||||||||||||||||||||||||
Sbjct: 212006 CTCATTTTTTTCTTGTGAAGAAAAAAATTTGAATTTCATAGAGTGCGGTGCATATGTATA
212065
```

```
Query: 282    TATCTATATATGTTTGAAGTGTATATTAAAAATAAAGTCATTATTTGAATATTGGTTTCT
223
              ||||||||||||||||||||||||||||||||||||||||||||||||||||||||||||
Sbjct: 212066 TATCTATATATGTTTGAAGTGTATATTAAAAATAAAGTCATTATTTGAATATTGGTTTCT
212125


Query: 222    CGGTCTAAGAGCTTATACGTTTTAGACTGATCTGTTGTACTATCCGCTTCAAATAAATAG
163
              ||||||||||||||||||||||||||||||||||||||||||||||||||||||||||||
Sbjct: 212126 CGGTCTAAGAGCTTATACGTTTTAGACTGATCTGTTGTACTATCCGCTTCAAATAAATAG
212185


Query: 162    ATCATTGAAAGTGACGGGGATAACAGCATTTTACCTTTAAAAGACGTTCTCATAATAGAT
103
              ||||||||||||||||||||||||||||||||||||||||||||||||||||||||| ||
Sbjct: 212186 ATCATTGAAAGTGACGGGGATAACAGCATTTTACCTTTAAAAGACGTTCTCATAATACAT
212245
```

R1’

```
Query: 363    TTTAGGATTAATACAT 348
              ||||||||||||||||
Sbjct: 212246 TTTAGGATTAATACAT 212261


Query: 347    ATGCTTTTTTTTTATTCGAAATCTGGGGATTCTATACAGAGTTGTAAGTTAGGCAAACT
288
              ||||||||||||| ||||||||||||||||||||||||||||||||||||||||||||||
Sbjct: 212262 ATGCTTTTTTTTT-ATTCGAAATCTGGGGATTCTATACAGAGTTGTAAGTTAGGCAAACT
212320


Query: 287    AGAATTTGGTAATAATATTTTATTCTTGGGGCGACATATGGAGATACTTTATTTCCTTTT
228
              ||||||||||||||||||||||||||||||||||||||||||||||||||||||||||||
Sbjct: 212321 AGAATTTGGTAATAATATTTTATTCTTGGGGCGACATATGGAGATACTTTATTTCCTTTT
212380


Query: 227    CTTAATTATTAACGTATACCTATAAATTAACAAAGTATCTAAACAAAATACATAAGTGTA
168
              ||||||||||||||||||||||||||||||||||||||||||||||||||||||||||||
Sbjct: 212381 CTTAATTATTAACGTATACCTATAAATTAACAAAGTATCTAAACAAAATACATAAGTGTA
212440
```

VIII212300 F

```
Query: 115    CTCAAACTGAGTA 127
              |||||||||||||
```

```
Sbjct: 212441 CTCAAACTGAGTA 212453

Query: 128    GAATCGTCGATTAAACTTCCTTCTCCTTTTAAAAATTAAAAACAGCAAATAGTTAGATGA
187
              ||||||||||||||||||||||||||||||||||||||||||||||||||||||||||||
Sbjct: 212454 GAATCGTCGATTAAACTTCCTTCTCCTTTTAAAAATTAAAAACAGCAAATAGTTAGATGA
212513



Query: 188    ATATATTAAAGACTATTCGTTTTATTTCCCAGAGCAGCATGACTTCTTGGTTTCTTCAGA
247
              |||||||||||||||||||||| |||||||||||||||||||||||||||||||||||||
Sbjct: 212514 ATATATTAAAGACTATTCGTTTCATTTCCCAGAGCAGCATGACTTCTTGGTTTCTTCAGA
212573


Query: 248    CTTGTTACCGCAGGGGCATTTGTCGTCGCTGTTACACCCCGTTGGGCAGCTACATGATTT
307
              ||||||||||||||||||||||||||||||||||||||||||||||||||||||||||||
Sbjct: 212574 CTTGTTACCGCAGGGGCATTTGTCGTCGCTGTTACACCCCGTTGGGCAGCTACATGATTT
212633


Query: 308    TTGGCATTGTTCATTATTTTTGCAGCTACCACATTGGCATTGGCACTCATGACCTTCATT
367
              ||||||||||||||||||||||||||||||||||||||||||||||||||||||||||||
Sbjct: 212634 TTGGCATTGTTCATTATTTTTGCAGCTACCACATTGGCATTGGCACTCATGACCTTCATT
212693


Query: 368    TTGGAAGTTAATTAATTCGCTGAACATTTTATGTGATGATTGATTGATTG----TACGGT
423
              ||||||||||||||||||||||||||||||||||||||||||||||||||    ||| ||
Sbjct: 212694 TTGGAAGTTAATTAATTCGCTGAACATTTTATGTGATGATTGATTGATTGATTGTACAGT
212753


Query: 424    TTGTTTTTCTTAATATCTATTTCGATGACTTCTATATGATATTGCACTAACAAGAAGATA
483
              ||||||||||||||||||||||||||||||||||||||||||||||||||||||||||||
Sbjct: 212754 TTGTTTTTCTTAATATCTATTTCGATGACTTCTATATGATATTGCACTAACAAGAAGATA
212813


Query: 484    TTATAATGCAATTGATACAAGACAAGGAGTTATTTGCTTCTCTTTTATATGATTCTGACA
543
              ||||||||||||||||||||||||||||||||||||||||||||||||||||||||||||
Sbjct: 212814 TTATAATGCAATTGATACAAGACAAGGAGTTATTTGCTTCTCTTTTATATGATTCTGACA
212873


Query: 544    ATCCATATTGCGTTGGTAGTCTTTTTTGCTGGAACGGTTCAGCGGAAAAGACGCATCGCT
603
              ||||||||||||||||||||||||||||||||||||||||||||||||||||||||||||
```

```
Sbjct: 212874 ATCCATATTGCGTTGGTAGTCTTTTTTGCTGGAACGGTTCAGCGGAAAAGACGCATCGCT
212933


Query: 604    CTTTTTGCTTCTAGAAGAAATGCCAGCAAAAGAATCTCTTGACAGTGACTGACAGCAAAA
663
              ||||||||||||||||||||||||||||||||||||||||||||||||||||||||||||
Sbjct: 212934 CTTTTTGCTTCTAGAAGAAATGCCAGCAAAAGAATCTCTTGACAGTGACTGACAGCAAAA
212993
```

F1

```
Query: 291    ATGTCTTTTT 300
              ||||||||||
Sbjct: 212994 ATGTCTTTTT 213003


Query: 301    CTAACTAGTAACAAGGCTAAGATATCAGCCTGAAATAAAGGGTGGTGAAGTAATAATTAA
360
              ||||||||||||||||||||||||||||||||||||||||||||||||||||||||||||
Sbjct: 213004 CTAACTAGTAACAAGGCTAAGATATCAGCCTGAAATAAAGGGTGGTGAAGTAATAATTAA
213063


Query: 361    ATCATCCGTATAAACCTATACACATATATGAGGAAAAATAATACAAAAGTGTTTTAAATA
420
              ||||||||||||||||||||||||||||||||||||||||||||||||||||||||||||
Sbjct: 213064 ATCATCCGTATAAACCTATACACATATATGAGGAAAAATAATACAAAAGTGTTTTAAATA
213123


Query: 421    CAGATACATACATGAACATATGCACGTATAGCGTCCAAATGTCGGTAATGGGATCGGCTT
480
              ||||||||||||||||||||||||||||||||| ||||||||||||||||||||||||||
Sbjct: 213124 CAGATACATACATGAACATATGCACGTATAGCGCCCAAATGTCGGTAATGGGATCGGCTT
213183



Query: 481    ACTAATTATAAAATGCATCATAGAAATCGT 510
              ||||||||||||||||||||||||||||||
Sbjct: 213184 ACTAATTATAAAATGCATCATAGAAATCGT 213213
```

3. *CUP1-RSC30* (VIII212913-213513)

F1

```
Query: 214    CAGCGGAAAAGACGCATCGCTCTTTTTGCTTCTAGAAGAA 253
              ||||||||||||||||||||||||||||||||||||||||
Sbjct: 212913 CAGCGGAAAAGACGCATCGCTCTTTTTGCTTCTAGAAGAA 212952
```

```
Query: 254    ATGCCAGCAAAAGAATCTCTTGACAGTGACTGACAGCAAAAATGTCTTTTTCTAACTAGT
313
              ||||||||||||||||||||||||||||||||||||||||||||||||||||||||||||
Sbjct: 212953 ATGCCAGCAAAAGAATCTCTTGACAGTGACTGACAGCAAAAATGTCTTTTTCTAACTAGT
213012


Query: 314    AACAAGGCTAAGATATCAGCCTGAAATAAAGGGTGGTGAAGTAATAATTAAATCATCCGT
373
              ||||||||||||||||||||||||||||||||||||||||||||||||||||||||||||
Sbjct: 213013 AACAAGGCTAAGATATCAGCCTGAAATAAAGGGTGGTGAAGTAATAATTAAATCATCCGT
213072


Query: 374    ATAAACCTATACACATATATGAGGAAAAATAATACAAAAGTGTTTTAAATACAGATACAT
433
              ||||||||||||||||||||||||||||||||||||||||||||||||||||||||||||
Sbjct: 213073 ATAAACCTATACACATATATGAGGAAAAATAATACAAAAGTGTTTTAAATACAGATACAT
213132


Query: 434    ACATGAACATATGCACGTATAGCGTCCAAATGTCGGTAATGGGATCGGCTTACTAATTAT
493
              |||||||||||||||||||||||| |||||||||||||||||||||||||||||||||||
Sbjct: 213133 ACATGAACATATGCACGTATAGCGCCCAAATGTCGGTAATGGGATCGGCTTACTAATTAT
213192


Query: 494    AAAATGCATCATAGAAATCGTTGAAGTTTGCCGTAGTAATACCCAGATTATCAGATTCCA
553
              ||||||||||||||||||||||||||||||||||||||||||||||||||||||||||||
Sbjct: 213193 AAAATGCATCATAGAAATCGTTGAAGTTTGCCGTAGTAATACCCAGATTATCAGATTCCA
213252


Query: 554    AATCCTTGTCAATAATTATACTCCTTTGGAAAACTTCTCTTTCCATTAAAAAATCTGAAA
613
              |||||||||||||||||||||||||||||| |||||||||||||||||||||||||||||
Sbjct: 213253 AATCCTTGTCAATAATTATACTCCTTTGGACAACTTCTCTTTCCATTAAAAAATCTGAAA
213312


Query: 614    TCTCCTTAAATTTTAAATAGATTCTGTTCAGTTCACTAACGGGGAATTTCAAGAGAACAT
673
              ||||||||||||||||||||||||||||||||||||||||||||||||||||||||||||
Sbjct: 213313 TCTCCTTAAATTTTAAATAGATTCTGTTCAGTTCACTAACGGGGAATTTCAAGAGAACAT
213372
```

VIII213200 F

```
Query: 146    TTTTGTTCTTCGCCGACTGACTATAATCTGTAACATTA 183
              ||||||||||||||||||||||||||||||||||||||
Sbjct: 213373 TTTTGTTCTTCGCCGACTGACTATAATCTGTAACATTA 213410
```

```
Query: 184    TTGTTATCAGAGTTTCTCGCAAAATTTTGTTTTTTCTTGCTAAATCTCAGCATATATTTA
243
              ||||||||||||||||||||||||||||||||||||||||||||||||||||||||||||
Sbjct: 213411 TTGTTATCAGAGTTTCTCGCAAAATTTTGTTTTTTCTTGCTAAATCTCAGCATATATTTA
213470


Query: 244    ATCAGATTCAAAACCTTGTTGAAACCTTTAATAGATTTGAAAT 286
              ||||||||||||||||||||||||||||||||||||||||||
Sbjct: 213471 ATCAGATTCAAAACCTTGTTGAAACCTTTAATAGATTTGAAAC 213513
```

SNPs between YJM456 and S288c

Sequenced interval	Coordinate(s)	SNP in YJM456	SNP in S288c
CIC1-CUP1 VIII211275-211875			
	211352	A	G
	211462	C	T
	211534	A	T
	211655	G	T
	211693	A	G
	211701	A	G
	211705	A	G
	211825	A	G
CUP1 repeat VIII211575-213213			
	211655	G	T
	211693	A	G
	211701	A	G
	211705	A	G
	211825	A	G
	212243	G	C
	212266-212274	10 poly(T)	9 poly(T)
	212536	T	C
	212744-212747	4 bp deletion	ATTG
	212751	G	A
	213157	T	C
CUP1-RSC30 VIII212913-213513			
	213157	T	C
	213283	A	C
