# Supplementary material for: Structures of Naturally Evolved CUP1 Tandem Arrays in Yeast Indicate That These Arrays Are Generated by Unequal Nonhomologous Recombination
Source: G3 (Bethesda). 2014 Sep 17;4(11):2259–69. doi: 10.1534/g3.114.012922 (PMC4232551; doi:10.1534/g3.114.012922)
Supplement: Supporting Information [file supp_g3.114.012922_TableS9.pdf]

Table S9 Sequence analysis of the *CUP1* repeats (Type 5, 1.6 kb) of YJM969.

In this table, we show genomic sequences of YJM969 in three regions: 1) the sequences that flank the *CUP1* repeats adjacent to *CIC1*, 2) the sequence of the *CUP1* repeat, and 3) the sequences that flank the *CUP1* tandem array adjacent to *RCS30*. The sequences of YJM969 (denoted “Query” below) were compared in a BLAST search with sequences of S288c (denoted “Sbject”). SNPs that distinguish YJM969 and S288c sequences are summarized at the end of the table. The *CUP1* coding sequences are shown in red. The names of the primers used in the sequence analysis are shown in boldface. Additional details about the sequencing are in Supporting Data File S1.

1. ***CIC1-CUP1* (VIII211275-211875)**

VIII211185 F

```
Query: 1      AATTAGATTCAGAAGGAATCAAGGTTGATGAAATCATTTGCGGGAAAGACTTAAAGACCG 60
              ||||||||||||||||||||||||||||||||||||||||||||||||||||||||||||
Sbjct: 211275 AATTAGATTCAGAAGGAATCAAGGTTGATGAAATCATTTGCGGGAAAGACTTAAAGACCG
211334


Query: 61     TTTACAAGGCATATGAGGCTAGAAACGCTTTTATATCTCAGTTTTCTTTGATTTTGGCTG
120
              ||||||||||||||||||||||||||||||||||||||||||||||||||||||||||||
Sbjct: 211335 TTTACAAGGCATATGAGGCTAGAAACGCTTTTATATCTCAGTTTTCTTTGATTTTGGCTG
211394


Query: 121    ACGACAGTATAGTTACATCTTTGCCAAAACTTATGGGAGGCAAAGCCTACAACAAAGTAG
180
              ||||||||||||||||||||||||||||||||||||||||||||||||||||||||||||
Sbjct: 211395 ACGACAGTATAGTTACATCTTTGCCAAAACTTATGGGAGGCAAAGCCTACAACAAAGTAG
211454


Query: 181    AAACTACCCCTATATCAATTAGAACACATGCAAATAAGGAATTTTCCTTGACCACTTTGA
240
              ||||||| ||||||||||||||||||||||||||||||||||||||||||||||||||||
Sbjct: 211455 AAACTACTCCTATATCAATTAGAACACATGCAAATAAGGAATTTTCCTTGACCACTTTGA
211514


Query: 241    CGAACAATATCAAAAAGGTATACATGAATCAGTTGGCCGTTAAACTTCCAAGAGGTACCA
300
              ||||||||||||||||||| ||||||||||||||| ||||||||||||||||||||||||
```

```
Sbjct: 211515 CGAACAATATCAAAAAGGTTTACATGAATCAGTTGCCCGTTAAACTTCCAAGAGGTACCA
211574


Query: 301    CGTTGAATGTCCATTTGGGTAATTTAGAATGGTTAAGGCCAGAAGAGTTTGTAGATAACG
360
              ||||||||||||||||||||||||||||||||||||||||||||||||||||||||||||
Sbjct: 211575 CGTTGAATGTCCATTTGGGTAATTTAGAATGGTTAAGGCCAGAAGAGTTTGTAGATAACG
211634


Query: 361    TTGAATTAATTTCTGAACAGGTAATCAAAGCATACCAAATCAGATCCATTTTTATCAAAA
420
              |||||||||||||||||||| ||||||||||||||||||||||||||||||||||||| |
Sbjct: 211635 TTGAATTAATTTCTGAACAGTTAATCAAAGCATACCAAATCAGATCCATTTTTATCAAGA
211694


Query: 421    CCAATAAGTCGCCCGTATTGCCATTATACTATAACCAGGACGTTCTTGATGAACTTGAAG
480
              |||||| |||||||||||||||||||||||||||||||||||||||||||||||||||||
Sbjct: 211695 CCAATAGGTCGCCCGTATTGCCATTATACTATAACCAGGACGTTCTTGATGAACTTGAAG
211754


Query: 481    CTAAAAAGGACAAAATCGAAGAAACCCACGAAGATGACATGGTCACCATTGATGGTGTAC
540
              ||||||||||||||||||||||||||||||||||||||||||||||||||||||||||||
Sbjct: 211755 CTAAAAAGGACAAAATCGAAGAAACCCACGAAGATGACATGGTCACCATTGATGGTGTAC
211814


Query: 541    AAGTTCATTTATCTACCTTCAACAAGGGTTTGATGGAAATCGCCAATCCTTCCGAATTGG
600
              |||||||||| |||||||||||||||||||||||||||||||||||||||||||||||||
Sbjct: 211815 AAGTTCATTTGTCTACCTTCAACAAGGGTTTGATGGAAATCGCCAATCCTTCCGAATTGG
211874


Query: 601    G
              |
Sbjct: 211875 G
```

2. *CUP1* Repeat (VIII211575-213213)

F1

```
Query: 514    CGTTGAATGTCCATTTGGGTAAT 536
              |||||||||||||||||||||||
Sbjct: 211575 CGTTGAATGTCCATTTGGGTAAT 211597


Query: 537    TTAGAATGGTTAAGGCCAGAAGAGTTTGTAGATAACGTTGAATTAATTTCTGAACAGGTA
596
```

```
              ||||||||||||||||||||||||||||||||||||||||||||||||||||||||| ||
Sbjct: 211598 TTAGAATGGTTAAGGCCAGAAGAGTTTGTAGATAACGTTGAATTAATTTCTGAACAGTTA
211657


Query: 597    ATCAAAGCATACCAAATCAGATCCATTTTTATCAAAACCAATAAGTCGCCCGTATTGCCA
656
              ||||||||||||||||||||||||||||||||||| ||||||| ||||||||||||||||
Sbjct: 211658 ATCAAAGCATACCAAATCAGATCCATTTTTATCAAGACCAATAGGTCGCCCGTATTGCCA
211717


Query: 657    TTATACTATAACCAGGACGTTCTTGATGAACTTGAAGCTAAAAAGGACAAAATCGAAGAA
716
              ||||||||||||||||||||||||||||||||||||||||||||||||||||||||||||
Sbjct: 211718 TTATACTATAACCAGGACGTTCTTGATGAACTTGAAGCTAAAAAGGACAAAATCGAAGAA
211777


Query: 717    ACCCA 721
              |||||
Sbjct: 211778 ACCCA 211782
```

VIII212063 R

```
Query: 249    CGAAGATGACATGGTCACCATTGATGGTGTACAAGTTCATTTATCTACCTTCAACAAGGG
190
              |||||||||||||||||||||||||||||||||||||||||| |||||||||||||||||
Sbjct: 211783 CGAAGATGACATGGTCACCATTGATGGTGTACAAGTTCATTTGTCTACCTTCAACAAGGG
211842


Query: 189    TTTGATGGAAATCGCCAATCCTTCCGAATTGGGTTCAATTTTCTCTAAACAAATTAACAA
130
              ||||||||||||||||||||||||||||||||||||||||||||||||||||||||||||
Sbjct: 211843 TTTGATGGAAATCGCCAATCCTTCCGAATTGGGTTCAATTTTCTCTAAACAAATTAACAA
211902


Query: 129    TGCAAAAAAGAGATCTTCTAGCGAGCTTGAAAAAGAATCTAGCGAGTCAGAAGCTGTCAA 70
              ||||||||||||||||||||||||||||||||||||||||||||||||||||||||||||
Sbjct: 211903 TGCAAAAAAGAGATCTTCTAGCGAGCTTGAAAAAGAATCTAGCGAGTCAGAAGCTGTCAA
211962
```

R1'

```
Query: 665    GAAGGCTAAAAGTTAATTTGTTTCCTCCTTATCTATCTTTTCTCTCAT 594
              ||||||||||||||||||||||||||||||||||||||||||||||||
Sbjct: 211963 GAAGGCTAAAAGTTAATTTGTTTCCTCCTTATCTATCTTTTCTCTCAT 212010


Query: 593    TTTTTTCTTGTGAAGAAAAAAATTTGAATTTCATAGAGTGCGGTGCATATGTATATATCT
534
              ||||||||||||||||||||||||||||||||||||||||||||||||||||||||||||
```

```
Sbjct: 212011 TTTTTTCTTGTGAAGAAAAAAATTTGAATTTCATAGAGTGCGGTGCATATGTATATATCT
212070


Query: 533    ATATATGTTTGAAGTGTATATTAAAAATAAAGTCATTATTTGAATATTGGTTTCTCGGTC
474
              ||||||||||||||||||||||||||||||||||||||||||||||||||||||||||||
Sbjct: 212071 ATATATGTTTGAAGTGTATATTAAAAATAAAGTCATTATTTGAATATTGGTTTCTCGGTC
212130


Query: 473    TAAGAGCTTATACGTTTTAGACTGATCTGTTGTACTATCCGCTTCAAATAAATAGATCAT
414
              ||||||||||||||||||||||||||||||||||||||||||||||||||||||||||||
Sbjct: 212131 TAAGAGCTTATACGTTTTAGACTGATCTGTTGTACTATCCGCTTCAAATAAATAGATCAT
212190


Query: 413    TGAAAGTGACGGGGATAACAGCATTTTACCTTTAAAAGACGTTCTCATAATAGATTTTAG
354
              |||||||||||||||||||||||||||||||||||||||||||||||||||| |||||||
Sbjct: 212191 TGAAAGTGACGGGGATAACAGCATTTTACCTTTAAAAGACGTTCTCATAATACATTTTAG
212250


Query: 353    GATTAATACATATGCTTTTTTTTTTATTCGAAATCTGGGGATTCTATACAGAGTTGTAAG
294
              |||||||||||||||||||||||| |||||||||||||||||||||||||||||||||||
Sbjct: 212251 GATTAATACATATGCTTTTTTTTT-ATTCGAAATCTGGGGATTCTATACAGAGTTGTAAG
212309


Query: 293    TTAGGCAAACTAGAATTTGGTAATAATATTTTATTCTTGGGGCGACATATGGAGATACTT
234
              ||||||||||||||||||||||||||||||||||||||||||||||||||||||||||||
Sbjct: 212310 TTAGGCAAACTAGAATTTGGTAATAATATTTTATTCTTGGGGCGACATATGGAGATACTT
212369
```

VIII212300 F

```
Query: 42     TATTTCCTTTTCTTAATTATTAAC 65
              ||||||||||||||||||||||||
Sbjct: 212370 TATTTCCTTTTCTTAATTATTAAC 212393


Query: 66     GTATACCTATAAATTAACAAAGTATCTAAACAAAATACATAAGTGTACTCAAACTGAGTA
125
              ||||||||||||||||||||||||||||||||||||||||||||||||||||||||||||
Sbjct: 212394 GTATACCTATAAATTAACAAAGTATCTAAACAAAATACATAAGTGTACTCAAACTGAGTA
212453


Query: 126    GAATCGTCGATTAAACTTCCTTCTCCTTTTAAAAATTAAAAACAGCAAATAGTTAGATGA
185
```

```
                ||||||||||||||||||||||||||||||||||||||||||||||||||||||||||||
Sbjct: 212454 GAATCGTCGATTAAACTTCCTTCTCCTTTTAAAAATTAAAAACAGCAAATAGTTAGATGA
212513

Query: 186    ATATATTAAAGACTATTCGTTTTATTTCCCAGAGCAGCATGACTTCTTGGTTTCTTCAGA
245
              |||||||||||||||||||||| |||||||||||||||||||||||||||||||||||||
Sbjct: 212514 ATATATTAAAGACTATTCGTTTCATTTCCCAGAGCAGCATGACTTCTTGGTTTCTTCAGA
212573


Query: 246    CTTGTTACCGCAGGGGCATTTGTCGTCGCTGTTACACCCCGTTGGGCAGCTACATGATTT
305
              ||||||||||||||||||||||||||||||||||||||||||||||||||||||||||||
Sbjct: 212574 CTTGTTACCGCAGGGGCATTTGTCGTCGCTGTTACACCCCGTTGGGCAGCTACATGATTT
212633

Query: 306    TTGGCATTGTTCATTATTTTTGCAGCTACCACATTGGCATTGGCACTCATGACCTTCATT
365
              ||||||||||||||||||||||||||||||||||||||||||||||||||||||||||||
Sbjct: 212634 TTGGCATTGTTCATTATTTTTGCAGCTACCACATTGGCATTGGCACTCATGACCTTCATT
212693

Query: 366    TTGGAAGTTAATTAATTCGCTGAACATTTTATGTGATGATTGATTGATTG----TACGGT
421
              ||||||||||||||||||||||||||||||||||||||||||||||||||    ||| ||
Sbjct: 212694 TTGGAAGTTAATTAATTCGCTGAACATTTTATGTGATGATTGATTGATTGATTGTACAGT
212753

Query: 422    TTGTTTTTCTTAATATCTATTTCGATGACTTCTATATGATATTGCACTAACAAGAAGATA
481
              ||||||||||||||||||||||||||||||||||||||||||||||||||||||||||||
Sbjct: 212754 TTGTTTTTCTTAATATCTATTTCGATGACTTCTATATGATATTGCACTAACAAGAAGATA
212813

Query: 482    TTATAATGCAATTGATACAAGACAAGGAGTTATTTGCTTCTCTTTTATATGATTCTGACA
541
              ||||||||||||||||||||||||||||||||||||||||||||||||||||||||||||
Sbjct: 212814 TTATAATGCAATTGATACAAGACAAGGAGTTATTTGCTTCTCTTTTATATGATTCTGACA
212873

Query: 542    ATCCATATTGCGTTGGTAGTCTTTTTTGCTGGAACGGTTCAGCGGAAAAGACGCATCGCT
601
              ||||||||||||||||||||||||||||||||||||||||||||||||||||||||||||
Sbjct: 212874 ATCCATATTGCGTTGGTAGTCTTTTTTGCTGGAACGGTTCAGCGGAAAAGACGCATCGCT
212933
```

F1

```
Query: 234    CTTTTTGCTTCTA 246
              |||||||||||||
Sbjct: 212934 CTTTTTGCTTCTA 212946


Query: 247    GAAGAAATGCCAGCAAAAGAATCTCTCGACAGTGACTGACAGCAAAAATGTCTTTTTCTA 
306
              |||||||||||||||||||||||||| |||||||||||||||||||||||||||||||||
Sbjct: 212947 GAAGAAATGCCAGCAAAAGAATCTCTTGACAGTGACTGACAGCAAAAATGTCTTTTTCTA 
213006


Query: 307    ACTAGTAACAAGGCTAAGATATCAGCCTGAAATAAAGGGTGGTGAAGTAATAATTAAATC 
366
              ||||||||||||||||||||||||||||||||||||||||||||||||||||||||||||
Sbjct: 213007 ACTAGTAACAAGGCTAAGATATCAGCCTGAAATAAAGGGTGGTGAAGTAATAATTAAATC 
213066


Query: 367    ATCCGTATAAACCTATACACATATATGAGGAAAAATAATACAAAAGTGTTTTAAATACAG 
426
              ||||||||||||||||||||||||||||||||||||||||||||||||||||||||||||
Sbjct: 213067 ATCCGTATAAACCTATACACATATATGAGGAAAAATAATACAAAAGTGTTTTAAATACAG 
213126


Query: 427    ATACATACATGAACATATGCACGTATAGCGTCCAAATGTCGGTAATGGGATCGGCTTACT 
486
              |||||||||||||||||||||||||||||| |||||||||||||||||||||||||||||
Sbjct: 213127 ATACATACATGAACATATGCACGTATAGCGCCCAAATGTCGGTAATGGGATCGGCTTACT 
213186


Query: 487    AATTATAAAATGCATCATAGAAATCGT 513
              |||||||||||||||||||||||||||
Sbjct: 213187 AATTATAAAATGCATCATAGAAATCGT 213213
```

3. *CUP1-RSC30* (VIII212913-213513)

VIII212300 F

```
Query: 581    CAGCGGAAAAGACGCATCGCT 601
              |||||||||||||||||||||
Sbjct: 212913 CAGCGGAAAAGACGCATCGCT 212933
```

F1

```
Query: 234    CTTTTTGCTTCTA 246
              |||||||||||||
Sbjct: 212934 CTTTTTGCTTCTA 212946
```

```
Query: 247    GAAGAAATGCCAGCAAAAGAATCTCTCGACAGTGACTGACAGCAAAAATGTCTTTTTCTA
306
              |||||||||||||||||||||||||| |||||||||||||||||||||||||||||||||
Sbjct: 212947 GAAGAAATGCCAGCAAAAGAATCTCTTGACAGTGACTGACAGCAAAAATGTCTTTTTCTA
213006

Query: 307    ACTAGTAACAAGGCTAAGATATCAGCCTGAAATAAAGGGTGGTGAAGTAATAATTAAATC
366
              ||||||||||||||||||||||||||||||||||||||||||||||||||||||||||||
Sbjct: 213007 ACTAGTAACAAGGCTAAGATATCAGCCTGAAATAAAGGGTGGTGAAGTAATAATTAAATC
213066

Query: 367    ATCCGTATAAACCTATACACATATATGAGGAAAAATAATACAAAAGTGTTTTAAATACAG
426
              ||||||||||||||||||||||||||||||||||||||||||||||||||||||||||||
Sbjct: 213067 ATCCGTATAAACCTATACACATATATGAGGAAAAATAATACAAAAGTGTTTTAAATACAG
213126

Query: 425    ATACATACATGAACATATGCACGTATAGCGTCCAAATGTCGGTAATGGGATCGGCTTA 482
              |||||||||||||||||||||||||||||| |||||||||||||||||||||||||||
Sbjct: 213127 ATACATACATGAACATATGCACGTATAGCGCCCAAATGTCGGTAATGGGATCGGCTTA
213184

Query: 483    CTAATTATAAAATGCATCATAGAAATCGTTGAAGTTTGCCGTAGTAATACCCAGATTATC
542
              ||||||||||||||||||||||||||||||||||||||||||||||||||||||||||||
Sbjct: 213185 CTAATTATAAAATGCATCATAGAAATCGTTGAAGTTTGCCGTAGTAATACCCAGATTATC
213244

Query: 543    AGATTCCAAATCCTTGTCAATAATTATACTCCTTTGGAAAACTTCTCTTTCCATTAAAAA
602
              |||||||||||||||||||||||||||||||||||||| |||||||||||||||||||||
Sbjct: 213245 AGATTCCAAATCCTTGTCAATAATTATACTCCTTTGGACAACTTCTCTTTCCATTAAAAA
213304

Query: 79     ATCTGAAATCTCCTTAAATTTTAAATAGATTCTGTTCAGTTCACTAACGG 128
              ||||||||||||||||||||||||||||||||||||||||||||||||||
Sbjct: 213305 ATCTGAAATCTCCTTAAATTTTAAATAGATTCTGTTCAGTTCACTAACGG 213354

Query: 129    GGAATTTCAAGAGAACATTTTTGTTCTTCGCCGACTGACTATAATCTGTAACATTATTGT
188
              ||||||||||||||||||||||||||||||||||||||||||||||||||||||||||||
Sbjct: 213355 GGAATTTCAAGAGAACATTTTTGTTCTTCGCCGACTGACTATAATCTGTAACATTATTGT
213414

Query: 189    TATCAGAGTTTCTCGCAAAATTTTGTTTTTTCTTGCTAAATCTCAGCATATATTTAATCA
248
              ||||||||||||||||||||||||||||||||||||||||||||||||||||||||||||
```

```
Sbjct: 213415 TATCAGAGTTTCTCGCAAAATTTTGTTTTTTCTTGCTAAATCTCAGCATATATTTAATCA
213474


Query: 249    GATTCAAAACCTTGTTGAAACCTTTAATAGATTTGAAAT 287
              ||||||||||||||||||||||||||||||||||||||
Sbjct: 213475 GATTCAAAACCTTGTTGAAACCTTTAATAGATTTGAAAC 213513
```

SNPs between YJM969 and S288c

Sequenced interval	Coordinate(s)	SNP in YJM969	SNP in S288c
CIC1-CUP1 VIII211275-211875			
	211462	C	T
	211534	A	T
	211550	G	C
	211655	G	C
	211693	A	G
	211701	A	G
	211825	A	G
CUP1 repeat<br>VIII211575-213213			
	211655	G	T
	211693	A	G
	211701	A	G
	211825	A	G
	212243	G	C
	212266-212274	10 T's	9 T's
	212536	T	C
	212744-212747	4 bp deletion	ATTG
	212751	G	A
	212973	C	T
	213157	T	C
CUP1-RSC30<br>VIII212913-213513			
	212973	C	T
	213157	T	C
	213283	A	C
