# Supplementary material for: Structures of Naturally Evolved CUP1 Tandem Arrays in Yeast Indicate That These Arrays Are Generated by Unequal Nonhomologous Recombination
Source: G3 (Bethesda). 2014 Sep 17;4(11):2259–69. doi: 10.1534/g3.114.012922 (PMC4232551; doi:10.1534/g3.114.012922)
Supplement: Supporting Information [file supp_g3.114.012922_TableS10.pdf]

Table S10 Sequence analysis of strain (DTY3) that has a single copy of *CUP1*.

To determine the sequence of the single-copy *CUP1* gene and its flanking sequences, we generated three PCR fragments that had overlapping sequences as described in Supporting Data File S1. These fragments were sequenced using the primers shown in boldface below. The region between the 5’ end of *CIC1* and the 5’ end of *RSC30*, including the *CUP1* gene, is shown as two continuous sequences. One sequence matches with SGD coordinates 210848 (5’ end of *CIC1*) to 213868 (in the 3’ region of *RSC30*). The other sequence matches between 215867 (3’ region of *RSC30*) to 217834 (the 5’ end of *RSC30*). The discontinuity in the comparison between the DTY3 sequences and those of S288c arises because the S288c sequence contains two copies of the 2.0 kb Type 1 *CUP1* repeat instead of the single *CUP1* gene present in DTY3.

1. VIII210848-VIII213868

VIII210632 F

```
Query: 189    ATGGCTAAAAAGAGTAACTCAAAGAAATCTACGCCTGTAAGTACACCAAGCAAAG 243
              |||||||||||||||||||||||||||||||||||||||||||||||||||||||
Sbjct: 210848 ATGGCTAAAAAGAGTAACTCAAAGAAATCTACGCCTGTAAGTACACCAAGCAAAG 210902


Query: 244    AAAAGAAGAAGGTTATTGAAAAGAAATCTTCCACAGCCATTCCTAGGGAAAGAGTTATTA
303
              ||||||||||||||||||||||||||||||||||||||||||||||||||||||||||||
Sbjct: 210903 AAAAGAAGAAGGTTATTGAAAAGAAATCTTCCACAGCCATTCCTAGGGAAAGAGTTATTA
210962


Query: 304    AAGCTGTCAACGAGCTTATAAAATTCACTTCCAAGCCACAAGATGAAAATAATGAAGAAG
363
              ||||||||||||||||||||||||||||||||||||||||||||||||||||||||||||
Sbjct: 210963 AAGCTGTCAACGAGCTTATAAAATTCACTTCCAAGCCACAAGATGAAAATAATGAAGAAG
211022


Query: 364    GAAATAACGGTAAGAAAAACCTATTGGAAGATGATGAAGAAGAATTGAAGAAAGATCTGC
423
              ||||||||||||||||||||||||||||||||||||||||||||||||||||||||||||
Sbjct: 211023 GAAATAACGGTAAGAAAAACCTATTGGAAGATGATGAAGAAGAATTGAAGAAAGATCTGC
211082


Query: 424    AATTAATCGTAGTAAATAATAAATCATTCACCGGTACTTCCAAATCATTCAAATTGAAAT
483
              ||||||||||||||||||||||||||||||||||||||||||||||||||||||||||||
```

```
Sbjct: 211083 AATTAATCGTAGTAAATAATAAATCATTCACCGGTACTTCCAAATCATTCAAATTGAAAT
211142

Query: 484    TACTAAATGTCAAACATTCGTTTTACAAGCCTTGGAAAGAAGCCAGTGCAACAGCGGTTA
543
              ||||||||||||||||||||||||||||||||||||||||||||||||||||||||||||
Sbjct: 211143 TACTAAATGTCAAACATTCGTTTTACAAGCCTTGGAAAGAAGCCAGTGCAACAGCGGTTA
211202

Query: 544    AGGATTTCAAAGTTTTATTAATTTTGAAGGATTCTGATATTAAGAAAGTTTCAGAAGATG
603
              ||||||||||||||||||||||||||||||||||||||||||||||||||||||||||||
Sbjct: 211203 AGGATTTCAAAGTTTTATTAATTTTGAAGGATTCTGATATTAAGAAAGTTTCAGAAGATG
211262

Query: 604    ATTTATTTGATCAATTAGATTCAGAAGGAATCAAGGTTGATGAAATCATTTGCGGGAAAG
663
              ||||||||||||||||||||||||||||||||||||||||||||||||||||||||||||
Sbjct: 211263 ATTTATTTGATCAATTAGATTCAGAAGGAATCAAGGTTGATGAAATCATTTGCGGGAAAG
211322

Query: 664    ACTTAAAGACCGTTTACAAGGCATATGAGGCTAGAAACGCTTTTATATCTCAGTT 718
              |||||||||||||||||||||||||||||||||||||||||||||||||||||||
Sbjct: 211323 ACTTAAAGACCGTTTACAAGGCATATGAGGCTAGAAACGCTTTTATATCTCAGTT 211377
```

VIII212063 R

```
Query: 658    TTCTTTGATTTTGGCTGACGACAGTATAGTTACATCTTTGCCAAAACTTATGGGAGGCAA
599
              ||||||||||||||||||||||||||||||||||||||||||||||||||||||||||||
Sbjct: 211378 TTCTTTGATTTTGGCTGACGACAGTATAGTTACATCTTTGCCAAAACTTATGGGAGGCAA
211437

Query: 598    AGCCTACAACAAAGTAGAAACTACTCCTATATCAATTAGAACACATGCAAATAAGGAATT
539
              ||||||||||||||||||||||||||||||||||||||||||||||||||||||||||||
Sbjct: 211438 AGCCTACAACAAAGTAGAAACTACTCCTATATCAATTAGAACACATGCAAATAAGGAATT
211497

Query: 538    TTCCTTGACCACTTTGACGAACAATATCAAAAAGGTTTACATGAATCAGTTGCCCGTTAA
479
              ||||||||||||||||||||||||||||||||||||||||||||||||||||||||||||
Sbjct: 211498 TTCCTTGACCACTTTGACGAACAATATCAAAAAGGTTTACATGAATCAGTTGCCCGTTAA
211557

Query: 478    ACTTCCAAGAGGTACCACGTTGAATGTCCATTTGGGTAATTTAGAATGGTTAAGGCCAGA
419
              ||||||||||||||||||||||||||||||||||||||||||||||||||||||||||||
```

```
Sbjct: 211558 ACTTCCAAGAGGTACCACGTTGAATGTCCATTTGGGTAATTTAGAATGGTTAAGGCCAGA
211617


Query: 418    AGAGTTTGTAGATAACGTTGAATTAATTTCTGAACAGTTAATCAAAGCATACCAAATCAG
359
              ||||||||||||||||||||||||||||||||||||||||||||||||||||||||||||
Sbjct: 211618 AGAGTTTGTAGATAACGTTGAATTAATTTCTGAACAGTTAATCAAAGCATACCAAATCAG
211677


Query: 358    ATCCATTTTTATCAAAACCAATAAGTCGCCCGTATTGCCATTATACTATAACCAGGACGT
299
              ||||||||||||||| ||||||| ||||||||||||||||||||||||||||||||||||
Sbjct: 211678 ATCCATTTTTATCAAGACCAATAGGTCGCCCGTATTGCCATTATACTATAACCAGGACGT
211737


Query: 298    TCTTGATGAACTTGAAGCTAAAAAGGACAAAATCGAAGAAACCCACGAAGATGACATGGT
239
              ||||||||||||||||||||||||||||||||||||||||||||||||||||||||||||
Sbjct: 211738 TCTTGATGAACTTGAAGCTAAAAAGGACAAAATCGAAGAAACCCACGAAGATGACATGGT
211797


Query: 238    CACCATTGATGGTGTACAAGTTCATTTATCTACCTTCAACAAGGGTTTGATGGAAATCGC
179
              ||||||||||||||||||||||||||| ||||||||||||||||||||||||||||||||
Sbjct: 211798 CACCATTGATGGTGTACAAGTTCATTTGTCTACCTTCAACAAGGGTTTGATGGAAATCGC
211857


Query: 178    CAATCCTTCCGAATTGGGTTCAATTTTCTCTAAACAAATTAACAATGCAAAAAA 112
              ||||||||||||||||||||||||||||||||||||||||||||||||||||||
Sbjct: 211858 CAATCCTTCCGAATTGGGTTCAATTTTCTCTAAACAAATTAACAATGCAAAAAA 211910
```

VIII211849 F

```
Query: 33     GAGATCTTCTAGCGAGCTTGAAAAAGAATCTA 64
              ||||||||||||||||||||||||||||||||
Sbjct: 211911 GAGATCTTCTAGCGAGCTTGAAAAAGAATCTA 211943


Query: 65     GCGAGTCAGAAGCTGTCAAGAAGACTAAAAGTTAATTTGTGTCCTCCTTATCTATCTTTT
124
              ||||||||||||||||||||||| |||||||||||||||| |||||||||||||||||||
Sbjct: 211944 GCGAGTCAGAAGCTGTCAAGAAGGCTAAAAGTTAATTTGTTTCCTCCTTATCTATCTTTT
212003


Query: 125    CTCTCATTTTTTTCTTGTGAAGAAAAAAATTTGAATTTCATAGAGTGCGGTGCATATGTA
184
              ||||||||||||||||||||||||||||||||||||||||||||||||||||||||||||
Sbjct: 212004 CTCTCATTTTTTTCTTGTGAAGAAAAAAATTTGAATTTCATAGAGTGCGGTGCATATGTA
212063
```

```
Query: 185    TATATCTATATATGTTTGAAGTGTATATTAAAAATAAAGTCATTATTTGAATATTGGTTT
244
              ||||||||||||||||||||||||||||||||||||||||||||||||||||||||||||
Sbjct: 212064 TATATCTATATATGTTTGAAGTGTATATTAAAAATAAAGTCATTATTTGAATATTGGTTT
212123


Query: 245    CTCGGTCTAAGAGCTTATACGTTTTAGACTGATCTGTTGTACTATCCGCTTCAAATAAAT
304
              ||||||||||||||||||||||||||||||||||||||||||||||||||||||||||||
Sbjct: 212124 CTCGGTCTAAGAGCTTATACGTTTTAGACTGATCTGTTGTACTATCCGCTTCAAATAAAT
212183


Query: 305    AGATCATTGAAAGTGACGGGGATAACAGCATTTTACCTTTAAAAGACGTTCTCATAATAC
364
              ||||||||||||||||||||||||||||||||||||||||||||||||||||||||||||
Sbjct: 212184 AGATCATTGAAAGTGACGGGGATAACAGCATTTTACCTTTAAAAGACGTTCTCATAATAC
212243


Query: 365    ATTTTAGGATTAATACATATGCTTTTTTTTTATTCAAAATCTGGGGATTTTATACAGAGT
424
              |||||||||||||||||||||||||||||||||||| ||||||||||||| ||||||||||
Sbjct: 212244 ATTTTAGGATTAATACATATGCTTTTTTTTTATTCGAAATCTGGGGATTCTATACAGAGT
212303


Query: 425    TGTAAGTTAGGCAAACTAGAATTTGGTAATAATATTTTATTCTTGGGGCGACATATGGAG
484
              ||||||||||||||||||||||||||||||||||||||||||||||||||||||||||||
Sbjct: 212304 TGTAAGTTAGGCAAACTAGAATTTGGTAATAATATTTTATTCTTGGGGCGACATATGGAG
212363


Query: 485    ATACTTTATTTCCTTTTCTTAATTATTAACGTATACCTATAAATTAACAAAGTATCTAAA
544
              ||||||||||||||||||||||||||||||||||||||||||||||||||||||||||||
Sbjct: 212364 ATACTTTATTTCCTTTTCTTAATTATTAACGTATACCTATAAATTAACAAAGTATCTAAA
212423


Query: 545    CAAAATACATAAGTGTACTCAAACTGAGTAGAATCGTCGATTAAACTTCCTTCTCCTTTT
604
              ||||||||||||||||||||||||||||||||||||||||||||||||||||||||||||
Sbjct: 212424 CAAAATACATAAGTGTACTCAAACTGAGTAGAATCGTCGATTAAACTTCCTTCTCCTTTT
212483
```

VIII212300 F

```
Query: 157    AAAAATTAAAAACAGCAAATAGTTAAATG 185
              ||||||||||||||||||||||||| |||
Sbjct: 212484 AAAAATTAAAAACAGCAAATAGTTAGATG 212512
```

```
Query: 186    AATATATTAAAGACTATTCGTTTTATTTCCCAGAGCAGCATGATTTCTTGGTTTCTTCAG
245
              ||||||||||||||||||||||| ||||||||||||||||||| ||||||||||||||||
Sbjct: 212513 AATATATTAAAGACTATTCGTTTCATTTCCCAGAGCAGCATGACTTCTTGGTTTCTTCAG
212572


Query: 246    ACTTGTTACCGCAGGGGCATTTGTCGTCGCTGTTACACCCCGTTGGGCAGCTACATGATT
305
              ||||||||||||||||||||||||||||||||||||||||||||||||||||||||||||
Sbjct: 212573 ACTTGTTACCGCAGGGGCATTTGTCGTCGCTGTTACACCCCGTTGGGCAGCTACATGATT
212632


Query: 306    TTTGGCATTGTTCATTATTTTTGCAGCTACCACATTGGCATTGGCACTCATGACCTTCAT
365
              ||||||||||||||||||||||||||||||||||||||||||||||||||||||||||||
Sbjct: 212633 TTTGGCATTGTTCATTATTTTTGCAGCTACCACATTGGCATTGGCACTCATGACCTTCAT
212692


Query: 366    TTTGGAAGTTAATTAATTCGCTGAACATTTTATGTGATGATTGATTGATTG----TACGG
421
              |||||||||||||||||||||||||||||||||||||||||||||||||||    ||| |
Sbjct: 212693 TTTGGAAGTTAATTAATTCGCTGAACATTTTATGTGATGATTGATTGATTGATTGTACAG
212752


Query: 422    TTTGTTTTTGTTAATATCTATTTCGATGACTTCTATATGATATTGCACTAACAAGAAGAT
481
              ||||||||| ||||||||||||||||||||||||||||||||||||||||||||||||||
Sbjct: 212753 TTTGTTTTTCTTAATATCTATTTCGATGACTTCTATATGATATTGCACTAACAAGAAGAT
212812


Query: 482    ATTATAATGCAATTGGTACAAGACAAGGAGTTATTTGCTTCTCTTTTATATGATTCTGAC
541
              ||||||||||||||| ||||||||||||||||||||||||||||||||||||||||||||
Sbjct: 212813 ATTATAATGCAATTGATACAAGACAAGGAGTTATTTGCTTCTCTTTTATATGATTCTGAC
212872


Query: 542    AATCCATATTGCGTTGGTAGTCTTTTTTGCTGGAACGGTTCAGCGGAAAAGACGCATCGC
601
              ||||||||||||||||||||||||||||||||||||||||||||||||||||||||||||
Sbjct: 212873 AATCCATATTGCGTTGGTAGTCTTTTTTGCTGGAACGGTTCAGCGGAAAAGACGCATCGC
212932
```

F1

```
Query: 242    TCTTTTTGCTTCTAGAAGAAATGCCAGCAAAAGAATCTCTTGACAGTGACTGACAGCAAA
301
              ||||||||||||||||||||||||||||||||||||||||||||||||||||||||||||
```

```
Sbjct: 212933 TCTTTTTGCTTCTAGAAGAAATGCCAGCAAAAGAATCTCTTGACAGTGACTGACAGCAAA
212992


Query: 302    AATGTCTTTTTCTAACTAGTAACAAGGCTAAGATATCAGCCTGAAATAAAGGGTGGTGAA
361
              ||||||||||||||||||||||||||||||||||||||||||||||||||||||||||||
Sbjct: 212993 AATGTCTTTTTCTAACTAGTAACAAGGCTAAGATATCAGCCTGAAATAAAGGGTGGTGAA
213052


Query: 362    GTAATAATTAAATCATCCGTATAAACCTATACACATATATGAGGAAAAAATAATACAAAA
421
              ||||||||||||||||||||||||||||||||||||||||||||||||| ||||||||||
Sbjct: 213053 GTAATAATTAAATCATCCGTATAAACCTATACACATATATGAGGAAAAA-TAATACAAAA
213111


Query: 422    GTGTTTTAAATACAGATACATACATGAACATATGCACGTATAGCGTCCAAATGTCGGTAA
481
              ||||||||||||||||||||||||||||||||||||||||||||| ||||||||||||||
Sbjct: 213112 GTGTTTTAAATACAGATACATACATGAACATATGCACGTATAGCGCCCAAATGTCGGTAA
213171


Query: 482    TGGGATCGGCTTACTAATTATAAAATGCATCATAGAAATCGTTGAAGTTTGCCGTAGTAA
541
              ||||||||||||||||||||||||||||||||||||||||||||||||||||||||||||
Sbjct: 213172 TGGGATCGGCTTACTAATTATAAAATGCATCATAGAAATCGTTGAAGTTTGCCGTAGTAA
213231


Query: 542    TACCCAGATTATCAGATTCCAAATCCTTGTCAATAATTATACTCCTTTGGAAAACTTCTC
601
              ||||||||||||||||||||||||||||||||||||||||||||||||||| ||||||||
Sbjct: 213232 TACCCAGATTATCAGATTCCAAATCCTTGTCAATAATTATACTCCTTTGGACAACTTCTC
213291

Query: 602    TTTCCATTAAAAAATCTGAAATCTCCTTAAATTTTAAATAGATTCTGTTCAGTTCACTAA
661
              ||||||||||||||||||||||||||||||||||||||||||||||||||||||||||||
Sbjct: 213292 TTTCCATTAAAAAATCTGAAATCTCCTTAAATTTTAAATAGATTCTGTTCAGTTCACTAA
213351
```

VIII213234 F

```
Query: 87     CGGGGAATTTCAAGAGAACATTTTTGTTCTTCGCCGA 123
              |||||||||||||||||||||||||||||||||||||
Sbjct: 213352 CGGGGAATTTCAAGAGAACATTTTTGTTCTTCGCCGA 213388


Query: 124    CTGACTATAATCTGTAACATTATTATTATCAGAGTTTCTCGCAAAATTTTGTTTTTTCTT
183
              |||||||||||||||||||||||| |||||||||||||||||||||||||||||||||||
```

```
Sbjct: 213389 CTGACTATAATCTGTAACATTATTGTTATCAGAGTTTCTCGCAAAATTTTGTTTTTTCTT
213448


Query: 184    GCTAAATCTCAGCATATATTTAATCAGATTCAAAACCTTGTTGAAACCTTTAATAGATTT
243
              ||||||||||||||||||||||||||||||||||||||||||||||||||||||||||||
Sbjct: 213449 GCTAAATCTCAGCATATATTTAATCAGATTCAAAACCTTGTTGAAACCTTTAATAGATTT
213508


Query: 244    GAAATTTCCGTTGCTATTCATTTCATCCCGTAAAAAGGATACGATAATTTCTATTTTTTT
303
              |||| |||||||||||||||||||||| ||||||||||||||||||||||||||||||||
Sbjct: 213509 GAAACTTCCGTTGCTATTCATTTCATCTCGTAAAAAGGATACGATAATTTCTATTTTTTT
213568


Query: 304    TAAAATTTCCAAAATCTTGTCATGAATCAATAGCAATTGAACATTAATCTCCTCATTTGA
363
              ||||||||||||||||||||||||||||||||||||||||||||||||||||||||||||
Sbjct: 213569 TAAAATTTCCAAAATCTTGTCATGAATCAATAGCAATTGAACATTAATCTCCTCATTTGA
213628


Query: 364    AAGATTTTTGTAAAATTCGTCATATAATATTACTTCACAACGTTGGAAAATAGCAAATGT
423
              ||||||||||||||||||||||||||||||||||||||||||||||||||||||||||||
Sbjct: 213629 AAGATTTTTGTAAAATTCGTCATATAATATTACTTCACAACGTTGGAAAATAGCAAATGT
213688


Query: 424    GATTGCTATAAAATTCTGTAAGATTTCAATAAAATGATTTGCGAATAAAAATTCTTTACC
483
              ||||||||||||||||||||||||||||||||||||||||||||||||||||||||||||
Sbjct: 213689 GATTGCTATAAAATTCTGTAAGATTTCAATAAAATGATTTGCGAATAAAAATTCTTTACC
213748


Query: 484    ATTAGAATGAAAGCGATTATTGCCGCTTGAAAATGACTTTATCGACTTTATGGGGAAGAT
543
              ||||||||||||||||||||||||||||||||||||||||||||||||||||||||||||
Sbjct: 213749 ATTAGAATGAAAGCGATTATTGCCGCTTGAAAATGACTTTATCGACTTTATGGGGAAGAT
213808


Query: 544    AAAATTAAATGTTACTGAGTAAAAAATGTGCATATTAGAAATAATTTTCATCAGATCCTT
603
              |||||||||||||| |||||||||||||||||||||||||||||||||||||||||||||
Sbjct: 213809 AAAATTAAATGTTATTGAGTAAAAAATGTGCATATTAGAAATAATTTTCATCAGATCCTT
213868
```

2. VIII215871-VIII217834

VIII213601 F

```
Query: 240    TGCA 243
              ||||
Sbjct: 215867 TGCA 215870


Query: 244    CATCTTTCAGAGTTCGAGGTCTTATTGTTGTTAGAAGAATGCTGAACTGCCATGGACAAA
303
              ||||||||||||||||||||||||||||||||||||||||| ||||||||||||||||||
Sbjct: 215871 CATCTTTCAGAGTTCGAGGTCTTATTGTTGTTAGAAGAATGTTGAACTGCCATGGACAAA
215930


Query: 304    GAGGATTCGTTTTGAACAAAAAGGAAAAAATTTGTATAAACAATGGTATTGATAAAATTT
363
              ||||||||||||||||||||||||||||||||||||||||||||||||||||||||||||
Sbjct: 215931 GAGGATTCGTTTTGAACAAAAAGGAAAAAATTTGTATAAACAATGGTATTGATAAAATTT
215990


Query: 364    AAAGTGTCTTTCCATTCTTTTCTGACTTCGTTGTCATGAAAATATAAGTCTACTGTATTA
423
              ||||||||||||||||||||||||||||||||||||||||||||||||||||||||||||
Sbjct: 215991 AAAGTGTCTTTCCATTCTTTTCTGACTTCGTTGTCATGAAAATATAAGTCTACTGTATTA
216050


Query: 424    CTCACGCCCATAGTCAAGGTTTCTAACAGACTTTCAATTTTGGTTAAATTTACTGGCAAG
483
              ||||||||||||||||||||||||||||||||||||||||||||||||||||||||||||
Sbjct: 216051 CTCACGCCCATAGTCAAGGTTTCTAACAGACTTTCAATTTTGGTTAAATTTACTGGCAAG
216110


Query: 484    TAGAAAGGAACATCTTGCAGAATATTTATCAATTTTGCTTGCGTTTCCAGTAATTTTAAA
543
              |||||||||||| |||||||||||||||||||||||||||||||||||||||||||||||
Sbjct: 216111 TAGAAAGGAACACCTTGCAGAATATTTATCAATTTTGCTTGCGTTTCCAGTAATTTTAAA
216170


Query: 544    TCGTTAGCAATTAAAGGAATGTCGTTCGTATCAATAGAGGCAGGTATCGGAGATAGGTTT
603
              ||||||||||||||||||||||||||||||||||||||||||||||||||||||||||||
Sbjct: 216171 TCGTTAGCAATTAAAGGAATGTCGTTCGTATCAATAGAGGCAGGTATCGGAGATAGGTTT
216230


Query: 604    TCAGTAGCGGGTACCATGAATGAAGACTGACCTAGAAGCGAATGTCTTGAGTAATACATT
663
              |||| |||||||||||||||||||||||||||||||||||||||||||||||||||||||
Sbjct: 216231 TCAGCAGCGGGTACCATGAATGAAGACTGACCTAGAAGCGAATGTCTTGAGTAATACATT
216290
```

```
Query: 664    TTTTTCAAAAAATGGAATAACTTGAAATCTTTTATCTGGAAGCTTAA 710
              |||||||||||||||||||||||||||||||||||||||||||||||
Sbjct: 216291 TTTTTCAAAAAATGGAATAACTTGAAATCTTTTATCTGGAAGCTTAA 216337
```

VIII216859 R

```
Query: 489    GAGACAGCTCAAATCTTCG 471
               ||||||||||||||||||
Sbjct: 216338 CAGACAGCTCAAATCTTCG 216356


Query: 470    TCTAAACTTGATTTATGATCATCATGCATGTACAGAGATTCATAAAATTTTGTGATGGCA
411
              ||||||||||||||||||||||||||||||||||||||||||||||||||||||||||||
Sbjct: 216357 TCTAAACTTGATTTATGATCATCATGCATGTACAGAGATTCATAAAATTTTGTGATGGCA
216416


Query: 410    ATAAATTTGATTGTCTCTTGGTCATAATATTCCAGGTTTATCAATGTGATTTGGCTTCGA
351
              ||||||||||||||||||||||||||||||||||||||||||||||||||||||||||||
Sbjct: 216417 ATAAATTTGATTGTCTCTTGGTCATAATATTCCAGGTTTATCAATGTGATTTGGCTTCGA
216476


Query: 350    ATCAACCTCAAATTATTCATTAATGCCGAAACATGGTTATTAATAGCCTGCTTATTGAAT
291
              ||||||||||||||||||||||||||||||||||||||||||||||||||||||||||||
Sbjct: 216477 ATCAACCTCAAATTATTCATTAATGCCGAAACATGGTTATTAATAGCCTGCTTATTGAAT
216536


Query: 290    AGGGTCATTGAATCGTTTAACAACAGCAGAAGCACAGTTAATTCGCCTAGTTTGGTCAGT
231
              |||||||||||||||||||||||||| |||||||||||||||||||||||||||||||||
Sbjct: 216537 AGGGTCATTGAATCGTTTAACAACAGTAGAAGCACAGTTAATTCGCCTAGTTTGGTCAGT
216596


Query: 230    TGAGATAAAGGAAGATCGACCGTTGAGAATGAAGGACAGGTATCACCATTTGGAAAAGAG
171
              ||||||||||||||||||||||||||||||||||||||||||||||||||||||||||||
Sbjct: 216597 TGAGATAAAGGAAGATCGACCGTTGAGAATGAAGGACAGGTATCACCATTTGGAAAAGAG
216656


Query: 170    TTATGAACAAATTCTAGTAAGAGGGTTTTGTCAAAGATTGGCAAAAATTCCTCAATATTC
111
              |||||||||||||||||||||||||||||||||||||||||||||||||| |||||||||
Sbjct: 216657 TTATGAACAAATTCTAGTAAGAGGGTTTTGTCAAAGATTGGCAAAAATTCTTCAATATTC
216716


Query: 110    AGATTGTTAAGGGATTCAGAGTTTAGTGTCTCAATAATTATTGCTTTTGGTGGGAAGTTA 51
```

```
               ||||||||||||||||||||||||||||||||||||||||||||||||||||||||||||
Sbjct: 216717  AGATTGTTAAGGGATTCAGAGTTTAGTGTCTCAATAATTATTGCTTTTGGTGGGAAGTTA
216776
```

VIII216894 R

```
Query: 89      ACTTTGCAAACTTCCATATATTGTTGGTGATTTTTATCGTTAGGAGGACT 40
               ||||||||||||||||||||||||||||||||||||| ||||||||||||
Sbjct: 216777  ACTTTGCAAACTTCCATATATTGTTGGTGATTTTTATTGTTAGGAGGACT 216826
```

VIII216763 F

```
Query: 38      TACTTTGAACTGATTAATATGGC 60
               |||||||||||||||||||||||
Sbjct: 216827  TACTTTGAACTGATTAATATGGC 216849


Query: 61      AGATATCGTGCAAAATATTATAGAACTTGAAAAGATAATGATCTCTAAAAATGATGAAGT
120
               ||||||||||||||||||||||||||||||||||||||||||||||||||||||||||||
Sbjct: 216850  AGATATCGTGCAAAATATTATAGAACTTGAAAAGATAATGATCTCTAAAAATGATGAAGT
216909


Query: 121     TTTTAGTGAAAAGGTAGTTGGGGGTATCCTTCAACGGTTTCACTTGGACTAAACTTTGCA
180
               ||||||||||||||||||||||||||||||||||||||||||||||||||||||||||||
Sbjct: 216910  TTTTAGTGAAAAGGTAGTTGGGGGTATCCTTCAACGGTTTCACTTGGACTAAACTTTGCA
216969


Query: 181     CATCTGTTATTGTGAGCGCTGTAAAGTATTTTTGTGAGGACTGAGAAGTCCTTGAAATCT
240
               |||||||||||||||||||||||||||||||||||||||||||||||||||||||| |||
Sbjct: 216970  CATCTGTTATTGTGAGCGCTGTAAAGTATTTTTGTGAGGACTGAGAAGTCCTTGAAGTCT
217029


Query: 241     GATGATCAAATCCGGCTTTTTTACCGTCAACCTTGCCTTTCTTAGAATTCTCACCTGTTT
300
               ||||||||||||||||| ||||||||||||||||||||||||||||||||||||||||||
Sbjct: 217030  GATGATCAAATCCGGCTCTTTTACCGTCAACCTTGCCTTTCTTAGAATTCTCACCTGTTT
217089


Query: 301     TGTTAATTTTGTTTGCCGTGGTATTGTCGGCATTTAAATTCGATTTTTCTTTCAAACTTT
360
               ||||||||||||||||||||||||||||||||||||||||||||||||||||||||||||
Sbjct: 217090  TGTTAATTTTGTTTGCCGTGGTATTGTCGGCATTTAAATTCGATTTTTCTTTCAAACTTT
217149
```

```
Query: 361    TCAGTTGCAAGCTTTGTAAATCAAGCAATTTGTTTTTTAAAAAGTCTATCTCCTTGATTA
420
              ||||||||||||||||||||||||||||||||||||||||||||||||||||||||||||
Sbjct: 217150 TCAGTTGCAAGCTTTGTAAATCAAGCAATTTGTTTTTTAAAAAGTCTATCTCCTTGATTA
217209


Query: 421    ATACTTCCTCTTGAGTAAACGGAGAAGTCATTAGATCGAAAACGGCCGAGTCTTGATTGA
480
              ||||||||||||||||||||||||||||||||||||||||||||||||||||||||||||
Sbjct: 217210 ATACTTCCTCTTGAGTAAACGGAGAAGTCATTAGATCGAAAACGGCCGAGTCTTGATTGA
217269


Query: 481    TACCAACATTAAAAGAGTATGGGTTATCTGTATAGTCAAAACTTTGAGCGTTAACCAGTT
540
              ||||||||||||||||||||||||||||||||||||||||||||||||||||||||||||
Sbjct: 217270 TACCAACATTAAAAGAGTATGGGTTATCTGTATAGTCAAAACTTTGAGCGTTAACCAGTT
217329


Query: 541    GTAATCGTGTGTTATATTCTCTAATCTGATCCATGGAGGGCACATCTGGACTATCGCTTC
600
              ||||||||||||||||||||||||||||||||||||||||||||||||||||||||||||
Sbjct: 217330 GTAATCGTGTGTTATATTCTCTAATCTGATCCATGGAGGGCACATCTGGACTATCGCTTC
217389


Query: 601    TCACAATAGTGTTTCCATGAACATTACTACTAACGGTACTACTATTCTGACGAGGAGCAG
660
              ||||||||||||||||||||||||||||||||||||||||||||||||||||||||||||
Sbjct: 217390 TCACAATAGTGTTTCCATGAACATTACTACTAACGGTACTACTATTCTGACGAGGAGCAG
217449
```

VIII218008 R

```
Query: 551    TATTATT 545
              |||||||
Sbjct: 217450 TATTATT 217456


Query: 544    ATTGGTGTTATTATTCTGGTAGCTAGCGGCCTTGGTCCACTGCATCGCAGTATCCACAGA
485
              ||||||||||||||||||||||||||||||||||||||||||||||||||||||||||||
Sbjct: 217457 ATTGGTGTTATTATTCTGGTAGCTAGCGGCCTTGGTCCACTGCATCGCAGTATCCACAGA
217516


Query: 484    GGGGTTGAAAGAGAAGTTGGATGCCTCTATCGACGTTTGCATAATCGGATACTGCGTTTG
425
              ||||||||| ||||||||| ||||||||||||||||||||||||||||||||||||||||
Sbjct: 217517 GGGGTTGAACGAGAAGTTGAATGCCTCTATCGACGTTTGCATAATCGGATACTGCGTTTG
217576
```

```
Query: 424    AATCATTACGTTTTTCTGATTTACACCATTTCCCTGACTAAAATGGTTGGAACCTTGGCC
365
              ||||||||||||||||||||||||||| ||||||||||||||||||||||||||||||||
Sbjct: 217577 AATCATTACGTTTTTCTGATTTACACCGTTTCCCTGACTAAAATGGTTGGAACCTTGGCC
217636


Query: 364    ATTGCCGTGCGTGGACATCCCGGACGCAGAGGGCACAGCGACCATCTTACCAGGTCCATC
305
              ||||||||||||||||||||||||||||||||||||||||||||||||||||||||||||
Sbjct: 217637 ATTGCCGTGCGTGGACATCCCGGACGCAGAGGGCACAGCGACCATCTTACCAGGTCCATC
217696


Query: 304    TGGATAAAAACAGTCCGGCTTGTTATACTTGACGCAATTCCCACATATCGGTTTTGCCCT
245
              ||||||||||||||||||||||||||||||||||||||||||||||||||||||||||||
Sbjct: 217697 TGGATAAAAACAGTCCGGCTTGTTATACTTGACGCAATTCCCACATATCGGTTTTGCCCT
217756


Query: 244    GTCGCACCCGATCTTTCTCTTCCTGCATTGGGTGCAAGCAGGCGGCTTCCTCACTTTTCT
185
              ||||||||||||||||||||||||||||||||||||||||||||||||||||||||||||
Sbjct: 217757 GTCGCACCCGATCTTTCTCTTCCTGCATTGGGTGCAAGCAGGCGGCTTCCTCACTTTTCT
217816



Query: 184    CACTTGCATGTCCATCAT 167
              ||||||||||||||||||
Sbjct: 217817 CACTTGCATGTCCATCAT 217834
```

SNPs between DTY3 and S288c

Sequenced interval	Coordinate(s)	SNP in DTY3	SNP in S288c
VIII210848-VIII213868			
	211693	A	G
	211701	A	G
	211825	A	G
	211967	A	G
	211984	G	T
	212279	A	G
	212293	T	C

	212509	A	G
	212536	T	C
	212556	T	C
	212744-212747	4 bp deletion	ATTG
	212751	G	A
	212762	G	C
	212828	G	A
	213097-213101	6 A’s	5 A’s
	213157	T	C
	213283	A	C
	213413	A	G
	213513	T	C
	213536	C	T
	213823	C	T
VIII215871-VIII217834			
	215192	C	T
	216123	T	C
	216235	T	C
	216563	C	T
	216707	C	T
	216814	C	T
	217026	A	G
	217047	T	C
	217626	A	C
	217536	G	A
	217604	A	G
